# Novel 1-(1-Arylimiazolin-2-Yl)-3-Arylalkilurea Derivatives with Modulatory Activity on Opioid MOP Receptors

**DOI:** 10.3390/molecules29030571

**Published:** 2024-01-24

**Authors:** Dominik Straszak, Sylwia Woźniak, Agata Siwek, Monika Głuch-Lutwin, Marcin Kołaczkowski, Aldona Pietrzak, Bartłomiej Drop, Dariusz Matosiuk

**Affiliations:** 1 Department of Synthesis and Chemical Technology of Pharmaceutical Substances, Faculty of Pharmacy, Medical University, Chodzki 4A, 20-093 Lublin, Poland; dominik.straszak@gmail.com (D.S.); sylwia.wozniak@umlub.pl (S.W.); 2 Faculty of Pharmacy, Jagiellonian University Medical College, Medyczna 9, 30-688 Kraków, Poland; agat.siwek@uj.edu.pl (A.S.); monika.gluch@uj.edu.pl (M.G.-L.); marcin.kolaczkowski@uj.edu.pl (M.K.); 3 Department of Dermatology, Venereology, and Paediatric Dermatology, Faculty of Medicine, Medical University, Staszica 11, 20-080 Lublin, Poland; aldona.pietrzak@umlub.pl; 4 Department of Medical Informatics and Statistics, Medical University, Jaczewskiego 4, 20-090 Lublin, Poland; bartlomiej.drop@umlub.pl

**Keywords:** MOP receptor, OP3 receptor, allosterism, β-arrestin, 1-aryl-2-aminoimidazoline-2

## Abstract

μ-opioid receptor ligands such as morphine and fentanyl are the most known and potent painkillers. However, the severe side effects seen with their use significantly limit their widespread use. The continuous broadening of knowledge about the properties of the interactions of the MOP receptor (human mu opioid receptor, OP3) with ligands and specific intracellular signaling pathways allows for the designation of new directions of research with respect to compounds with analgesic effects in a mechanism different from classical ligands. Allosteric modulation is an extremely promising line of research. Compounds with modulator properties may provide a safer alternative to the currently used opioids. The aim of our research was to obtain a series of urea derivatives of 1-aryl-2-aminoimidazoline and to determine their activity, mechanism of biological action and selectivity toward the MOP receptor. The obtained compounds were subjected to functional tests (cAMP accumulation and β-arrestin recruitment) in vitro. One of the obtained compounds, when administered alone, did not show any biological activity, while when co-administered with DAMGO, it inhibited β-arrestin recruitment. These results indicate that this compound is a negative allosteric modulator (NAM) of the human MOP receptor.

## 1. Introduction

Opioid receptors are rhodopsin-like G protein-coupled receptors (GPCRs). They play an important role in the neuromodulation of the nociception process at all levels of neuronal pathways, both at the central and peripheral levels [1]. The main molecular target of opioid drugs, such as morphine, fentanyl or oxycodone used in pain therapy, is the μ-opioid receptor. Its activation triggers a cascade of intracellular signaling pathways related to G proteins, arrestin and scaffolding proteins. The key to inducing analgesia is, among others, the inhibition of adenylate cyclase, VGCCs and the opening of GIRK channels [2]. Despite the high effectiveness of opiates in pain control, their widespread use is limited by the incidence of side effects, including nausea, vomiting and constipation, as well as the life-threatening depression of the respiratory center [3,4]. Additionally, the use of opioids leads to an imbalance and the structural remodeling of the reward system and, consequently, addiction [5]. For these reasons, there are ongoing studies on the discovery and development of analgesic drugs, the safety profile of which will be more favorable than that of the opioids currently used.

One of the most promising strategies developed in the search for novel opioid analgesics is allosteric modulation. Positive allosteric modulators (PAMs) stimulate the orthosteric pocket of the MOP receptor by interacting with the allosteric site. Allosteric modulators have characteristic properties that distinguish them from other types of ligands. Particularly important in the pharmacological context is their high selectivity. In contrast to the orthosteric pocket, which is highly conserved in many related receptors, the allosteric site can be located in various areas and exhibits high structural variability, which allows for selectivity toward particular types and even subtypes of GPCRs. In addition, the allosteric modulator, stabilizing the particular conformational state of the receptor, has a different effect on the affinity and/or efficacy of individual orthosteric ligands. What is more, the modulator can induce bias receptor signaling, triggering only the selected signaling pathway [6]. Thus, allosteric modulators are an extremely attractive target for the search of new compounds with specific biological activity. They make it possible to obtain drugs with high receptor selectivity and limited side effects, especially compared to some that are already present or have just been introduced to the market, like PAM ligands for the calcium-sensing receptor (cinacalcet), GABA_A_ (benzodiazepines), NAM ligands for chemokine receptor CCR5 (maraviroc), mGluR5 (fenobam, raseglurant, dipraglurant and VU-29) or muscarinic receptor (M1 PAM – MK7622), or are in advanced stages of clinical trials (M2/M4 PAM, CB2 PAM, 5-HT_2C_ PAM, CCR5 NAM, CXCR3 NAM and β_2_A NAM).

**Figure 1:**
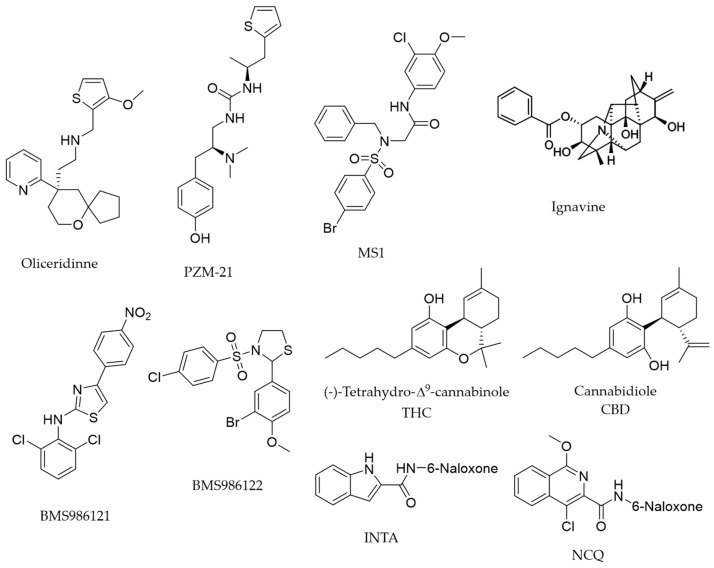
Structures of some positive, negative, allosteric and biased ligands of the opioid MOP receptor.

Currently, only a few (Figure 1) exogenous PAMs for the MOP receptor are known: BMS986121, BMS986122 [7], ignavine [8], MS1 and some of its analogs [9]; and NAMs (THC and CBD [10], cannabis secondary metabolites) and two dualsteric ligands, INTA and NCQ [11,12]. Developing biased agonists of the MOP receptor could be very promising due to their activation of only one pathway—the G protein pathway, without β-arrestin-2 involvement. Oliceridine was first mentioned in the literature [13,14] and accepted by FDA [15]. Unfortunately, after prolonged administration, it produced side effects similar to those of typical MOP receptor agonists, not distinguishing between both pathways and building up to drug tolerance and abuse [16]. A new, very promising compound from that group is PZM-21 [17], which is currently undergoing clinical tests. In recent years, Matosiuk et al. have obtained a number of 1-aryl-imidazoline-2-yl urea derivatives with interesting antinociceptive properties [18,19]. Some of them have MOP receptor affinity at the micromolar level, which was evaluated using binding assay tests. On the other hand, behavioral tests (‘hot plate’ and ‘writhing test’) carried out on mice confirmed their strong analgesic effects, partially reversible after the administration of naloxone. Their administration in under-threshold doses increased significantly the effect of morphine (lower than 5 mg/kg) or DAMGO (lower than 0.5 mg/kg) [20], producing strong antinociception. Importantly, these compounds did not cause depression of the respiratory center in laboratory animals. The obtained results indicate that they can interact with the MOP receptor in a different way than its classical and non-classical ligands [19].

**Scheme 1:**
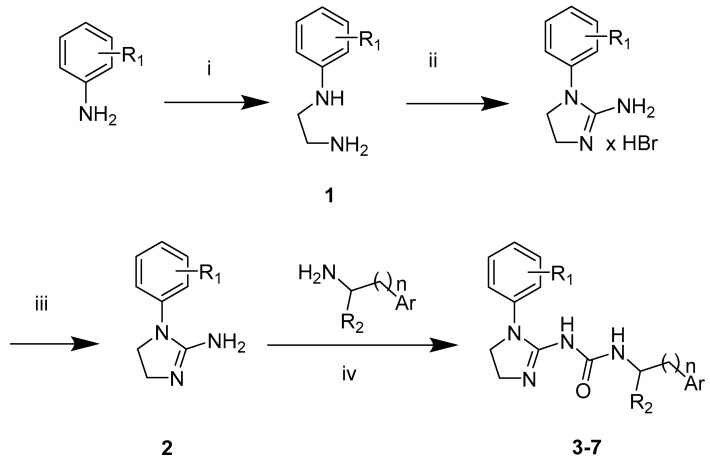
Synthesis of the title 1-(1-arylimidazoline) urea derivatives. i = aziridine, AlCl_3_, toluene; or CH_2_O, NaCN, NH_4_OH. ii = BrCN, 2-propanol, room temp. iii = NaOH. iv = triphosgene, EtN_3_, toluene, room temperature for 2 h and then 100 °C for 5 h. Physicochemical and spectral data are presented in the Supplementary Materials.

In this paper, we present the results of the synthesis of 1-arylimidazoline urea derivatives (Scheme 1, Table 1.) and in vitro examination of their effect on the MOP receptor, including functional tests (cAMP inhibition and β-arrestin recruitment assays). 1-aryl-2-aminoimidazoline hydrobromides, which were necessary for synthesis, were obtained from respective N-aryl-1,2-diaminoethanes and cyanogen bromide according to our previous publication [17]. 1,2-Diaminoethanes were obtained through the Lehmann method [21] with the use of aziridine and anhydrous aluminum trichloride or through the Strecker method with the Takeda modification [22,23] (for methoxy derivatives) with the use of methanal, sodium cyanide and a solution of ammonia. Commercially available arylakylamines were bought. Other branched arylalkylamines were synthesized from the beginning. 1-Arylpropyl-2-amine derivatives (**6a**–**r**) were obtained from nitroethane and aromatic aldehydes via Henry’s method (acetic acid, ammonium acetate, catalytic reduction with hydrogen in the presence of Raney nickel) [24,25] or heteroaromatic aldehydes (methanol in the presence of butyl amine, reduction with lithium aluminum tetrahydride) [26]. Benzylideneacetone derivatives were obtained via the Cleisen–Schmidt method [27]. Further on, they were transformed into oximes [28]. The reduction of oximes with hydrogen and Raney nickel or with lithium aluminum tetrahydride led to respective 1-arylbutyl-3-amines (**7a**–**p**) [26,29]. The presence of the urea moiety diminished the solubility of obtained compounds, whereas the introduction of longer and branched aliphatic linkers improved it. Therefore, from among 54 synthesized derivatives, only 22 were selected and tested in vitro. The selection was made based on the verification of solubility via spectrophotometric methods. Less soluble derivatives were omitted due to the production of unreliable results. The methodology of that spectral technique was described in our previous publication from this series [19].

## 2. Results

**Figure 2:**
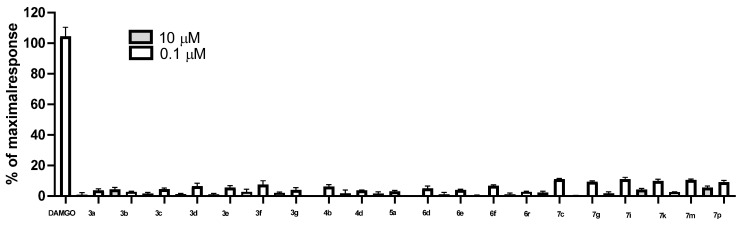
Screening for agonistic properties at MOP receptor performed for tested compounds at 10 and 0.1 µM concentrations.

**Figure 3:**
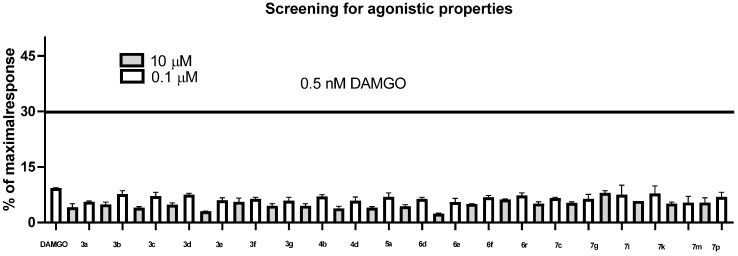
Screening for allosteric properties at opioid mu receptor performed for tested compounds at 10 and 0.1 µM concentration.

The tested compounds (in agonist mode, Table 2, Figure 2) exhibited no effect on cAMP accumulation both in 0.1 and 10 μM concentrations or on allosteric properties at both concentrations (Table 3, Figure 3).

**Figure 4:**
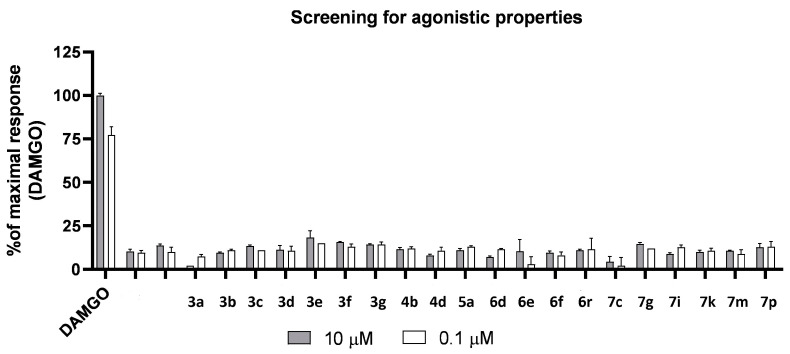
Screening for agonistic properties at opioid mu receptor performed for tested compounds at 10 µM and 0.1 µM concentrations.

**Figure 5:**
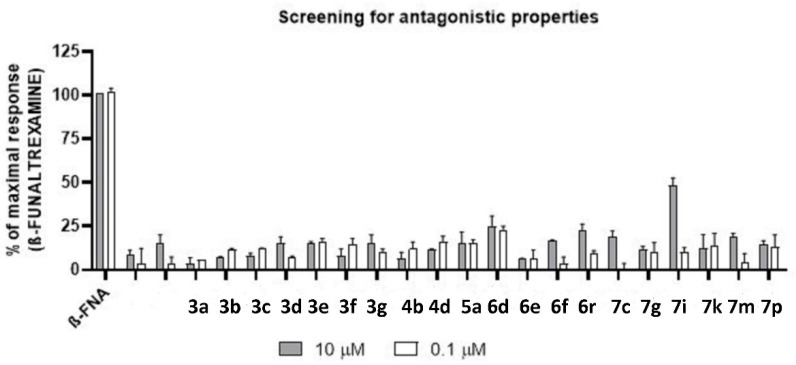
Screening for antagonistic properties at opioid mu receptor performed for tested compounds at 10 µM and 0.1 µM concentrations.

The tested compounds (in agonist and antagonist mode, Table 4, Figure 4 and Figure 5) had no effect on β-arrestin recruitment, except for compound **7i**, for which its Emax% at a concentration of 10 µM in the antagonistic mode was 48%.

### Structure–Activity Relationship

In the structure of the investigated compounds, two distinct fragments can be recognized. One is based on an imidazoline ring with an aromatic substituent in position 1. The imidazoline system makes a rigid element for the structure and, even though it contains nitrogen atoms, shows no basicity due to its incorporation into the urea moiety and the very strong polarization effect of the carbonyl group. Therefore, activity is expected to be affected only by substituents in the aromatic ring. Pi–Pi interactions or hydrophobic interactions produced by the aromatic ring and its substituents can be the main effects taken into account. The second fragment is an aromatic-aliphatic moiety with different lengths of the aliphatic linker and its branching. The linker, especially the branched one, was introduced to enhance solubility and bioavailability. The aromatic ring at the end of the linker plays a similar role to the imidazoline substituent—it is responsible for generating hydrophobic interactions. It was confirmed that compounds with a short linker (one or two carbon atoms) (**3a**–**g**, **4a**–**g**, **5a**–**g**) exhibited very weak activity and no differences between agonist and antagonist modes of action. For three- (**6a**–**r**) and especially four-carbon (**7a**–**p**) branched linkers, more profound activity was confirmed for derivatives containing 1-(4-chlorophenyl) (three carbon atom—**6d**, **6f**, **6r**) or 1-(4-methylphenyl)imidazoline (four carbon atoms—**7c**, **7i**, **7m**) moiety and 4-chloro- (**6d**) or 4-fluorosubstituted (**6f**, **7i**) phenyl rings. Significant activity was also confirmed for derivatives containing thiophene-2-yl (**7m**) or 5-methyl-thiophene-2-yl substituents (**6r**). It is interesting that similar patterns, namely the presence of the urea moiety, branched aliphatic linker and thiophen-2-yl aromatic substituent, can be observed in the structure of PMZ-21 [17].

## 3. Materials and Methods

### 3.1. Chemistry

Chemicals were purchased from Sigma-Aldrich (Saint Louis, MO, USA) or Merck KGaA (Damstadt, Germany) and used without further purification. Melting points were measured using a Boetius apparatus and are given uncorrected. ^1^H (600 MHz) and ^13^C (151 MHz) NMR spectra were recorded on a Bruker Avance 600 apparatus (Bruker BioSpin GmbH, Rheinstetten, Germany) in DMSO-*d*_6_ with TMS as the internal standard. Mass analyses were carried out using the atmospheric pressure chemical ionization (APCI) mass spectrometer microTOF-Q II (Bruker Daltonics, Bremen, Germany). TLC was performed on commercial Merck silica gel F254 plates with hexane/ethyl acetate (7:3) eluent system; visualization was performed under UV light with λ = 254 nm. Flash chromatography was performed using a puriFlash 430 apparatus using commercial column PF-15SIHP puriFlash (Interchim, Montluçon, France). The purity of the compounds based on spectral data was over 95%.

### 3.2. General Procedure

Quantities of 0.0083 mol (2.46 g) of triphosgene and 30 cm^3^ of dry toluene were placed in a round-bottom flask equipped with a reflux condenser and a magnetic stirrer. A solution of 0.025 mol of arylalkylamine and 0.025 mol (2.53 g) of triethylamine in 30 cm^3^ of dry toluene was slowly added dropwise to the mixture. The mixture was stirred for 2 h at room temperature; then, a solution of 0.025 mol of 1-aryl-2-aminoimidazoline-2 and 0.025 mol (2.53 g) of triethylamine in 30 cm^3^ of dry toluene was slowly added dropwise and heated at 100 °C for 5 h. Triethylamine hydrochloride (reaction by-product) was filtered out, the solvent of the filtrate was distilled off in a vacuum evaporator and the yellow-brown oily residue was dissolved in a small amount of propan-2-ol. The obtained white precipitate of 1-(1-arylimidazoline) urea derivative was dried and purified via flash chromatography using a puriFlash 430 apparatus and a PF-15SIHP puriFlash column. Hexane and ethyl acetate were used as the eluent system in a 7:3 volume ratio.

### 3.3. Preparation of Solutions of Test and Reference Compounds

We prepared 10^−2^ M stock solutions of the test and reference compounds by weighing and dissolving min. 1 mg of each compound tested in a relevant volume of DMSO (dimethyl sulfoxide). Next, we used a vortex to stir the compound solutions, which then stood for 15 min in an ultrasonic water bath. We used an epMotion 5070 automated pipetting system (Eppendorf, Hamburg, Germany) to prepare serial dilutions in PBS (phosphate buffered saline). We checked for any opalescence or precipitation before starting the assays. We used DAMGO, β-FNA, and morphine as reference substances to assess the compounds in terms of the μ-opioid receptor affinity and intrinsic activity. The assays were carried out in two independent experiments, in duplicates.

### 3.4. Functional Assays

#### 3.4.1. cAMP Ultra LANCE Assay

In order to measure the μ-opioid receptor activity, we monitored the activity of adenylyl cyclase using c expressing OP3. The cells were thawed and then re-suspended in a stimulation buffer at 2 × 10^5^ cells/mL (HBSS, 5 mM HEPES, 0.5 IBMX, 0.1% BSA [pH 7.4]). Next, we added 10 μL of cell suspension with 10 μM forskolin to the compounds tested. The samples were subsequently transferred onto a white opaque 96-well half-area assay plate. An agonist assay was performed for a group of twenty newly investigated compounds (**3a**–**g**, **4b**, **4d**, **5a**, **6d**–**f**, **6r**, **7c**, **7g**, **7i**, **7k**, **7m**, **7p**) and reference compounds in the range of concentrations from 10^−5^ M to 10^−7^ M. The antagonist response was performed at the same range of concentrations as the agonist assay (10^−5^ M to 10^−7^ M). In this case, as a reference agonist, DAMGO was used in four concentrations: EC20 (1 nM), EC50 (4.6 nM), EC80 (18 nM) and EC87 (30 nM). The EC values were experimentally obtained from the curve of DAMGO and were calculated using GraphPad Software v. 8.0.

We added the agonist at the same time. However, with the antagonist assay, the reference agonist was added after a 30 min pre-incubation with the compounds. Then, we performed cell stimulation (30 min) at room temperature (22 °C). We measured cAMP by means of a homogeneous TR-FRET immunoassay using the LANCE Ultra cAMP kit (PerkinElmer, Waltham, MA, USA). We added, mixed and incubated 10 μL of ULight-anti-cAMP Tracer Working Solution and 10 μL of Eu cAMP Tracer Working Solution for 1 h. We used an EnVision microplate reader (PerkinElmer, USA) to read the TR-FRET signal. Prism 6.0 (GraphPad Software) was used to calculate the data by generating dose–response curves—inhibition (IC50) or three-parameter dose–response curves (EC50).

#### 3.4.2. β-Arrestin Tango Method

In order to measure the μ-opioid receptor activity, we monitored β-arrestin recruitment using Life Technologies’ cryopreserved U2OS cells expressing the MOP receptor. This line contains a TEV protease site with a linked human MOP (Opioid Receptor Mu) 1 receptor and a Tango GPCR-bla U2OS parental cell line with an integrated Gal4-VP16 transcription factor. A β-arrestin/TEV protease fusion protein and the beta-lactamase reporter gene are stably expressed by the parental line under the control of a UAS response element. The cells were thawed and then re-suspended in the DMEM high-glucose medium and GlutaMAX, 0.1 mM non-essential amino acids (NEAAs), 10% charcoal-stripped fetal bovine serum (FBS), 25 mM HEPES and streptomycin 100 μg/mL/penicillin 100 U/mL (antibiotics) at 3.125 × 10^5^ cells/mL. The samples were subsequently transferred onto a black view 384-well assay plate and incubated for 16 h. For the measurement of antagonistic activity, cells were preincubated with antagonists and tested compounds for 30 min before the addition of reference μ-opioid agonist DAMGO in EC80 (15 nM).

Afterwards, we added 8 μL of the LiveBLAzer TR-FRET B/G substrate (Invitrogen, Waltham, MA, USA) with solution D per well to obtain a final 1 μM concentration. Next, we incubated the plates in the dark at room temperature for 2 h. After incubation, we used FluoStar OPTIMA BMG Labtech (Ortenberg, Germany) with emission wavelengths at 440 nm and 530 nm and excitation at 410 nm to read the microplates.

Data were calculated as a percentage of the maximal response of the control (DAMGO or β-funaltrexamine) at 10 μM and 0.1 μM concentration using Prism 6.0 (GraphPad Software).

#### 3.4.3. Reagents and Materials

β-arrestin Tango™ OPRM1-bla U2OS DA Assay Kit, Life Technologies, Carlsbad, CA, USA, K1599; β-Funaltrexamine hydrochloride (β-FNA), 0926; calcium- and magnesium-free PBS, Gibco, New York, NY, USA, 14190144; cAMP LANCE Ultra cAMP Detection Kit, PerkinElmer, TRF0263; cAMP Zen, Human μ-opioid (OP3) Receptor, Frozen Cells, PerkinElmer, ES-542-CFDAMGO, Sigma-Aldrich, St. Louis, MO, USA, E7384; Dimethyl sulfoxide CZDA, ≥99.7%, POCh, 363550117; DMEM, Life Technologies, 10569; Elmer, 6007460; EDTA disodium salt dihydrate for mol. biol., ≥99%, Sigma-Aldrich, E5134; FBS dialyzed, Life Technologies, 26400044; Fetal Bovine Serum, charcoal stripped, USDA-approved regions, Life Technologies, 12676; Filtermat B, GF/B, PerkinElmer, 1450-442; Forskolin, Sigma-Aldrich, F6886; Ham’s F-12 Nutrient Mix, GlutaMAX™ Supplement, Life Technologies, 31765; HEPES, Gibco, H0887; IBMX, Sigma-Aldrich, I-5879; L-Glutamine, Gibco, 25030081; MeltiLex, for Microbeta Filters, PerkinElmer, 1450-441; Membrane Target System; human μ-opioid receptor, PerkinElmer, ES-542-M400UA; Non-essential amino acids (NEAA), Gibco, 11140; Penicillin/Streptomycin (antibiotics), Life Technologies, 15140; Sodium Pyruvate, Gibco, 25200072; Standard HBSS (with CaCl_2_ and MgCl_2_), Gibco, 14025; Trizma^®^ base BioPerformance Certified ≥ 99.0%, Sigma-Aldrich, T6066; Trizma^®^ hydrochloride BioPerformance Certified ≥ 99.0%, Sigma-Aldrich, T5941; Trypsin-EDTA, Gibco, 25200072; ViewPlate-384 Black, Optically Clear Bottom, Tissue Culture Treated, Sterile, 384-Well, Perkin; 1/2 AREAPLATE-96, White, 199 μL volume, 1/2 well, PerkinElmer, 6005560; [3H]-DAMGO, 250 μCi/mL, PerkinElmer, NET902250UC; 96 Polypropylene Microplate, U bottom, clear, Greiner Bio-One, 650201.

## 4. Conclusions

The synthesized 1-aryl-2-aminoimidazoline-2 derivatives were examined in functional tests. To determine the possible modes of their interactions with MOP, their effects on two independent intracellular signaling pathways were determined via cAMP inhibition and β-arrestin recruitment assays. In the first assay, tested compounds administered in the presence of the full opioid agonist DAMGO showed no significant effect on cAMP levels at any tested concentration. Interestingly, all tested compounds revealed several times higher activity when used in the concentration of 10^−7^ M than in the concentration of 10^−5^ M. In β-arrestin recruitment tests, none of the compounds administered alone showed biological activity. When administered in the presence of DAMGO, 1-[1-(4-methylphenyl)imidazolin-2-yl]-3-[4-(4-fluorophenyl)but-2-yl]urea (**7i**) exhibited significant antagonistic properties, and its Emax in relation to β-funaltrexamine was 48% at the used concentration of 10 µM. This lack of activity when administered by itself and the inhibition efficacy of DAMGO suggest that **7i** is a negative allosteric modulator (NAM) of μ-opioid arrestin pathways. The obtained results indicate that imidazoline derivatives interact with MOP in a different way than classic ligands; therefore, further studies are necessary to fully define their mechanism of action. Biological evaluation may contribute to the discovery of new compounds with modulating potential.

## Figures and Tables

**Table 1:** List of synthesized urea derivatives.

Compound Yield	R1	R2	n	Ar
**3a** 51%	H	H	0	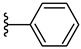
**3b** 31%	2-CH_3_	H	0	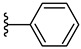
**3c** 41%	3-CH_3_	H	0	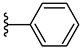
**3d** 48%	4-CH_3_	H	0	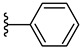
**3e** 32%	2-Cl	H	0	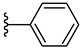
**3f** 44%	3-Cl	H	0	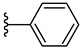
**3g** 56%	4-Cl	H	0	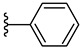
**4a** 58%	H	CH_3_	0	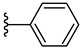
**4b** 34%	2-CH_3_	CH_3_	0	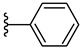
**4c** 46%	3-CH_3_	CH_3_	0	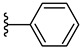
**4d** 39%	4-CH_3_	CH_3_	0	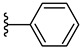
**4e** 46%	2-Cl	CH_3_	0	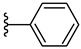
**4f** 42%	3-Cl	CH_3_	0	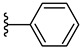
**4g** 51%	4-Cl	CH_3_	0	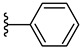
**5a** 49%	H	H	1	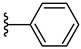
**5b** 49%	2-CH_3_	H	1	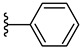
**5c** 45%	3-CH_3_	H	1	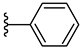
**5d** 51%	4-CH_3_	H	1	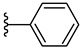
**5e** 32%	2-Cl	H	1	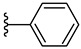
**5f** 41%	3-Cl	H	1	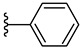
**5g** 48%	4-Cl	H	1	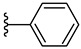
**6a** 41%	4-CH_3_	CH_3_	1	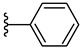
**6b** 38%	4-Cl	CH_3_	1	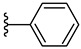
**6c** 46%	4-CH_3_	CH_3_	1	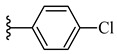
**6d** 46%	4-Cl	CH_3_	1	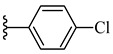
**6e** 54%	4-CH_3_	CH_3_	1	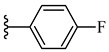
**6f** 44%	4-Cl	CH_3_	1	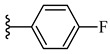
**6g** 39%	4-CH_3_	CH_3_	1	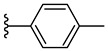
**6h** 36%	4-Cl	CH_3_	1	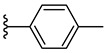
**6i** 32%	4-CH_3_	CH_3_	1	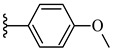
**6j** 44%	4-CH_3_	CH_3_	1	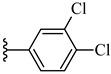
**6k** 45%	4-Cl	CH_3_	1	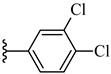
**6l** 37%	4-CH_3_	CH_3_	1	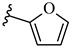
**6m** 42%	4-Cl	CH_3_	1	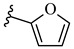
**6n** 33%	4-CH_3_	CH_3_	1	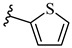
**6o** 39%	4-Cl	CH_3_	1	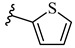
**6p** 29%	4-CH_3_	CH_3_	1	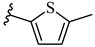
**6r** 31%	4-Cl	CH_3_	1	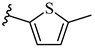
**7a** 34%	4-OCH_3_	CH_3_	2	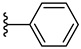
**7b** 42%	4-Cl	CH_3_	2	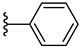
**7c** 38%	4-CH_3_	CH_3_	2	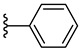
**7d** 32%	4-OCH_3_	CH_3_	2	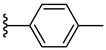
**7e** 30%	4-CH_3_	CH_3_	2	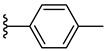
**7f** 39%	4-OCH_3_	CH_3_	2	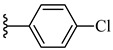
**7g** 45%	4-CH_3_	CH_3_	2	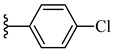
**7h** 42%	4-OCH_3_	CH_3_	2	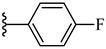
**7i** 41%	4-CH_3_	CH_3_	2	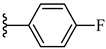
**7j** 37%	4-CH_3_	CH_3_	2	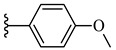
**7k** 37%	4-CH_3_	CH_3_	2	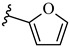
**7l** 39%	4Cl	CH_3_	2	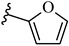
**7m** 31%	4-CH_3_	CH_3_	2	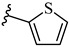
**7n** 42%	4-Cl	CH_3_	2	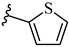
**7o** 33%	4-CH_3_	CH_3_	2	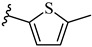
**7p** 29%	4-Cl	CH_3_	2	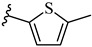

**Table 2:** The effect of tested compounds on forskolin-induced cAMP levels in CHO cells stably expressing MOP receptor (OP3). Results are normalized to the maximal response induced by the reference OP3 receptor agonist—DAMGO. (n.d.—not determined).

Compound	Percentage of Maximal Response at Tested Concentrations (% ± SEM)	
	10 μM	0.1 μM
DAMGO	n.d.	104.5 ± 5.9
**3a**	0.8 ± 1.5	3.8 ± 0.9
**3b**	4.4 ± 1.2	2.8 ± 0.5
**3c**	1.6 ± 0.8	4.6 ± 0.7
**3d**	1.3 ± 0.4	6.6 ± 1.8
**3e**	1.1 ± 0.6	5.6 ± 1.3
**3f**	2.8 ± 1.6	7.5 ± 2.6
**3g**	2.0 ± 0.6	4.1 ± 1.5
**4b**	0.0 ± 0.3	6.3 ± 1.2
**4d**	1.8 ± 2.2	3.7 ± 0.2
**5a**	1.7 ± 1.0	3.1 ± 0.7
**6d**	0.0 ± 0.1	5.0 ± 1.6
**6e**	1.1 ± 1.3	4.0 ± 0.4
**6f**	0.1 ± 0.7	6.8 ± 0.5
**6r**	1.1 ± 0.9	2.7 ± 0.4
**7c**	2.4 ± 0.9	11.0 ± 0.5
**7g**	0.1 ± 0.3	9.4 ± 0.5
**7i**	1.8 ± 1.0	11.1 ± 1.2
**7k**	4.2 ± 0.8	9.9 ± 1.2
**7m**	2.6 ± 0.3	10.5 ± 0.7
**7p**	5.5 ± 1.0	9.2 ± 1.1

**Table 3:** Effect of tested compounds on cAMP levels in CHO-hOP3R cells stimulated with 0.5 nM DAMGO and 10 µM forskolin. Results are normalized to the maximal response induced by the saturating concentration of DAMGO.

Compound	Percent of Maximal Response at Tested Concentration (% ± SEM)	
	10 μM	0.1 μM
Vehicle	9.3 ± 0.1	
**3a**	4.1 ± 1.0	5.6 ± 0.2
**3b**	4.9 ± 0.6	7.7 ± 0.9
**3c**	4.0 ± 0.3	7.1 ± 1.1
**3d**	4.8 ± 0.5	7.5 ± 0.4
**3e**	3.0 ± 0.1	6.0 ± 0.7
**3f**	5.6 ± 1.0	6.4 ± 0.4
**3g**	4.5 ± 0.6	5.9 ± 0.9
**4b**	4.5 ± 0.6	7.0 ± 0.5
**4d**	3.8 ± 0.6	5.9 ± 1.0
**5a**	4.0 ± 0.3	6.9 ± 1.1
**6d**	4.4 ± 0.4	6.4 ± 0.4
**6e**	2.4 ± 0.1	5.5 ± 1.0
**6f**	5.0 ± 0.1	6.8 ± 0.5
**6r**	6.2 ± 0.2	7.3 ± 0.7
**7c**	5.1 ± 0.5	6.6 ± 0.2
**7g**	5.3 ± 0.3	6.4 ± 1.2
**7i**	8.0 ± 0.6	7.5 ± 2.6
**7k**	5.8 ± 0.0	7.8 ± 2.1
**7m**	5.1 ± 0.4	7.4 ± 1.7
**7p**	5.4 ± 1.3	6.9 ± 1.3

**Table 4:** The effect of the tested compounds on β-arrestin recruitment in U2OS cells with stable MOP expression in both agonist and antagonist modes.

Compound	Agonist Mode		Antagonist Mode	
	Emax% (10 µM)	Emax% (0.1 µM)	Emax% (10 µM)	Emax% (0.1 µM)
DAMGO	100%	77%	-	-
ß-FUNALTREXAMINE	-	-	100%	100%
**3a**	2%	7%	4%	6%
**3b**	10%	11%	7%	12%
**3c**	13%	11%	8%	12%
**3d**	11%	11%	15%	8%
**3e**	18%	15%	15%	16%
**3f**	16%	13%	8%	15%
**3g**	14%	14%	16%	10%
**4b**	12%	12%	7%	12%
**4d**	8%	11%	12%	16%
**5a**	11%	13%	15%	15%
**6d**	7%	12%	25%	23%
**6e**	11%	3%	6%	7%
**6f**	10%	8%	17%	4%
**6r**	11%	12%	22%	10%
**7c**	4%	2%	19%	0%
**7g**	15%	12%	12%	10%
**7i**	9%	13%	48%	10%
**7k**	10%	11%	12%	14%
**7m**	11%	9%	19%	5%
**7p**	13%	13%	14%	13%

Results are normalized to the maximal response induced by the reference OP3 receptor agonist, DAMGO (agonist mode), and to the maximal response induced by the reference OP3 receptor antagonist, ß-Funaltrexamine (antagonist mode).

## Data Availability

Data are contained within the article and Supplementary Materials.

## Supplementary Materials

The following supporting information can be downloaded at: https://www.mdpi.com/article/10.3390/molecules29030571/s1, 1. Chemical and spectral data, page 1–17; 2. NMR and MS spectra, page 18–100.

## Abbreviations

OP3—opioid receptor type 3; MOP—mu opioid peptide; GPCRs—G protein-coupled receptors; VGCC—voltage-gated calcium channel; GIRK—G protein-coupled inwardly-rectifying potassium channels); DOP—delta opioid peptide; KOP—kappa opioid peptide; DAMGO—(2*S*)-2-[[2-[[(2*R*)-2-[[(2*S*)-2-Amino-3-(4-hydroxyphenyl)propanoyl]-amino]-propanoyl]amino]acetyl]-methylamino]-*N*-(2-hydroxyethyl)-3-phenylpropanamide (Ala^2^-MePhe^4^-Glyol^5^-enkephalin); cAMP—cyclic adenosine monophosphate; CHO-K1—Chinese hamster ovarian cloned cell line; β-FNA—beta-funaltrexamine; DMSO-d_6_—deuterated dimethyl sulfoxide.
