# Supplementary material for: Novel 1-(1-Arylimiazolin-2-Yl)-3-Arylalkilurea Derivatives with Modulatory Activity on Opioid MOP Receptors
Source: Molecules. 2024 Jan 24;29(3):571. doi: 10.3390/molecules29030571 (PMC10856196; doi:10.3390/molecules29030571)

Novel 1-(1-Arylimiazolin-2-Yl)-3-Arylalkilurea Derivatives with Modulatory Activity on Opioid MOP Receptors

Dominik Straszak [1], Sylwia Woźniak [1], Agata Siwek [2], Monika Głuch-Lutwin [2], Marcin Kołaczkowski [2], Aldona Pietrzak [3], Bartłomiej Drop [4] and Dariusz Matosiuk [1,*]

[1] Department of Synthesis and Chemical Technology of Pharmaceutical Substances, Faculty of Pharmacy, Medical University, Chodzki 4A, 20-093 Lublin, Poland; dominik.straszak@gmail.com (D.S.); sylwia.wozniak@umlub.pl (S.W.)
[2] Faculty of Pharmacy, Jagiellonian University Medical College, Medyczna 9, 30-688 Kraków, Poland; agat.siwek@uj.edu.pl (A.S.); monika.gluch@uj.edu.pl (M.G.-L.); marcin.kolaczkowski@uj.edu.pl (M.K.)
[3] Department of Dermatology, Venereology, and Paediatric Dermatology, Faculty of Medicine, Medical University, Staszica 11, 20-080 Lublin, Poland; aldona.pietrzak@umlub.pl
[4] Department of Medical Informatics and Statistics, Medical University, Jaczewskiego 4, 20-090 Lublin, Poland; bartlomiej.drop@umlub.pl
* Correspondence: dariusz.matosiuk@umlub.pl

Supplementary Materials

Content:

1. **Chemical and spectral data** **page 1-17**
2. **Pictures of NMR and MS spectra** **page 18-100**

Chemical part

1-(1-Phenylimidazolin-2-yl)-3-benzylurea (3a)

Compound 3a was prepared according to the general procedure using 1-phenyl-2-iminoimidazolidine, benzylamine and triphosgene; a white solid with 51% yield was obtained. Formula: $C_{17}H_{18}N_4O$ (m.m. calc. 295.1553). M.p. 199–200°C. HRMS (ESI) *m/z* $[M+H]^+$: 295.1644. ^{1}H NMR (600 MHz, DMSO-d$_6$) δ 8.64 (s, 1H, NH), 7.76 (t, *J* = 2.5 Hz, 1H, NH),7.35–7.23 (m, 8H, Ar-H), 7.03 (tt, *J* = 7.7, 1.3, 2H, Ar-H), 4.21 (d, *J* = 6.2 Hz, 2H, CH$_2$), 3.85 (dd, *J* = 9.5, 7.5 Hz, 2H, CH$_2$), 3.55 (dd, *J* = 9.3, 6.9 Hz, 2H, CH$_2$). ^{13}C NMR (151 MHz, DMSO-d$_6$) δ 159.5, 154.1, 141.7, 139.2, 129.6 (d), 128.4 (d), 127.7, 127.3 (d), 125.0, 121.1 (d), 50.5, 48.0, 42.8.

1-[1-(2-Methylphenyl)imidazolin-2-yl]-3-benzylurea (3b)

Compound 3b was prepared according to the general procedure using 1-(2-methylphenyl)-2-iminoimidazolidine, benzylamine and triphosgene; a white solid with 31% yield was obtained. Formula: $C_{18}H_{20}N_4O$ (m.m. calc. 309.1710). M.p. 119–120°C. HRMS (ESI) *m/z* $[M+H]^+$: 309.1364. ^{1}H NMR (600 MHz, DMSO-d$_6$) δ 8.42 (s, 1H, NH), 7.26–7.20 (m, 7H, Ar-H), 7.19–

7.17 (m, 2H, Ar-H), 6.87 (t, *J* = 7.0 Hz, 1H, NH), 4.20 (d, *J* = 6.5 Hz, 2H, CH_2), 3.65 (dd, *J* = 9.0, 7.4 Hz, 2H, CH_2), 3.61 (dd, *J* = 8.9, 7.2 Hz, 2H, CH_2), 2.23 (s, 3H, CH_3). ^{13}C NMR (151 MHz, DMSO-d$_6$) δ 155.2, 154.2, 140.0, 139.2, 133.6, 131.0, 128.9, 128.5 (d), 128.1, 127.6, 127.2 (d), 124.4, 50.5, 48.8, 42.9, 17.8.

1-[1-(3-Methylphenyl)imidazolin-2-yl]-3-benzylurea (3c)

Compound 3c was prepared according to the general procedure using 1-(3-methylphenyl)-2-iminoimidazolidine, benzylamine and triphosgene; a white solid with 41% yield was obtained. Formula: $C_{18}H_{20}N_4O$ (m.m. calc. 309.1710). M.p. 125–127°C. HRMS (ESI) *m/z* $[M+H]^+$: 309.1370. ^{1}H NMR (600 MHz, DMSO-d$_6$) δ 8.63 (s, 1H, NH), 7.53 (t, *J* = 2.0 Hz, 1H, NH), 7.32–7.25 (m, 6H, Ar-H), 7.24–7.18 (m, 2H, Ar-H), 7.18 (ddd, *J* = 8.1, 2.5, 1.0 Hz, 1H, Ar-H), 4.22 (d, *J* = 6.4 Hz, 2H, CH_2), 3.83 (dd, *J* = 9.6, 7.0 Hz, 2H, CH_2), 3.54 (dd, *J* = 9.4, 7.1 Hz, 2H, CH_2), 2.31 (s, 3H, CH_3). ^{13}C NMR δ (151 MHz, DMSO-d$_6$) δ 159.2, 154.2, 139.8, 139.8, 139.3, 129.6, 128.4 (d), 127.6, 127.2 (d), 123.2, 120.8, 119.9, 50.5, 48.0, 42.8, 21.8.

1-[1-(4-Methylphenyl)imidazolin-2-yl]-3-benzylurea (3d)

Compound 3d was prepared according to the general procedure using 1-(4-methylphenyl)-2-iminoimidazolidine, benzylamine and triphosgene; a white solid with 48% yield was obtained. Formula: $C_{18}H_{20}N_4O$ (m.m. calc. 309.1710). M.p. 146–148°C. HRMS (ESI) *m/z* $[M+H]^+$: 309.1545. ^{1}H NMR (600 MHz, DMSO-d$_6$) δ 8.60 (s, 1H, NH), 7.39–7.32 (m, 5H, Ar-H), 7.28–7.27 (m, 2H, Ar-H), 7.27–7.11 (m, 2H, Ar-H), 6.46 (t, *J* = 7.9 Hz, 1H, NH), 4.22 (d, *J* = 6.6 Hz, 2H, CH_2), 3.84 (dd, *J* = 9.5, 7.0 Hz, 2H, CH_2), 3.57 (dd, *J* = 9.4, 7.0 Hz, 2H, CH_2), 2.25 (s, 3H, CH_3). ^{13}C NMR (151 MHz, DMSO-d$_6$) δ159.6, 154.1, 140.0, 139.2, 135.0, 129.7 (d), 128.5 (d), 127.7, 127.2 (d), 120.7 (d), 50.5, 48.0, 42.9, 20.7.

1-[1-(2-Chlorophenyl)imidazolin-2-yl]-3-benzylurea (3e)

Compound 3e was prepared according to the general procedure using 1-(2-chlorophenyl)-2-iminoimidazolidine, benzylamine and triphosgene; a white solid with 32% yield was obtained. Formula: $C_{17}H_{17}ClN_4O$ (m.m. calc. 329.1164). M.p. 114–116°C. HRMS (ESI) *m/z* $[M+H]^+$: 329.1158. ^{1}H NMR (600 MHz, DMSO-d$_6$) δ 8.49 (s, 1H, NH), 7.54 (ddd, *J* = 8.1, 1.6, 0.5 Hz, 1H, Ar-H), 7.46 (ddd, *J* = 7.7, 1.3, 1.1 Hz, 1H, Ar-H), 7.44–7.31 (m, 7H, Ar-H), 7.26 (t, *J* = 7.8 Hz, 1H, NH), 4.10 (d, *J* = 5.9 Hz, 2H, CH_2), 3.67 (dd, *J* = 9.4, 7.5 Hz, 2H, CH_2), 3.63 (dd, *J* = 9.3, 6.9 Hz, 2H, CH_2). ^{13}C NMR δ (151 MHz, DMSO-d$_6$) δ 155.2, 154.3, 140.6, 139.3, 131.3, 130.1, 128.7 (d), 128.1 (d), 127.8, 127.3 (d), 124.7, 50.5, 48.8, 42.9.

1-[1-(3-Chlorophenyl)imidazolin-2-yl]-3-benzylurea (3f)

Compound 3f was prepared according to the general procedure using 1-(3-chlorophenyl)-2-iminoimidazolidine, benzylamine and triphosgene; a white solid with 44% yield was obtained.

Formula: $C_{17}H_{17}ClN_4O$ (m.m. calc. 329.1164). M.p. 171–173°C. HRMS (ESI) *m/z* $[M+H]^+$: 329.1231. ^{1}H NMR (600 MHz, DMSO-d_6) δ 8.72 (s, 1H, NH), 7.93 (t, *J* = 2.2 Hz, 1H, NH), 7.77 (td, *J* = 1.7, 0.5 Hz, 1H, Ar-H), 7.32–7.20 (m, 7H, Ar-H), 7.07 (dt, *J* = 8.2, 1.6 Hz, 1H, Ar-H), 4.22 (d, *J* = 6.1 Hz, 2H, CH_2), 3.86 (dd, *J* = 9.0, 7.1 Hz, 2H, CH_2), 3.56 (dd, *J* = 9.3, 7.3 Hz, 2H, CH_2). ^{13}C NMR (151 MHz, DMSO-d_6) δ 159.2, 154.0, 140.3, 139.1, 136.5, 128.4 (d), 128.1, 127.7 (d), 127.3 (d), 123.2, 120.0, 50.6, 48.1, 42.9.

1-[1-(4-Chlorophenyl)imidazolin-2-yl]-3-benzylurea (3g)

Compound 3g was prepared according to the general procedure using 1-(4-chlorophenyl)-2-iminoimidazolidine, benzylamine and triphosgene; a white solid with 56% yield was obtained. Formula: $C_{17}H_{17}ClN_4O$ (m.m. calc. 329.1164). M.p. 174–175°C. HRMS (ESI) *m/z* $[M+H]^+$: 329.1158. ^{1}H NMR (600 MHz, DMSO-d_6) δ 8.66 (s, 1H, NH), 7.84 (t, *J* = 2.9 Hz, 1H, NH), 7.35 (ddd, *J* = 8.1, 1.5, 0.5 Hz, 2H, Ar-H), 7.32–7.20 (m, 7H, Ar-H), 4.21 (d, *J* = 6.4 Hz, 2H, CH_2), 3.84 (dd, *J* = 9.6, 7.0, 2H, CH_2), 3.55 (dd, *J* = 9.4, 7.1 Hz, 2H, CH_2). ^{13}C NMR δ (151 MHz, DMSO-d_6) δ 159.4, 154.2, 140.4, 139.3, 130.0, 129.4 (d), 128.4 (d), 127.7, 127.2 (d), 123.3 (d), 50.6, 48.0, 42.8.

1-(1-Phenylimidazolin-2-yl) -3- (1-phenylethyl) urea (4a)

Compound 4a was prepared according to the general procedure using 1-phenyl-2-iminoimidazolidine, 1-phenylethylamine and triphosgene; a white solid with 58% yield was obtained. Formula: $C_{18}H_{20}N_4O$ (m.m. calc. 309.1710). M.p. 183–185°C. HRMS (ESI) *m/z* $[M+H]^+$: 309.1667. ^{1}H NMR (600 MHz, DMSO-d_6) δ 8.55 (s, 1H, NH), 7.63 (d, *J* = 2.0 Hz, 1H, Ar-H), 7.33–7.27 (m, 5H, Ar-H), 7.18-7.10 (m, 4H, Ar-H), 6.88 (d, *J* = 8.5 Hz, 1H, NH), 4.80–4.77 (m, 1H, CH), 3.79 (dd, *J* = 9.4, 7.1 Hz, 2H, CH_2), 3.51 (dd, *J* = 9.3, 7.5 Hz, 2H, CH_2), 1.35 (d, *J* = 6.9 Hz, 3H, CH_3). ^{13}C NMR (151 MHz, DMSO-d_6) δ 159.7, 154.0, 143.2, 141.7, 129.6 (d), 128.3 (d), 127.4, 126.3 (d), 124.9, 121.0 (d), 50.5, 49.9, 48.1, 22.6.

1-[1-(2-Methylphenyl)imidazolin-2-yl]-3-(1-phenylethyl) urea (4b)

Compound 4b was prepared according to the general procedure using 1-(2-methylphenyl)-2-iminoimidazolidine, 1-phenylethylamine and triphosgene; a white solid with 34% yield was obtained. Formula: $C_{19}H_{22}N_4O$ (m.m. calc. 323.1866). M.p. 140–141°C. HRMS (ESI) *m/z* $[M+H]^+$: 323.1820. ^{1}H NMR (600 MHz, DMSO-d_6) δ 8.59 (s, 1H, NH), 7.69–7.27 (m, 7H, Ar-H), 7.22–7.12 (m, 2H, Ar-H), 6.86 (d, *J* = 8.4 Hz, 1H, NH), 4.81–4.78 (m, 1H, CH), 3.82 (dd, *J* = 9.5, 7.3 Hz, 2H, CH_2), 3.51 (dd, *J* = 9.3, 6.9 Hz, 2H, CH_2), 2.33 (s, 3H, CH_3), 1.32 (d, *J* =

7.0 Hz, 3H, CH_3). ^{13}C NMR (151 MHz, DMSO-d_6) δ 155.2, 154.0, 143.5, 140.9, 133.5, 131.0, 129.2 (d), 128.5 (d), 128.1, 127.6 (d), 126.3, 120.7 (d), 50.6, 49.9, 48.8, 22.6, 17.8.

1-[1-(3-Methylphenyl)imidazolin-2-yl]-3-(1-phenylethyl) urea (4c)

Compound 4c was prepared according to the general procedure using 1-(3-methylphenyl)-2-iminoimidazolidine, 1-phenylethylamine and triphosgene; a white solid with 46% yield was obtained. Formula: $C_{19}H_{22}N_4O$ (m.m. calc. 323.1866). M.p. 157–158°C. HRMS (ESI) *m/z* $[M+H]^+$: 323.1850. ^1H NMR (600 MHz, DMSO-d_6) δ 8.57 (s, 1H, NH), 7.70 (ddd, *J* = 8.2, 1.7, 1.3 Hz, 1H, Ar-H), 7.41–7.27 (m, 5H, Ar-H), 7.20–7.18 (m, 2H, Ar-H), 7.13 (ddd, *J* = 8.1, 2.5, 1.0 Hz, 1H, Ar-H), 7.01 (d, *J* = 8.7 Hz, 1H, NH), 4.80–4.77 (m, 1H, CH), 3.82 (dd, *J* = 9.2, 6.9 Hz, 2H, CH_2), 3.52 (dd, *J* = 8.9, 7.3 Hz, 2H, CH_2), 2.33 (s, 3H, CH_3), 1.35 (d, *J* = 7.2 Hz, 3H, CH_3). ^{13}C NMR (151 MHz, DMSO-d_6) δ 159.2, 153.8, 143.5, 139.9, 139.7, 129.5, 128.4 (d), 127.4, 126.3 (d), 123.2, 120.9, 119.8, 50.6, 50.0, 48.0, 22.5, 21.8.

1-[1-(4-Methylphenyl)imidazolin-2-yl]-3-(1-phenylethyl) urea (4d)

Compound 4d was prepared according to the general procedure using 1-(4-methylphenyl)-2-iminoimidazolidine, 1-phenylethylamine and triphosgene; a white solid with 39% yield was obtained. Formula: $C_{19}H_{22}N_4O$ (m.m. calc. 323.1866). M.p. 154–156°C. HRMS (ESI) *m/z* $[M+H]^+$: 323.1545. ^1H NMR (600 MHz, DMSO-d_6) δ 8.55 (s, 1H, NH), 7.62 (d, *J* = 2.4 Hz, 1H, NH), 7.33–7.28 (m, 4H, Ar-H), 7.18–7.13 (m, 5H, Ar-H), 4.80–4.78 (m, 1H, CH), 3.79 (dd, *J* = 9.5, 7.0 Hz, 2H, CH_2), 3.51 (dd, *J* = 9.6, 7.3 Hz, 2H, CH_2), 2.28 (s, 3H, CH_3), 1.34 (d, *J* = 7.0 Hz, 3H). ^{13}C NMR (151 MHz, DMSO-d_6) δ 159.6, 153.9, 143.5, 139.9, 135.1, 129.7 (d), 128.4 (d), 127.4, 126.2 (d), 120.7 (d), 50.5, 49.9, 48.0, 22.5, 20.6.

1-[1-(2-Chlorophenyl)imidazolin-2-yl]-3-(1-phenylethyl) urea (4e)

Compound 4e was prepared according to the general procedure using 1-(2-chlorophenyl)-2-iminoimidazolidine, 1-phenylethylamine and triphosgene; a white solid with 46% yield was obtained. Formula: $C_{18}H_{19}ClN_4O$ (m.m. calc. 343.1320). M.p. 172–174°C. HRMS (ESI) *m/z* $[M+H]^+$: 343.2094. ^1H NMR (600 MHz, DMSO-d_6) δ 8.69 (s, 1H, NH), 7.87 (ddd, *J* = 8.1, 1.6, 0.5 Hz, 1H, Ar-H), 7.80 (d, *J* = 2.6 Hz, 1H, NH), 7.34 (ddd, *J* = 7.7, 1.3, 1.1 Hz, 1H, Ar-H), 7.32–7.07 (m, 7H, Ar-H), 4.82-4.80 (m, 1H, CH), 3.84 (dd, *J* = 9.4, 7.5 Hz, 2H, CH_2), 3.52 (dd, *J* = 9.0, 7.3 Hz, 2H, CH_2), 1.34 (d, *J* = 7.2 Hz, 3H, CH_3). ^{13}C NMR δ (151 MHz, DMSO-d_6) δ 155.0, 153.9, 143.4, 140.6, 131.3, 130.1, 128.4 (d), 128.2, 128.1 (d), 127.4, 126.2 (d), 124.6, 50.5, 49.9, 48.8, 22.6.

1-[1-(3-Chlorophenyl)imidazolin-2-yl]-3-(1-phenylethyl) urea (4f)

Compound 4f was prepared according to the general procedure using 1-(3-chlorophenyl)-2-iminoimidazolidine, 1-phenylethylamine and triphosgene; a white solid with 42% yield was

obtained. Formula: $C_{18}H_{19}ClN_4O$ (m.m. calc. 343.1320). M.p. 174–176°C. HRMS (ESI) *m/z* $[M+H]^+$: 343.1365. ^{1}H NMR (600 MHz, DMSO-d_6) δ 8.68 (s, 1H, NH), 7.87 (d, *J* = 1.9 Hz, 1H, NH), 7.77 (td, *J* = 1.7, 0.5 Hz, 1H, Ar-H), 7.35–7.18 (m, 7H, Ar-H), 7.07 (dt, *J* = 8.2, 1.6 Hz, 1H, Ar-H), 4.82–4.81 (m, 1H, CH), 3.85 (dd, *J* = 9.2, 7.1 Hz, 2H, CH_2), 3.52 (dd, *J* = 9.1, 7.0 Hz, 2H, CH_2), 1.36 (d, *J* = 7.1 Hz, 3H, CH_3). ^{13}C NMR δ (151 MHz, DMSO-d_6) δ 159.2, 154.0, 143.5, 140.5, 136.5, 128.4 (d), 128.1, 127.7, 127.4, 126.2 (d), 123.2, 120.0, 50.6, 50.0, 48.1, 22.5.

1-[1-(4-Chlorophenyl)imidazolin-2-yl]-3-(1-phenylethyl) urea (4g)

Compound 4g was prepared according to the general procedure using 1-(4-chlorophenyl)-2-iminoimidazolidine, 1-phenylethylamine and triphosgene; a white solid with 51% yield was obtained. Formula: $C_{18}H_{19}ClN_4O$ (m.m. calc. 343.1320). M.p. 162–164°C. HRMS (ESI) *m/z* $[M+H]^+$: 343.1291. ^{1}H NMR (600 MHz, DMSO-d_6) δ 8.62 (s, 1H, NH), 7.85 (ddd, *J* = 8.1, 1.5, 0.5 Hz, 2H, Ar-H), 7.36–7.18 (m, 7H, Ar-H), 7.24 (d, *J* = 8.8 Hz, 1H, NH), 4.81–4.78 (m, 1H, CH), 3.82 (dd, *J* = 9.4, 6.9 Hz, 2H, CH_2), 3.52 (dd, *J* = 9.4, 7.2 Hz, 2H, CH_2), 1.35 (d, *J* = 7.0 Hz, 3H, CH_3). ^{13}C NMR δ (151 MHz, DMSO-d_6) δ 159.6, 153.8, 143.5, 140.4, 130.1, 129.4 (d), 128.5 (d), 127.4, 126.3 (d), 123.4 (d), 50.6, 49.9, 48.0, 22.5.

1-(1-Phenylimidazolin-2-yl)-3-(2-phenylethyl)urea (5a)

Compound 5a was prepared according to the general procedure using 1-phenyl-2-iminoimidazolidine, 2-phenylethylamine and triphosgene; a white solid with 49% yield was obtained. Formula $C_{18}H_{20}N_4O$ (m.m. calc. 309.1710). M.p. 121–122°C. HRMS (ESI) *m/z* $[M+H]^+$: 309.1688. ^{1}H NMR (600 MHz, DMSO) δ 8.66 (s, 1H, NH), 7.34–7.25 (m, 6H, Ar-H), 7.21 (tt, *J* = 7.7, 1.3 Hz, 1H, Ar-H), 7.08–7.00 (m, 3H, Ar-H), 6.78 (t, *J* = 5.8 Hz, 1H, NH), 3.82 (dd, *J* = 9.2, 6.9 Hz, 2H, CH_2), 3.62 (dd, *J* = 8.9, 7.1 Hz, 2H, CH_2), 3.27–3.20 (m, 2H, CH_2), 2.76–2.69 (m, 2H, CH_2). ^{13}C NMR δ (151 MHz, DMSO-d_6) δ 159.6, 155.1, 141.7, 139.2, 129.6 (d), 128.9 (2 x d), 128.8, 126.1, 124.9, 121.0 (d), 50.6, 48.0, 41.6, 34.9.

1-[1-(2-Methylphenyl)imidazolin-2-yl]-3-(2-phenylethyl)urea (5b)

Compound 5b was prepared according to the general procedure using 1-(2-methylphenyl)-2-iminoimidazolidine, 2-phenylethylamine and triphosgene; a white solid with 49% yield was obtained. Formula $C_{19}H_{22}N_4O$ (m.m. calc. 323.1866). M.p. 148–150°C. HRMS (ESI) *m/z* $[M+H]^+$: 323.2229. ^{1}H NMR (600 MHz, DMSO) δ 8.69 (s, 1H, NH), 7.39–7.28 (m, 6H, Ar-H), 7.26–7.22 (m, 3H, Ar-H), 6.90 (t, *J* = 5.5 Hz, 1H, NH), 4.11 (dd, *J* = 8.9, 7.1 Hz, 2H, CH_2), 4.01 (dd, *J* = 9.4, 6.9 Hz, 2H, CH_2), 3.91–3.87 (m, 2H, CH_2), 2.82–2.79 (m, 2H, CH_2), 2.25 (s,

3H, CH_3). ^{13}C NMR δ (151 MHz, DMSO-d_6) δ 155.2, 155.1, 140.9, 139.2, 133.5, 131.0, 129.0, 128.9 (2 x d), 128.8, 128.1, 126.5, 124.4, 50.5, 48.8, 41.6, 34.9, 17.8.

1-[1-(3-Methylphenyl)imidazolin-2-yl]-3-(2-phenylethyl)urea (5c)

Compound 5c was prepared according to the general procedure using 1-(3-methylphenyl)-2-iminoimidazolidine, 2-phenylethylamine and triphosgene; a white solid with 45% yield was obtained. Formula $C_{19}H_{22}N_4O$ (m.m. calc. 323.1866). M.p. 115–117°C. HRMS (ESI) *m/z* $[M+H]^+$: 323.1535. ^{1}H NMR (600 MHz, DMSO) δ 8.83 (s, 1H, NH), 7.92 (ddd, *J* = 5.8 Hz, 1H, Ar-H), 7.62 (ddd, *J* = 8.4, 2.2, 0.9 Hz, 1H, Ar-H), 7.34–7.28 (m, 3H, Ar-H), 7.26–7.18 (m, 4H, Ar-H), 6.88 (t, *J* = 2.1 Hz, 1H, NH), 3.89 (dd, *J* = 9.5, 7.0 Hz, 2H, CH_2), 3.64 (dd, *J* = 9.3, 7.2 Hz, 2H, CH_2), 3.26–3.22 (m, 2H, CH_2), 2.75–2.71 (m, 2H, CH_2), 2.29 (s, 3H, CH_3). ^{13}C NMR δ (151 MHz, DMSO-d_6) δ 159.2, 155.2, 139.9, 139.7, 139.2, 129.5, 129.0, 128.8 (2 x d), 126.6, 123.2, 120.9, 119.8, 50.6, 48.0, 41.6, 34.9, 21.8.

1-[1-(4-Methylphenyl)imidazolin-2-yl]-3-(2-phenylethyl)urea (5d)

Compound 5d was prepared according to the general procedure using 1-(4-methylphenyl)-2-iminoimidazolidine, 2-phenylethylamine and triphosgene; a white solid with 51% yield was obtained. Formula $C_{19}H_{22}N_4O$ (m.m. calc. 323.1866). M.p. 97–99°C. HRMS (ESI) *m/z* $[M+H]^+$ oznaczono: 323.1534. ^{1}H NMR (600 MHz, DMSO) δ 8.67 (s, 1H, NH), 7.85–7.84 (m, 3H, Ar-H), 7.30–7.28 (m, 3H, Ar-H), 7.21–7.19 (m, 3H, Ar-H), 6.68 (t, *J* = 5.9 Hz, 1H, NH), 4.45 (dd, *J* = 9.2, 7.3 Hz, 2H, CH_2), 3.53 (dd, *J* = 9.4, 6.9 Hz, 2H CH_2), 3.22–3.20 (m, 2H, CH_2), 2.67–2.65 (m, 2H, CH_2), 2.29 (s, 3H, CH_3). ^{13}C NMR δ (151 MHz, DMSO-d_6) δ 159.6, 155.1, 140.0, 139.2, 135.1, 129.7 (d), 128.9 (2 x d), 128.8, 126.5, 120.7 (d), 50.6, 48.0, 41.5, 34.9, 20.7.

1-[1-(2-Chlorophenyl)imidazolin-2-yl]-3-(2-phenylethyl)urea (5e)

Compound 5e was prepared according to the general procedure using 1-(2-chlorophenyl)-2-iminoimidazolidine, 2-phenylethylamine and triphosgene; a white solid with 32% yield was obtained. Formula $C_{18}H_{19}ClN_4O$ (m.m. calc. 343.1320). M.p. 113–115°C. HRMS (ESI) *m/z* $[M+H]^+$: 343.1271. ^{1}H NMR (600 MHz, DMSO) δ 8.70 (s, 1H, NH), 7.58–7.53 (m, 2H, Ar-H), 7.29–7.21 (m, 5H, Ar-H), 7.21–7.20 (m, 2H, Ar-H), 7.09 (t, *J* = 6.2 Hz, 1H, NH), 3.82 (dd, *J* = 9.2, 7.2 Hz, 2H, CH_2), 3.55 (dd, *J* = 8.9, 7.1 Hz, 2H, CH_2), 3.29–3.20 (m, 2H, CH_2), 2.68–2.65 (m, 2H, CH_2). ^{13}C NMR δ (151 MHz, DMSO-d_6) δ 155.1, 155.0, 140.6, 139.3, 131.3, 130.1, 128.9, 128.8 (d), 128.2, 128.1, 126.6, 124.6, 50.5, 48.8, 41.5, 34.9.

1-[1-(3-Chlorophenyl)imidazolin-2-yl]-3-(2-phenylethyl)urea (5f)

Compound 5f was prepared according to the general procedure using 1-(3-chlorophenyl)-2-iminoimidazolidine, 2-phenylethylamine and triphosgene; a white solid with 41% yield was obtained. Formula $C_{18}H_{19}ClN_4O$ (m.m. calc. 343.1320). M.p. 105–107°C. HRMS (ESI) *m/z*

$[M+H]^+$: 343.1297. ^{1}H NMR (600 MHz, DMSO) δ 8.73 (s, 1H, NH), 7.91 (td, *J* = 1.7, 0.5 Hz, 2H, Ar-H), 7.72 (dt, *J* = 8.4, 2.2, 0.9 Hz, 1H, Ar-H), 7.35–7.17 (m, 5H, Ar-H), 7.11–7.08 (m, 1H, Ar-H), 6.88 (t, *J* = 5.5 Hz, 1H, NH), 3.87 (dd, *J* = 9.3, 7.0 Hz, 2H, CH_2), 3.56 (dd, *J* = 9.0, 7.2 Hz, 2H, CH_2), 3.25–3.21 (m, 2H, CH_2), 2.74–2.69 (m, 2H, CH_2). ^{13}C NMR δ (151 MHz, DMSO-d_6) δ 159.2, 155.1, 140.3, 139.3, 136.5, 128.9 (d), 128.1, 127.7, 126.5, 123.2, 120.0, 50.6, 48.0, 41.6, 35.0.

1-[1-(4-Chlorophenyl)imidazolin-2-yl]-3-(2-phenylethyl)urea (5g)

Compound 5g was prepared according to the general procedure using 1-(4-chlorophenyl)-2-iminoimidazolidine, 2-phenylethylamine and triphosgene; a white solid with 48% yield was obtained. Formula $C_{18}H_{19}ClN_4O$ (m.m. calc. 343.1320). M.p. 122–123°C. HRMS (ESI) *m/z* $[M+H]^+$: 343.1316. ^{1}H NMR (600 MHz, DMSO) δ 8.67 (s, 1H, NH), 7.85 (ddd, *J* = 7.8, 1.5, 0.5, Hz, 2H, Ar-H), 7.37 (ddd, *J* = 8.1, 1.7, 0.5 Hz, 2H, Ar-H), 7.23–7.16 (m, 5H, Ar-H), 6.84 (t, *J* = 6.1 Hz, 1H, NH), 3.86 (dd, *J* = 9.1, 7.0 Hz, 2H, CH_2), 3.57 (dd, *J* = 9.2, 7.1 Hz, 2H, CH_2), 3.24–3.18 (m, 2H, CH_2), 2.76–2.68 (m, 2H, CH_2). ^{13}C NMR δ (151 MHz, DMSO-d_6) δ 159.6, 155.1, 140.4, 139.2, 130.1, 129.4 (d), 128.9 (2 x d), 128.8, 126.6, 123.4 (d), 50.6, 48.0, 41.6, 34.9.

1-[1-(4-Methylphenyl)imidazolin-2-yl]-3-(1-phenylprop-2-yl)urea (6a)

Compound 6a was prepared according to the general procedure using 1-(4-methylphenyl)-2-iminoimidazolidine, 1-phenylpropyl-2-amine and triphosgene; a white solid with 41% yield was obtained. Formula $C_{20}H_{24}N_4O$ (m.m. calc. 337.2023). M.p. 152–153°C. HRMS (ESI) *m/z* $[M+H]^+$: 337.1959. ^{1}H NMR (600 MHz, DMSO-d_6) δ 8.42 (s, 1H, NH), 7.83–7.79 (m, 1H, Ar-H), 7.46–7.28 (m, 5H, Ar-H), 7.28–7.16 (m, 3H, Ar-H), 6.70 (d, *J* = 8.2 Hz, 1H, NH), 4.32 (dd, *J* = 9.0, 7.2 Hz, 2H, CH_2), 4.03–3.98 (m, 1H, CH), 3.77 (dd, *J* = 9.1, 7.0 Hz 2H, CH_2), 2.75 (dd, *J* = 12.8, 5.0 Hz, 1H, CH_2), 2.62 (dd, *J* = 12.8, 8.0 Hz, 1H, CH_2), 2.32 (s, 3H, CH_3), 1.12 (d, *J* = 6.2 Hz, 3H, CH_3). ^{13}C NMR δ (151 MHz, DMSO-d_6) δ 159.2, 154.4, 140.0, 138.6, 135.1, 129.7 (d), 129.1 (d), 128.8 (d), 127.2, 120.7 (d), 50.5, 48.1, 47.5, 42.0, 20.8, 20.7.

1-[1-(4-Chlorophenyl)imidazolin-2-yl]-3-(1-phenylprop-2-yl)urea (6b)

Compound 6b was prepared according to the general procedure using 1-(4-chlorophenyl)-2-iminoimidazolidine, 1-phenylpropyl-2-amine and triphosgene; a white solid with 38% yield was obtained. Formula $C_{19}H_{21}ClN_4O$ (m.m. calc. 357.1477). M.p. 141–142°C. HRMS (ESI) *m/z* $[M+H]^+$: 357.1048. ^{1}H NMR (600 MHz, DMSO-d_6) δ 8.56 (s, 1H, NH), 7.56–7.51 (m, 1H, Ar-H), 7.44–7.41 (m, 1H, Ar-H), 7.40–7.37 (m, 1H, Ar-H), 7.36–7.31 (m, 1H, Ar-H), 7.25–

7.20 (m, 2H, Ar-H), 7.19–7.08 (m, 3H, Ar-H), 6.92 (d, *J* = 8.0 Hz, 1H, NH), 4.05 (dd, *J* = 8.9, 6.9 Hz, 2H, CH_2), 3.88–3.84 (m, 1H, CH), 3.60 (dd, *J* = 9.0, 6.9 Hz, 2H, CH_2), 2.81 (dd, *J* = 13.0, 5.4 Hz, 1H, CH_2), 2.68 (dd, *J* = 12.5, 8.0 Hz, 1H, CH_2), 1.11 (d, *J* = 6.5 Hz, 3H, CH_3). ^{13}C NMR δ (151 MHz, DMSO-d_6) δ 159.2, 154.4, 140.4, 138.6, 130.1, 129.4 (d), 129.2 (d), 128.8 (d), 127.2, 123.4 (d), 50.6, 48.0, 47.6, 41.9, 20.8.

1-[1-(4-Methylphenyl)imidazolin-2-yl]-3-[1-(4-chlorophenyl)prop-2-yl]urea (6c)

Compound 6c was prepared according to the general procedure using 1-(4-methylphenyl)-2-iminoimidazolidine, 1-(4-chlorophenyl)propyl-2-amine and triphosgene; a white solid with 46% yield was obtained. Formula $C_{20}H_{23}ClN_4O$ (m.m. calc. 371.1633). M.p. 121–122°C. HRMS (ESI) *m/z* $[M+H]^+$: 371.1621. ^{1}H NMR (600 MHz, DMSO-d_6) δ 8.37 (s, 1H, NH), 7.66–7.58 (m, 1H, Ar-H), 7.49–7.40 (m, 2H, Ar-H), 7.38–7.30 (m, 2H, Ar-H), 7.28–7.19 (m, 3H, Ar-H), 7.05 (d, *J* = 8.0 Hz, 1H, NH), 4.29 (dd, *J* = 9.1, 7.4 Hz, 2H, CH_2),4.03–3.99 (m, 1H, CH), 3.70 (dd, *J* = 9.2, 7.1 Hz, 2H, CH_2), 2.67–2.54 (m, 2H, CH_2), 2.33 (s, CH_3), 1.08 (d, *J* = 6.3 Hz, 3H, CH_3). ^{13}C NMR δ (151 MHz, DMSO-d_6) δ 159.3, 154.5, 140.0, 136.9, 135.0, 132.1, 130.7 (d), 129.8 (d), 128.6 (d), 120.6 (d), 50.5, 48.0, 47.5, 42.1, 21.0, 20.7.

1-[1-(4-Chlorophenyl)imidazolin-2-yl]-3-[1-(4-chlorophenyl)prop-2-yl]urea (6d)

Compound 6d was prepared according to the general procedure using 1-(4-chlorophenyl)-2-iminoimidazolidine, 1-(4-chlorophenyl)propyl-2-amine and triphosgene; a white solid with 46% yield was obtained. Formula $C_{19}H_{20}Cl_2N_4O$ (m.m. calc. 391.1087). M.p. 133–134°C. HRMS (ESI) *m/z* $[M+H]^+$: 391.1052. ^{1}H NMR (600 MHz, DMSO-d_6) δ 8.46 (s, 1H, NH), 7.57–7.46 (m, 2H, Ar-H), 7.29–7.21 (m, 2H, Ar-H), 7.20–7.10 (m, 4H, Ar-H), 7.00 (d, *J* = 8.4 Hz, 1H, NH), 4.18 (dd, *J* = 9.2, 7.3 Hz, 2H, CH_2), 3.98–3.91 (m, 1H, CH), 3.64 (dd, *J* = 9.0, 6.9 Hz, 2H, CH_2), 2.65–2.57 (m, 2H, CH_2), 1.13 (d, *J* = 6.0 Hz, 3H, CH_3). ^{13}C NMR δ (151 MHz, DMSO-d_6) δ 159.2, 154.4, 140.5, 136.9, 132.1, 130.6 (d), 130.1, 129.4 (d), 128.6 (d), 123.4 (d), 50.6, 48.0, 47.4, 42.1, 20.8.

1-[1-(4-Methylphenyl)imidazolin-2-yl]-3-[1-(4-fluorophenyl)prop-2-yl]urea (6e)

Compound 6e was prepared according to the general procedure using 1-(4-methylphenyl)-2-iminoimidazolidine, 1-(4-fluorophenyl)propyl-2-amine and triphosgene; a white solid with 54% yield was obtained. Formula $C_{20}H_{23}FN_4O$ (m.m. calc. 355.1929). M.p. 149–150°C. HRMS (ESI) *m/z* $[M+H]^+$: 355.1891. ^{1}H NMR (600 MHz, DMSO-d_6) δ 8.47 (s, 1H, NH), 7.54–7.48 (m, 2H, Ar-H), 7.29–7.19 (m, 3H, Ar-H), 7.18–7.11 (m, 3H, Ar-H), 6.98 (d, *J* = 8.6 Hz, 1H, NH), 4.13 (dd, *J* = 9.2, 6.9 Hz, 2H, CH_2), 3.88–3.82 (m, 1H, CH), 3.68 (dd, *J* = 9.0, 7.2 Hz, 2H, CH_2), 2.64–2.53 (m, 2H, CH_2), 2.25 (s, 3H, CH_3), 1.12 (d, *J* = 5.9 Hz, 3H, CH_3). ^{13}C NMR δ

(151 MHz, DMSO-d6) δ 161.0, 159.2, 154.4, 140.0, 134.9, 130.9 (d), 129.7 (d), 120.6 (d), 115.6, 114.4, 50.5, 48.0, 47.5, 41.8, 21.0, 20.7.

1-[1-(4-Chlorophenyl)imidazolin-2-yl]-3-[1-(4-fluorophenyl)prop-2-yl]urea (6f)

Compound 6f was prepared according to the general procedure using 1-(4-chlorophenyl)-2-iminoimidazolidine, 1-(4-fluorophenyl)propyl-2-amine and triphosgene; a white solid with 44% yield was obtained. Formula $C_{19}H_{20}ClFN_4O$ (m.m. calc. 375.1382). M.p. 125–126°C. HRMS (ESI) *m/z* $[M+H]^+$: 375.1360. ^1H NMR (600 MHz, DMSO-d6) δ 8.66 (s, 1H, NH), 7.92–7.87 (m, 2H, Ar-H), 7.43–7.32 (m, 4H, Ar-H), 7.20–7.15 (m, 2H, Ar-H), 7.01 (d, *J* = 7.8 Hz, 1H, NH), 4.09 (dd, *J* = 9.1, 7.4 Hz, 2H, CH_2), 3.80–3.77 (m, 1H, CH), 3.59 (dd, *J* = 9.2, 7.3 Hz, 2H, CH_2), 2.68–2.61 (m, 2H, CH_2), 1.14 (d, *J* = 6.1 Hz, 3H, CH_3). ^{13}C NMR δ (151 MHz, DMSO-d6) δ 163.0, 159.3, 154.5, 140.3, 134.9, 130.9 (d), 130.1, 129.5 (d), 123.3 (d), 115.4, 50.6, 48.1, 47.4, 41.6, 21.0.

1-[1-(4-Methylphenyl)imidazolin-2-yl]-3-[1-(4-methylphenyl)prop-2-yl]urea (6g)

Compound 6g was prepared according to the general procedure using 1-(4-methylphenyl)-2-iminoimidazolidine, 1-(4-methylphenyl)propyl-2-amine and triphosgene; a white solid with 39% yield was obtained. Formula $C_{21}H_{26}N_4O$ (m.m. calc. 351.2179). M.p. 133–134°C. HRMS (ESI) *m/z* $[M+H]^+$: 351.2234. ^1H NMR (600 MHz, DMSO-d6) δ 8.59 (s, 1H, NH), 7.77 (d, *J* = 8.1 Hz, 1H, NH), 7.43–7.32 (m, 6H, Ar-H), 7.27–7.21 (m, 2H, Ar-H), 4.29 (dd, *J* = 9.3, 7.1 Hz, 2H, CH_2), 4.04–4.01 (m, 1H, CH), 3.65 (dd, *J* = 8.9, 7.1 Hz, 2H, CH_2), 2.64–2.52 (m, 2H, CH_2), 2.31 (s, 3H, CH_3), 2.22 (s, 3H, CH_3), 1.03 (d, *J* = 6.2 Hz, 3H, CH_3). ^{13}C NMR δ (151 MHz, DMSO-d6) δ 159.2, 154.3, 141.1, 136.3, 136.1, 135.1, 129.7 (d), 129.3, 129.2 (2 x d), 120.7 (d), 50.5, 48.0, 47.6, 41.9, 21.0, 20.9, 20.7.

1-[1-(4-Chlorophenyl)imidazolin-2-yl]-3-[1-(4-methylphenyl)prop-2-yl]urea (6h)

Compound 6h was prepared according to the general procedure using 1-(4-chlorophenyl)-2-iminoimidazolidine, 1-(4-methylphenyl)propyl-2-amine and triphosgene; a white solid with 36% yield was obtained. Formula $C_{20}H_{23}ClN_4O$ (m.m. calc. 371.1633). M.p. 139–140°C. HRMS (ESI) *m/z* $[M+H]^+$: 371.2033. ^1H NMR (600 MHz, DMSO-d6) δ 8.48 (s, 1H, NH), 7.52–7.49 (m, 1H, Ar-H), 7.45–7.41 (m, 1H, Ar-H), 7.39–7.34 (m, 1H, Ar-H), 7.34–7.30 (m, 1H, Ar-H), 7.28–7.20 (m, 2H, Ar-H), 7.19–7.11 (m, 2H, Ar-H), 6.98 (d, *J* = 8.2 Hz, 1H, NH), 4.07 (d, *J* = 8.9, 7.0 Hz, 2H, CH_2), 3.72–3.69 (m, 1H, CH), 3.59 (dd, *J* = 8.8, 7.0 Hz, 2H, CH_2), 2.61–2.53 (m, 2H, CH_2), 2.30 (s, 3H, CH_3), 1.12 (d, *J* = 6.4 Hz, 3H, CH_3). ^{13}C NMR δ (151 MHz, DMSO-d6) δ 159.3, 154.5, 140.4, 136.2, 136.2, 130.1, 129.4 (d), 129.2 (2 x d), 123.4 (d), 50.5, 48.0, 47.5, 42.0, 21.0, 20.8.

1-[1-(4-Methylphenyl)imidazolin-2-yl]-3-[1-(4-methoxyphenyl)prop-2-yl]urea (6i)

Compound 6i was prepared according to the general procedure using 1-(4-methylphenyl)-2-iminoimidazolidine, 1-(4-methoxyphenyl)propyl-2-amine and triphosgene; a white solid with 32% yield was obtained. Formula $C_{21}H_{26}N_4O_2$ (m.m. calc. 367.2128). M.p. 147–148°C. HRMS (ESI) *m/z* $[M+H]^+$: 367.2132. ^{1}H NMR (600 MHz, DMSO-d$_6$) δ 8.62 (s, 1H, NH), 7.59 (d, *J* = 8.1 Hz, 1H, NH), 7.53–7.47 (m, 3H, Ar-H), 7.44–7.38 (m, 3H, Ar-H), 7.32–7.27 (m, 2H, Ar-H), 4.19 (dd, *J* = 9.0, 7.0 Hz, 2H, CH_2), 3.99–3.88 (m, 1H, CH), 3.67 (s, 3H, OCH_3), 3.63 (dd, *J* = 9.1, 7.0 Hz, 2H, CH_2), 2.87–2.75 (m, 2H, CH_2), 2.30 (s, 3H, CH_3) 1.07 (d, *J* = 6.3 Hz, 3H, CH_3). ^{13}C NMR δ (151 MHz, DMSO-d$_6$) δ 159.2, 158.5, 154.4, 140.0, 135.3, 132.4, 130.2 (d), 129.6 (d), 120.8 (d), 113.8 (d), 55.4, 50.7, 48.0, 47.8, 42.1, 21.1, 20.8.

1-[1-(4-Methylphenyl)imidazolin-2-yl]-3-[1-(3,4-dichlorophenyl)prop-2-yl]urea (6j)

Compound 6j was prepared according to the general procedure using 1-(4-methylphenyl)-2-iminoimidazolidine, 1-(3,4-dichlorophenyl)propyl-2-amine and triphosgene; a white solid with 44% yield was obtained. Formula $C_{20}H_{22}Cl_2N_4O$ (m.m. calc. 405.1243). M.p. 117–118°C. HRMS (ESI) *m/z* $[M+H]^+$: 405.1271. ^{1}H NMR (600 MHz, DMSO-d$_6$) δ 8.72 (s, 1H, NH), 7.38–7.20 (m, 4H, Ar-H), 7.19–7.08 (m, 3H, Ar-H), 6.77 (d, *J* = 7.8 Hz, 1H, NH), 4.19 (dd, *J* = 9.0, 7.0 Hz, 2H, CH_2), 3.90–3.85 (m, 1H, CH), 3.53 (dd, *J* = 9.1, 6.9 Hz, 2H, CH_2), 2.65–2.53 (m, 2H, CH_2), 2.30 (m, 3H, CH_3), 1.14 (d, *J* = 6.0 Hz, 3H, CH_3). ^{13}C NMR δ (151 MHz, DMSO-d$_6$) δ 159.3, 154.5, 140.0, 138.2, 135.0, 133.6, 131.9, 130.4 (d), 129.7, 127.7 (d), 120.6 (d), 50.6, 48.0, 47.5, 42.0, 21.0, 20.6.

1-[1-(4-Chlorophenyl)imidazolin-2-yl]-3-[1-(3,4-dichlorophenyl)prop-2-yl]urea (6k)

Compound 6k was prepared according to the general procedure using 1-(4-chlorophenyl)-2-iminoimidazolidine, 1-(3,4-dichlorophenyl)propyl-2-amine and triphosgene; a white solid with 45% yield was obtained. Formula $C_{19}H_{19}Cl_3N_4O$ (m.m. calc. 425.0670). M.p. 109–110°C. HRMS (ESI) *m/z* $[M+H]^+$: 425.0679. ^{1}H NMR (600 MHz, DMSO-d$_6$) δ 8.68 (s, 1H, NH), 7.84–7.76 (m, 4H, Ar-H), 7.76–7.68 (m, 3H, Ar-H), 7.18 (d, *J* = 8.8 Hz, 1H, NH), 4.25 (dd, *J* = 9.1, 6.9 Hz, 2H, CH_2), 3.67–3.51 (m, 1H, CH), 3.51 (dd, *J* = 9.5, 7.3 Hz, 2H, CH_2), 2.67–2.51 (m, 2H, CH_2), 1.12 (d, J = 6.0 Hz, 3H, CH_3). ^{13}C NMR δ (151 MHz, DMSO-d$_6$) δ 159.1, 154.3, 140.4, 138.2, 133.6, 131.8, 130.5 (d), 130.1, 129.5 (d), 127.8, 123.4 (d), 50.6, 48.1, 47.5, 42.1, 21.1.

1-[1-(4-Methylphenyl)imidazolin-2-yl]-3-[1-(furan-2-yl)prop-2-yl]urea (6l)

Compound 6l was prepared according to the general procedure using 1-(4-methylphenyl)-2-iminoimidazolidine, 1-(furan-2-yl)propyl-2-amine and triphosgene; a white solid with 37% yield was obtained. Formula $C_{18}H_{22}N_4O_2$ (m.m. calc. 327.1816). M.p. 170–171°C. HRMS (ESI) *m/z* $[M+H]^+$: 327.2054. ^{1}H NMR (600 MHz, DMSO-d$_6$) δ 8.18 (s, 1H, NH), 7.41–7.19

(m, 5H, Ar-H), 6.70–6.65 (m, 2H, Ar-H), 6.42 (d, *J* = 8.7 Hz, 1H, NH), 4.39 (dd, *J* = 9.0, 7.5 Hz, 2H, CH_2), 4.23–4.19 (m, 1H, CH), 4.04 (dd, *J* = 8.8, 7.0 Hz, 2H, CH_2), 2.71–2.61 (m, 2H, CH_2), 2.30 (s, 3H, CH_3), 1.14 (d, *J* = 5.8 Hz, 3H, CH_3). ^{13}C NMR δ 159.2, 154.3, 152.9, 141.9, 140.0, 135.0, 129.8 (d), 120.7 (d), 110.8, 108.4, 50.6, 48.1, 45.9, 34.2, 20.9, 20.7.

1-[1-(4-Chlorophenyl)imidazolin-2-yl]-3-[1-(furan-2-yl)prop-2-yl]urea (6m)

Compound 6m was prepared according to the general procedure using 1-(4-chlorophenyl)-2-iminoimidazolidine, 1-(furan-2-yl)propyl-2-amine and triphosgene; a white solid with 42% yield was obtained. Formula $C_{17}H_{19}ClN_4O_2$ (m.m. calc. 347.1269). M.p. 162–163°C. HRMS (ESI) *m/z* $[M+H]^+$: 347.1232. ^{1}H NMR (600 MHz, DMSO-d_6) δ 8.20 (s, 1H, NH), 7.29–7.07 (m, 5H, Ar-H), 6.71–6.62 (m, 2H, Ar-H), 6.42 (d, *J* = 8.1 Hz, 1H, NH), 4.29 (dd, *J* = 9.8, 7.3 Hz, 2H, CH_2), 4.11–4.09 (m, 1H, CH), 3.93 (dd, *J* = 9.7, 7.5 Hz, 2H, CH_2), 3.31–3.22 (m, 2H, CH_2), 1.13 (d, *J* = 5.1 Hz, 3H, CH_3). ^{13}C NMR δ 159.2, 154.4, 152.8, 141.9, 140.4, 130.1, 129.4 (d), 123.4 (d), 110.8, 108.4, 50.6, 48.0, 46.0, 34.3, 20.8.

1-[1-(4-Methylphenyl)imidazolin-2-yl]-3-[1-(thiophen-2-yl)prop-2-yl]urea (6n)

Compound 6n was prepared according to the general procedure using 1-(4-methylphenyl)-2-iminoimidazolidine, 1-(thiophen-2-yl)propyl-2-amine and triphosgene; a white solid with 33% yield was obtained. Formula $C_{18}H_{22}N_4OS$ (m.m. calc. 343.1587). M.p. 144–145°C. HRMS (ESI) *m/z* $[M+H]^+$: 343.1728. ^{1}H NMR (600 MHz, DMSO-d_6) δ 8.22 (s, 1H, NH), 7.48–7.31 (m, 4H, Ar-H), 7.08–7.04 (m, 2H, Ar-H), 6.81–6.77 (m, 1H, Ar-H), 6.62 (d, *J* = 7.9 Hz, 1H, NH), 4.14 (dd, *J* = 9.2, 7.4 Hz, 2H, CH_2), 4.02–3.97 (m, 1H, CH), 3.81 (dd, *J* = 9.0, 7.1 Hz, 2H, CH_2), 3.19–3.07 (m, 2H, CH_2), 2.31 (s, 3H, CH_3), 1.11 (d, *J* = 5.8 Hz, 3H, CH_3). ^{13}C NMR δ 159.3, 154.5, 140.2, 140.0, 135.1, 129.8 (d), 127.4, 126.6, 125.3, 120.7 (d), 50.9, 48.1, 46.4, 37.0, 21.0, 20.7.

1-[1-(4-Chlorophenyl)imidazolin-2-yl]-3-[1-(thiophen-2-yl)prop-2-yl]urea (6o)

Compound 6o was prepared according to the general procedure using 1-(4-chlorophenyl)-2-iminoimidazolidine, 1-(thiophen-2-yl)propyl-2-amine and triphosgene; a white solid with 39% yield was obtained. Formula $C_{17}H_{19}ClN_4OS$ (m.m. calc. 363.1041). M.p. 151–152°C. HRMS (ESI) *m/z* $[M+H]^+$: 363.1733. ^{1}H NMR (600 MHz, DMSO-d_6) δ 8.29 (s, 1H, NH), 7.46–7.28 (m, 4H, Ar-H), 7.10–7.06 (m, 2H, Ar-H), 6.78 (t, J = 7.0 Hz, 1H, Ar-H), 6.59 (d, *J* = 8.2 Hz, 1H, NH), 4.19 (dd, *J* = 9.0, 7.3 Hz, 2H, CH_2), 4.03–3.99 (m, 2H, CH), 3.79 (dd, *J* = 9.1, 7.6 Hz, 2H, CH_2), 3.18–3.10 (m, 2H, CH_2), 1.21 (d, *J* = 5.3 Hz, 3H, CH_3). ^{13}C NMR δ 159.2, 154.4, 140.4, 140.3, 130.0, 129.5 (d), 127.3, 126.6, 125.2, 123.5 (d), 50.7, 48.1, 46.6, 37.1, 21.1.

1-[1-(4-Methylphenyl)imidazolin-2-yl]-3-[1-(5-methylthiophen-2-yl)prop-2-yl]urea (6p)

Compound 6p was prepared according to the general procedure using 1-(4-methylphenyl)-2-iminoimidazolidine, 1-(5-methylthiophen-2-yl)propyl-2-amine and triphosgene; a white solid with 29% yield was obtained. Formula $C_{19}H_{24}N_4OS$ (m.m. calc. 357.1744). M.p. 146–147°C. HRMS (ESI) *m/z* $[M+H]^+$: 357.2069. ^1H NMR (600 MHz, DMSO-d_6) δ 8.20 (s, 1H, NH), 7.21–7.12 (m, 4H, Ar-H), 6.81 (d, *J* = 7.6 Hz, 1H, NH), 6.74–6.67 (m, 2H, Ar-H), 4.11 (dd, *J* = 9.1, 6.9 Hz, 2H, CH_2), 3.96–3.92 (m, 1H, CH), 3.58 (dd, *J* = 9.0, 7.5 Hz, 2H, CH_2), 3.12–3.08 (m, 2H, CH_2), 2.32 (s, 3H, CH_3), 2.25 (s, 3H, CH_3), 1.11 (d, *J* = 5.4 Hz, 3H, CH_3). ^{13}C NMR δ 159.2, 154.4, 140.0, 139.4, 137.1, 135.1, 129.8 (d), 126.6, 125.6, 120.7 (d), 50.7, 48.1, 46.4, 38.3, 21.0, 20.7, 15.2.

1-[1-(4-Chlorophenyl)imidazolin-2-yl]-3-[1-(5-methylthiophen-2-yl)prop-2-yl]urea (6r)

Compound 6r was prepared according to the general procedure using 1-(4-chlorophenyl)-2-iminoimidazolidine, 1-(5-methylthiophen-2-yl)propyl-2-amine and triphosgene; a white solid with 31% yield was obtained. Formula $C_{18}H_{21}ClN_4OS$ (m.m. calc. 377.1197). M.p. 139–140°C. HRMS (ESI) *m/z* $[M+H]^+$: 377.1178. ^1H NMR (600 MHz, DMSO-d_6) δ 8.24 (s, 1H, NH), 7.38–7.17 (m, 4H, Ar-H), 6.81 (d, *J* = 7.1 Hz, 1H, NH), 6.63–6.59 (m, 2H, Ar-H), 4.19 (dd, *J* = 9.2, 7.4 Hz, 2H, CH_2), 3.91–3.88 (m, 1H, CH), 3.61 (dd, *J* = 9.2, 7.5 Hz, 2H, CH_2), 3.21–3.12 (m, 2H, CH_2), 2.24 (s, 3H, CH_3), 1.21 (d, *J* = 5.1 Hz, 3H, CH_3). ^{13}C NMR δ 159.3, 154.4, 140.5, 139.4, 137.1, 130.1, 129.5 (d), 126.5, 125.6, 123.3 (d), 50.6, 48.1, 46.4, 38.4, 21.0, 15.2.

1-[1-(4-Methoxyphenyl)imidazolin-2-yl]-3-(4-phenylbut-2-yl)urea (7a)

Compound 7a was prepared according to the general procedure using 1-(4-methoxyphenyl)-2-iminoimidazolidine, 4-phenylbutyl-2-amine and triphosgene; a white solid with 34% yield was obtained. Formula $C_{21}H_{26}N_4O_2$ (m.m. calc. 367.2128). M.p. 156–157°C. HRMS (ESI) *m/z* $[M+H]^+$: 367.2181. ^1H NMR (600 MHz, DMSO-d_6) δ 8.64 (s, 1H, NH), 7.87–7.84 (m, 2H, Ar-H), 7.38–7.34 (m, 2H, Ar-H), 7.29–7.27 (m, 1H, Ar-H), 7.21–7.10 (m, 4H, Ar-H), 6.63 (d, *J* = 8.1 Hz, 1H, NH), 3.87 (dd, *J* = 9.0, 7.2 Hz, 2H, CH_2), 3.68–3.64 (m, 1H, CH), 3.75 (s, 3H, OCH_3), 3.55 (dd, *J* = 9.4, 7.2 Hz, 2H, CH_2) 2.60–2.53 (m, 2H. CH_2), 1.74–1.63 (m, 1H, CH_2), 1.62–1.52 (m, 1H, CH_2), 1.07 (d, *J* = 6.5 Hz, 3H, CH_3). ^{13}C NMR δ (151 MHz, DMSO-d_6) δ 159.2, 155.8, 154.6, 141.6, 137.0, 128.6 (d), 128.5 (d), 126.5, 122.3 (d), 115.3 (d), 55.4, 50.6, 48.1, 45.6, 37.1, 32.5, 21.0.

1-[1-(4-Chlorophenyl)imidazolin-2-yl]-3-(4-phenylbut-2-yl)urea (7b)

Compound 7b was prepared according to the general procedure using 1-(4-chlorophenyl)-2-iminoimidazolidine, 4-phenylbutyl-2-amine and triphosgene; a white solid with 42% yield was obtained. Formula $C_{20}H_{23}ClN_4O$ (m.m. calc. 371.1633). M.p. 167–168°C. HRMS (ESI) *m/z* $[M+H]^+$: 371.1720. ^1H NMR (600 MHz, DMSO-d_6) δ 8.68 (s, 1H, NH), 7.62–7.51 (m, 2H, Ar-

H), 7.56–7.50 (m, 2H, Ar-H), 7.44 (d, *J* = 8.6 Hz, 1H, NH), 7.38–7.11 (m, 5H, Ar-H), 3.86 (dd, *J* = 9.4, 7.5 Hz, 2H, CH_2), 3.71–3.62 (m, 1H, CH), 3.59 (dd, *J* = 9.0, 7.1 Hz, 2H, CH_2), 2.63–2.52 (m, 2H, CH_2), 1.78–1.67 (m, 1H, CH_2), 1.66–1.56 (m, 1H, CH_2), 1.09 (d, *J* = 6.4 Hz, 3H, CH_3). ^{13}C NMR δ (151 MHz, DMSO-d_6) δ 159.1, 154.7, 141.5, 140.4, 130.2, 129.4 (d), 128.6 (d), 128.5 (d), 126.5, 123.4 (d), 50.4, 48.1, 45.6, 37.2, 32.7, 20.9.

1-[1-(4-Methylphenyl)imidazolin-2-yl]-3-(4-phenylbut-2-yl)urea (7c)

Compound 7c was prepared according to the general procedure using 1-(4-methylphenyl)-2-iminoimidazolidine, 4-phenylbutyl-2-amine and triphosgene; a white solid with 38% yield was obtained. Formula $C_{21}H_{26}N_4O$ (m.m. calc. 351.2179). M.p. 134–134°C. HRMS (ESI) *m/z* $[M+H]^+$: 351.2234. ^{1}H NMR (600 MHz, DMSO-d_6) δ 8.67 (s, 1H, NH), 7.66–7.62 (m, 2H, Ar-H), 7.58–7.52 (m, 1H, Ar-H), 7.49 (d, *J* = 8.5 Hz, 1H, NH), 7.38–7.35 (m, 2H, Ar-H), 7.23–7.19 (m, 4H, Ar-H), 4.08 (dd, *J* = 9.0, 6.9 Hz, 2H, CH_2), 3.75–3.71 (m, 1H, CH), 3.56 (dd, *J* = 9.3, 7.1 Hz, 2H, CH_2), 2.69–2.60 (m, 2H, CH_2), 2.31 (s, 3H, CH_3), 1.63–1.55 (m, 2H, CH_2), 1.08 (d, *J* = 6.8 Hz, 3H, CH_3). ^{13}C NMR δ 159.2, 154.0, 141.0, 140.0, 135.0, 129.8 (d), 128.8 (d), 128.4 (d), 126.4, 120.7 (d), 50.2, 48.2, 45.7, 37.0, 32.5, 20.8, 20.8.

1-[1-(4-Methoxyphenyl)imidazolin-2-yl]-3[4-(4-methylphenyl)but-2-yl)urea (7d)

Compound 7d was prepared according to the general procedure using 1-(4-methoxyphenyl)-2-iminoimidazolidine, 4-(4-methylphenyl)butyl-2-amine and triphosgene; a white solid with 32% yield was obtained. Formula $C_{22}H_{28}N_4O_2$ (m.m. calc. 381.2285). M.p. 131–132°C. HRMS (ESI) *m/z* $[M+H]^+$: 381.1758. ^{1}H NMR (600 MHz, DMSO-d_6) δ 8.55 (s, 1H, NH), 7.68–7.51 (m, 2H, Ar-H), 7.28–7.16 (m, 6H, Ar-H), 6.53 (d, *J* = 7.9 Hz, 1H, NH), 4.08 (dd, *J* = 9.3, 7.4 Hz, 2H, CH_2), 3.87–3.82 (m, 1H, CH), 3.77 (dd, *J* = 9.4, 7.2 Hz, 2H, CH_2), 3.57 (s, 3H, OCH_3), 2.64–2.52 (m, 2H, CH_2), 2.34 (s, 3H, CH_3), 1.72–1.61 (m, 1H, CH_2), 1.60–1.56 (m, 1H, CH_2), 1.08 (d, *J* = 6.3 Hz, 3H, CH_3). ^{13}C NMR δ 159.1, 155.9, 154.5, 138.6, 137.5, 137.0, 129.0 (d), 128.8 (d), 122.4 (d), 115.6 (d), 55.4, 50.4, 48.2, 45.7, 37.0, 32.1, 21.0, 20.8.

1-[1-(4-Metylphenyl)imidazolin-2-yl]-3[4-(4-methylphenyl)but-2-yl)urea (7e)

Compound 7e was prepared according to the general procedure using 1-(4-methylphenyl)-2-iminoimidazolidine, 4-(4-methylphenyl)butyl-2-amine and triphosgene; a white solid with 30% yield was obtained. Formula $C_{22}H_{28}N_4O$ (m.m. calc. 365.2336). M.p. 120–121°C. HRMS (ESI) *m/z* $[M+H]^+$: 365.2142. ^{1}H NMR (600 MHz, DMSO-d_6) δ 8.67 (s, 1H, NH), 7.71 (d, *J* = 7.5 Hz, 2H, Ar-H), 7.63–7.44 (m, 4H, Ar-H), 7.43–7.35 (m, 2H, Ar-H), 6.51 (d, *J* = 8.1 Hz, 1H, NH), 3.98 (dd, *J* = 9.0, 7.5 Hz, 2H, CH_2), 3.87–3.82 (m, 1H, CH), 3.67 (dd, *J* = 9.2, 7.5 Hz, 2H, CH_2), 2.63–2.58 (m, 2H, CH_2), 2.36 (s, 3H, CH_3), 2.29 (s, 3H, CH_3), 1.67–1.58 (m, 2H, CH_2),

1.08 (d, *J* = 6.0 Hz, 3H). ^{13}C NMR δ 159.2, 154.7, 139.9, 138.6, 137.4, 135.0, 129.8 (d), 129.0 (d), 128.9 (d), 120.7 (d), 50.5, 48.1, 45.6, 37.1, 32.1, 21.1, 21.0, 20.8.

1-[1-(4-Metoxyphenyl)imidazolin-2-yl]-3[4-(4-chlorophenyl)but-2-yl)urea (7f)

Compound 7f was prepared according to the general procedure using 1-(4-methoxyphenyl)-2-iminoimidazolidine, 4-(4-chlorophenyl)butyl-2-amine and triphosgene; a white solid with 39% yield was obtained. Formula $C_{21}H_{25}ClN_4O_2$ (m.m. calc. 401.1739). M.p. 168–169°C. HRMS (ESI) *m/z* [M+H]$^+$: 401.1365. ^{1}H NMR (600 MHz, DMSO-d$_6$) δ 8.18 (s, 1H, NH), 7.68–7.49 (m, 6H, Ar-H),7.39–7.35 (m, 2H, Ar-H), 6.67 (d, *J* = 8.6 Hz, 1H, NH), 4.14 (dd, *J* = 8.8, 7.1 Hz, 2H, CH$_2$), 3.90–3.86 (m, 1H, CH), 3.71 (s, 3H, OCH$_3$), 3.58 (dd, *J* = 9.5, 7.4 Hz, 2H, CH$_2$), 2.71–2.63 (m, 2H, CH$_2$), 1.94–1.90 (m, 1H, CH$_2$), 1.89–1.86 (m, 1H, CH$_2$), 1.18 (d, *J* = 6.9 Hz, 3H, CH$_3$). ^{13}C NMR δ 159.1, 155.9, 154.4, 139.6, 137.1, 132.6, 130.3 (d), 128.6 (d), 122.4 (d), 115.4 (d), 55.5, 50.4, 48.1, 45.4, 37.0, 32.6, 21.1.

1-[1-(4-Methylphenyl)imidazolin-2-yl]-3[4-(4-chlorophenyl)but-2-yl)urea (7g)

Compound 7g was prepared according to the general procedure using 1-(4-methylphenyl)-2-iminoimidazolidine, 4-(4-chlorophenyl)butyl-2-amine and triphosgene; a white solid with 45% yield was obtained. Formula $C_{21}H_{25}ClN_4O$ (m.m. calc. 385.1790). M.p. 176–177°C. HRMS (ESI) *m/z* [M+H]$^+$: 385.1604. ^{1}H NMR (600Hz, DMSO-d$_6$) δ ppm 8.68 (s, 1H, NH), 7.81–7.53 (m, 8H, Ar-H), 6.82 (d, *J* = 7.8 Hz, 1H, NH) 4.22 (dd, *J* = 8.8, 6.9 Hz, 2H, CH$_2$), 3.84–3.78 (m, 1H, CH), 3.59 (dd, *J* = 9.0, 7.0 Hz, 2H, CH$_2$), 2.76–2.70 (m, 2H, CH$_2$), 2.32 (s, 3H, CH$_3$), 1.94–1.87 (m, 2H, CH$_2$), 1.22 (d, *J* = 5.9 Hz, 3H, CH$_3$). ^{13}C NMR δ 159.3, 154.6, 139.9, 139.5, 135.0, 132.6, 130.3 (d), 129.7 (d), 128.6 (d), 120.7 (d), 50.5, 48.0, 45.6, 37.2, 32.5, 21.0, 20.6.

1-[1-(4-Methoxyphenyl)imidazolin-2-yl]-3[4-(4-fluorophenyl)but-2-yl)urea (7h)

Compound 7h was prepared according to the general procedure using 1-(4-methoxyphenyl)-2-iminoimidazolidine, 4-(4-fluorophenyl)butyl-2-amine and triphosgene; a white solid with 42% yield was obtained. Formula $C_{21}H_{25}FN_4O_2$ (m.m. calc. 385.2034). M.p. 183–184°C. HRMS (ESI) *m/z* [M+H]$^+$: 385.2501. ^{1}H NMR (600 MHz, DMSO-d$_6$) δ 8.93 (s, 1H, NH), 7.77–7.55 (m, 2H, Ar-H), 7.48–7.32 (m, 4H, Ar-H), 7.30–6.28 (m, 2H, Ar-H), 6.75 (d, *J* = 7.7 Hz, 1H, NH), 3.98 (dd, *J* = 9.1, 7.4 Hz, 2H, CH$_2$), 3.83–3.80 (m, 1H, CH), 3.71 (s, 3H, OCH$_3$), 3.43 (dd, *J* = 9.0, 7.5 Hz, 2H, CH$_2$), 2.68–2.61 (m, 2H, CH$_2$), 1.42–1.35 (m, 2H, CH$_2$), 1.11 (d, *J* = 6.7 Hz, 3H, CH$_3$). ^{13}C NMR δ 162.4, 159.1, 155.7, 154.5, 137.4, 137.4, 137.0, 130.6 (d), 130.5, 122.3 (d), 115.4 (2 x d), 115.3, 55.3, 50.5, 48.0, 45.8, 37.0, 31.7, 20.8.

1-[1-(4-Methylphenyl)imidazolin-2-yl]-3[4-(4-fluorophenyl)but-2-yl)urea (7i)

Compound 7i was prepared according to the general procedure using 1-(4-methylphenyl)-2-iminoimidazolidine, 4-(4-fluorophenyl)butyl-2-amine and triphosgene; a white solid with 41%

yield was obtained. Formula $C_{21}H_{25}FN_4O$ (m.m. calc. 369.2085). M.p. 189–190°C. HRMS (ESI) *m/z* $[M+H]^+$: 369.2069. ^{1}H NMR (600 MHz, DMSO-d_6) δ 8.12 (s, 1H, NH), 7.87–7.83 (m, 2H, Ar-H), 7.50–7.38 (m, 4H, Ar-H), 7.03–6.96 (m, 2H, Ar-H), 6.62 (d, *J* = 8.9 Hz, 1H, NH), 4.13 (dd, *J* = 9.6, 7.5 Hz, 2H, CH_2), 3.88–3.84 (m, 1H, CH) 3.55 (dd, *J* = 9.4, 7.5 Hz, 2H, CH_2), 2.81–2.78 (m, 2H, CH_2), 2.28 (s, 3H, CH_3), 1.69–1.54 (m, 2H, CH_2), 1.12 (d, *J* = 6.7 Hz, 3H, CH_3) ^{13}C NMR δ 160.4, 159.2, 154.6, 140.0, 137.4, 135.1, 130.7 (d), 130.5, 129.7 (d), 120.7 (d), 115.3, 50.4, 48.2, 45.5, 37.1, 31.7, 21.0, 20.7.

1-[1-(4-Methylphenyl)imidazolin-2-yl]-3[4-(4-methoxyphenyl)but-2-yl)urea (7j)

Compound 7j was prepared according to the general procedure using 1-(4-methylphenyl)-2-iminoimidazolidine, 4-(4-methoxyphenyl)butyl-2-amine and triphosgene; a white solid with 37% yield was obtained. Formula $C_{22}H_{28}N_4O_2$ (m.m. calc. 381.2285). M.p. 139–140°C. HRMS (ESI) *m/z* $[M+H]^+$: 381.234. ^{1}H NMR (600 MHz, DMSO-d_6) δ 8.92 (s, 1H, NH), 7.68–7.40 (m, 6H, Ar-H), 7.35 (d, *J* = 8.3 Hz, 2H, Ar-H), 6.72 (d, *J* = 8.3 Hz, 1H, NH), 4.08 (dd, *J* = 9.4, 7.5 Hz, 2H, CH_2), 3.87–3.85 (m, 1H, CH), 3.74 (s, 3H, OCH_3), 3.56 (dd, *J* = 9.4, 7.5 Hz, 2H, CH_2), 2.58–2.53 (m, 2H, CH_2), 2.31 (s, 3H, CH_3), 1.76–1.61 (m, 2H, CH_2), 1.08 (d, *J* = 6.5 Hz, 3H, CH_3). ^{13}C NMR δ 159.3, 158.0, 154.6, 139.9, 135.0, 134.7, 129.7 (2 x d), 129.6, 120.6 (d), 114.0 (d), 55.3, 50.6, 48.0, 45.5, 37.0, 32.3, 21.0, 20.7.

1-[1-(4-Methylphenyl)imidazolin-2-yl]-3[4-(furan-2-yl)but-2-yl)urea (7k)

Compound 7k was prepared according to the general procedure using 1-(4-methylphenyl)-2-iminoimidazolidine, 4-(furan-2-yl)butyl-2-amine and triphosgene; a white solid with 37% yield was obtained. Formula $C_{19}H_{24}N_4O_2$ (m.m. calc. 341.1972). M.p. 166–167°C. HRMS (ESI) *m/z* $[M+H]^+$: 341.2591. ^{1}H NMR (600 MHz, DMSO-d_6) δ 8.61 (s, 1H, NH), 7.46–7.28 (m, 6H, Ar-H), 6.81 (t, *J* = 6.0 Hz, 1H, Ar-H), 6.38 (d, *J* = 8.2 Hz, 1H, NH), 4.35 (dd, *J* = 9.2, 7.0 Hz, 2H, CH_2), 4.08–4.00 (m, 1H, CH), 3.81 (dd, *J* = 9.0, 7.7 Hz, 2H, CH_2), 2.94–2.89 (m, 2H, CH_2), 2.30 (s, 3H, CH_3), 1.96–1.87 (m, 2H, CH_2), 1.22 (d, *J* = 5.5 Hz, 3H, CH_3). ^{13}C NMR δ 159.3, 155.5, 154.5, 141.6, 139.9, 135.1, 129.7 (d), 120.7 (d), 110.0, 107.8, 50.6, 48.1, 46.2, 33.8, 25.5, 20.8, 20.6.

1-[1-(4-Chlorophenyl)imidazolin-2-yl]-3[4-(furan-2-yl)but-2-yl)urea (7l)

Compound 7l was prepared according to the general procedure using 1-(4-chlorophenyl)-2-iminoimidazolidine, 4-(furan-2-yl)butyl-2-amine and triphosgene; a white solid with 39% yield was obtained. Formula $C_{18}H_{21}ClN_4O_2$ (m.m. calc. 361.1426). M.p. 149–150°C. HRMS (ESI) *m/z* $[M+H]^+$: 361.2080. ^{1}H NMR (600 MHz, DMSO-d_6) δ 8.78 (s, 1H, NH), 7.49–7.28 (m, 6H,

Ar-H), 6.97–6.93 (m, 1H, Ar-H), 6.53 (d, *J* = 8.0 Hz, 1H, NH), 4.38 (dd, *J* = 9.4, 6.9 Hz, 2H, CH_2), 4.14–4.11 (m, 1H, CH), 3.91 (dd, *J* = 8.9, 7.1 Hz, 2H, CH_2), 2.98–2.91 (m, 2H, CH_2), 1.83–1.76 (m, 2H, CH_2) 1.11 (d, *J* = 5.6 Hz, 3H, CH_3). ^{13}C NMR δ 159.3, 155.6, 154.5, 141.5, 140.4, 130.0 (d), 129.4, 123.3 (d), 110.0, 107.8, 50.5, 48.1, 46.1, 33.9, 25.6, 20.8.

1-[1-(4-Methylphenyl)imidazolin-2-yl]-3[4-(thiophen-2-yl)but-2-yl)urea (7m)

Compound 7m was prepared according to the general procedure using 1-(4-methylphenyl)-2-iminoimidazolidine, 4-(thiophen-2-yl)butyl-2-amine and triphosgene; a white solid with 31% yield was obtained. Formula $C_{19}H_{28}N_4OS$ (m.m. calc. 357.1744). M.p. 171–172°C. HRMS (ESI) *m/z* $[M+H]^+$: 357.1744. ^{1}H NMR (600 MHz, DMSO-d_6) δ 8.39 (s, 1H, NH), 7.96–7.92 (m, 2H, Ar-H), 7.62–7.53 (m, 4H, Ar-H), 7.21–7.17 (m, 1H, Ar-H), 6.52 (d, *J* = 7.4 Hz, 1H, NH), 4.18 (dd, *J* = 9.2, 7.4 Hz, 2H, CH_2), 3.88–3.84 (m, 1H, CH), 3.54 (dd, *J* = 9.0, 7.8 Hz, 2H, CH_2), 2.76–2.69 (m, 2H, CH_2), 1.84–1.79 (m, 2H, CH_2), 1.21 (d, *J* = 4.4 Hz, 3H, CH_3). ^{13}C NMR δ 159.2, 154.5, 143.6, 140.0, 140.0, 135.0, 129.8 (d), 127.2, 125.8, 125.2, 120.7 (d), 50.5, 48.1, 46.0, 36.2, 29.5, 20.9, 20.6.

1-[1-(4-Chlorophenyl)imidazolin-2-yl]-3[4-(thiophen-2-yl)but-2-yl)urea (7n)

Compound 7n was prepared according to the general procedure using 1-(4-chlorophenyl)-2-iminoimidazolidine, 4-(thiophen-2-yl)butyl-2-amine and triphosgene; a white solid with 42% yield was obtained. Formula $C_{18}H_{21}ClN_4OS$ (m.m. calc. 377.1197). M.p. 154–155°C. HRMS (ESI) *m/z* $[M+H]^+$: 377.1122. ^{1}H NMR (600 MHz, DMSO-d_6) δ 8.07 (s, 1H, NH), 7.83–7.79 (m, 2H, Ar-H), 7.54–7.52 (m, 2H, Ar-H), 7.13–7.09 (m, 2H, Ar-H), 6.61 (t, *J* = 5.3 Hz, 1H, Ar-H), 6.39 (d, *J* = 7.9 Hz, 1H, NH), 4.18 (dd, *J* = 9.2, 7.7 Hz, 2H, CH_2), 3.88–3.85 (m, 1H, CH), 3.58 (dd, *J* = 9.2, 7.5 Hz, 2H, CH_2), 2.67–2.56 (m, 2H, CH_2), 1.93–1.88 (m, 2H, CH_2), 1.21 (d, *J* = 6.1 Hz, 3H, CH_3). ^{13}C NMR δ 159.1, 154.6 143.7, 140.4, 130.0, 129.4 (d), 127.1, 125.8, 125.8, 125.3, 123.3 (d), 50.5, 48.1, 46.0, 36.2, 29.4, 21.0.

1-[1-(4-Methylphenyl)imidazolin-2-yl]-3[4-(5-methylthiophen-2-yl)but-2-yl)urea (7o)

Compound 7o was prepared according to the general procedure using 1-(4-methylphenyl)-2-iminoimidazolidine, 4-(5-methylthiophen-2-yl)butyl-2-amine and triphosgene; a white solid with 33% yield was obtained. Formula $C_{20}H_{26}N_4OS$ (m.m. calc. 371.1900). M.p. 173–174°C. HRMS (ESI) *m/z* $[M+H]^+$: 371.1632. ^{1}H NMR (600 MHz, DMSO-d_6) δ 8.44 (s, 1H, NH), 7.74–7.67 (m, 2H, Ar-H), 7.48–7.41 (m, 2H, Ar-H), 7.04–6.98 (m, 2H, Ar-H), 6.51 (d, *J* = 7.7 Hz, 1H, NH), 4.48 (dd, *J* = 9.2, 7.4 Hz, 2H, CH_2), 4.16–4.13 (m, 1H, CH), 3.92 (dd, *J* = 9.2, 7.5 Hz, 2H, CH_2), 3.08–2.97 (m, 2H, CH_2), 2.34 (s, 3H, CH_3) 2.22 (s, 3H, CH_3), 1.81–1.73 (m, 2H, CH_2), 1.21 (d, *J* = 5.0 Hz, 3H, CH_3). ^{13}C NMR δ 159.3, 154.5, 143.0, 139.9, 137.5, 135.0, 129.7 (d), 126.0, 125.6, 120.7 (d), 50.5, 48.0, 46.0, 36.0, 30.6, 20.9, 20.6, 15.5.

1-[1-(4-Chlorophenyl)imidazolin-2-yl]-3[4-(5-methylthiophen-2-yl)but-2-yl)urea (7p)

Compound 7p was prepared according to the general procedure using 1-(4-chlorophenyl)-2-iminoimidazolidine, 4-(5-methylthiophen-2-yl)butyl-2-amine and triphosgene; a white solid with 29% yield was obtained. Formula $C_{19}H_{23}ClN_4OS$ (m.m. calc. 391.1354). M.p. 119–120°C. HRMS (ESI) *m/z* $[M+H]^+$: 391.1201. ^1H NMR (600 MHz, DMSO-d_6) δ 8.51 (s, 1H, NH), 7.55–7.49 (m, 4H, Ar-H), 7.12–7.06 (m, 2H, Ar-H), 6.49 (d, *J* = 7.6 Hz, 1H, NH), 4.11 (dd, *J* = 9.2, 7.1 Hz, 2H, CH_2), 3.79–3.72 (m, 1H, CH), 3.61 (dd, *J* = 9.0, 7.3 Hz, 2H, CH_2), 2.92–2.84 (m, 2H, CH_2), 2.29 (s, 3H, CH_3), 1.93–1.85 (m, 2H, CH_2), 1.24 (d, *J* = 5.8 Hz, 3H, CH_3). ^{13}C NMR δ 159.2, 154.8, 142.9, 140.4, 137.4, 130.0, 129.5 (d), 125.9, 125.6, 123.3 (d), 50.5, 48.0, 45.8, 36.0, 30.5, 20.9, 15.4.

Compound 3a

^{13}C NMR spectrum

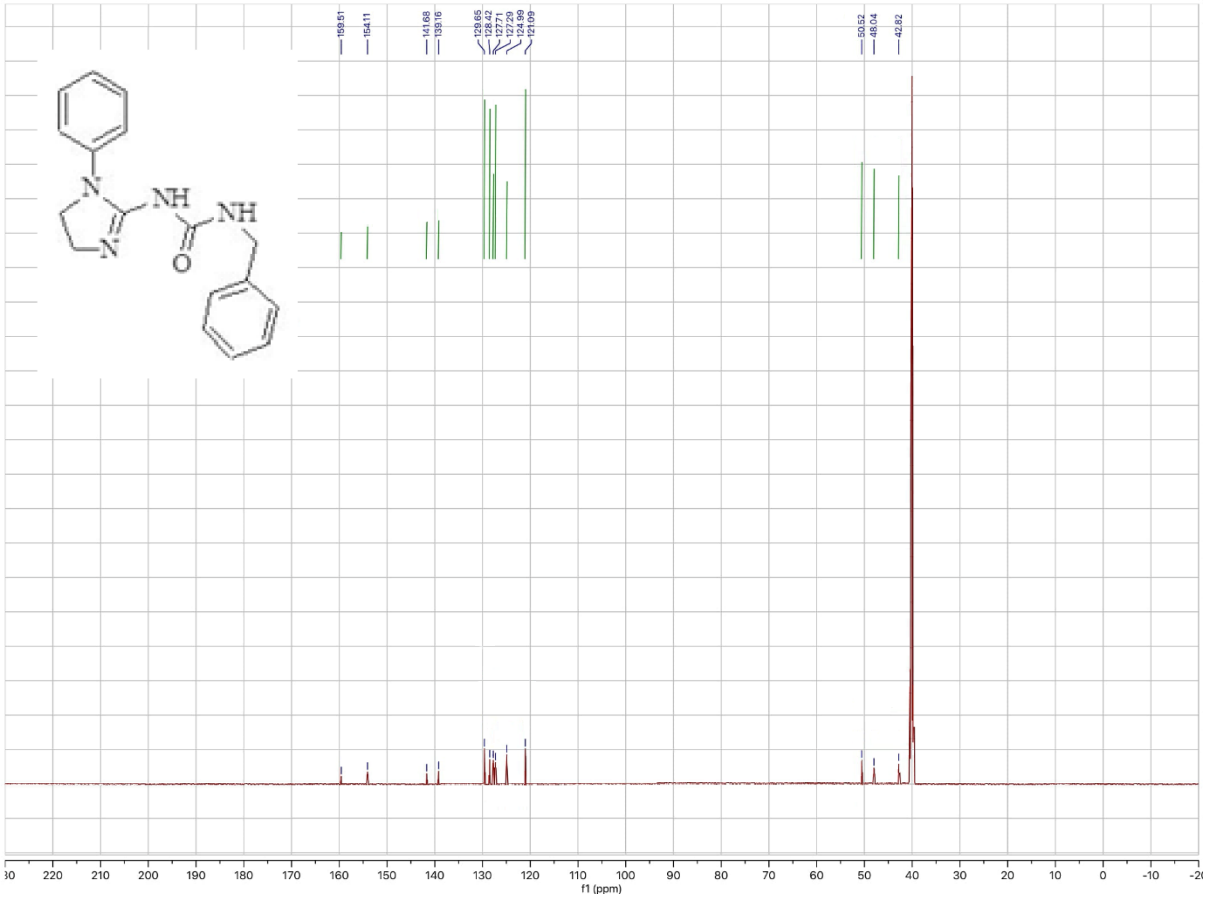

MS spectrum: $C_{17}H_{18}N_4O$ (m.m. calc. 295.1553). HRMS (ESI) *m/z* $[M+H]^+$: 295.1644.

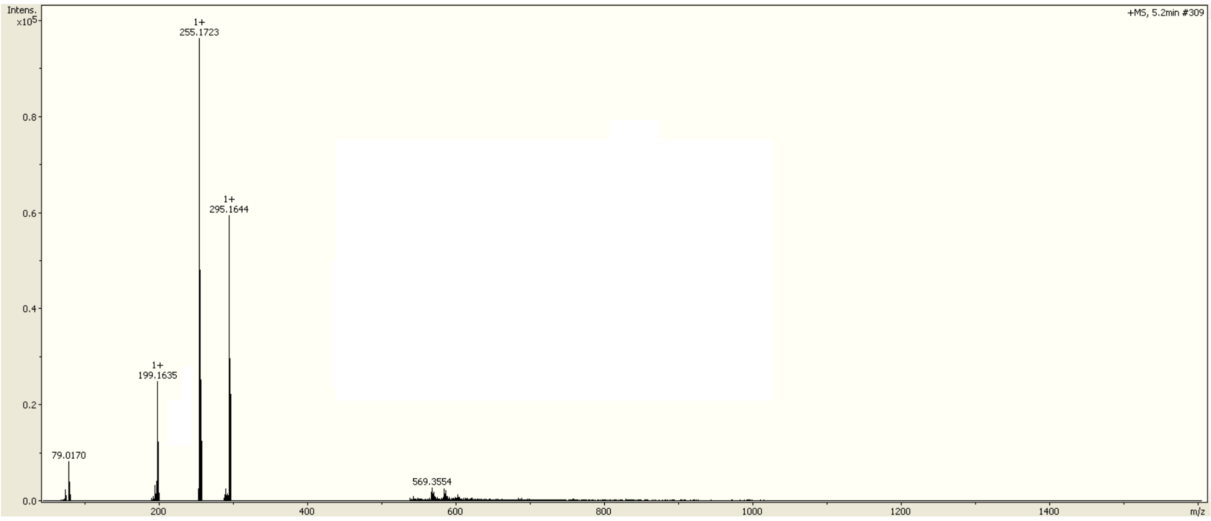

^{1}H NMR spectrum

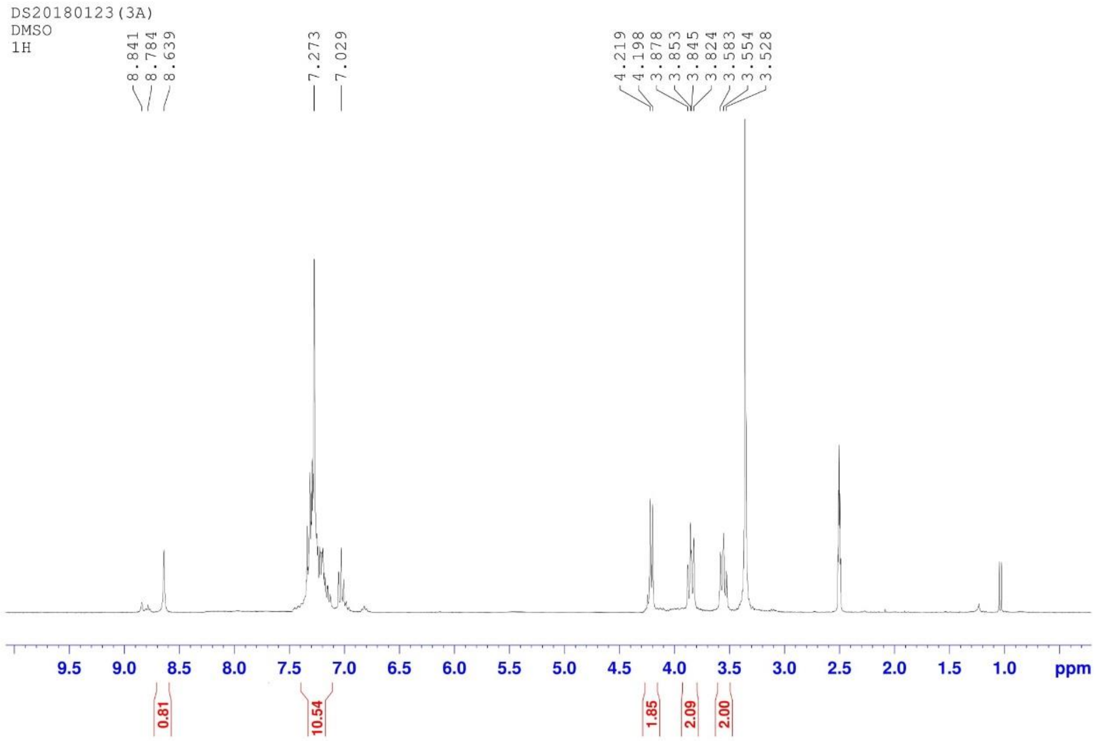

Compound 3b

^{13}C NMR spectrum

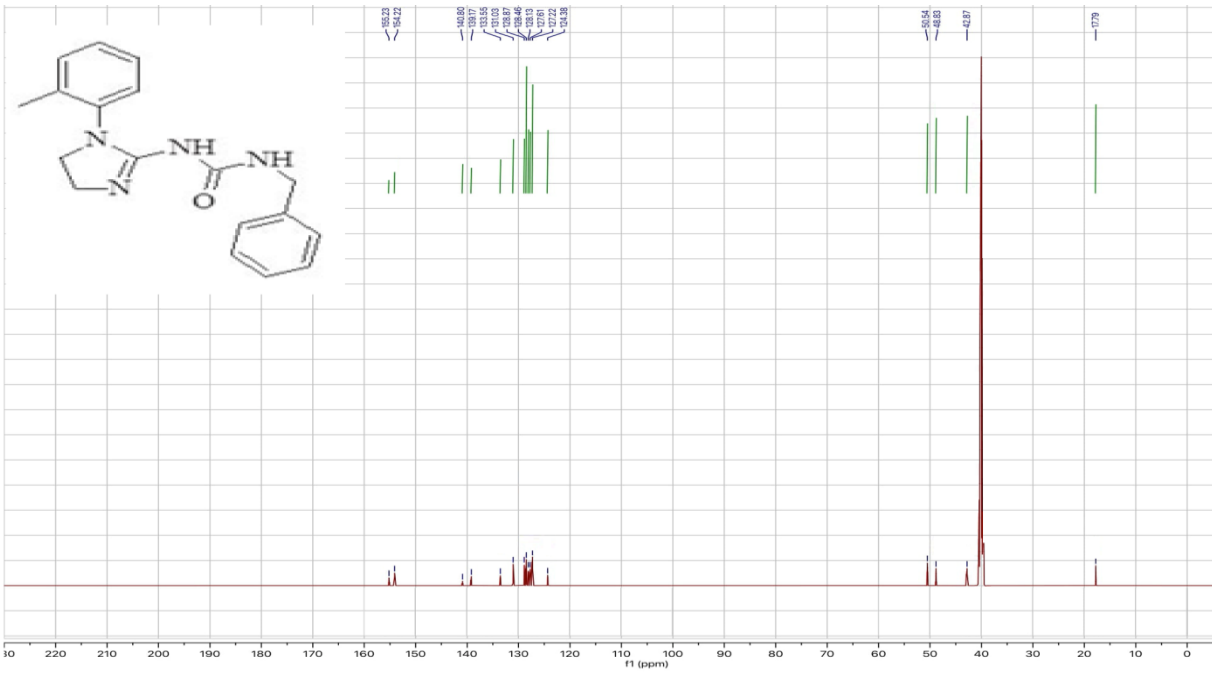

MS spectrum: $C_{18}H_{20}N_4O$ (m.m. calc. 309.1710). HRMS (ESI) *m/z* $[M+H]^+$: 309.1364

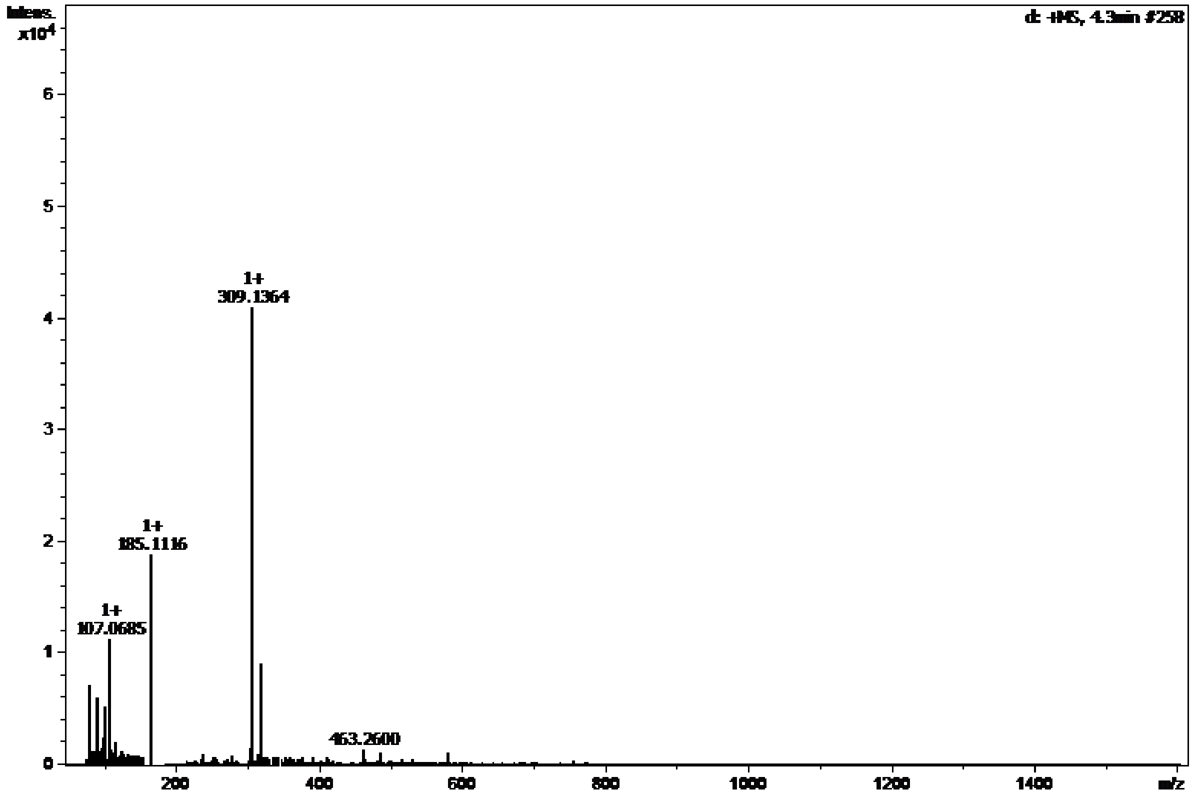

^{1}H NMR spectrum

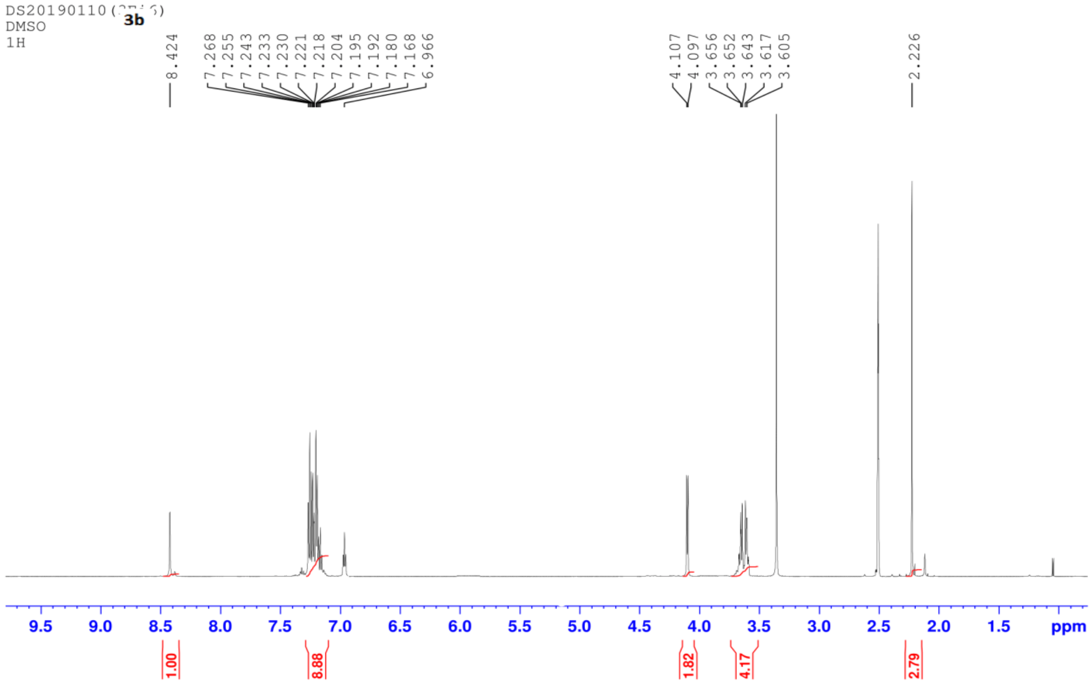

Compound 3c

^{13}C NMR spectrum

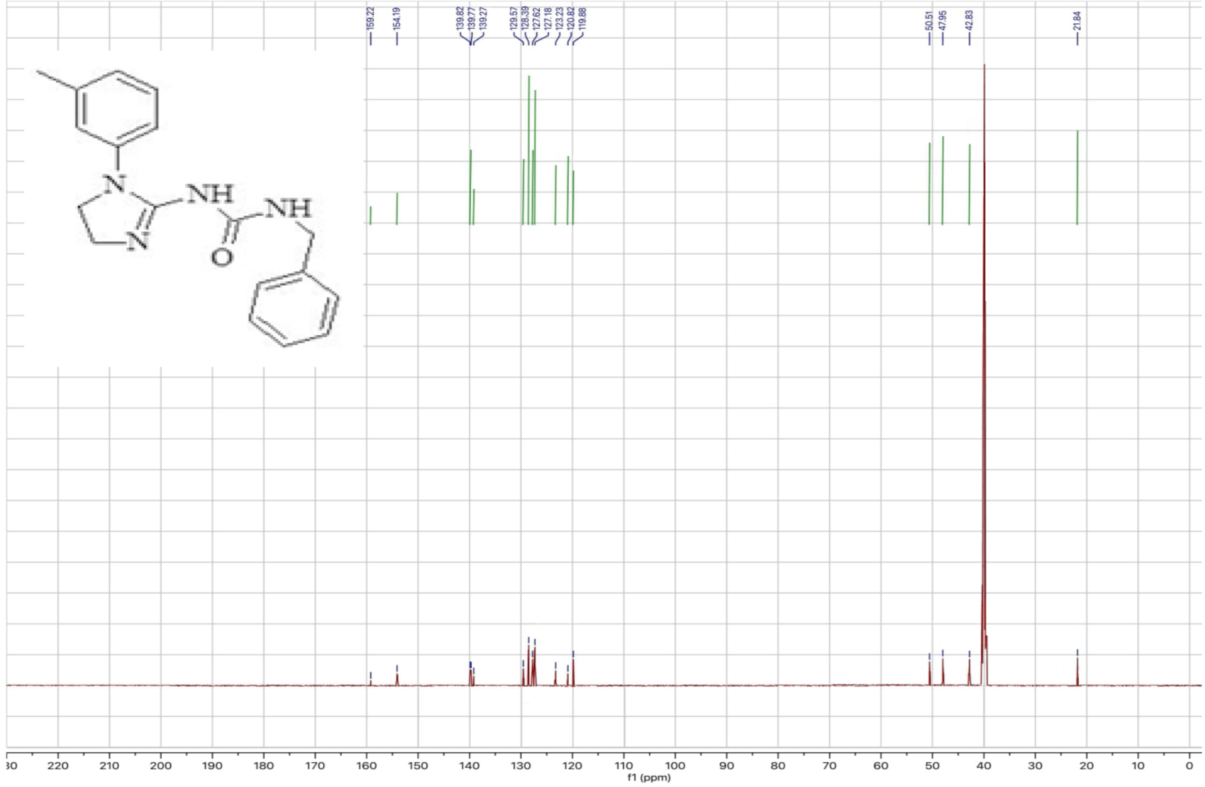

MS spectrum: $C_{18}H_{20}N_4O$ (m.m. calc. 309.1710). HRMS (ESI) *m/z* $[M+H]^+$: 309.1370

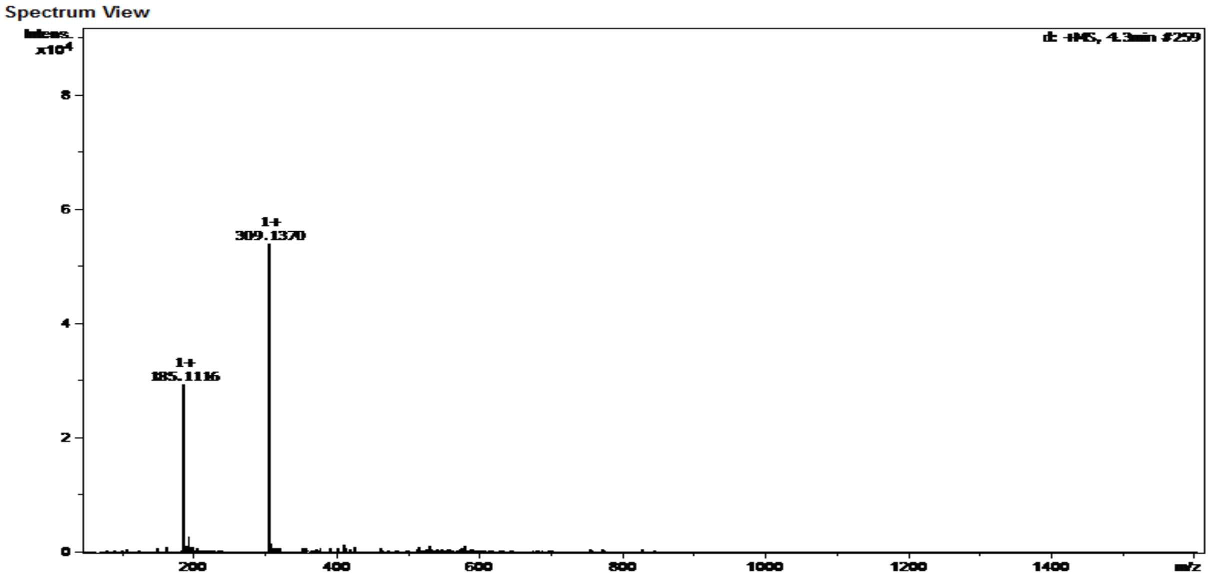

^{1}H NMR spectrum

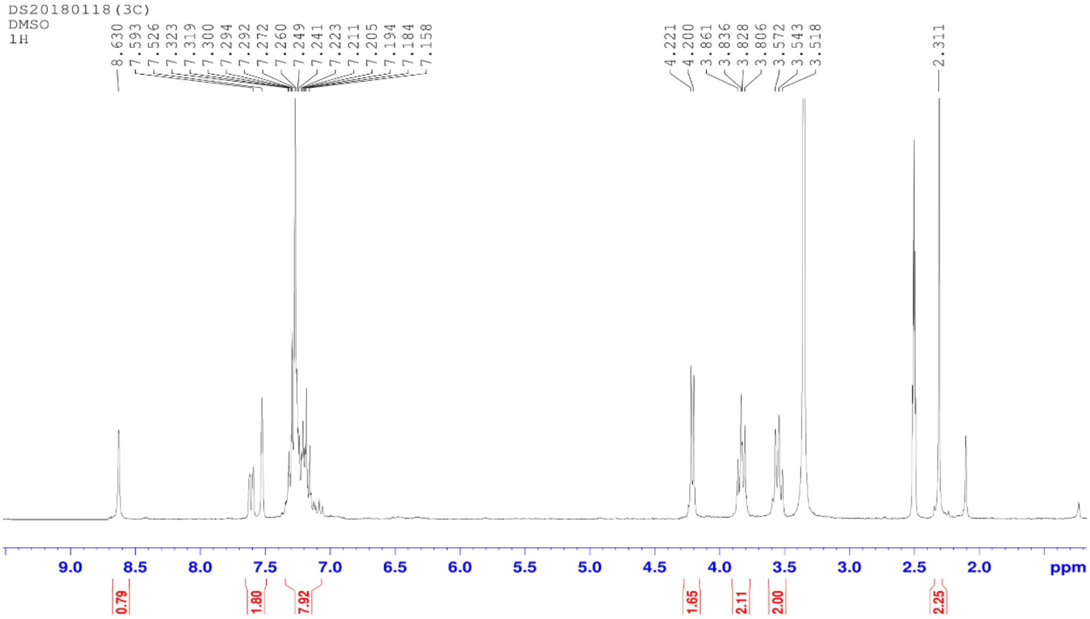

Compound 3d

^{13}C NMR spectrum

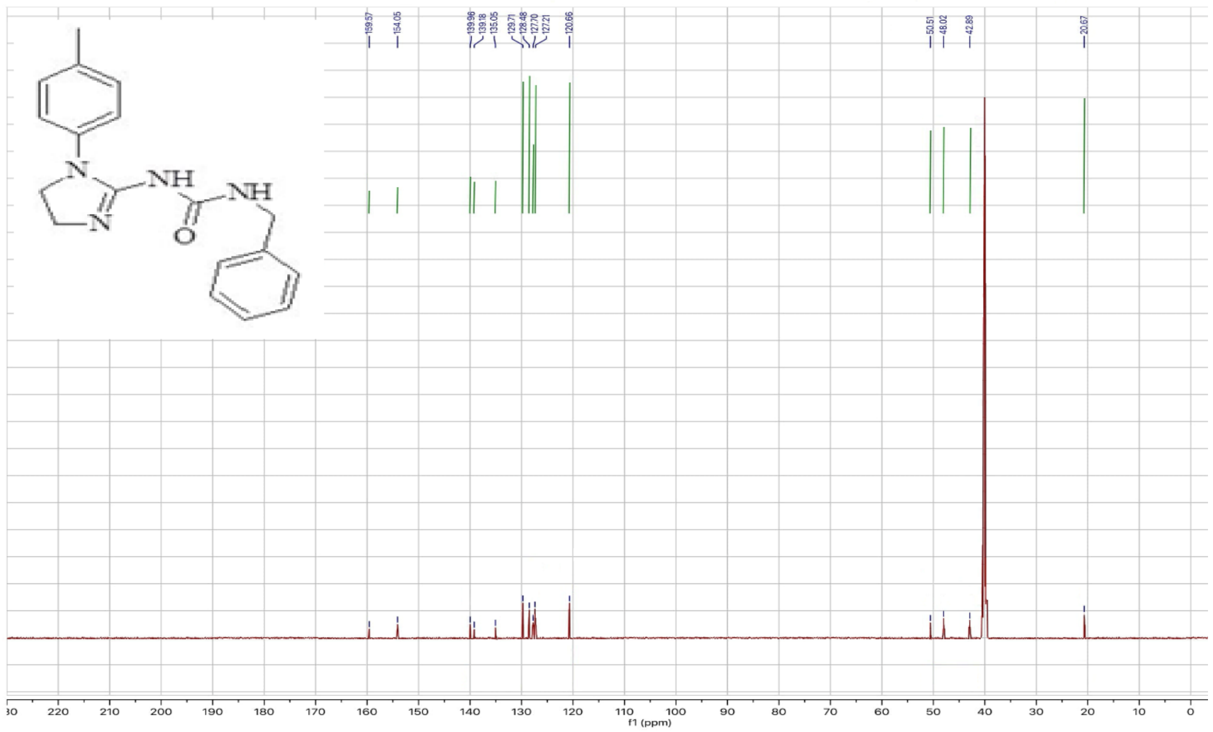

MS spectrum: $C_{18}H_{20}N_4O$ (m.m. calc. 309.1710). HRMS (ESI) *m/z* $[M+H]^+$: 309.1545.

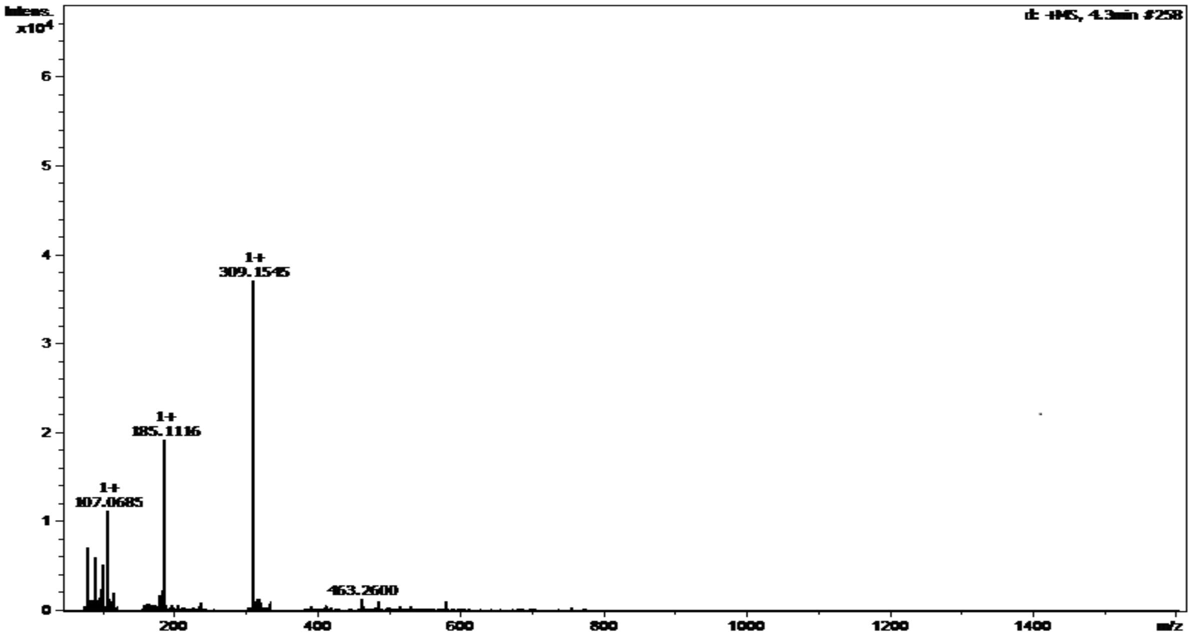

^{1}H NMR spectrum

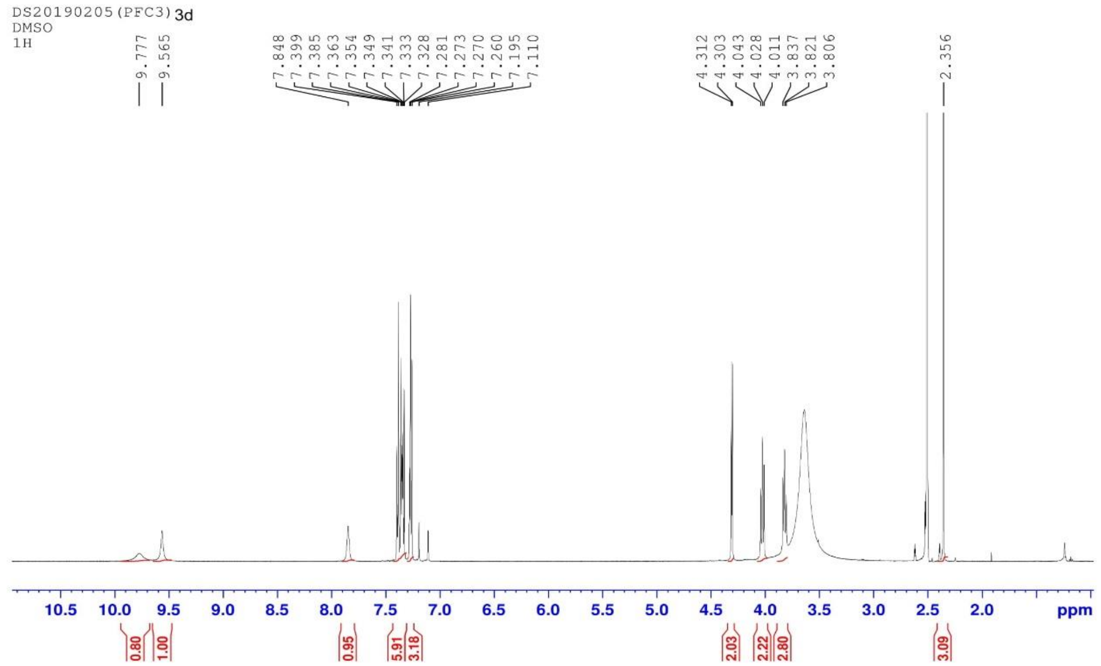

Compound 3e

^{13}C NMR spectrum

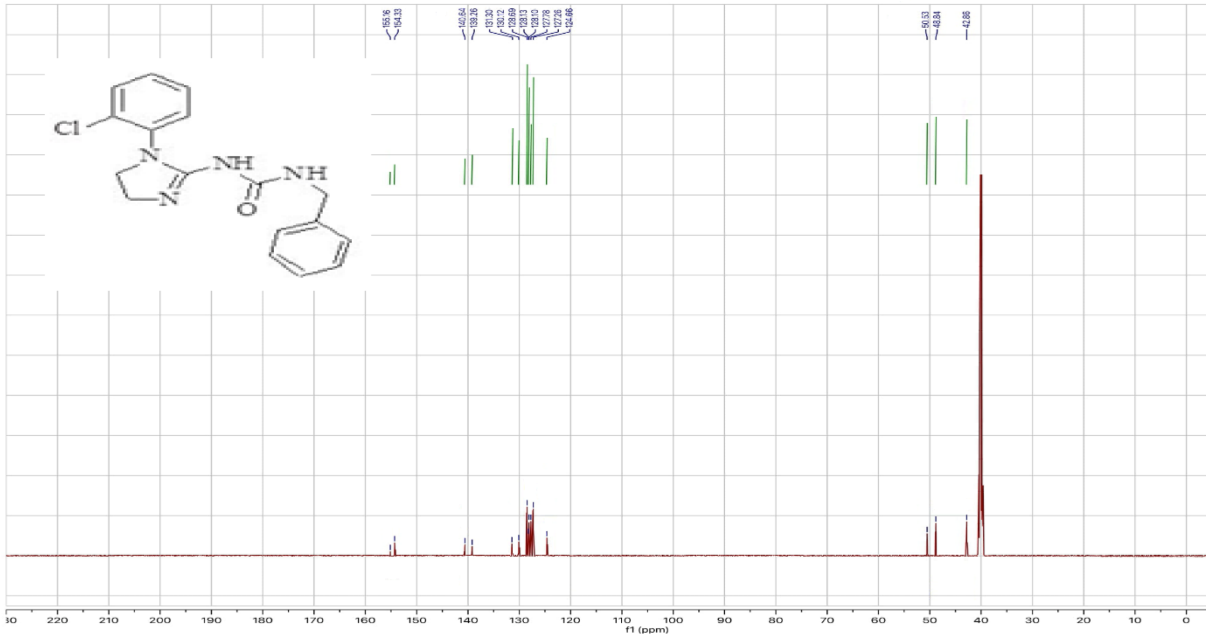

MS spectrum: $C_{17}H_{17}ClN_4O$ (m.m. calc. 329.1164). HRMS (ESI) *m/z* $[M+H]^+$: 329.1158.

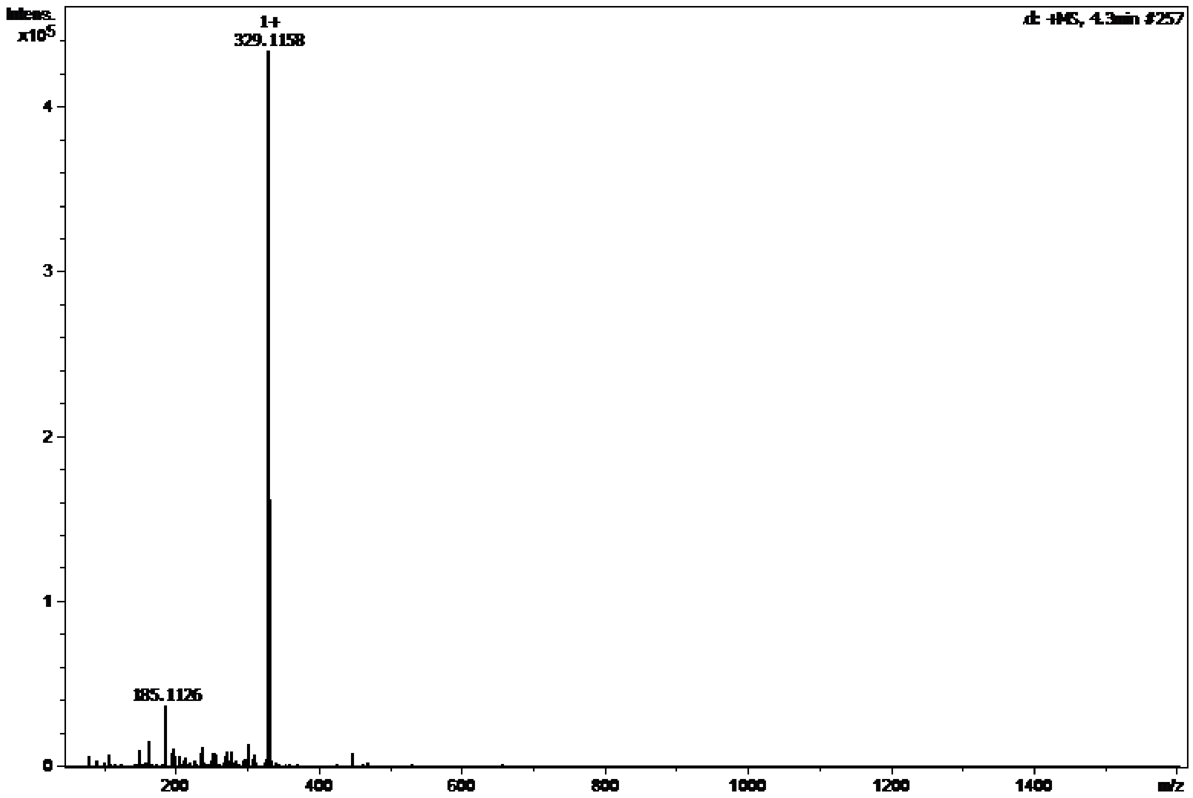

^{1}H NMR spectrum

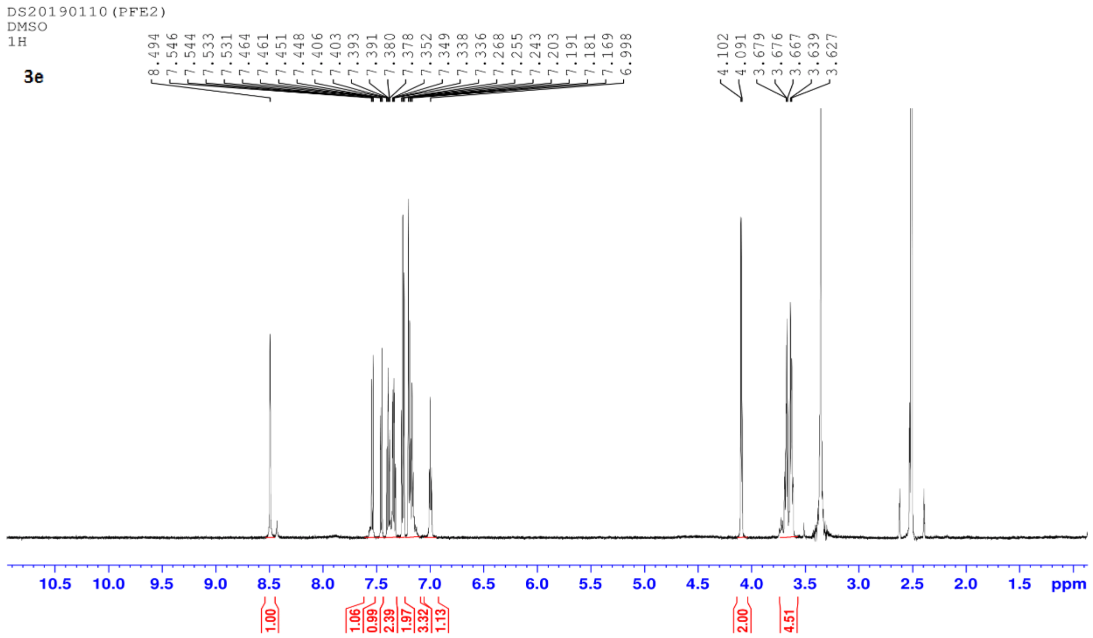

Compound 3f

^{13}C NMR spectrum

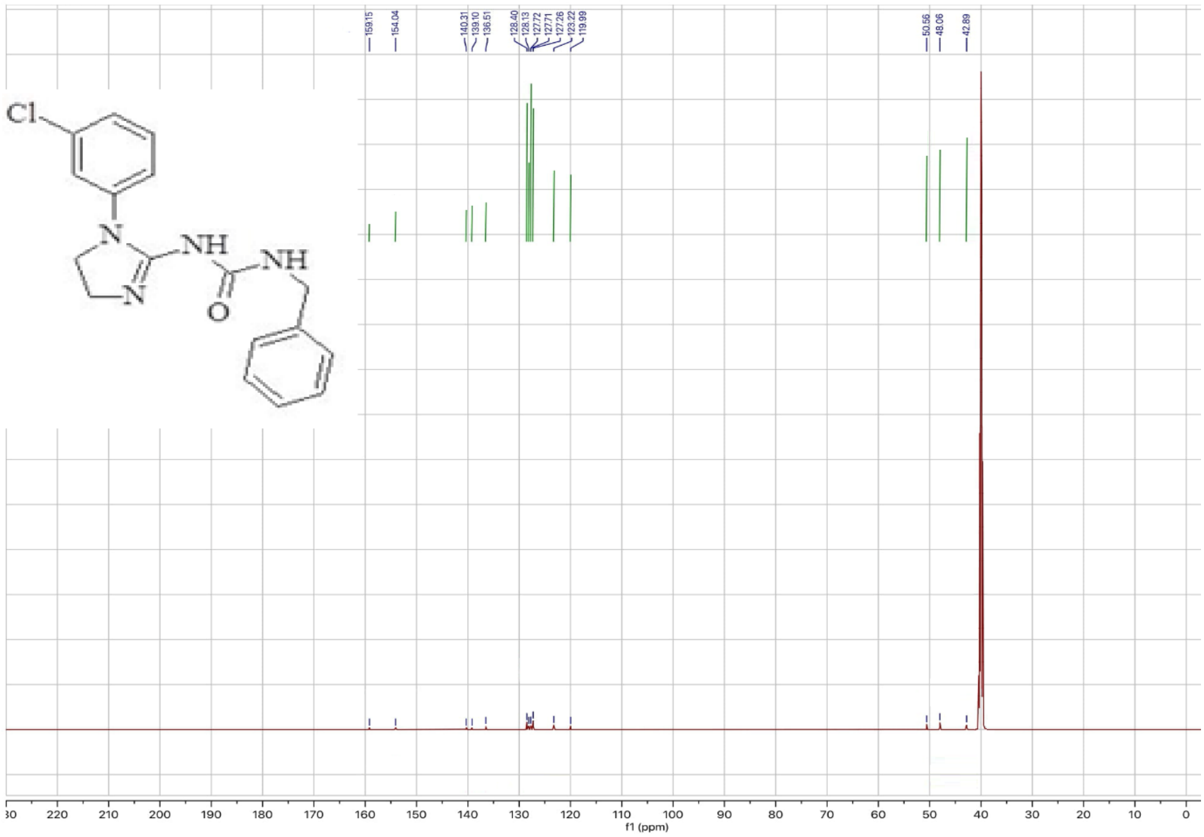

MS spectrum: $C_{17}H_{17}ClN_4O$ (m.m. calc. 329.1164). HRMS (ESI) *m/z* $[M+H]^+$: 329.1231.

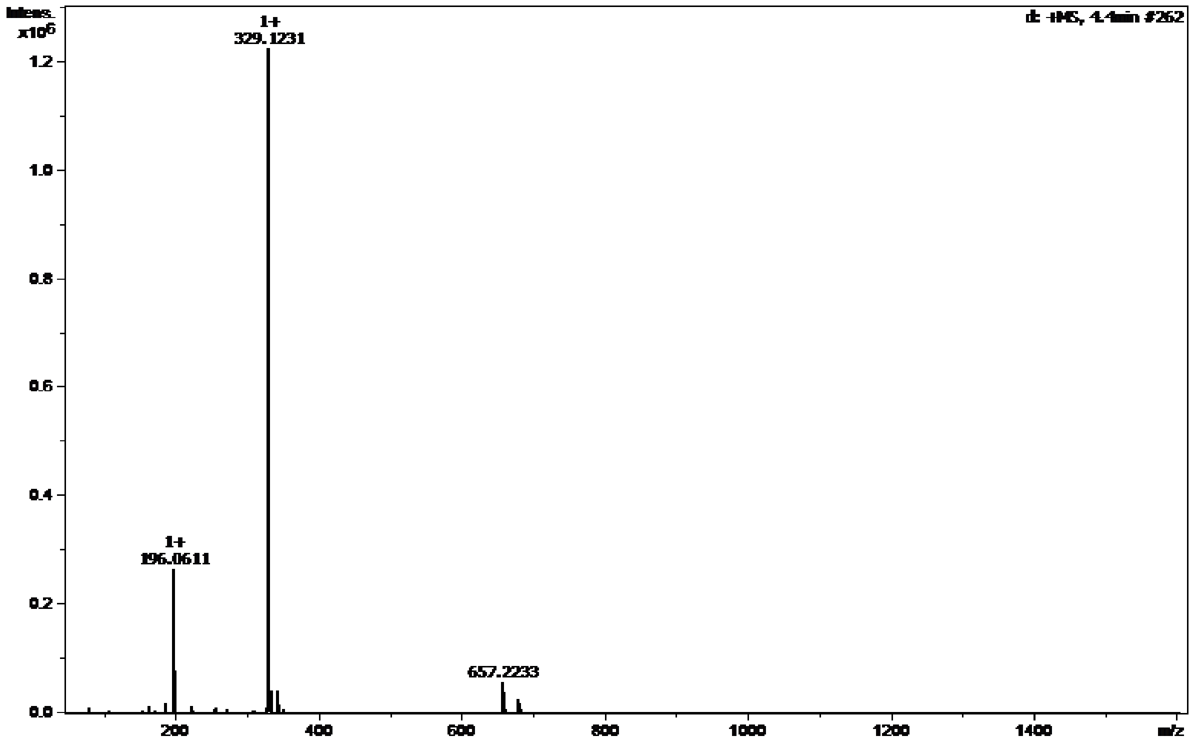

^{1}H NMR spectrum

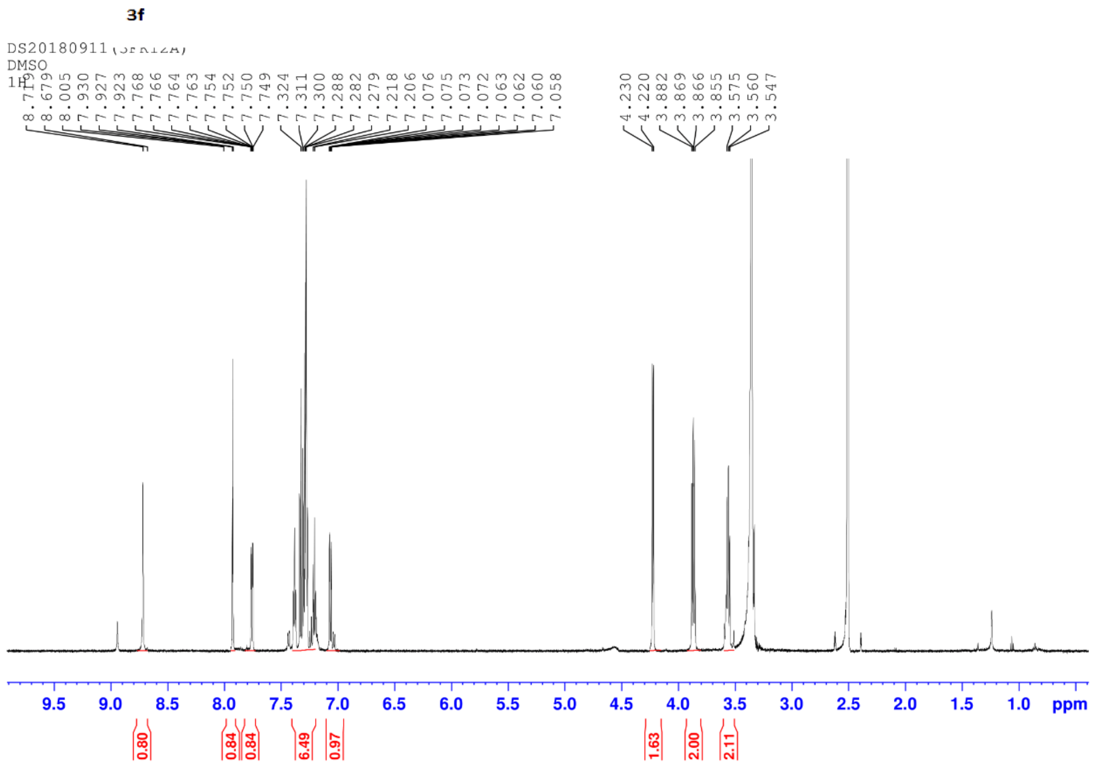

Compound 3g

^{13}C NMR spectrum

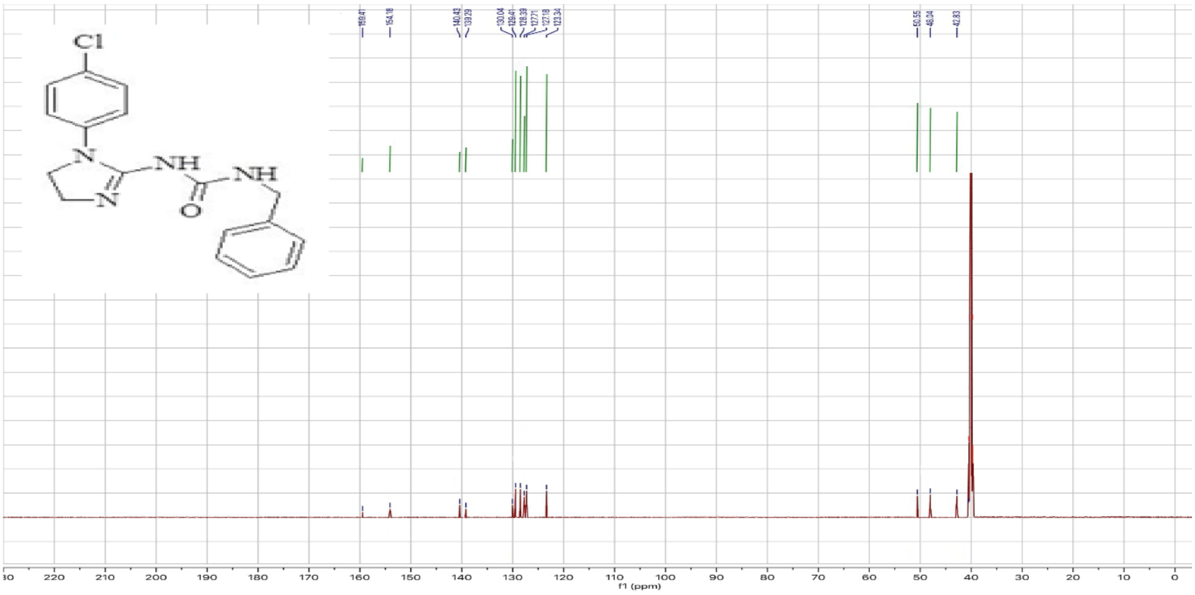

MS spectrum: $C_{17}H_{17}ClN_4O$ (m.m. calc. 329.1164). HRMS (ESI) *m/z* $[M+H]^+$: 329.1158.

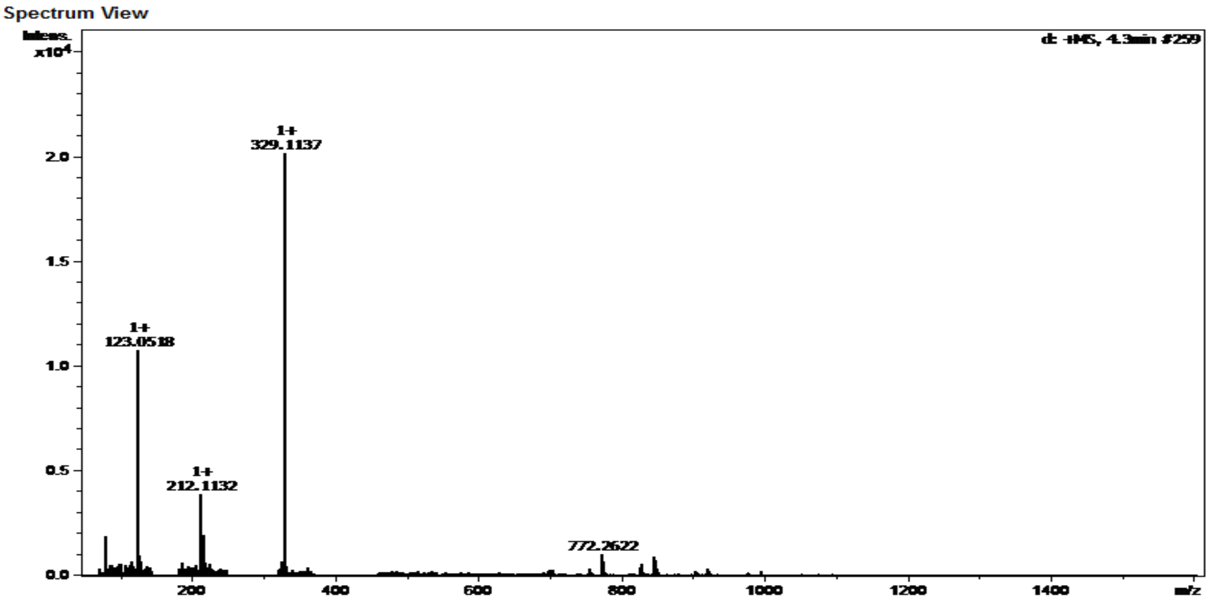

^{1}H NMR spectrum

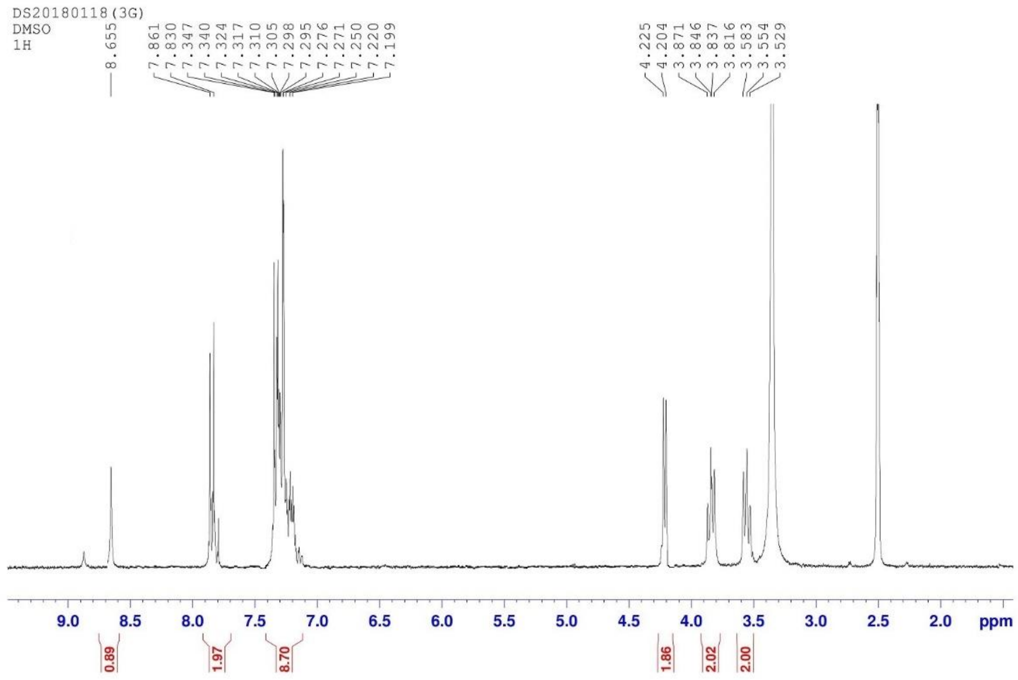

Compound 4a

^{13}C NMR spectrum

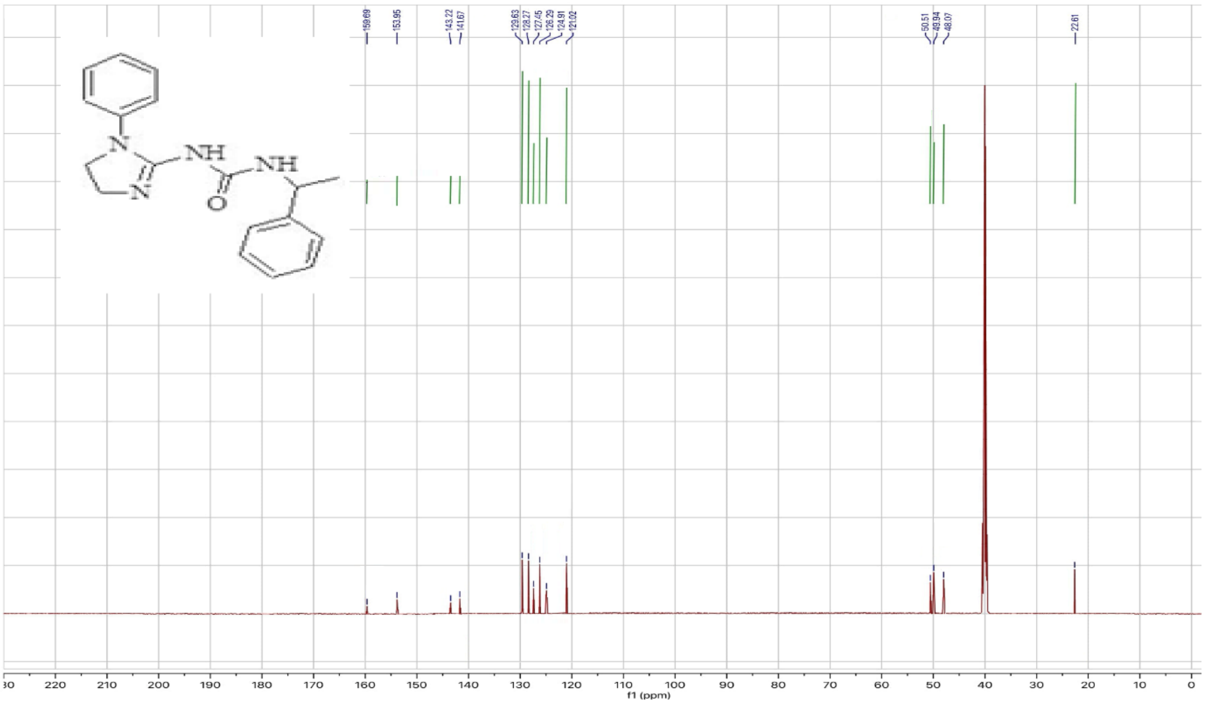

MS spectrum: $C_{18}H_{20}N_4O$ (m.m. calc. 309.1710). HRMS (ESI) *m/z* $[M+H]^+$: 309.1667.

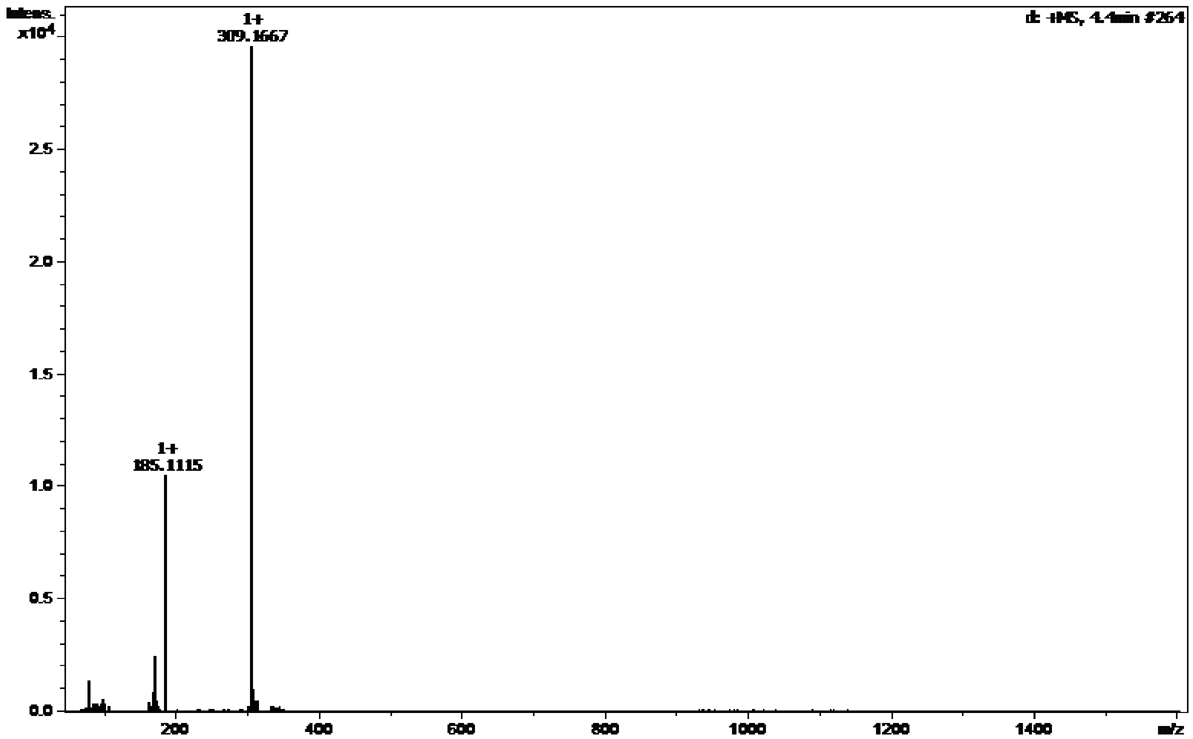

^{1}H NMR spectrum

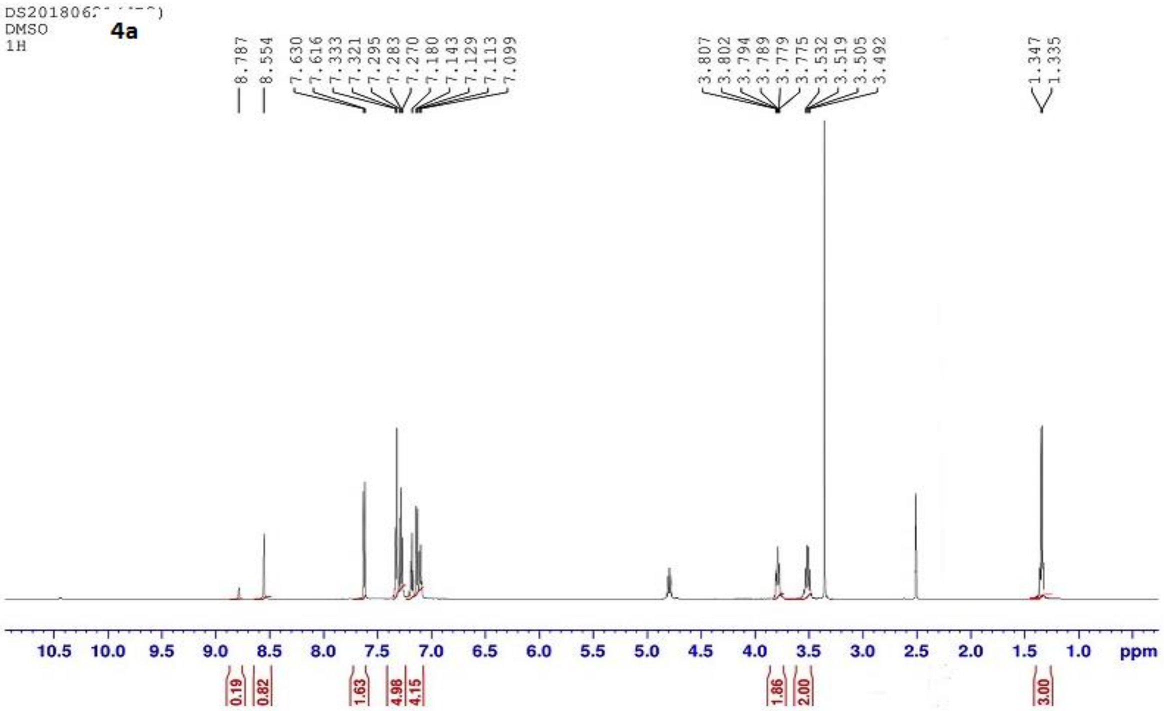

Compound 4b

^{13}C NMR spectrum

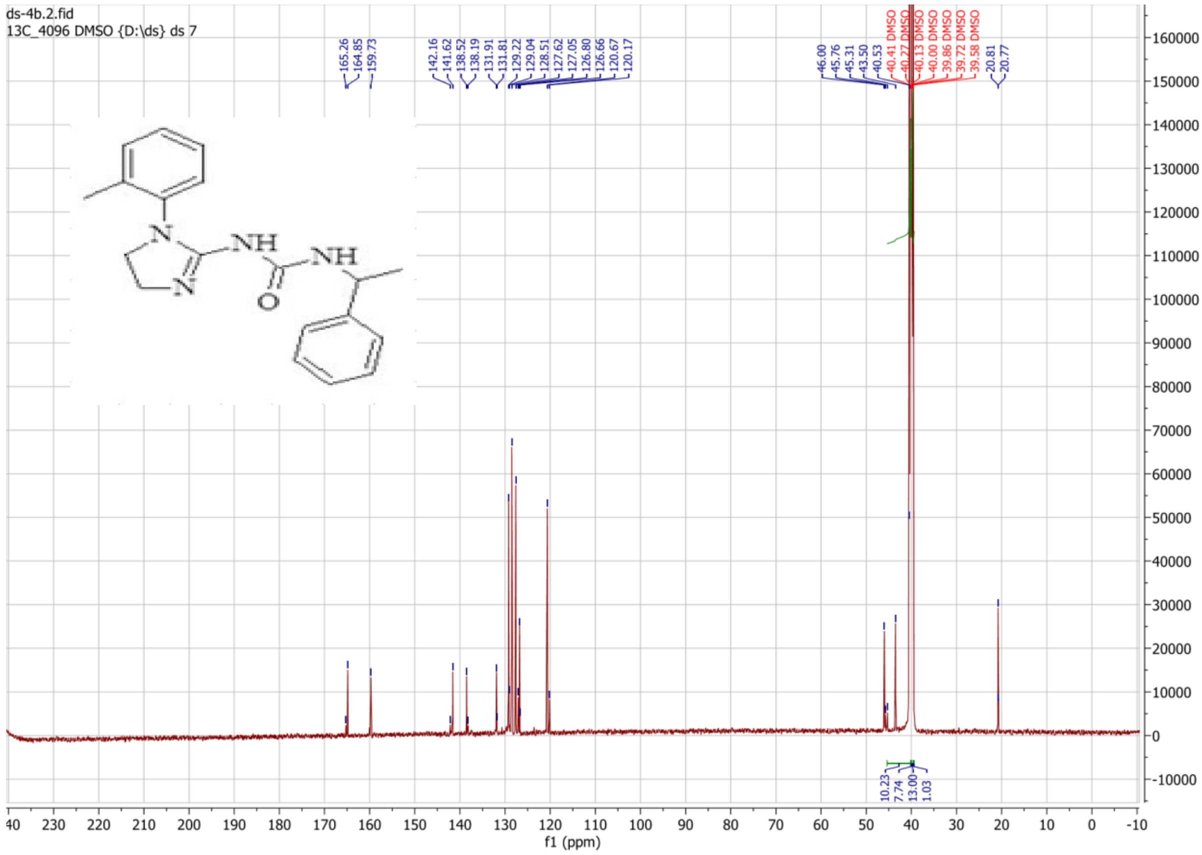

MS spectrum: $C_{19}H_{22}N_4O$ (m.m. calc. 323.1866). HRMS (ESI) *m/z* $[M+H]^+$: 323.1820.

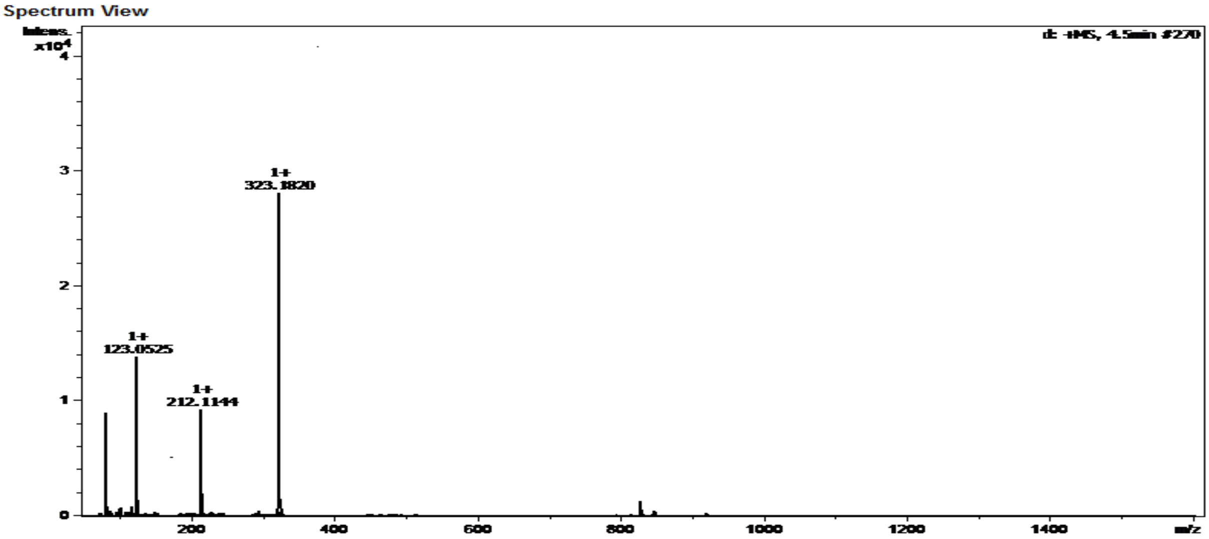

^{1}H NMR spectrum

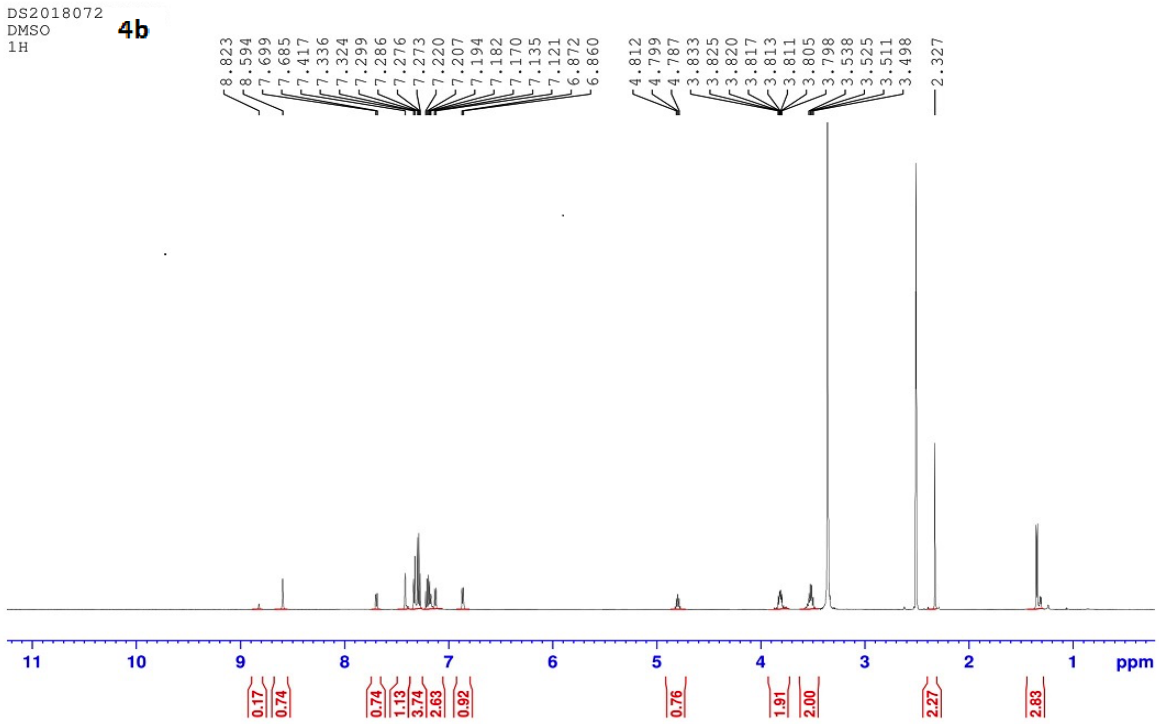

Compound 4c

^{13}C NMR spectrum

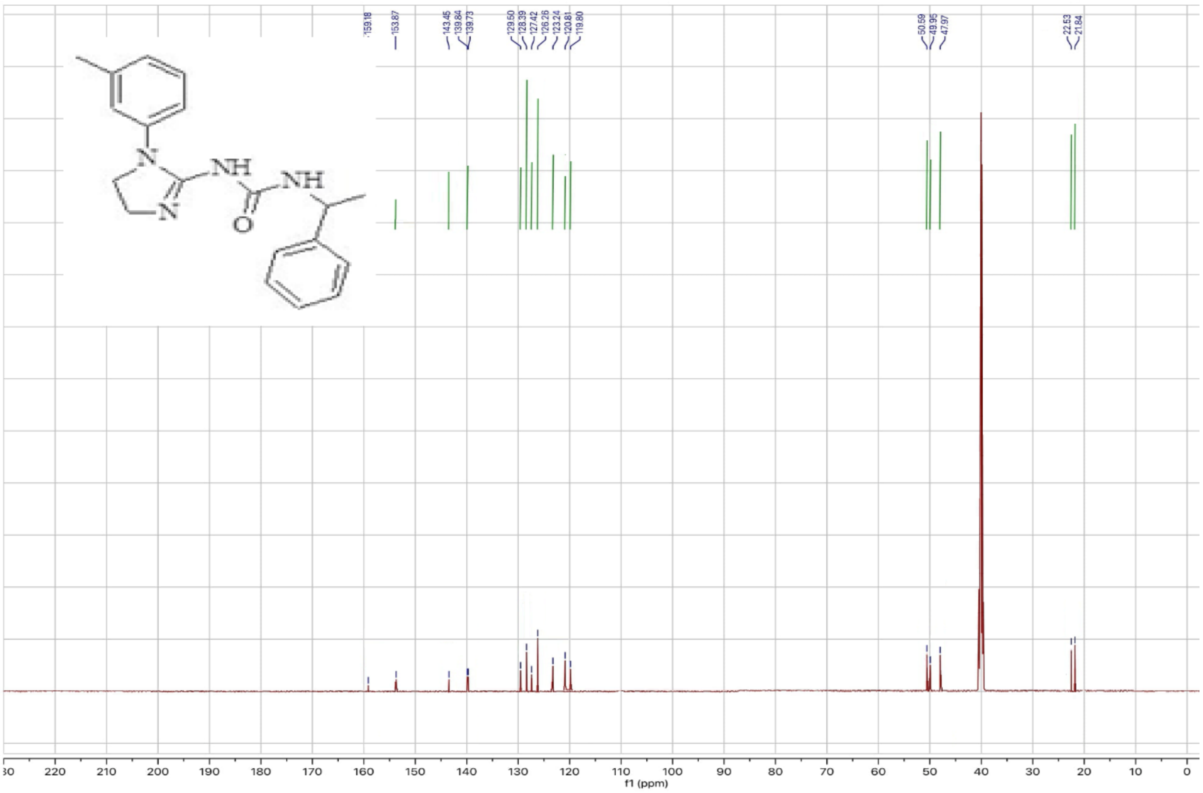

MS spectrum: $C_{19}H_{22}N_4O$ (m.m. calc. 323.1866). HRMS (ESI) m/z $[M+H]^+$: 323.1850.

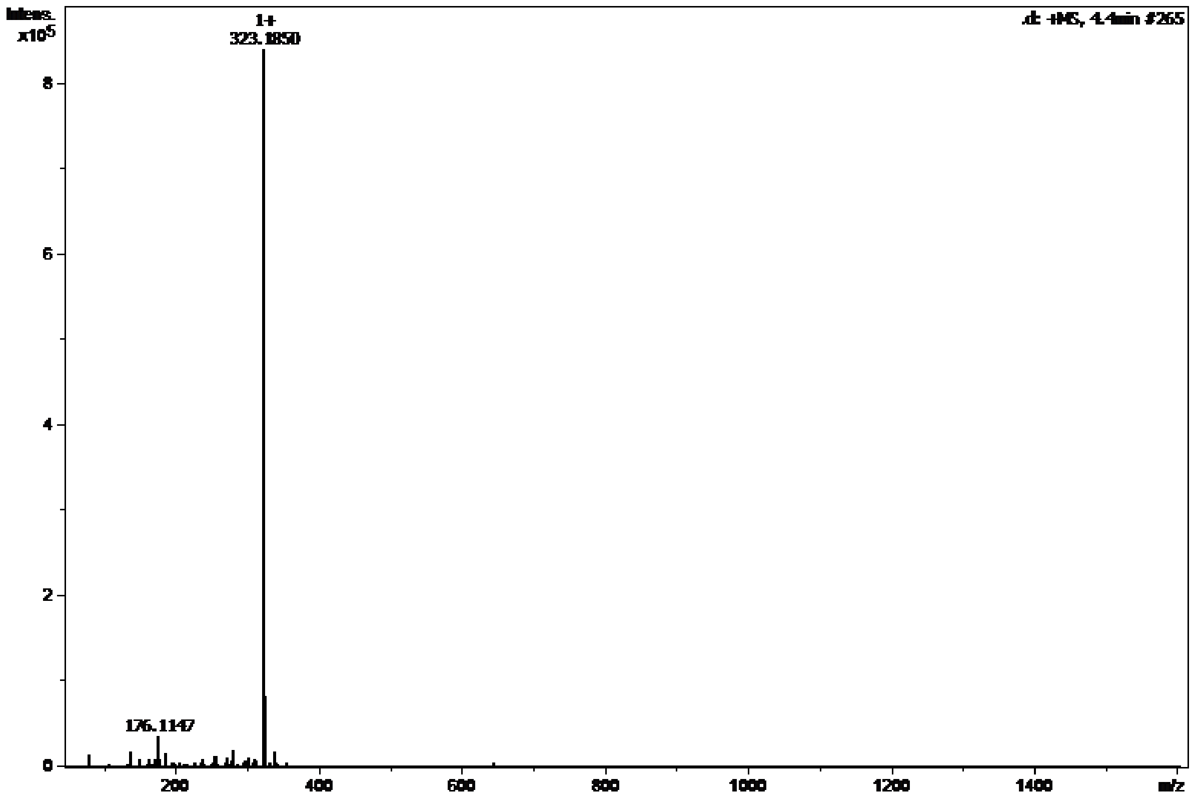

^{1}H NMR spectrum

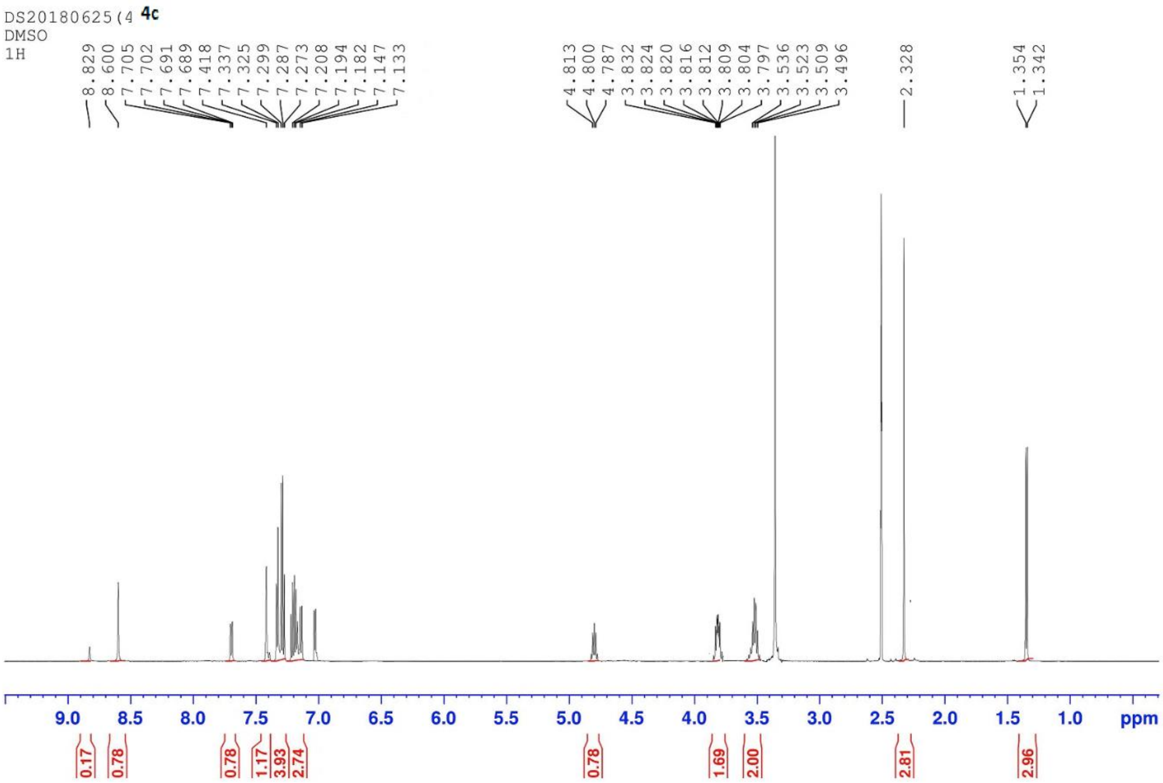

Compound 4d

^{13}C NMR spectrum

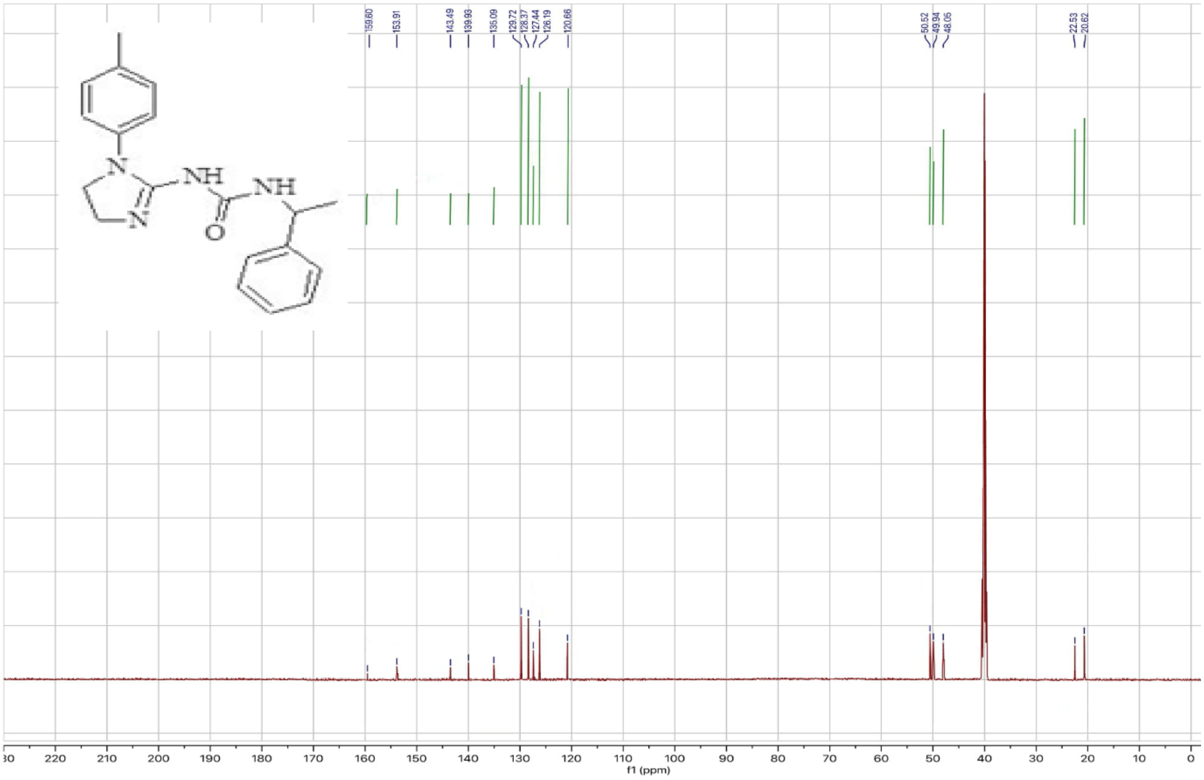

MS spectrum: $C_{19}H_{22}N_4O$ (m.m. calc. 323.1866). HRMS (ESI) *m/z* $[M+H]^+$: 323.1545.

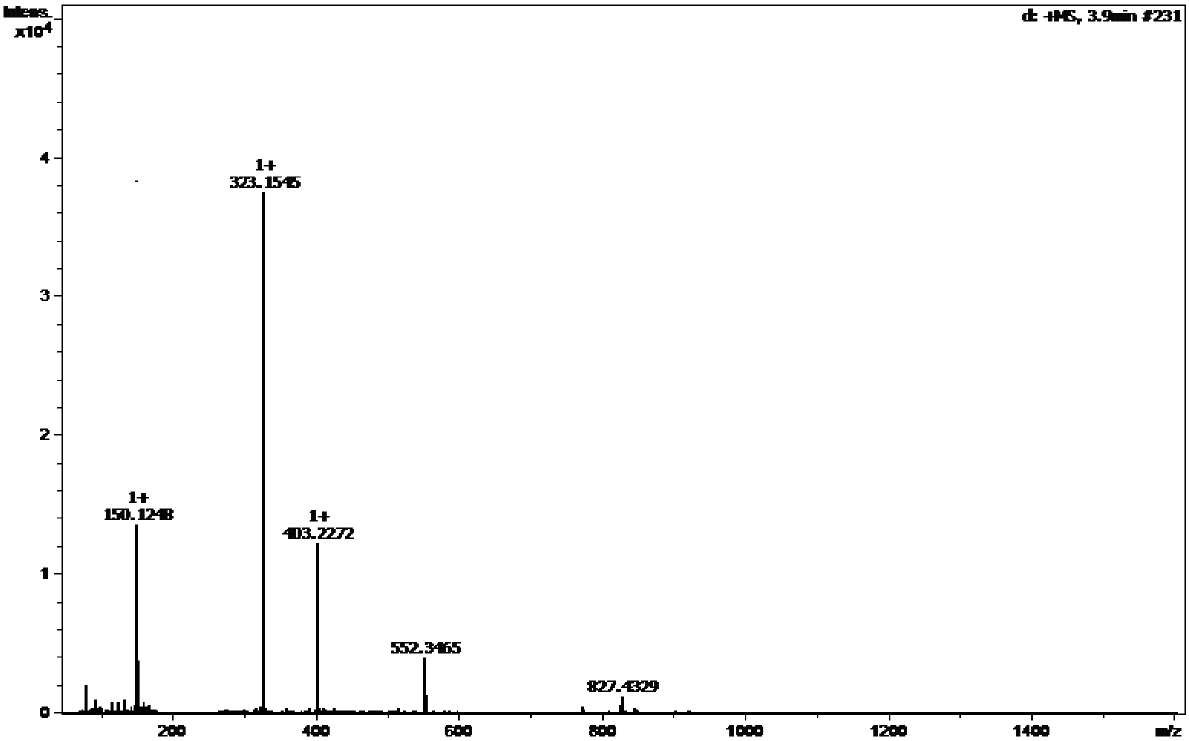

^{1}H NMR spectrum

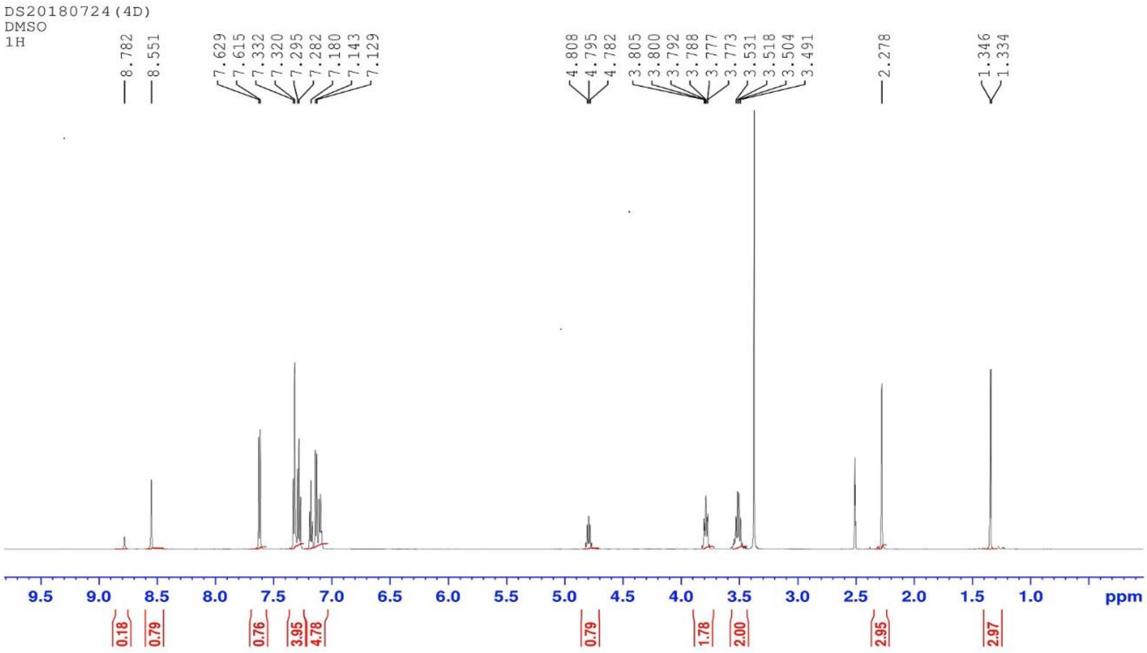

Compound 4e

^{13}C NMR spectrum

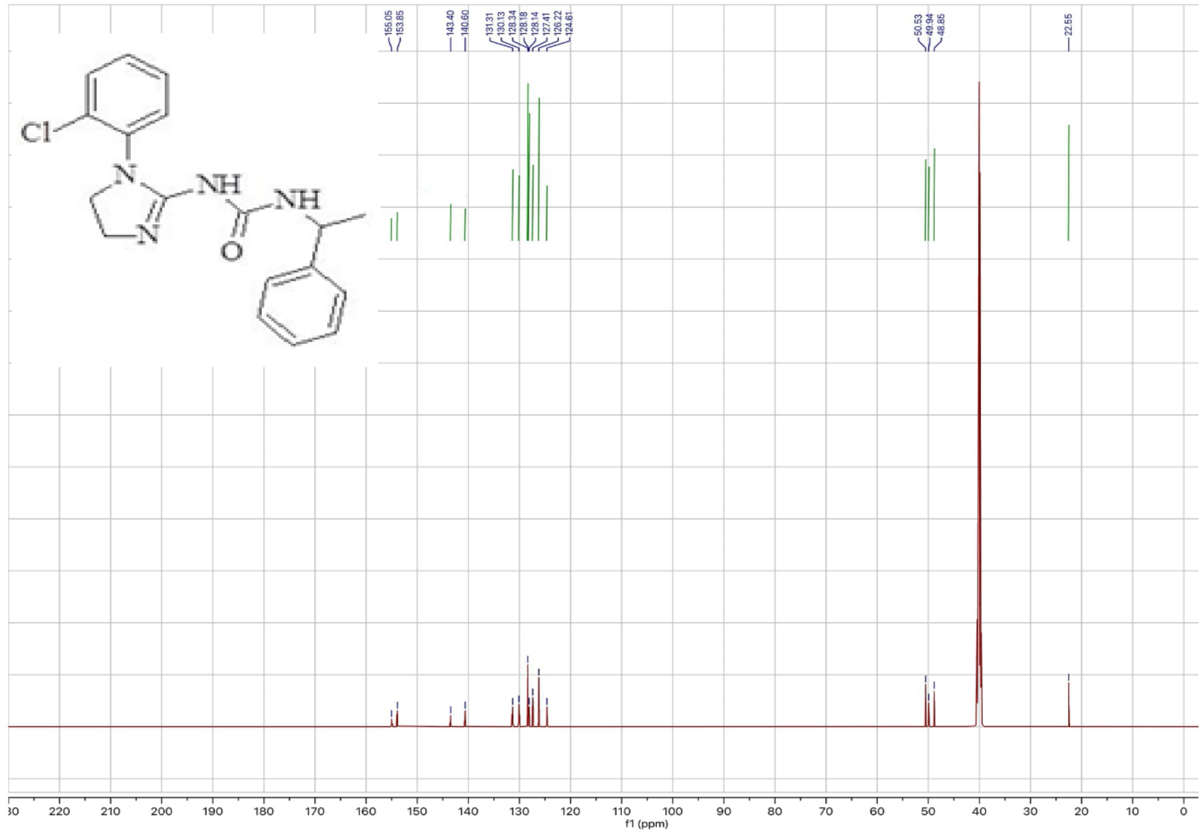

MS spectrum: $C_{18}H_{19}ClN_4O$ (m.m. calc. 343.1320). HRMS (ESI) *m/z* [M+H]$^+$: 343.2094.

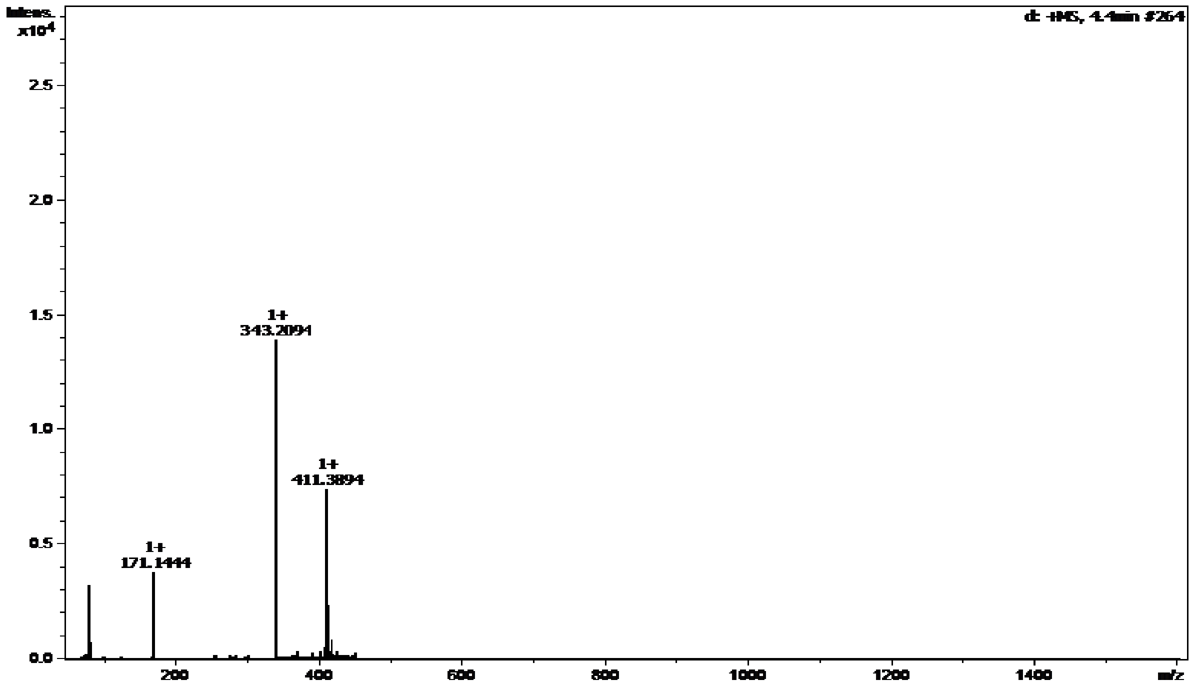

^1H NMR spectrum

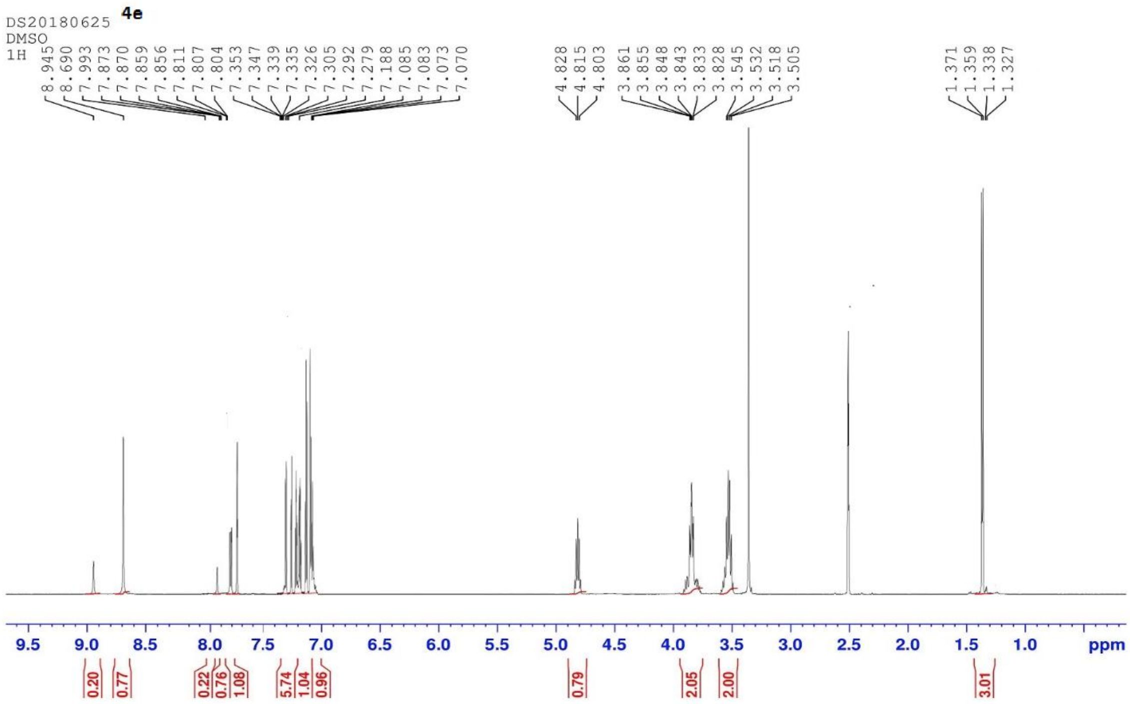

Compound 4f

^{13}C NMR spectrum

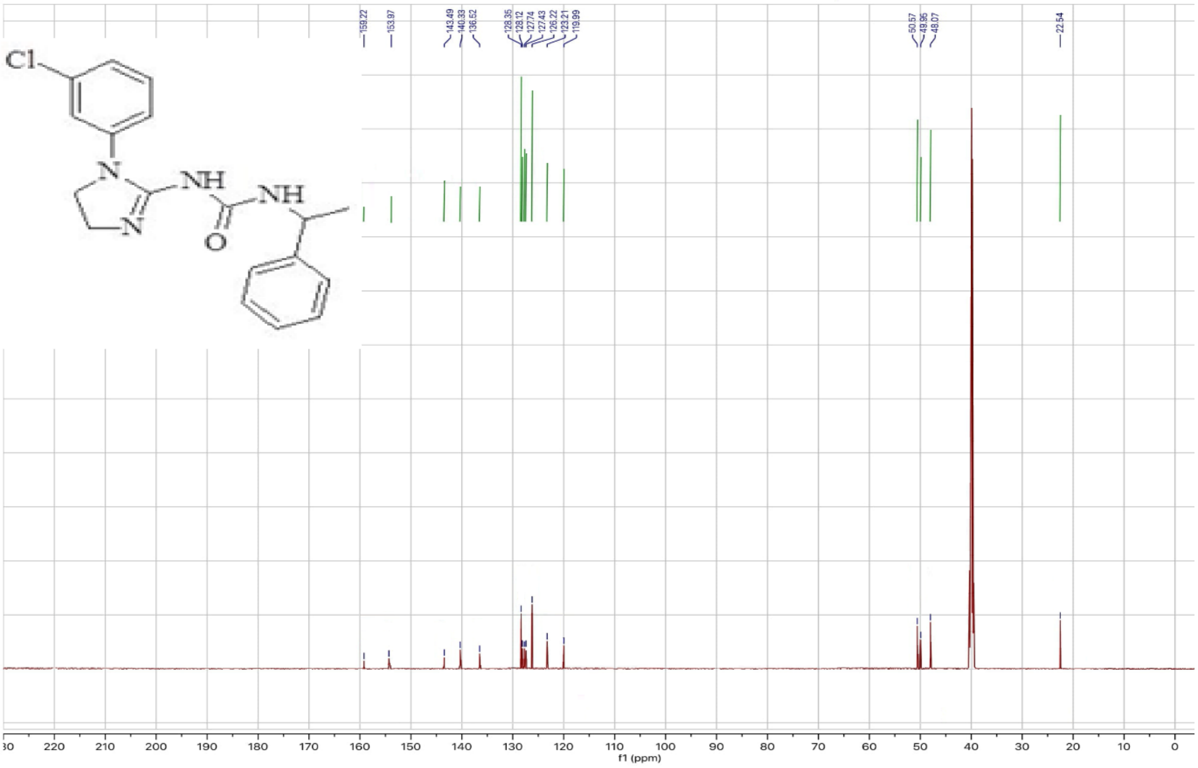

MS spectrum: $C_{18}H_{19}ClN_4O$ (m.m. calc. 343.1320). HRMS (ESI) *m/z* $[M+H]^+$: 343.1365.

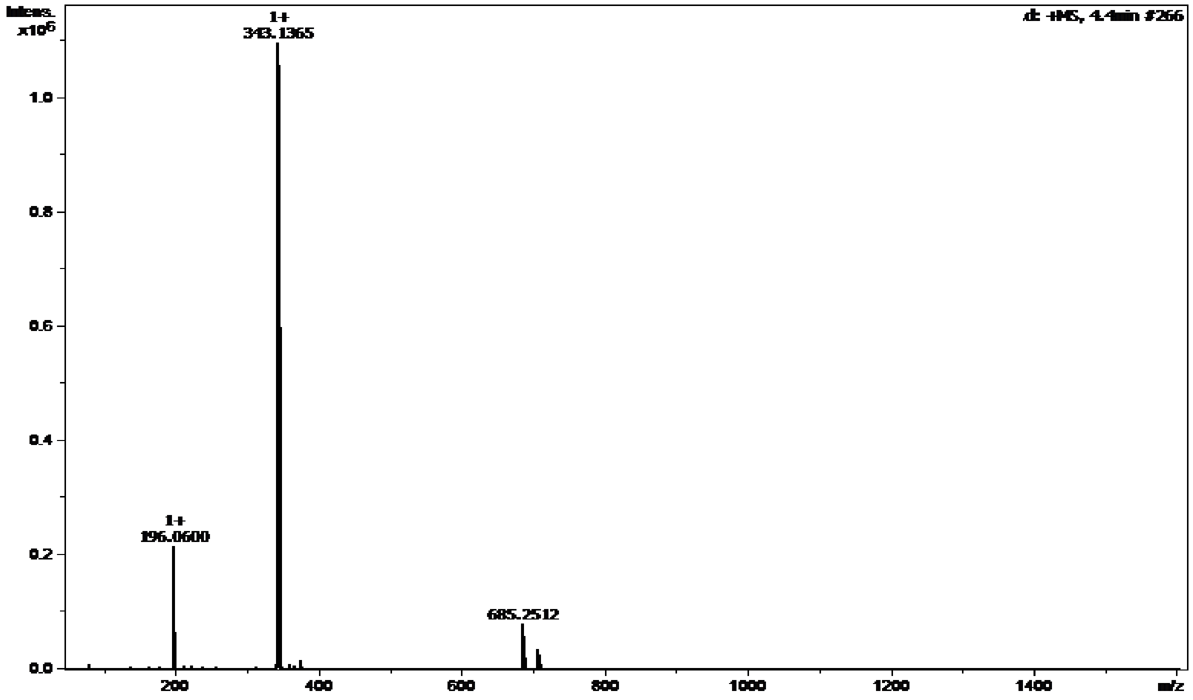

^{1}H NMR spectrum

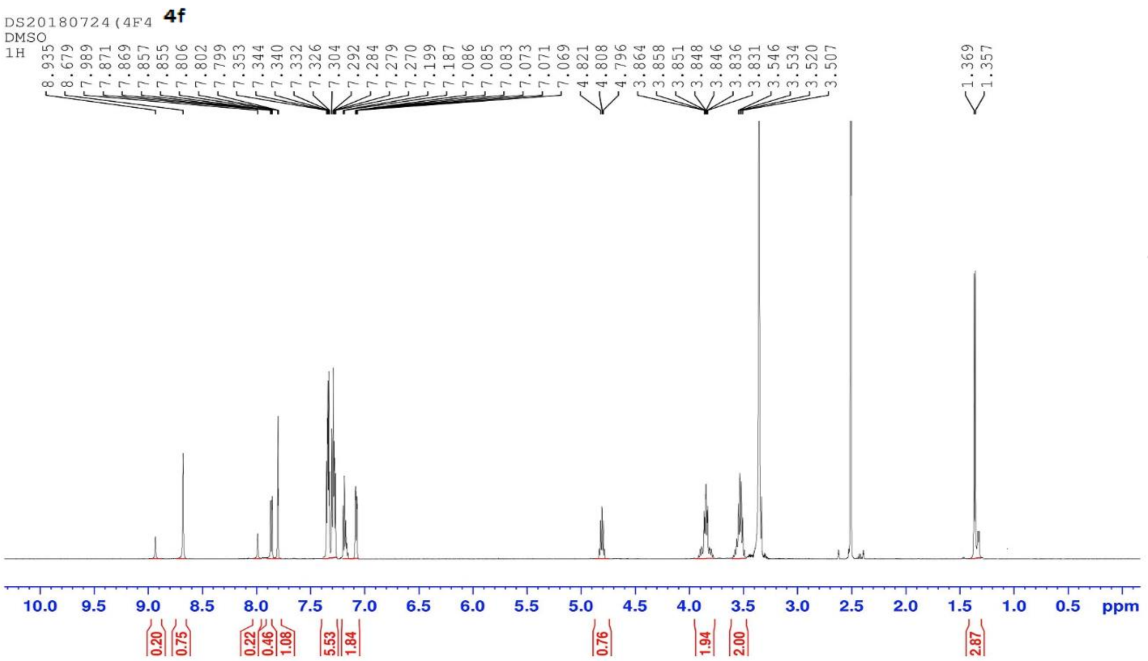

Compound 4g

^{13}C NMR spectrum

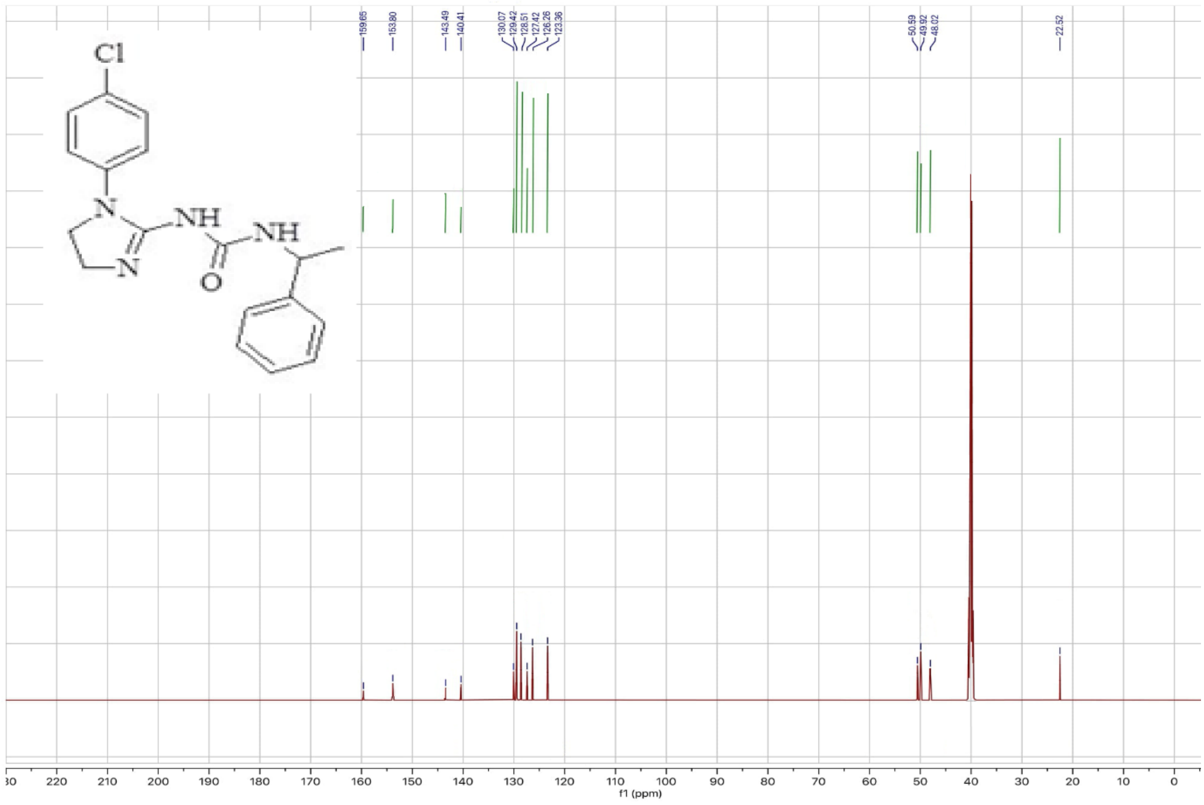

MS spectrum: $C_{18}H_{19}ClN_4O$ (m.m. calc. 343.1320). HRMS (ESI) *m/z* $[M+H]^+$: 343.1291.

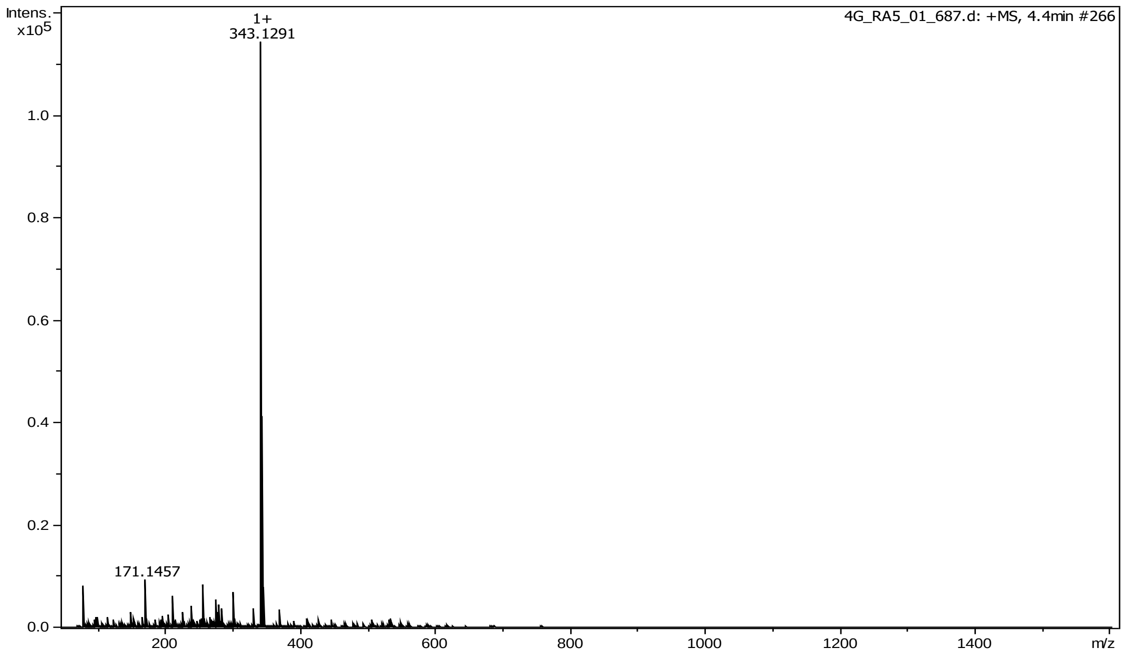

^{1}H NMR spectrum

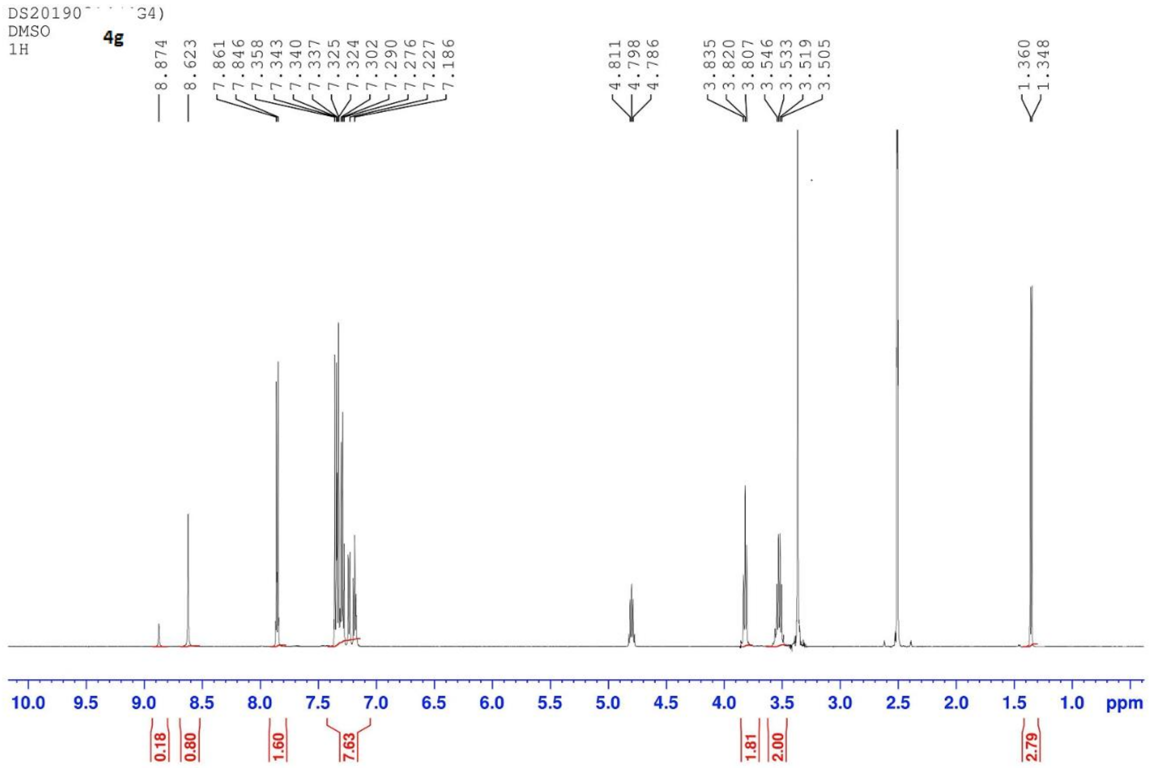

Compound 5a

^{13}C NMR spectrum

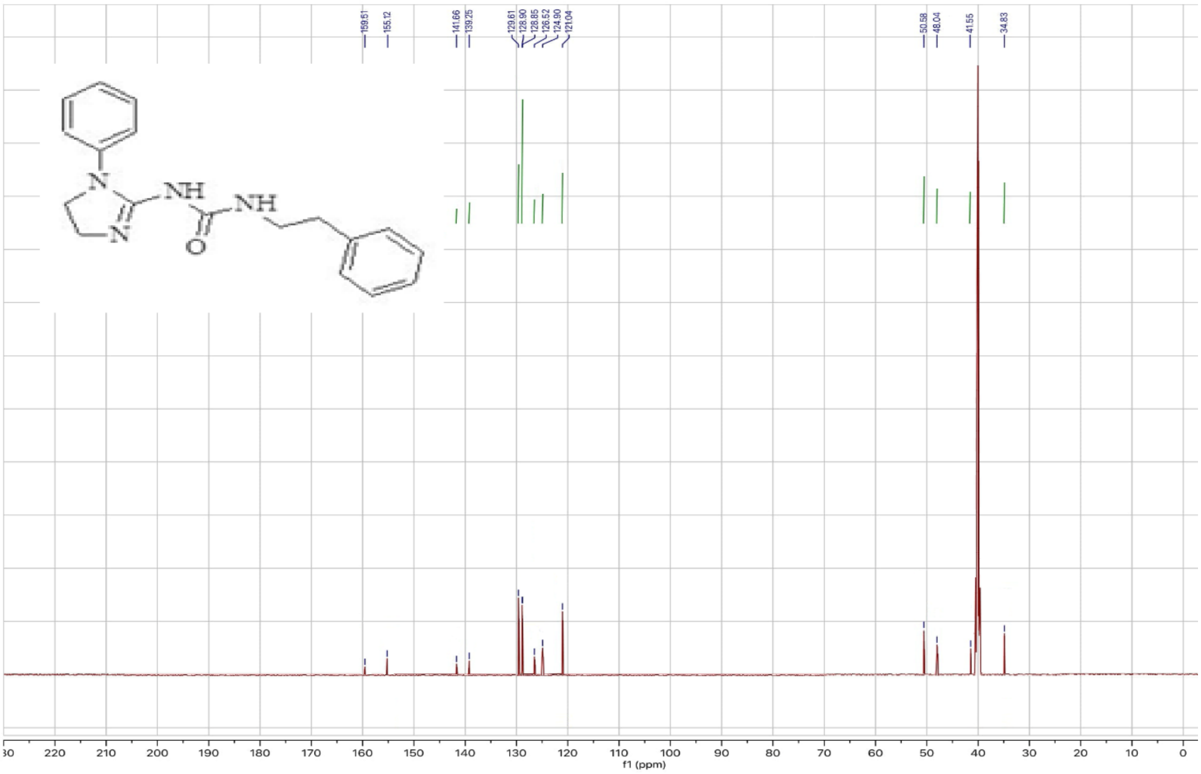

MS spectrum: Formula $C_{18}H_{20}N_4O$ (m.m. calc. 309.1710). HRMS (ESI) *m/z* $[M+H]^+$: 309.1688.

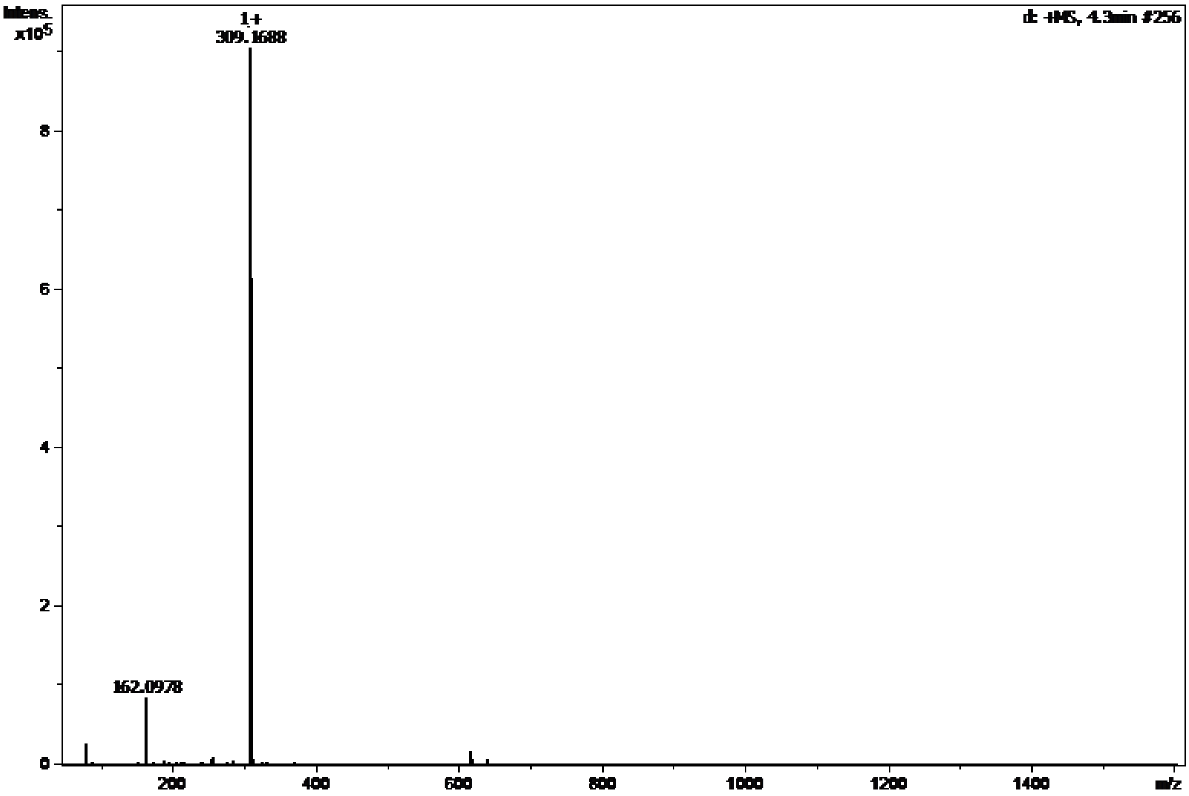

^{1}H NMR spectrum

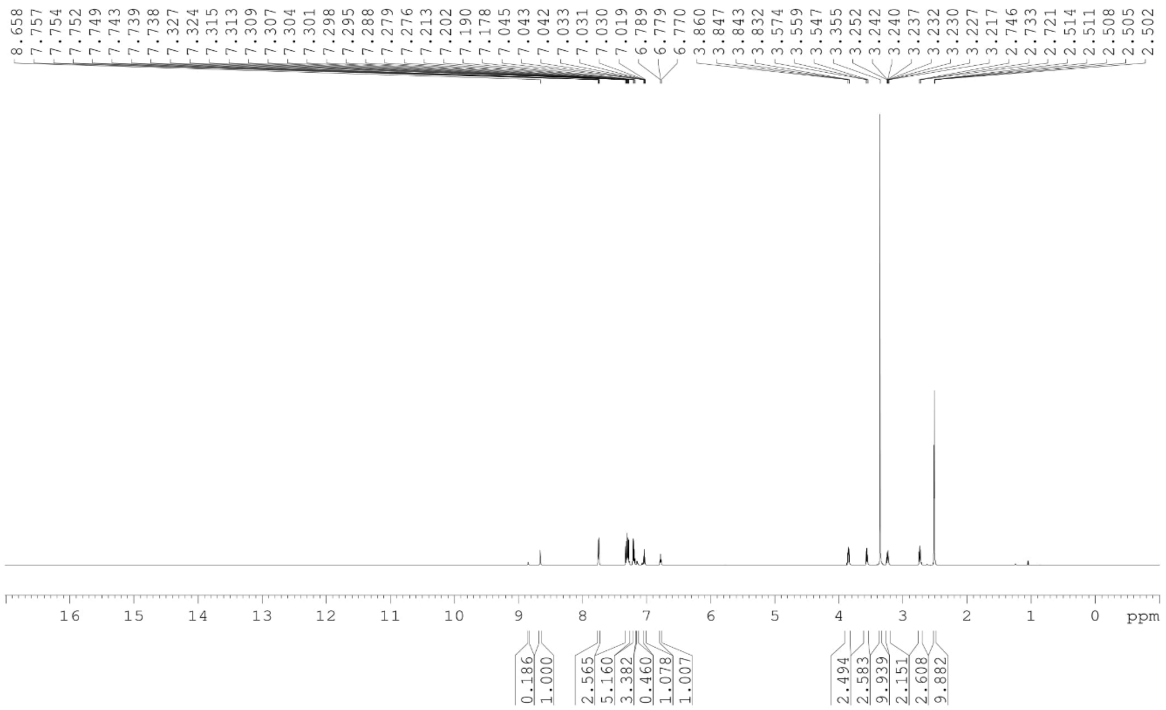

Compound 5b

^{13}C NMR spectrum

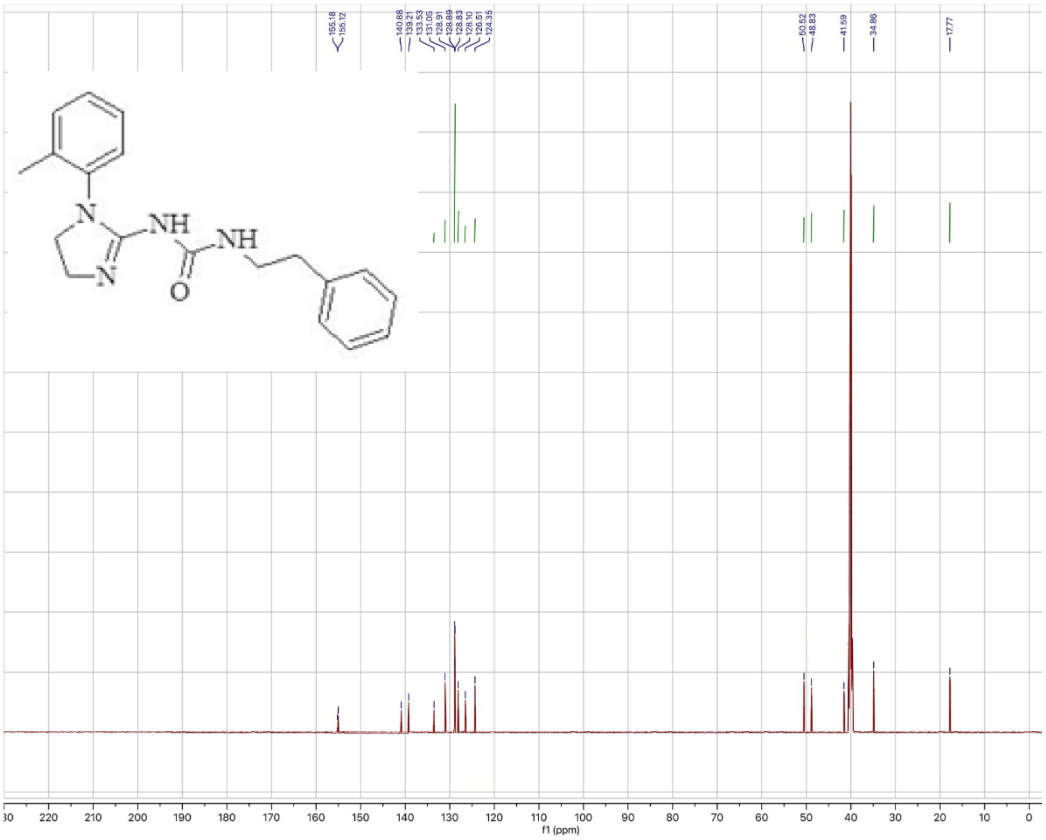

MS spectrum: $C_{19}H_{22}N_4O$ (m.m. calc. 323.1866). HRMS (ESI) *m/z* $[M+H]^+$: 323.2229.

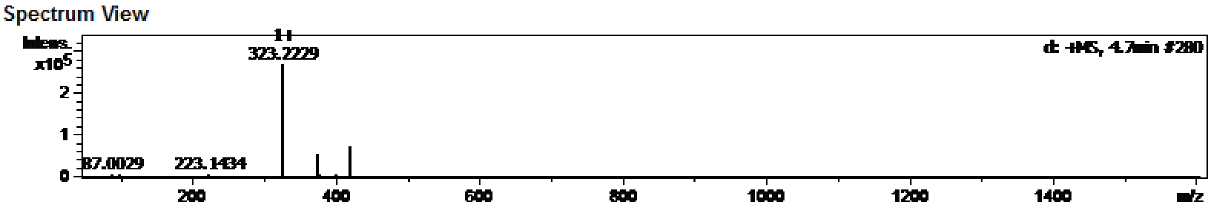

^{1}H NMR spectrum

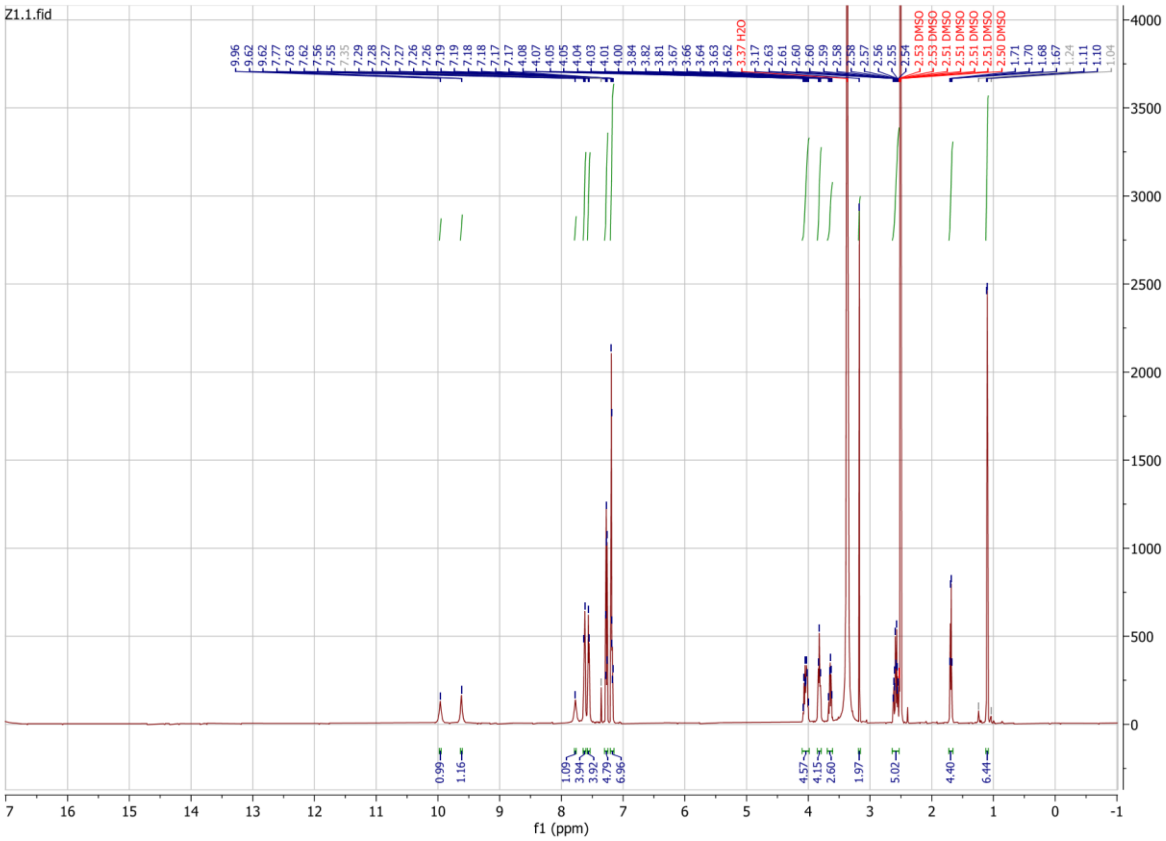

Compound 5c

^{13}C NMR spectrum

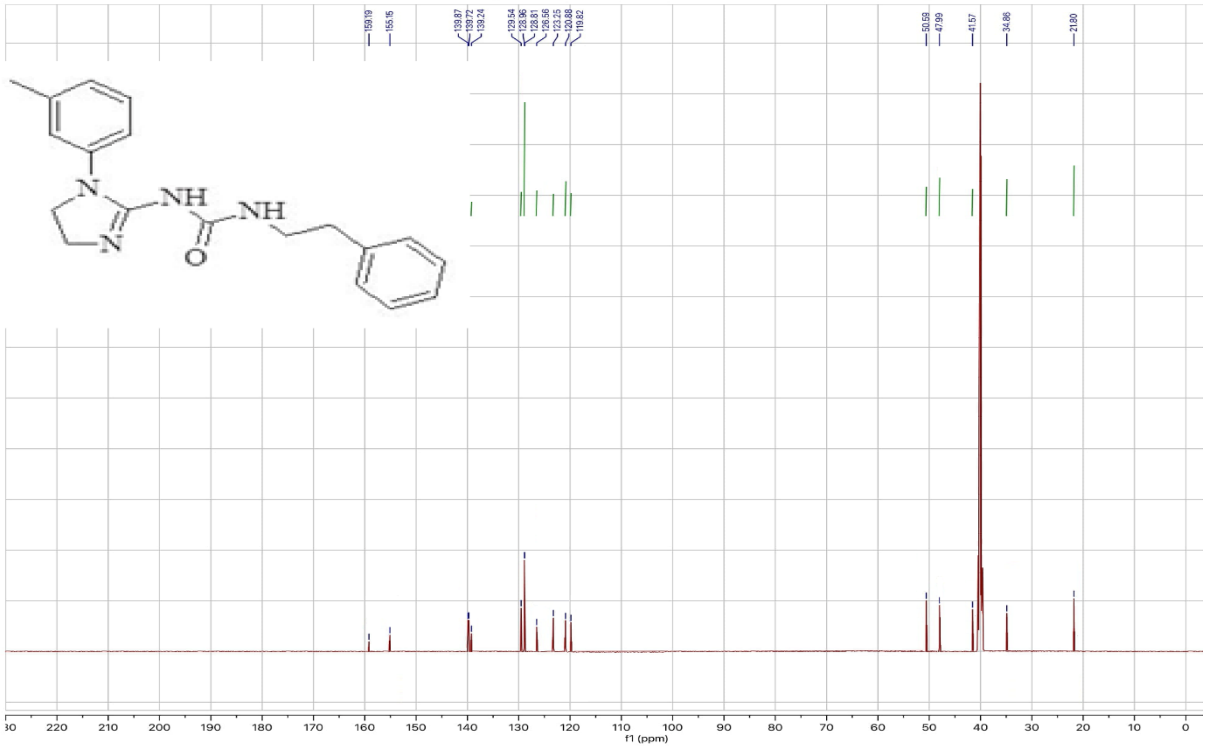

MS spectrum: $C_{19}H_{22}N_4O$ (m.m. calc. 323.1866). HRMS (ESI) *m/z* $[M+H]^+$: 323.1535.

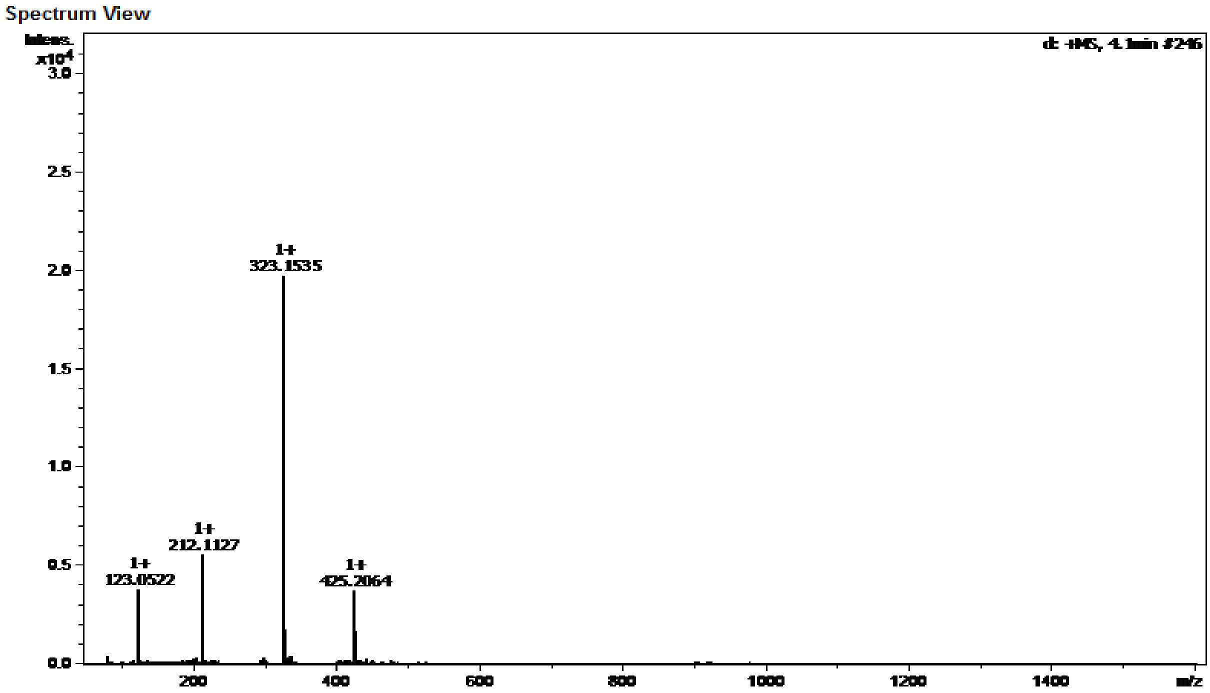

^{1}H NMR spectrum

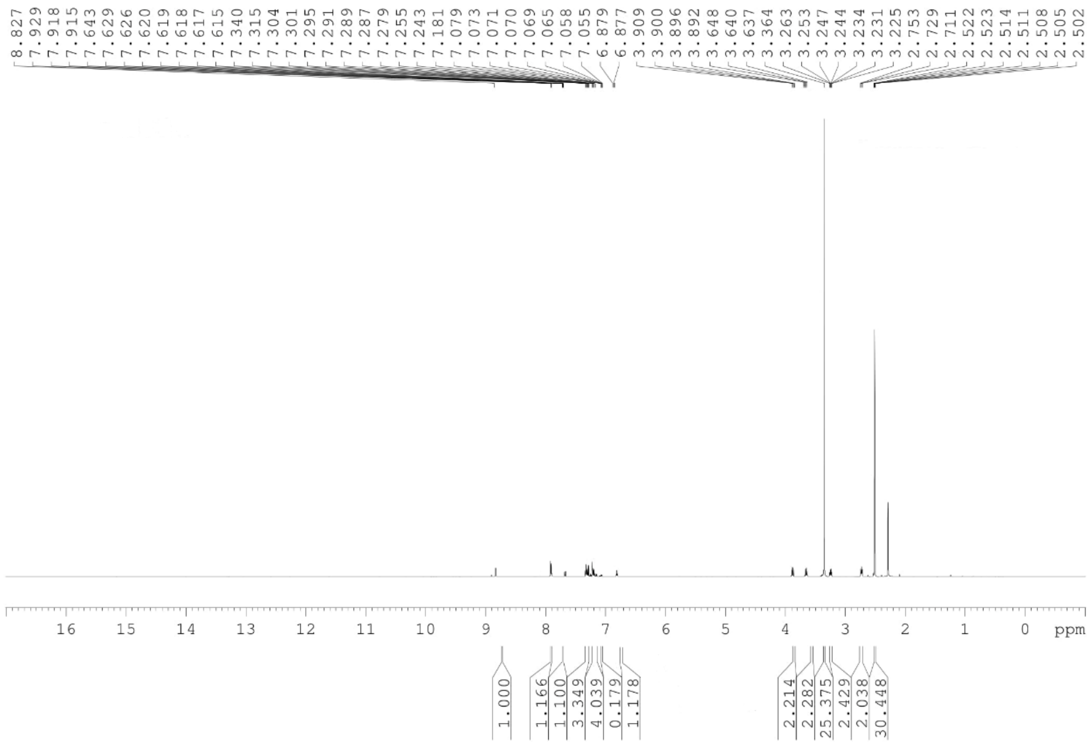

Compound 5d

^{13}C NMR spectrum

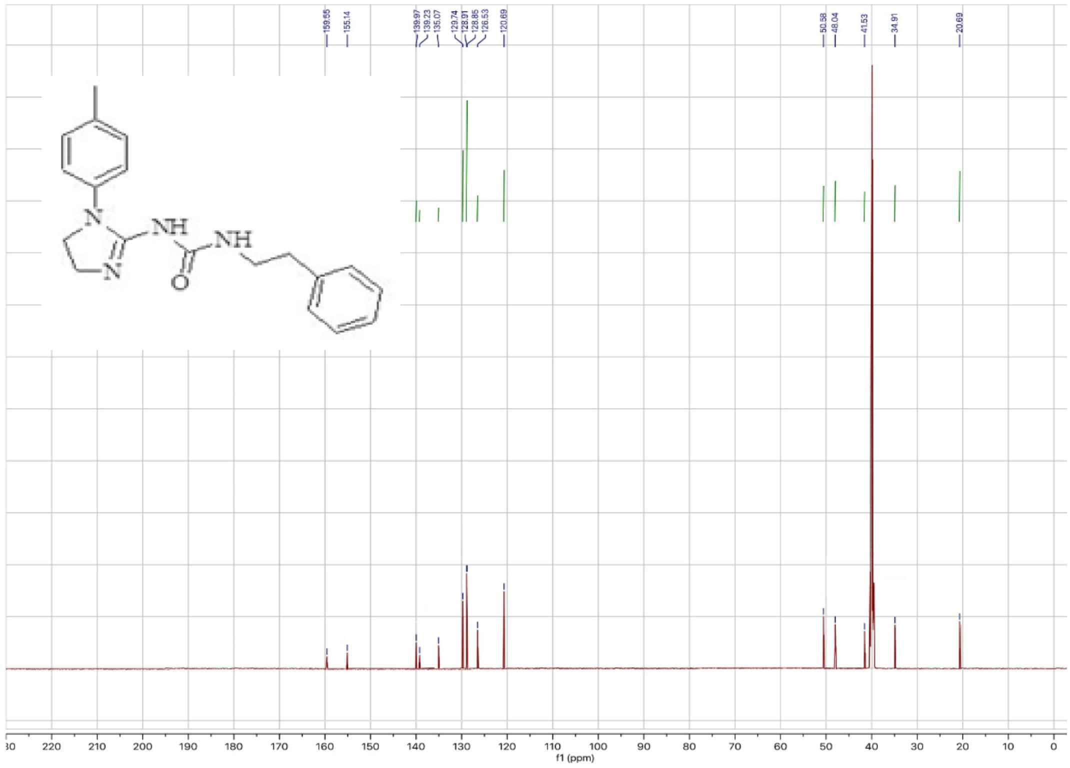

MS spectrum: $C_{19}H_{22}N_4O$ (m.m. calc. 323.1866). HRMS (ESI) *m/z* $[M+H]^+$ 323.1534.

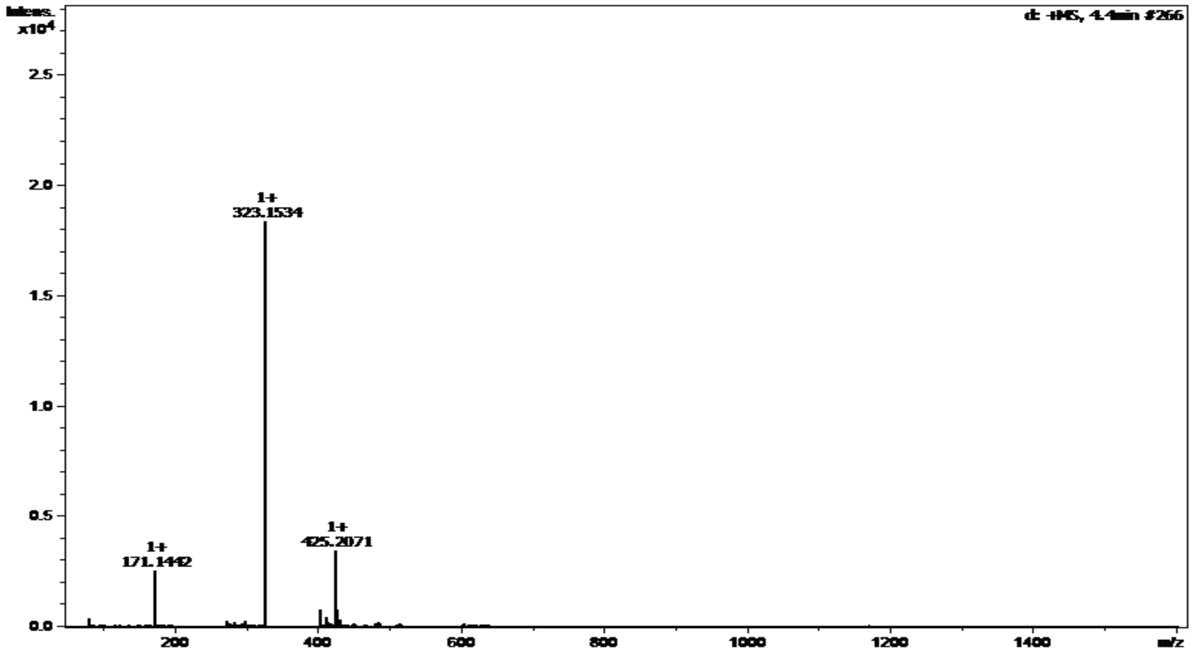

^{1}H NMR spectrum

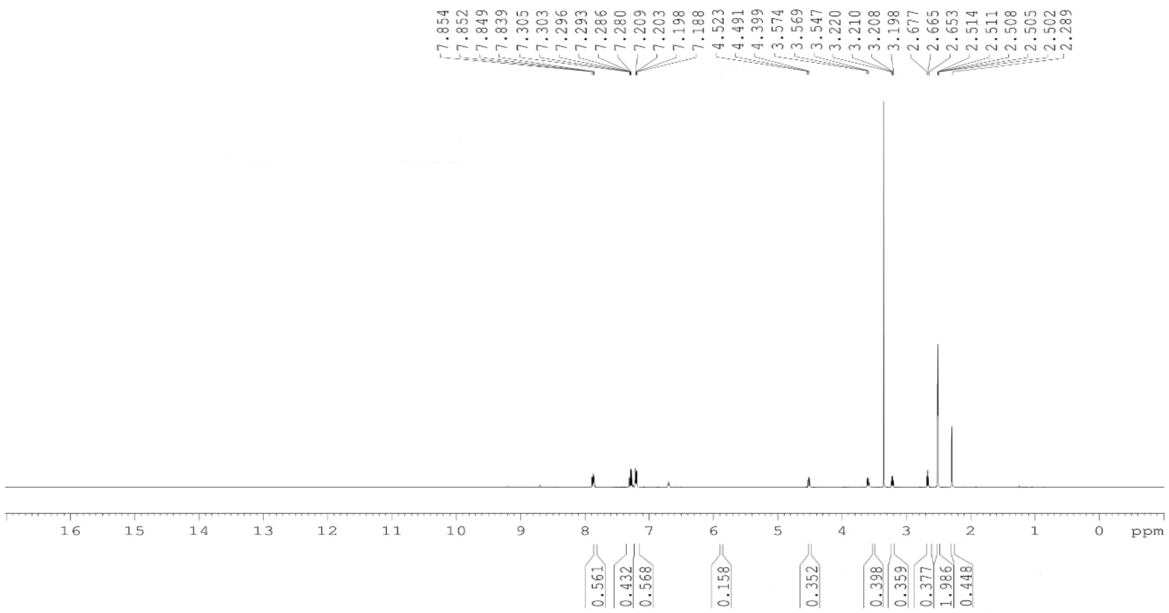

Compound 5e

^{13}C NMR spectrum

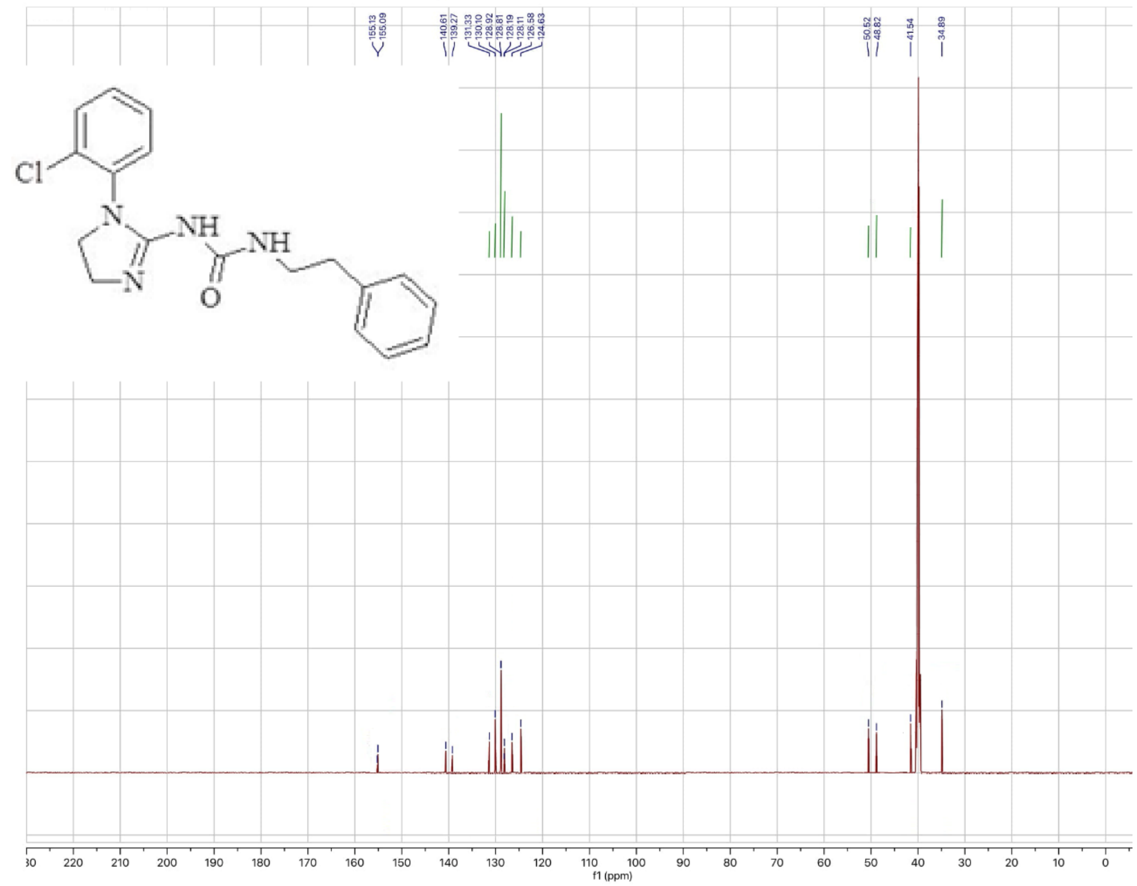

MS spectrum: $C_{18}H_{19}ClN_4O$ (m.m. calc. 343.1320). HRMS (ESI) *m/z* [M+H]$^+$: 343.1271.

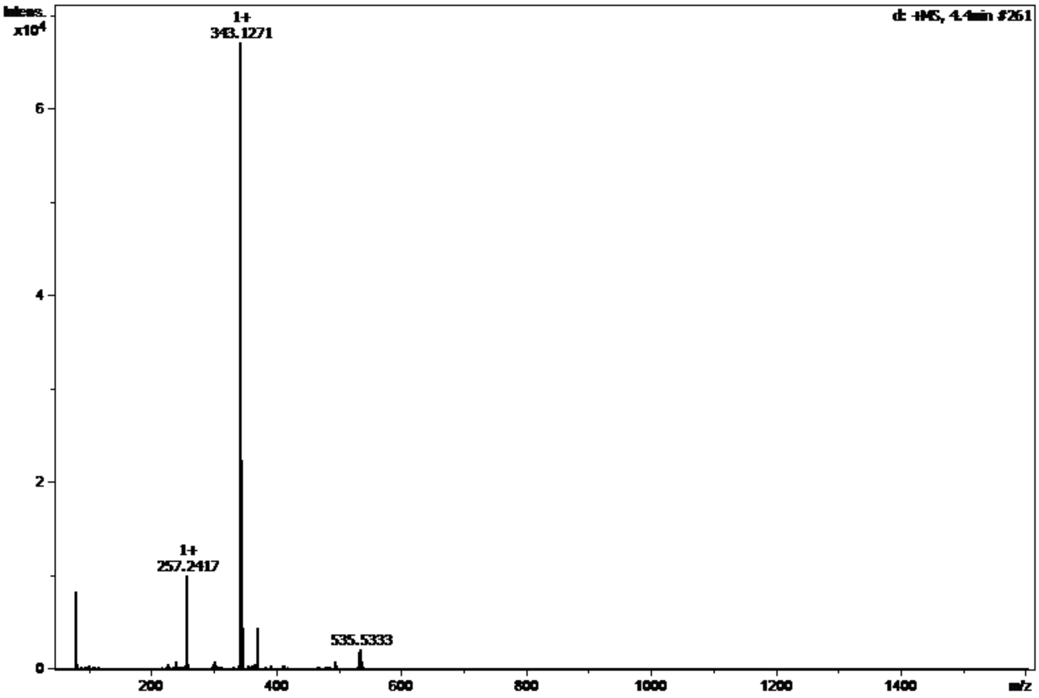

^{1}H NMR spectrum

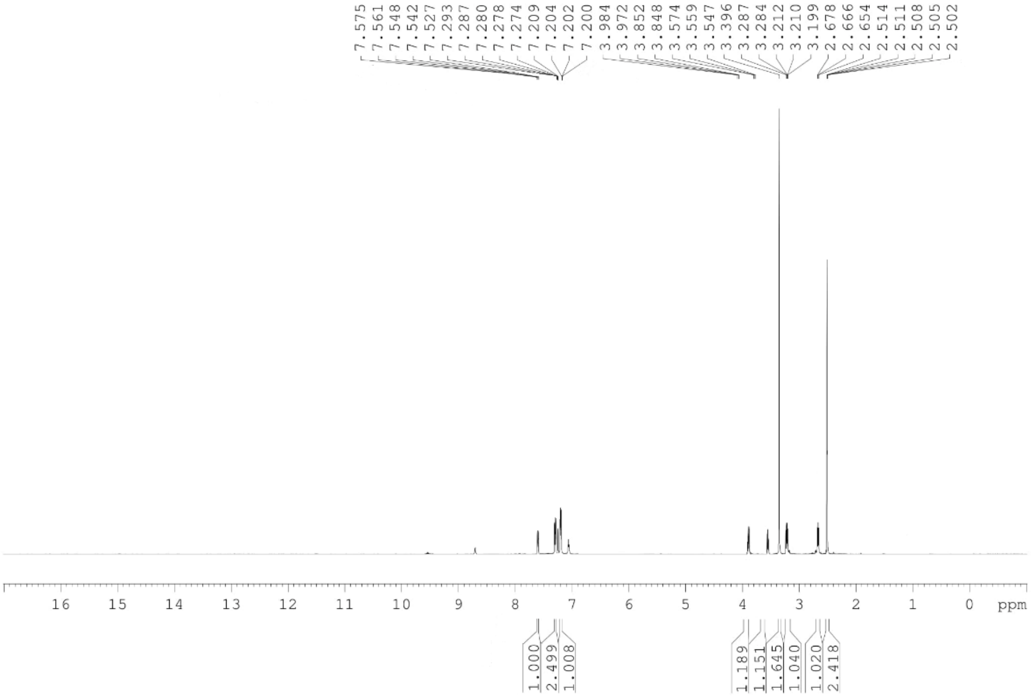

Compound 5f

^{13}C NMR spectrum

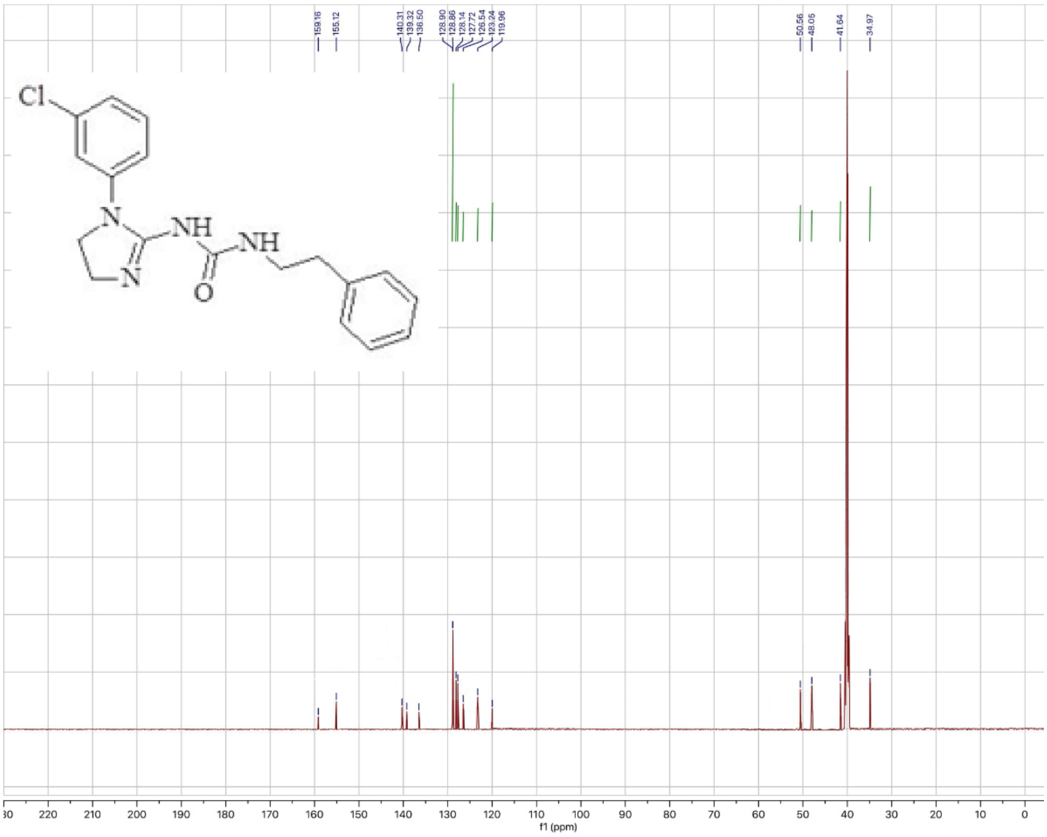

MS spectrum: $C_{18}H_{19}ClN_4O$ (m.m. calc. 343.1320). HRMS (ESI) *m/z* $[M+H]^+$: 343.1297.

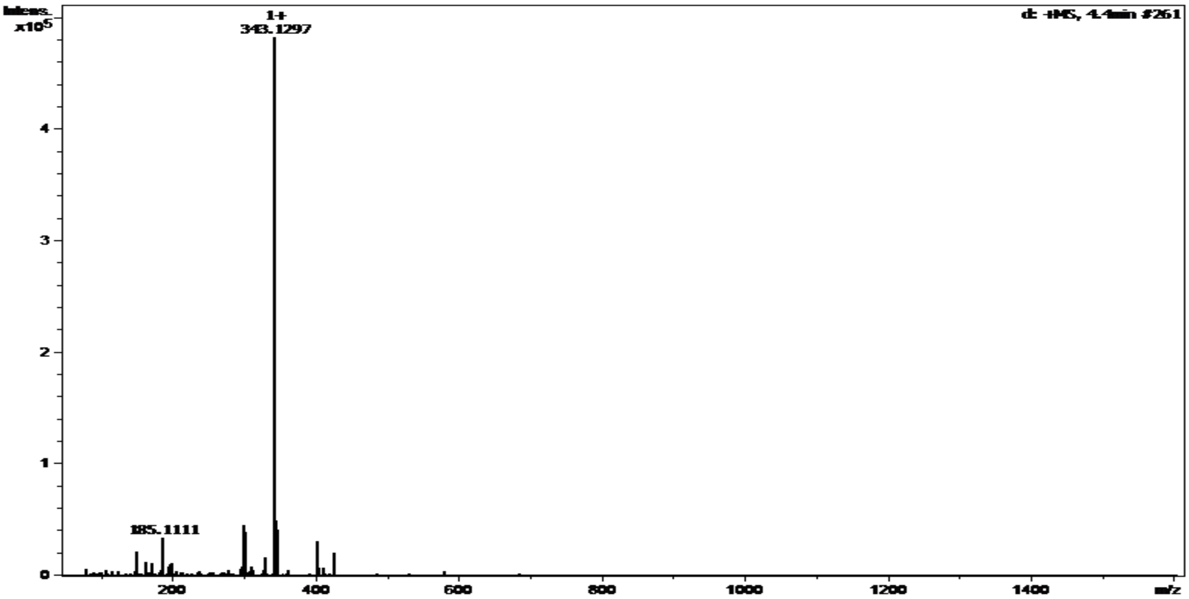

^{1}H NMR spectrum

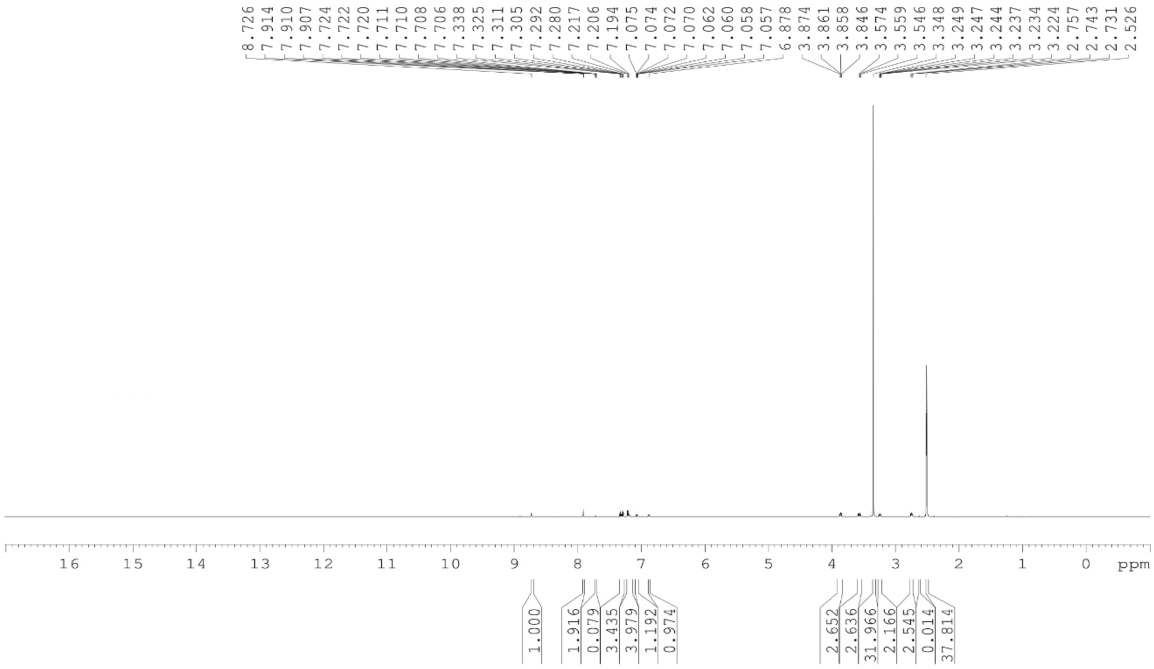

Compound 5g

^{13}C NMR spectrum

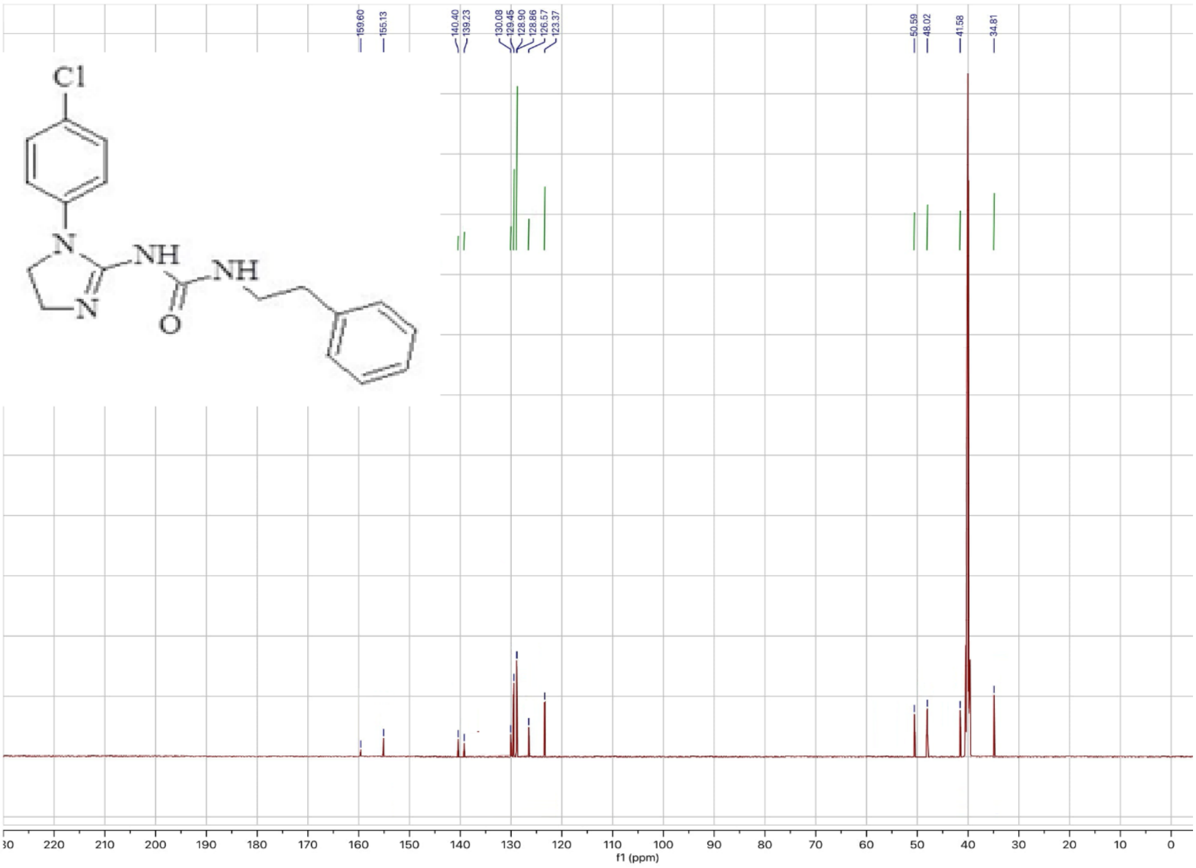

MS spectrum: $C_{18}H_{19}ClN_4O$ (m.m. calc. 343.1320). HRMS (ESI) *m/z* $[M+H]^+$: 343.1316.

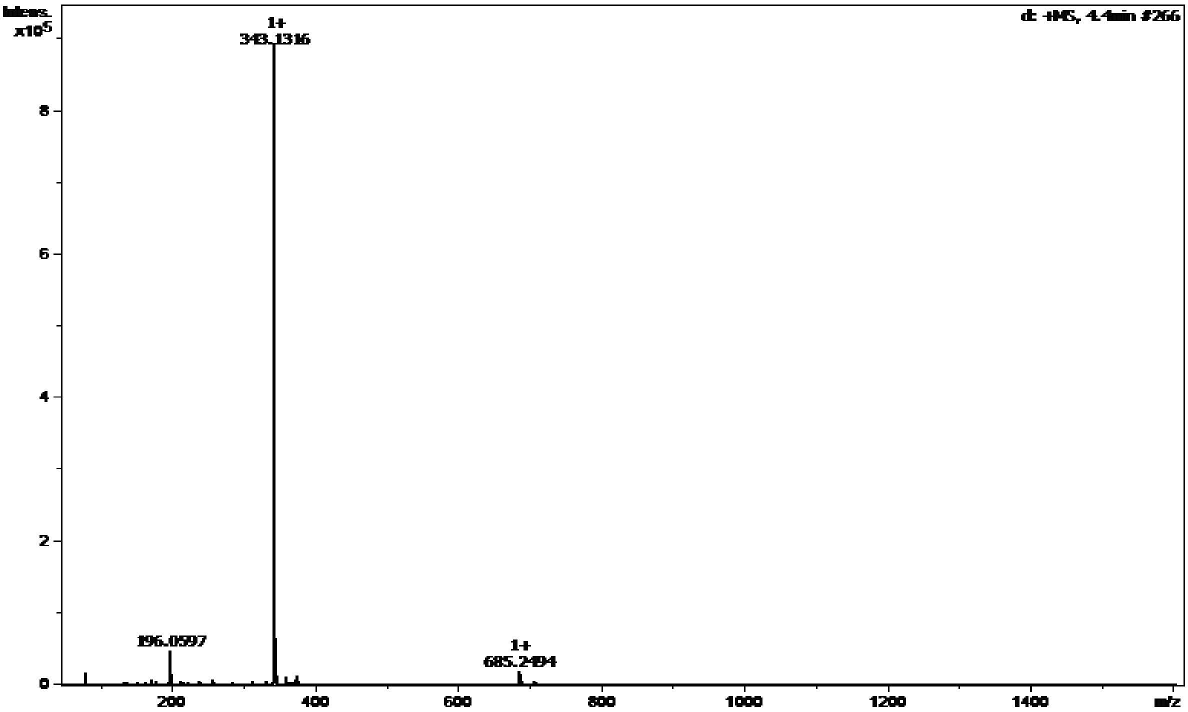

^{1}H NMR spectrum

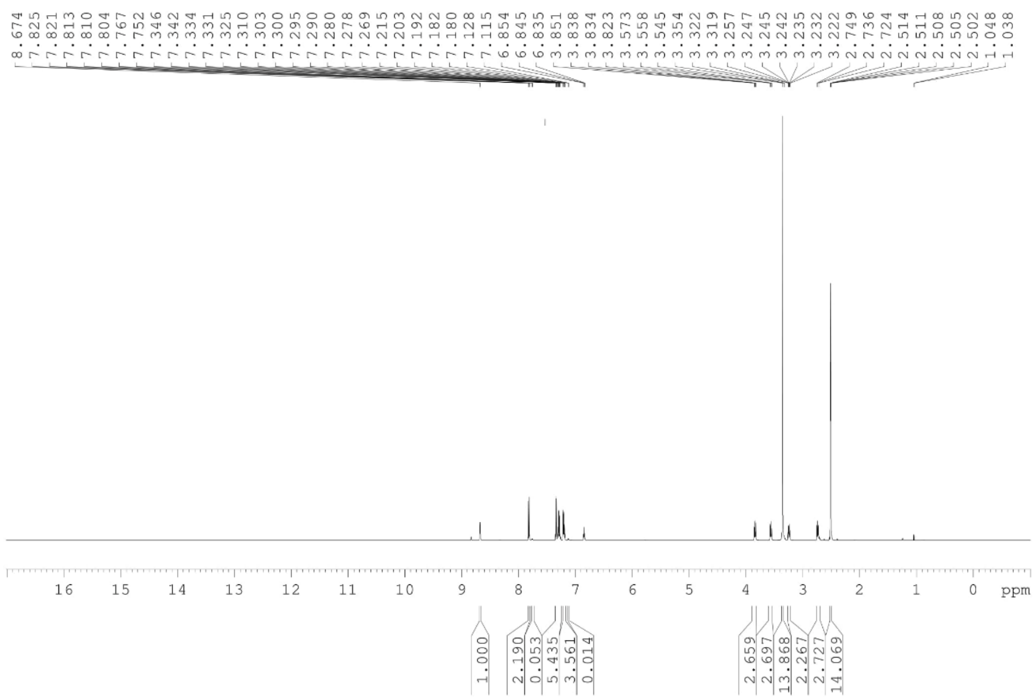

Compound 6a

^{13}C NMR spectrum

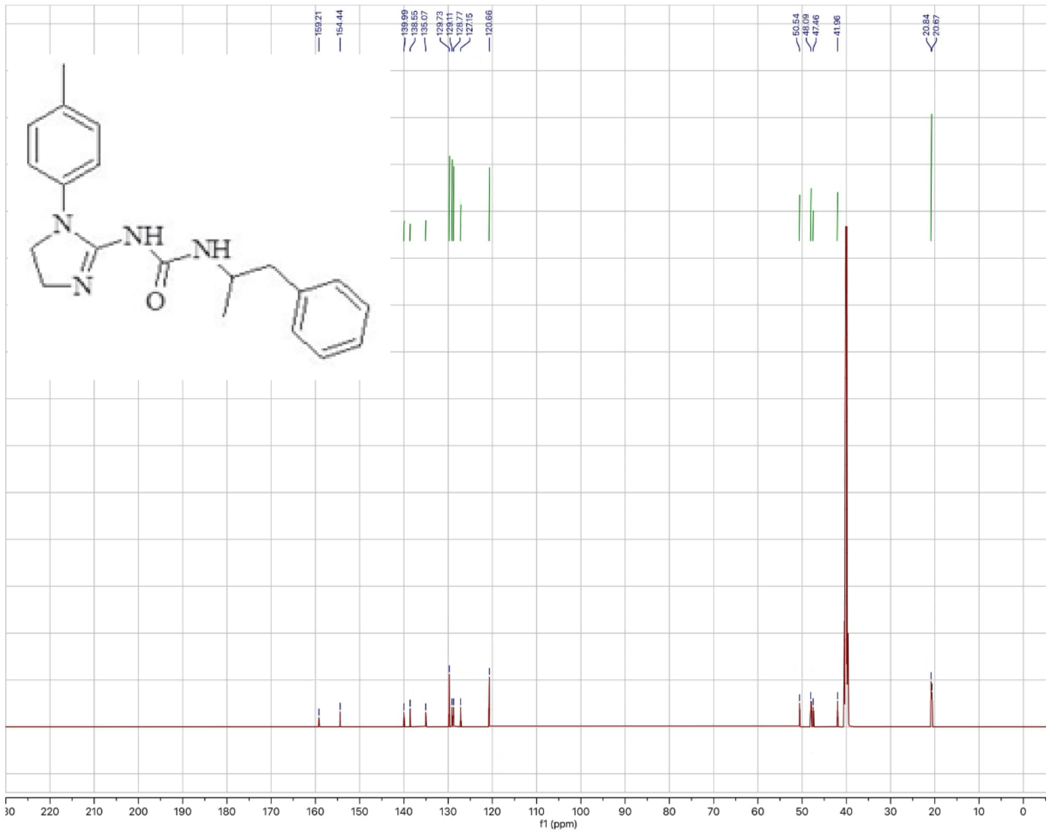

MS spectrum: $C_{20}H_{24}N_4O$ (m.m. calc. 337.2023). HRMS (ESI) *m/z* $[M+H]^+$: 337.1959.

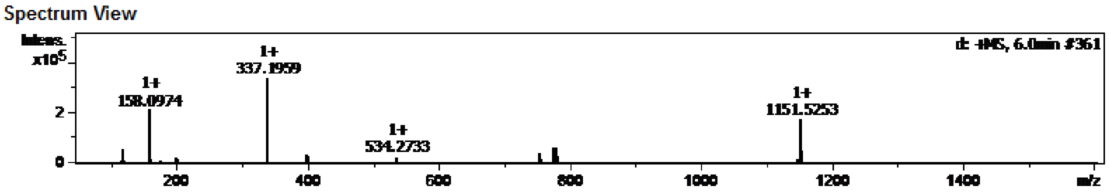

¹H NMR spectrum

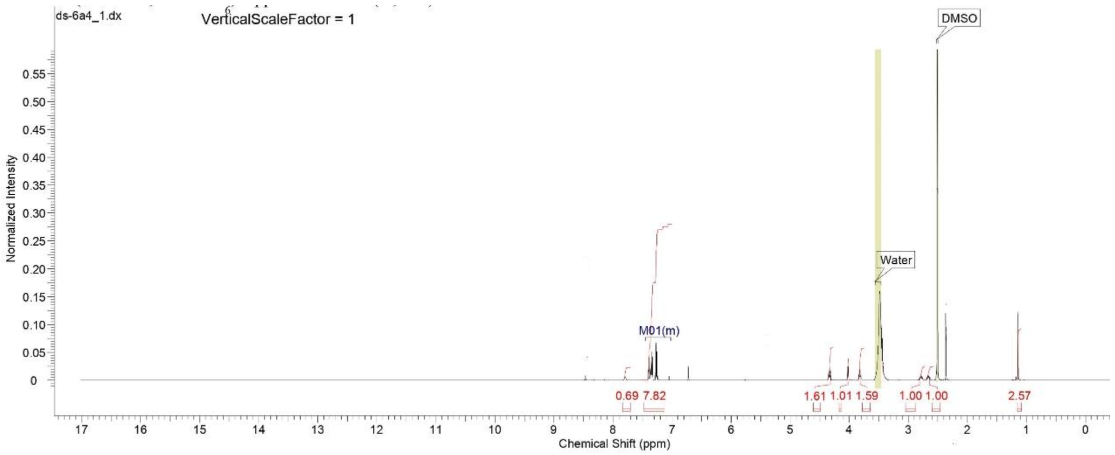

Compound 6b

¹³C NMR spectrum

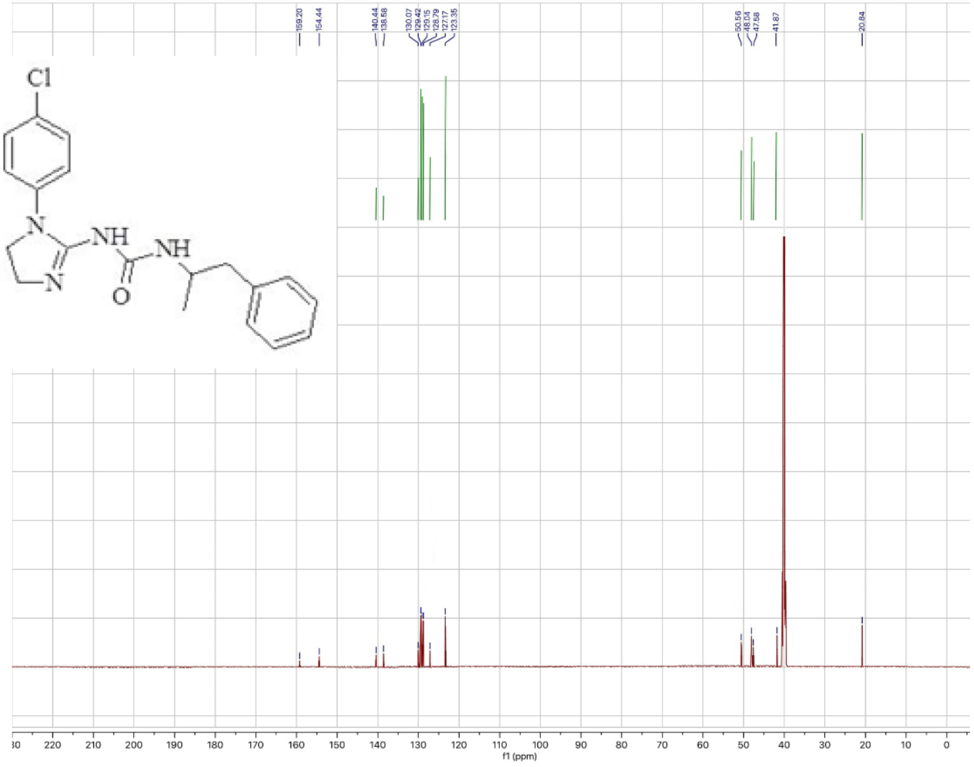

MS spectrum: $C_{19}H_{21}ClN_4O$ (m.m. calc. 357.1477). HRMS (ESI) *m/z* $[M+H]^+$: 357.1048.

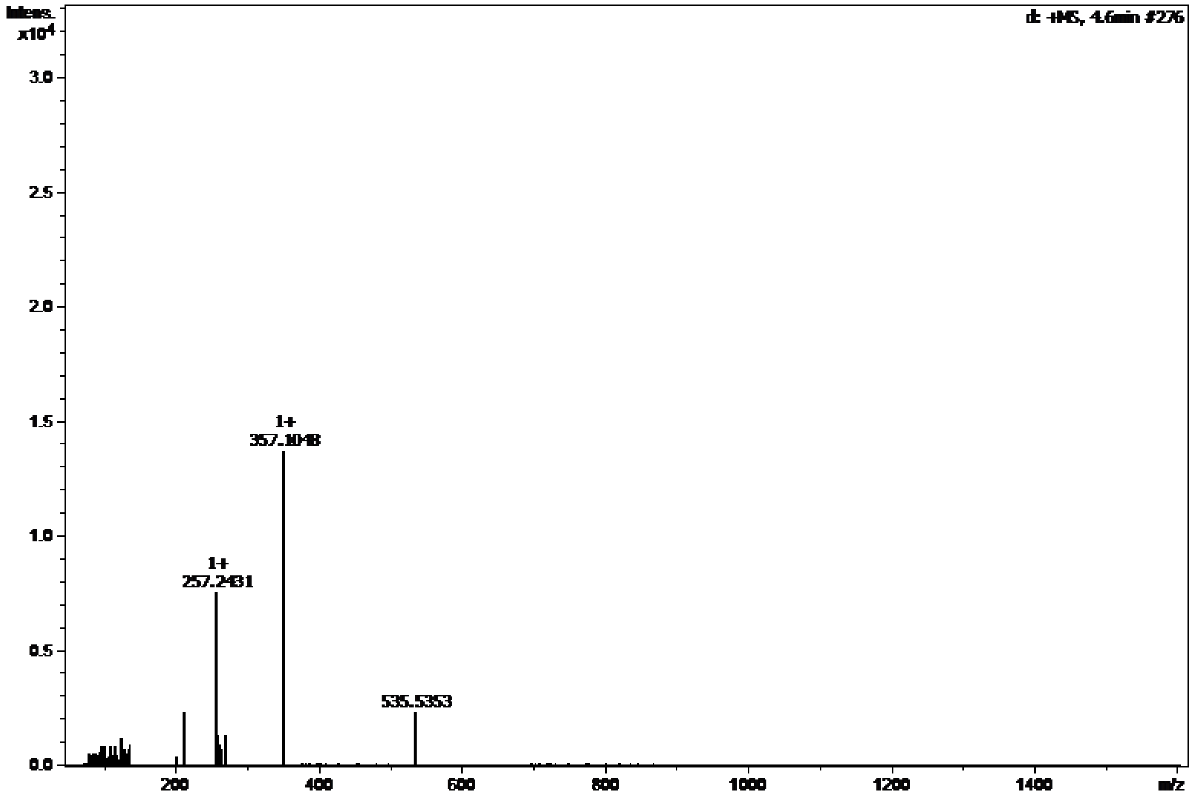

^{1}H NMR spectrum

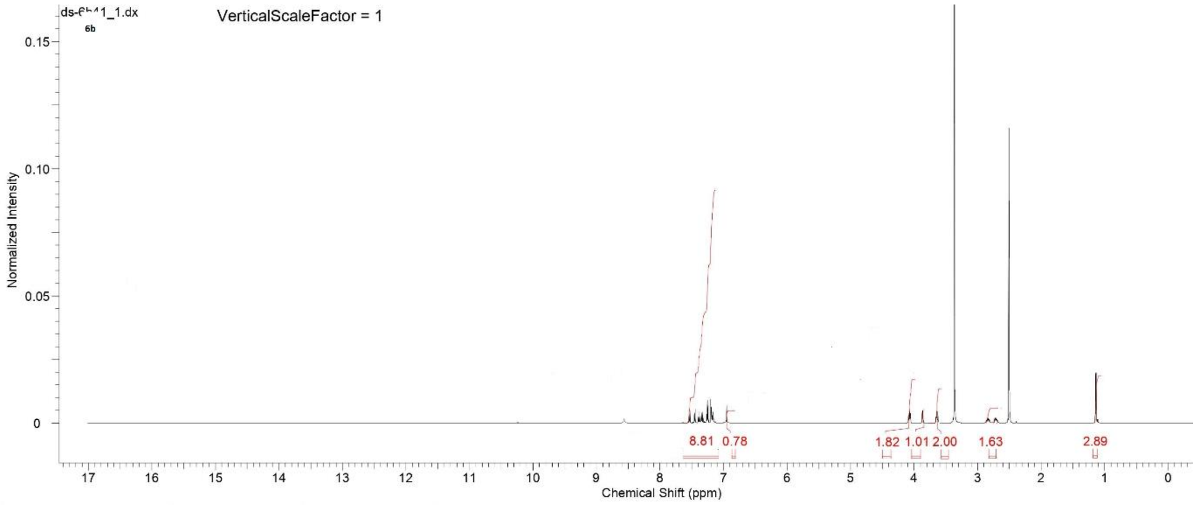

Compound 6c

^{13}C NMR spectrum

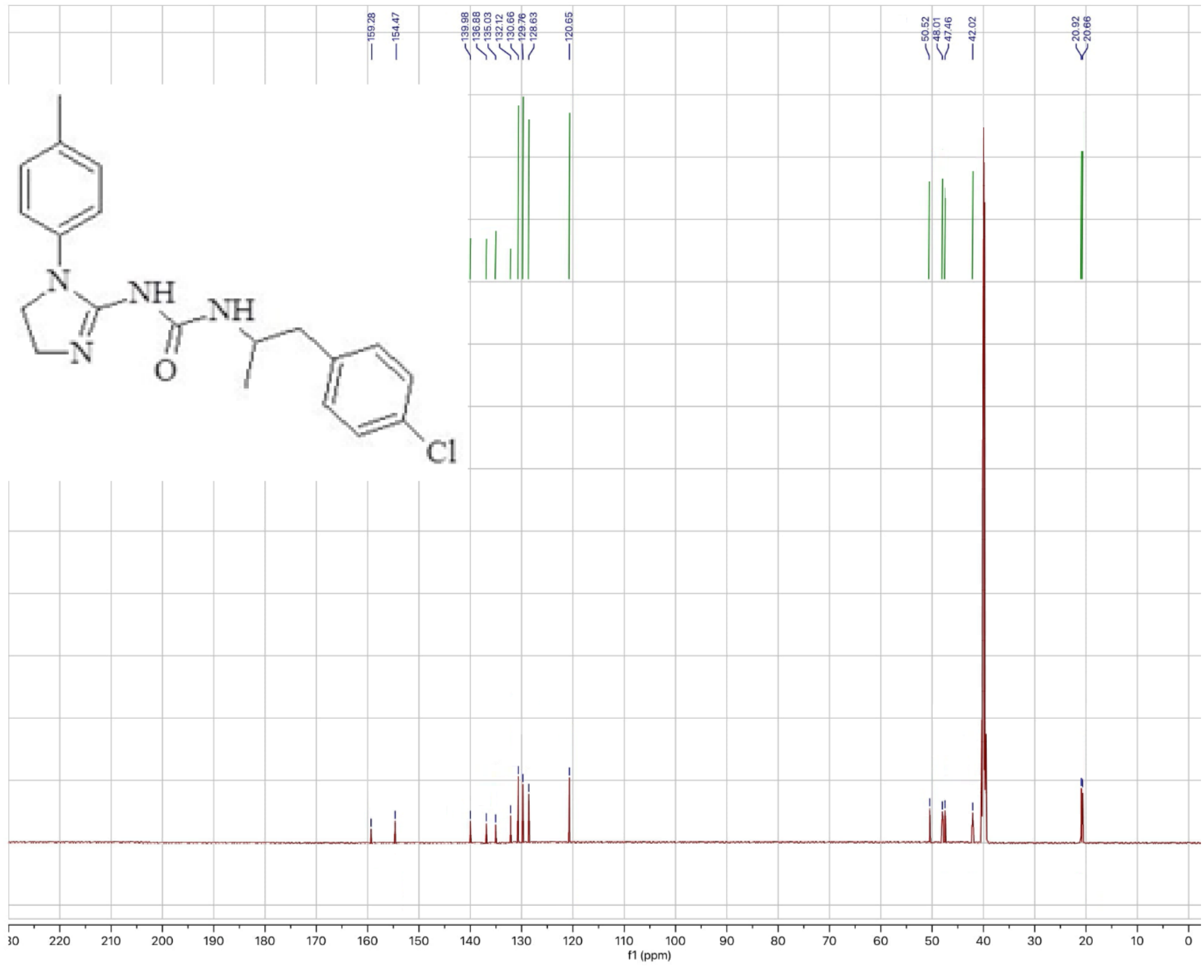

MS spectrum: $C_{20}H_{23}ClN_4O$ (m.m. calc. 371.1633). HRMS (ESI) *m/z* $[M+H]^+$: 371.1621.

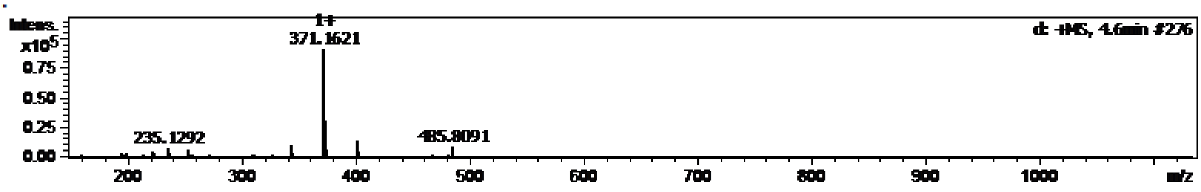

^{1}H NMR spectrum

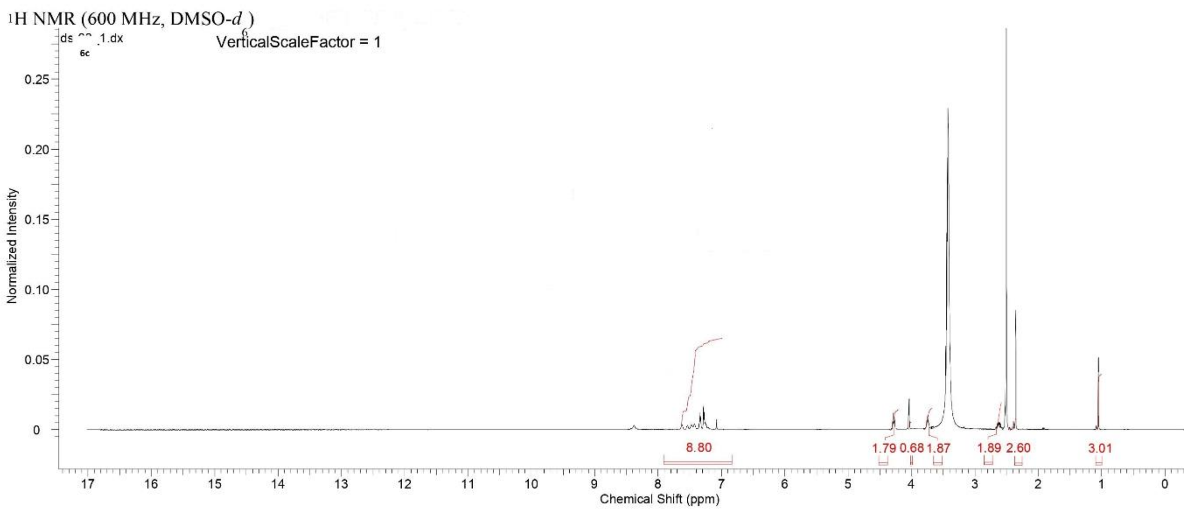

Compound 6d

^{13}C NMR spectrum

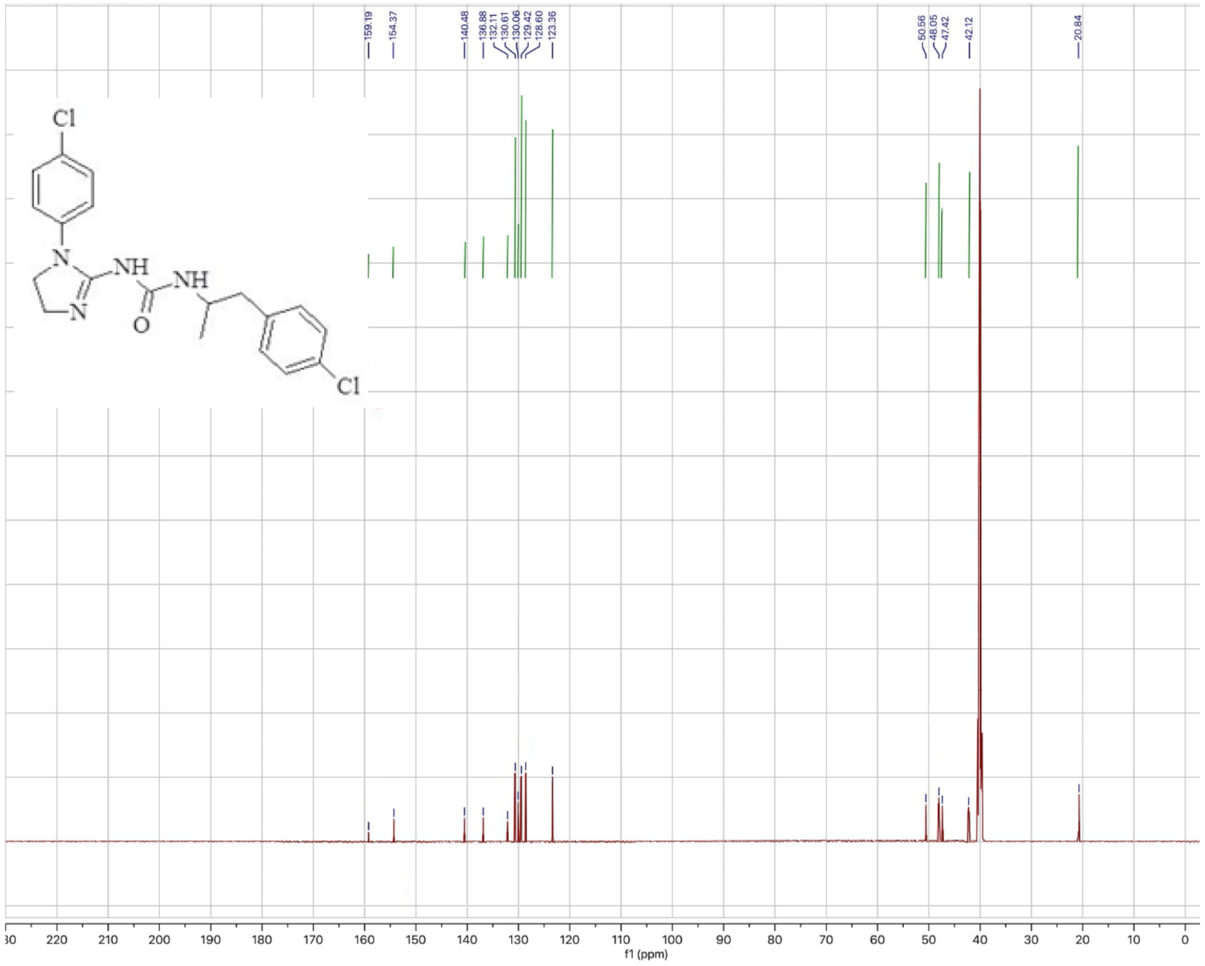

MS spectrum: $C_{19}H_{20}Cl_2N_4O$ (m.m. calc. 391.1087). HRMS (ESI) *m/z* $[M+H]^+$: 391.1052.

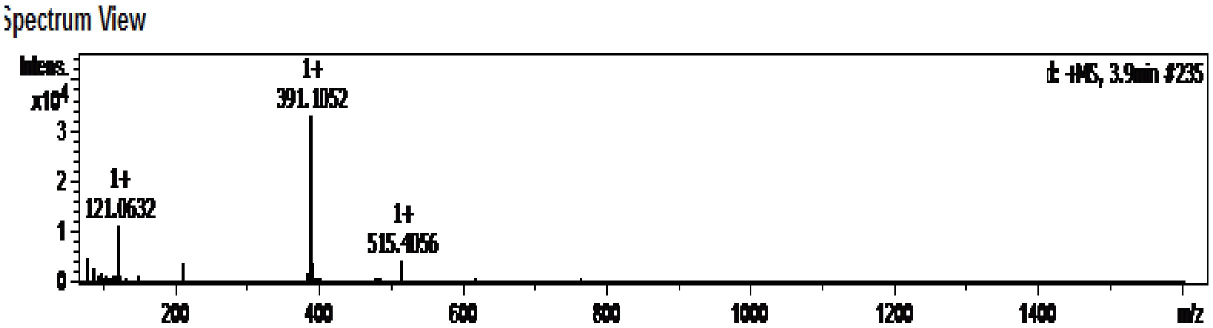

^{1}H NMR spectrum

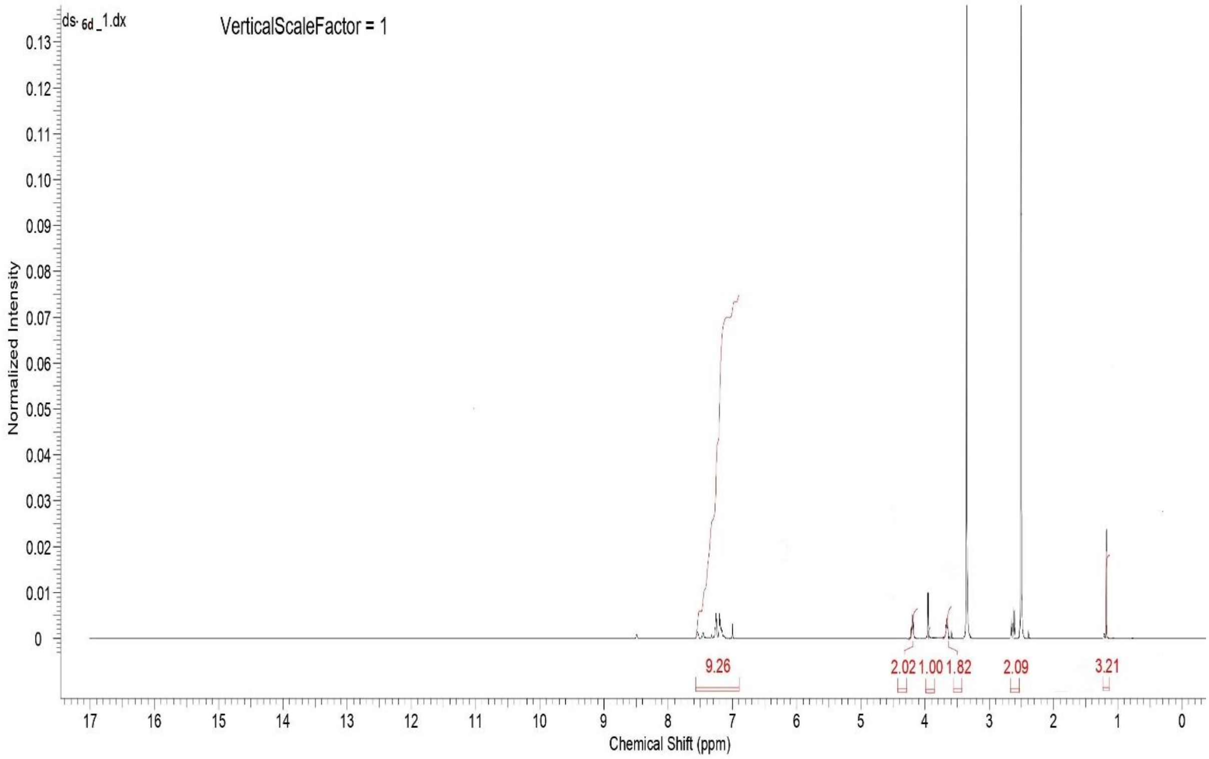

Compound 6e

^{13}C NMR spectrum

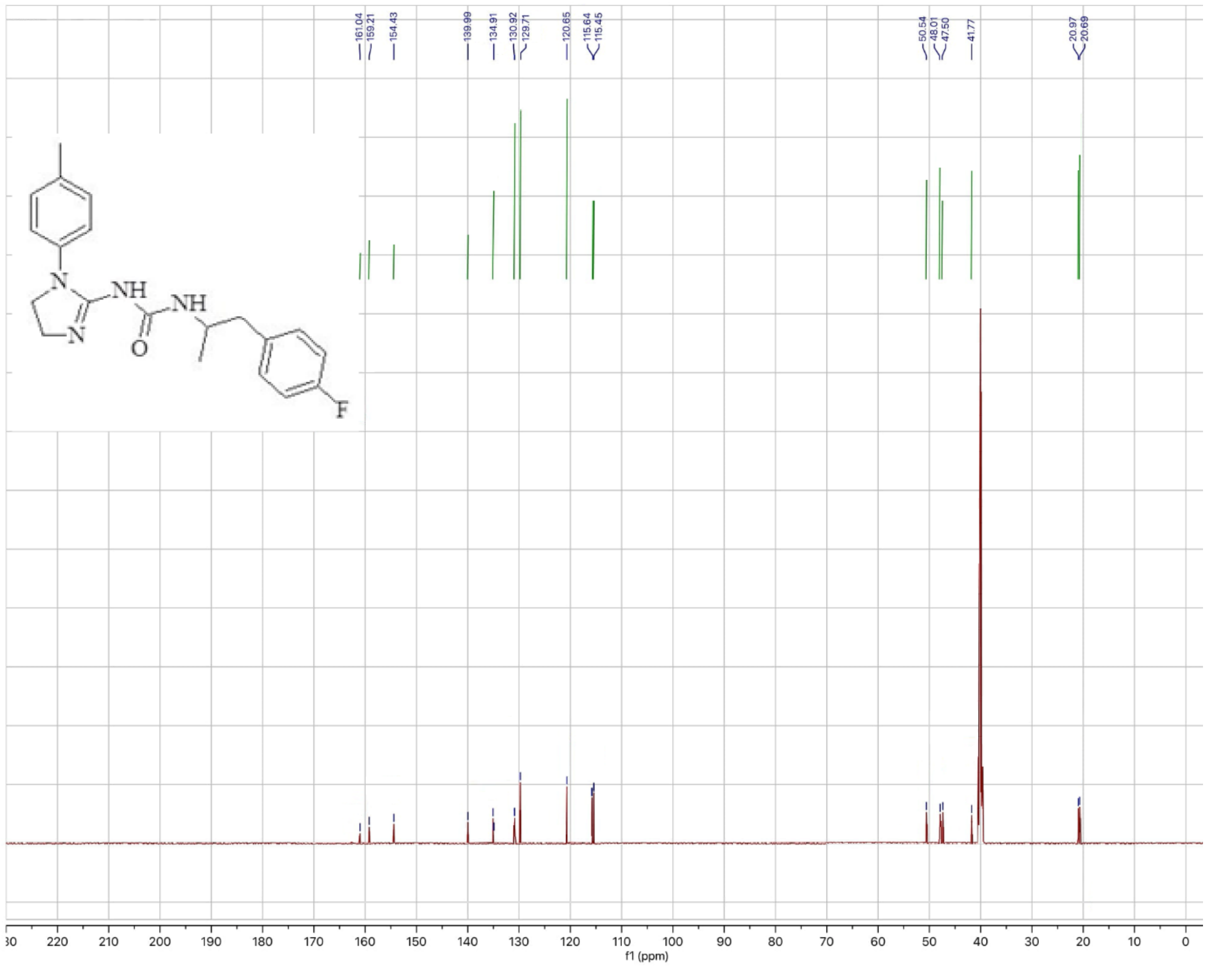

MS spectrum: $C_{20}H_{23}FN_4O$ (m.m. calc. 355.1929). HRMS (ESI) *m/z* [M+H]$^+$: 355.1891.

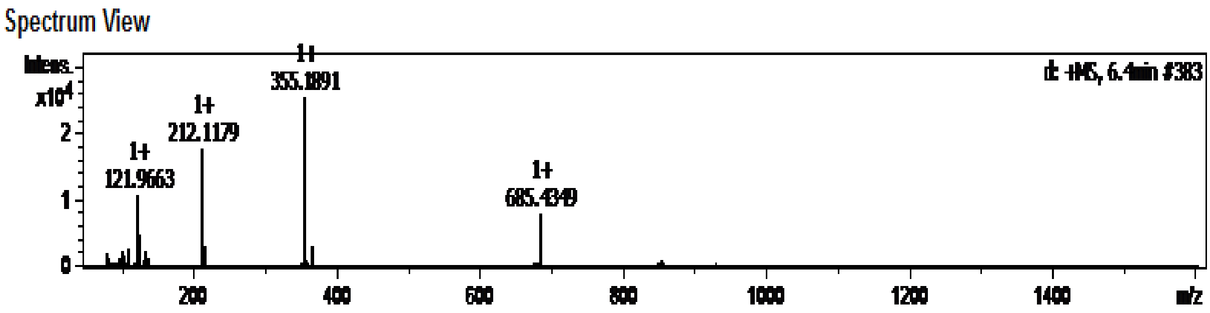

^{1}H NMR spectrum

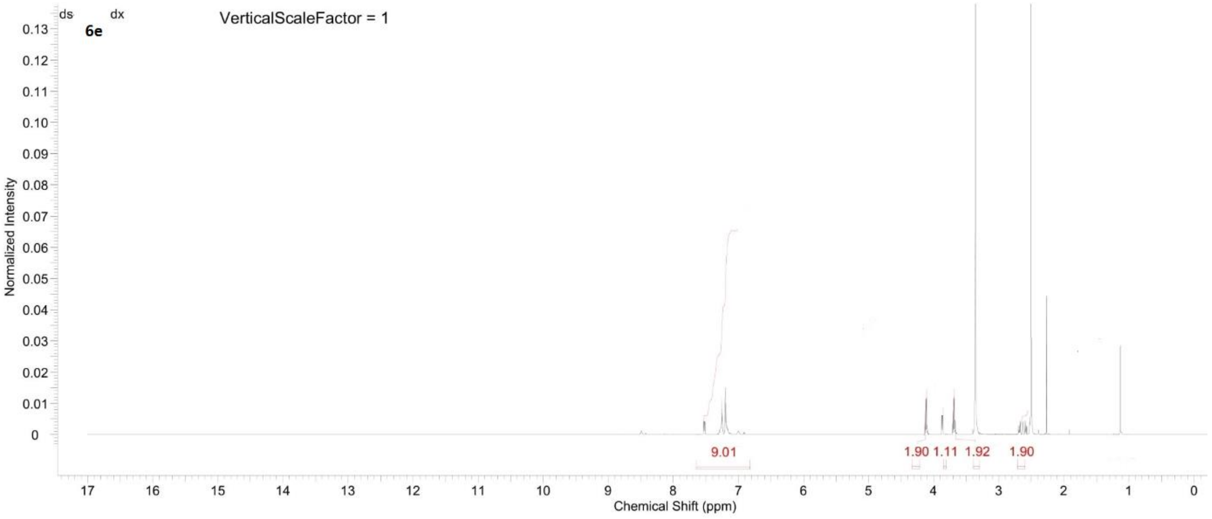

Compound 6f

^{13}C NMR spectrum

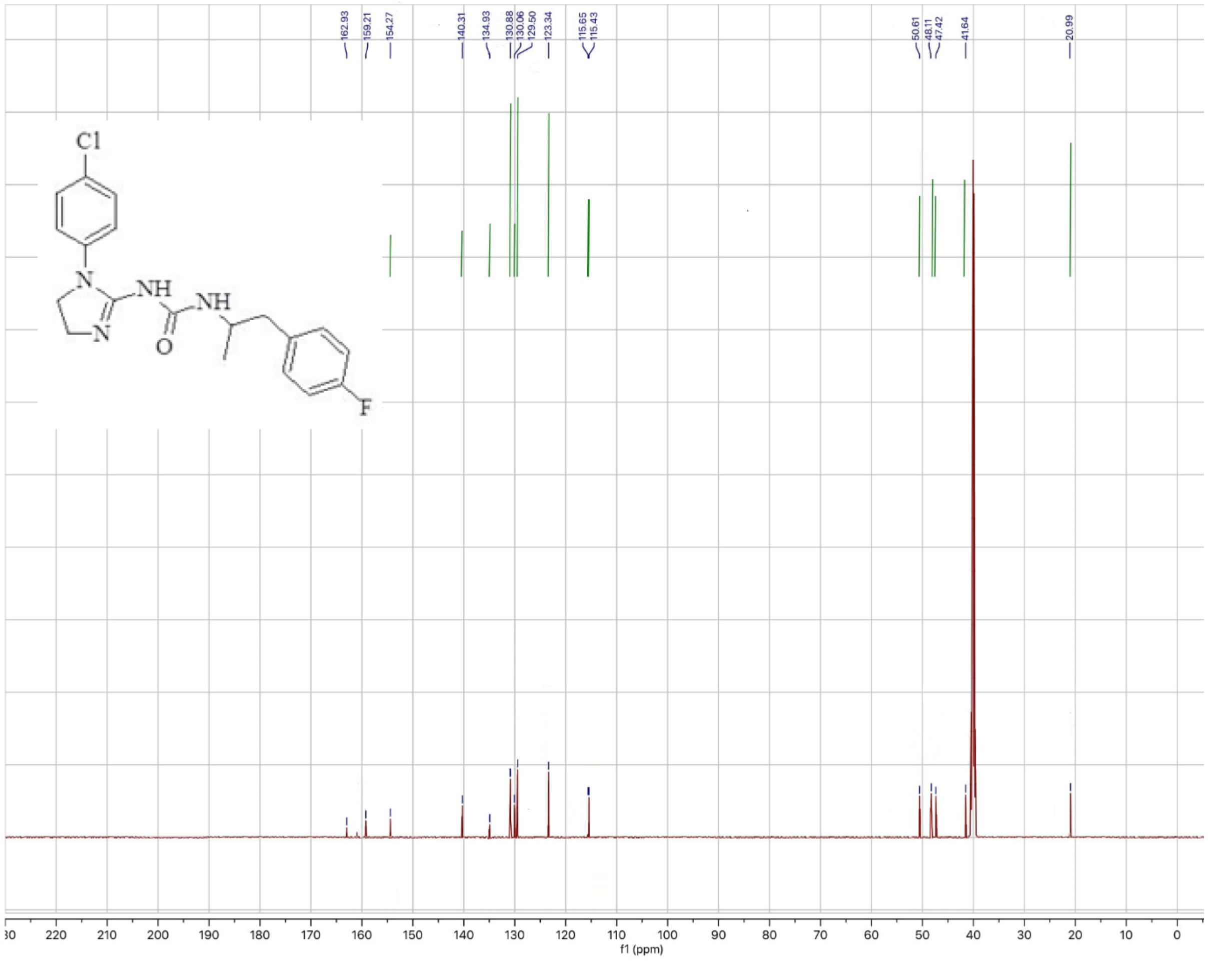

MS spectrum: $C_{19}H_{20}ClFN_4O$ (m.m. calc. 375.1382). HRMS (ESI) *m/z* $[M+H]^+$: 375.1360.

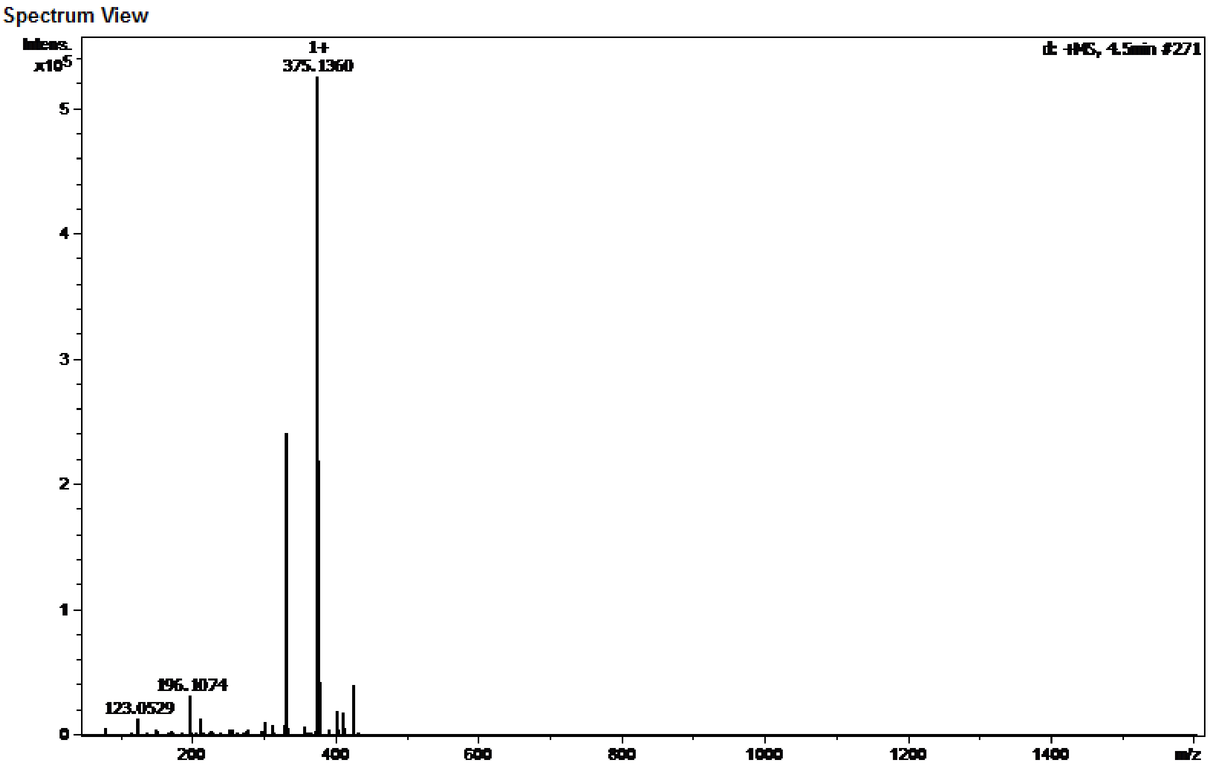

^{1}H NMR spectrum

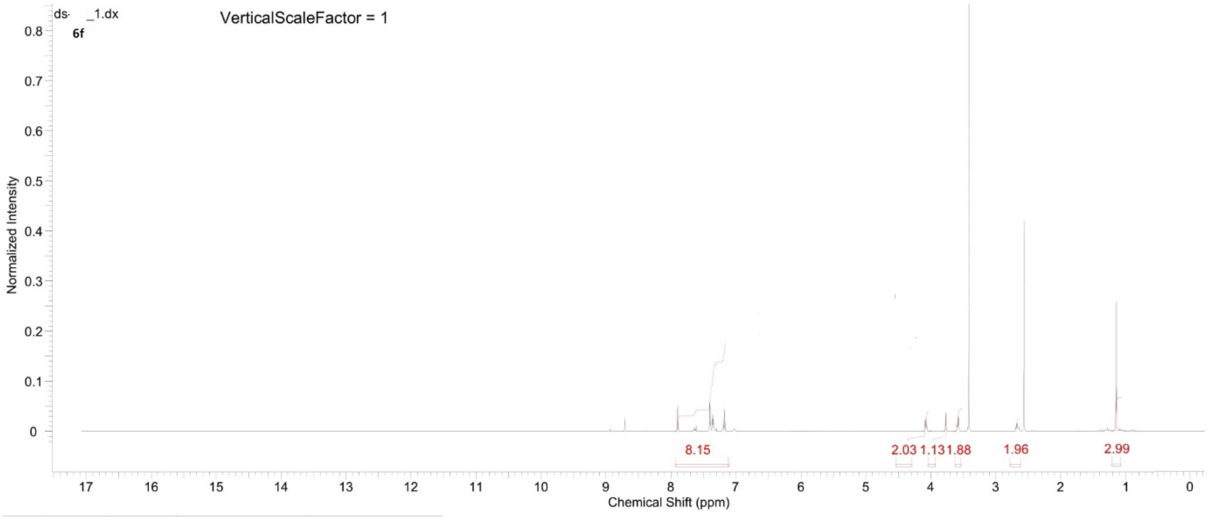

Compound 6g

^{13}C NMR spectrum

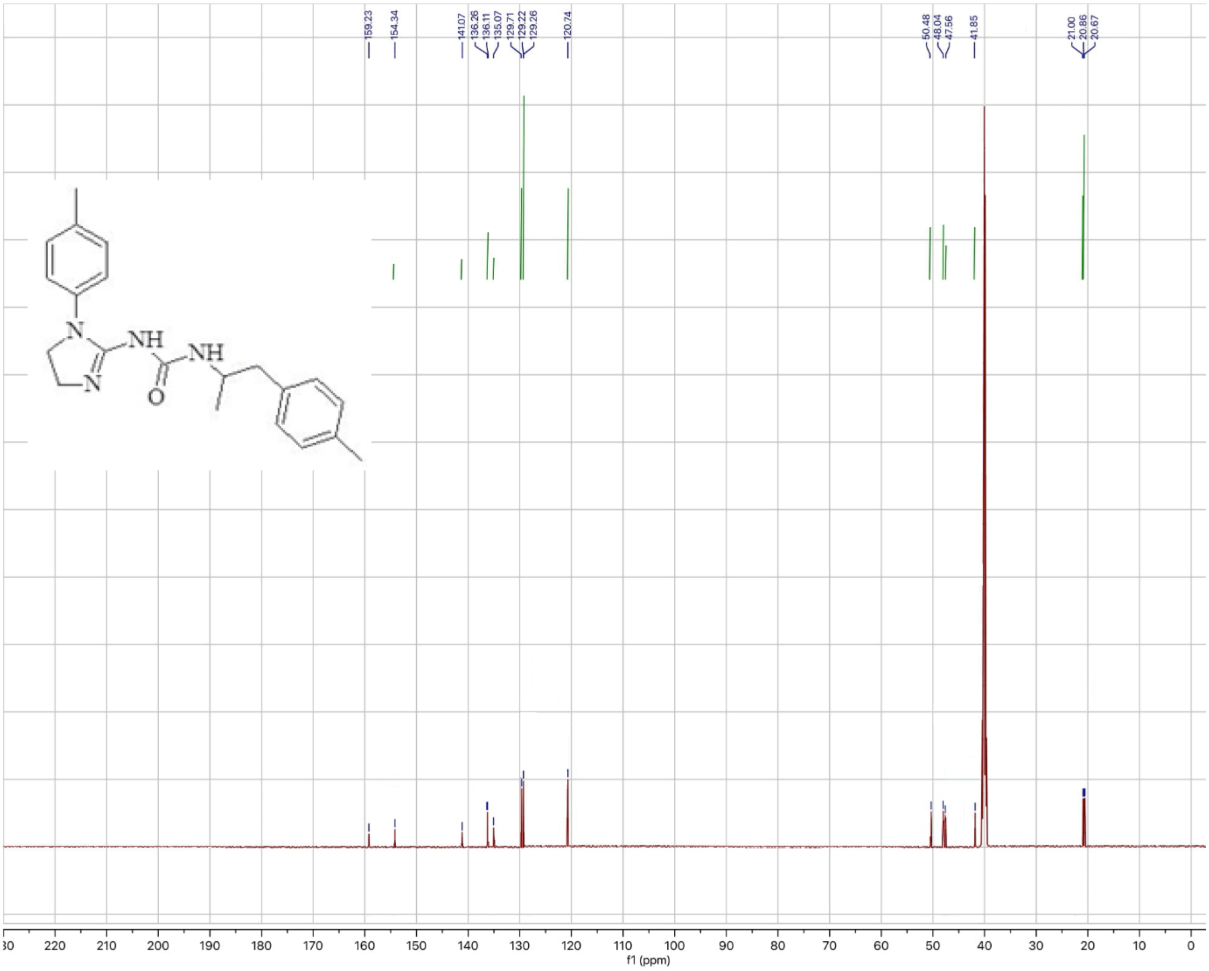

MS spectrum: $C_{21}H_{26}N_4O$ (m.m. calc. 351.2179). HRMS (ESI) *m/z* $[M+H]^+$: 351.2234.

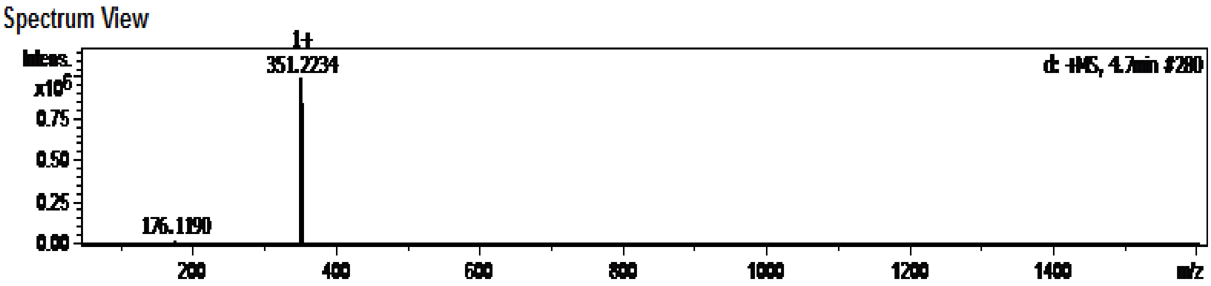

¹H NMR spectrum

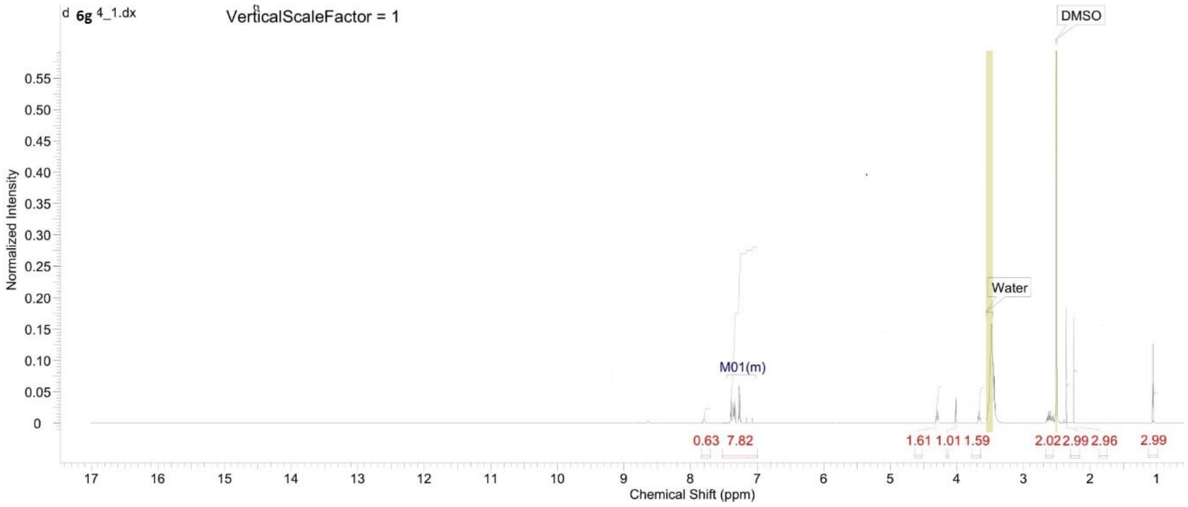

Compound 6h

¹³C NMR spectrum

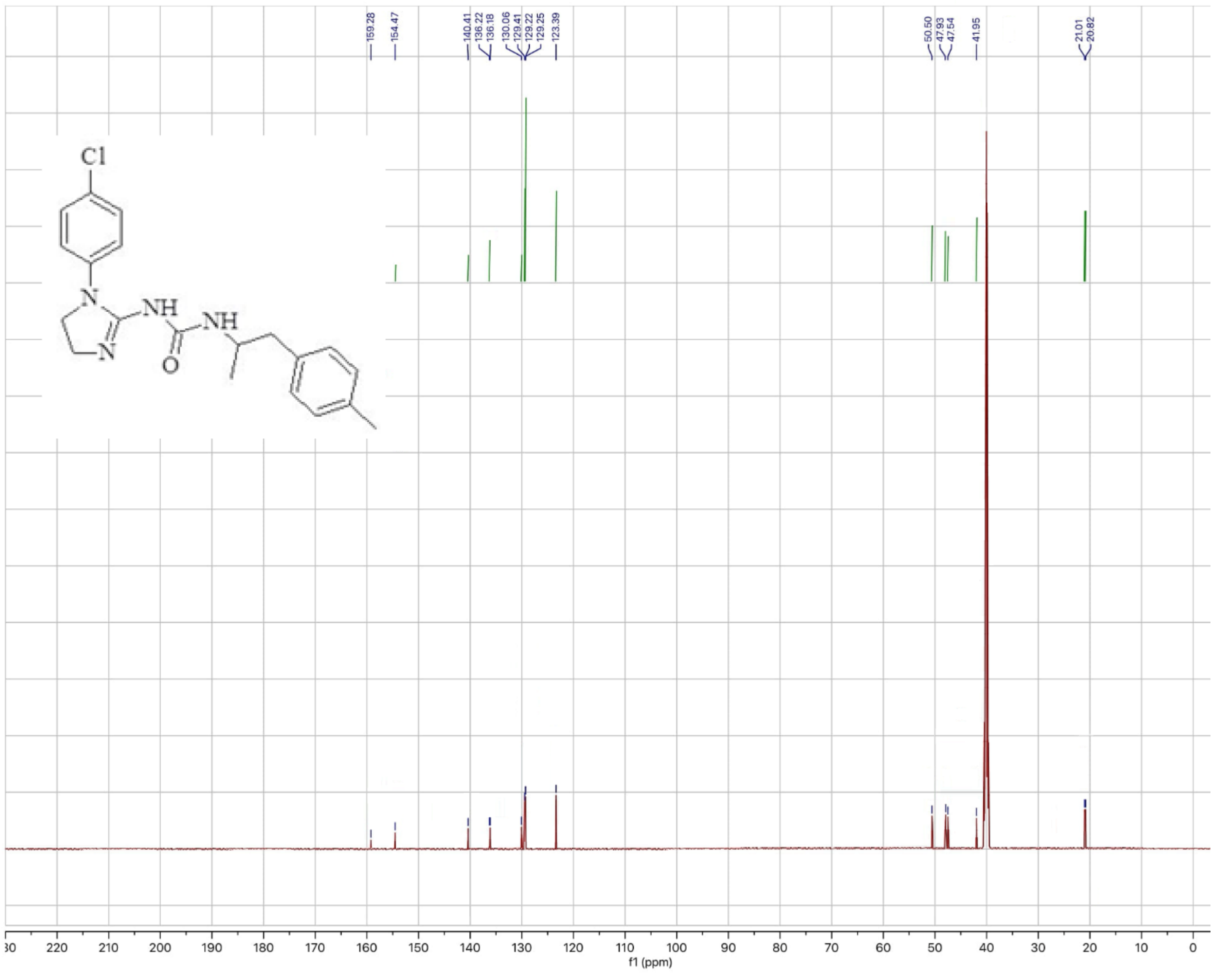

MS spectrum: $C_{20}H_{23}ClN_4O$ (m.m. calc. 371.1633). HRMS (ESI) *m/z* $[M+H]^+$: 371.2033.

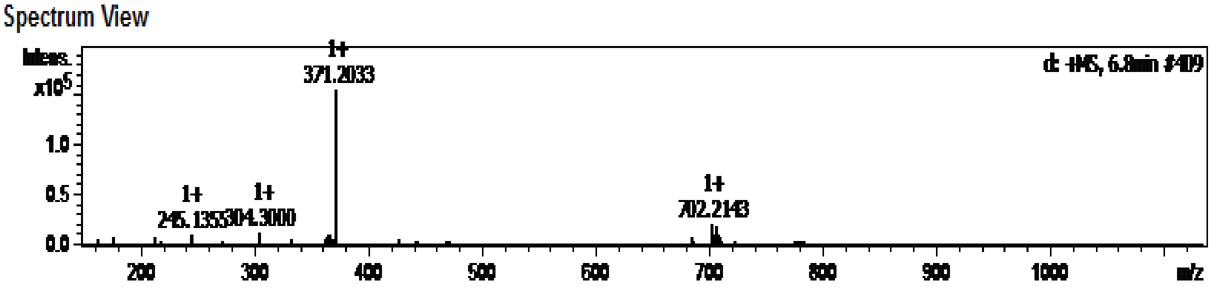

^{1}H NMR spectrum

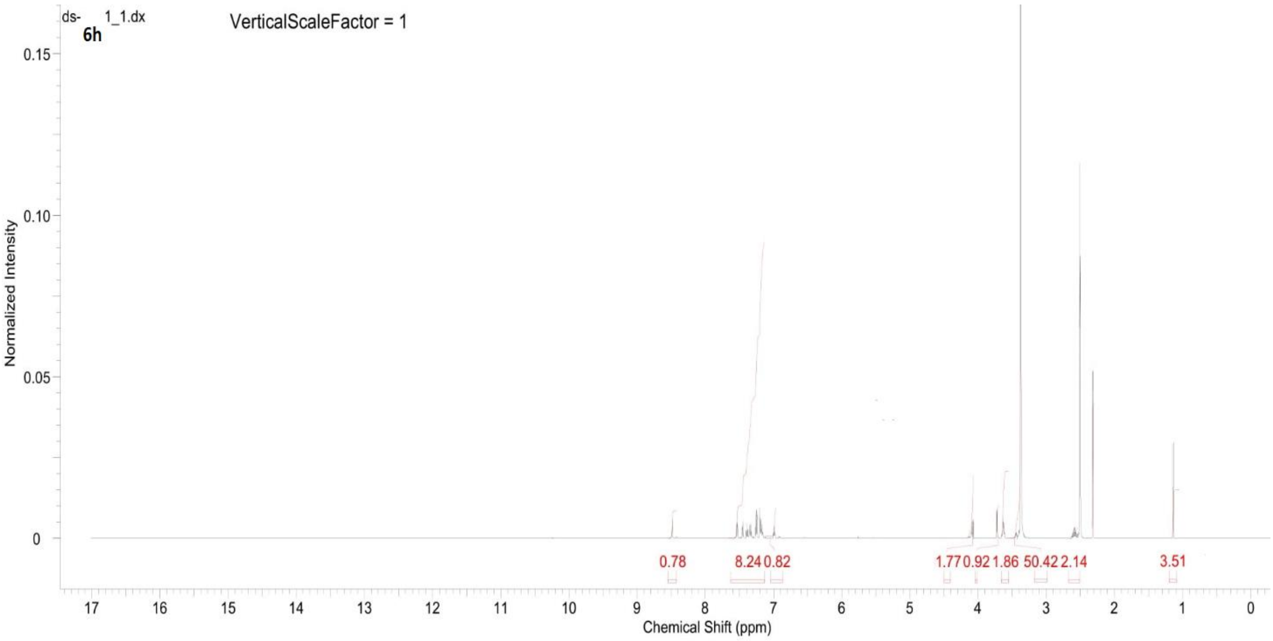

Compound 6i

^{13}C NMR spectrum

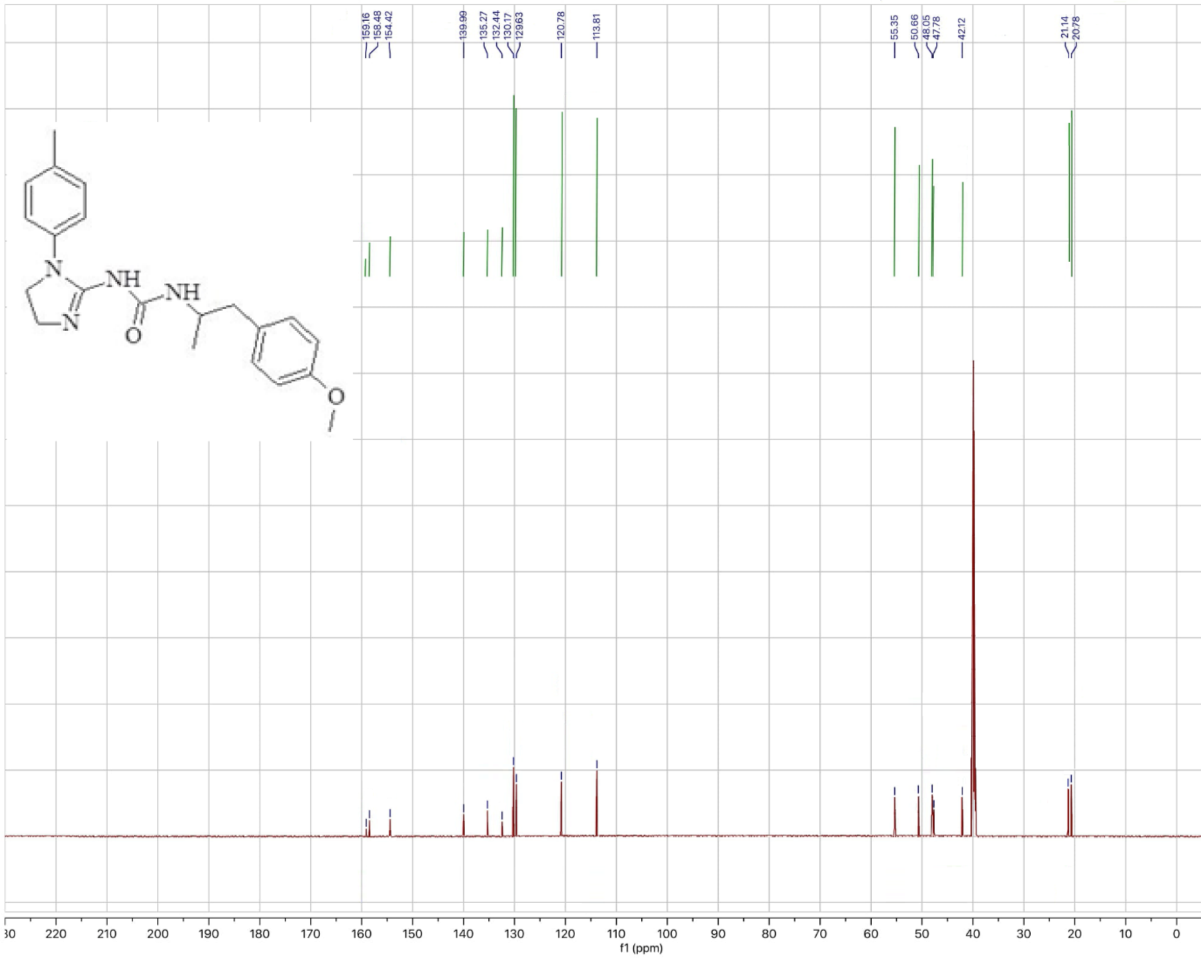

MS spectrum: $C_{21}H_{26}N_4O_2$ (m.m. calc. 367.2128). HRMS (ESI) *m/z* [M+H]$^+$: 367.2132.

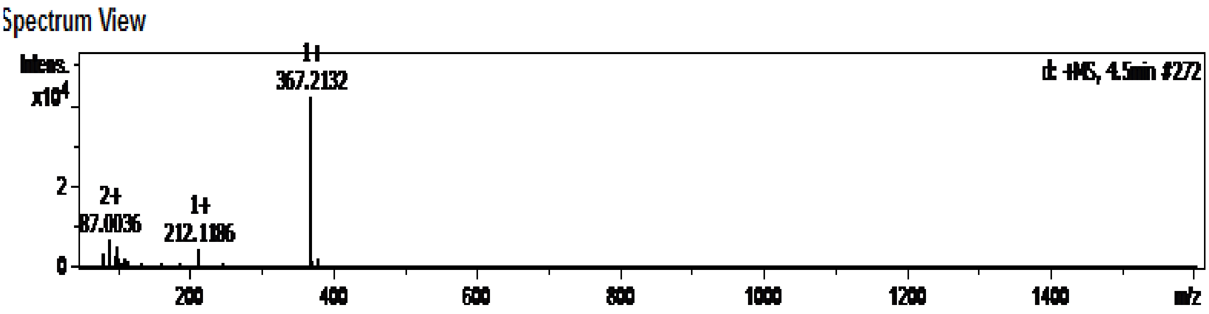

^{1}H NMR spectrum

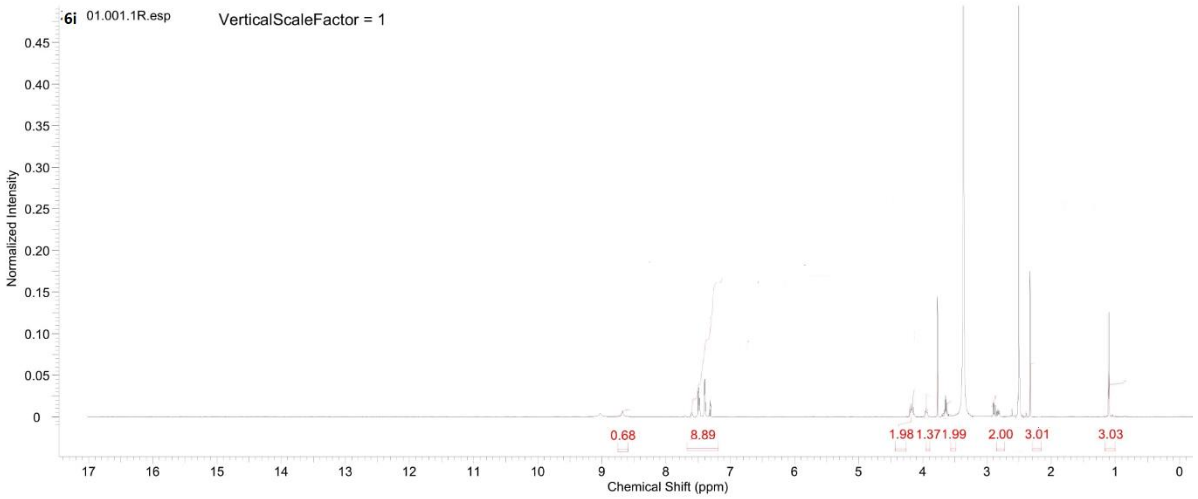

Compound 6j

^{13}C NMR spectrum

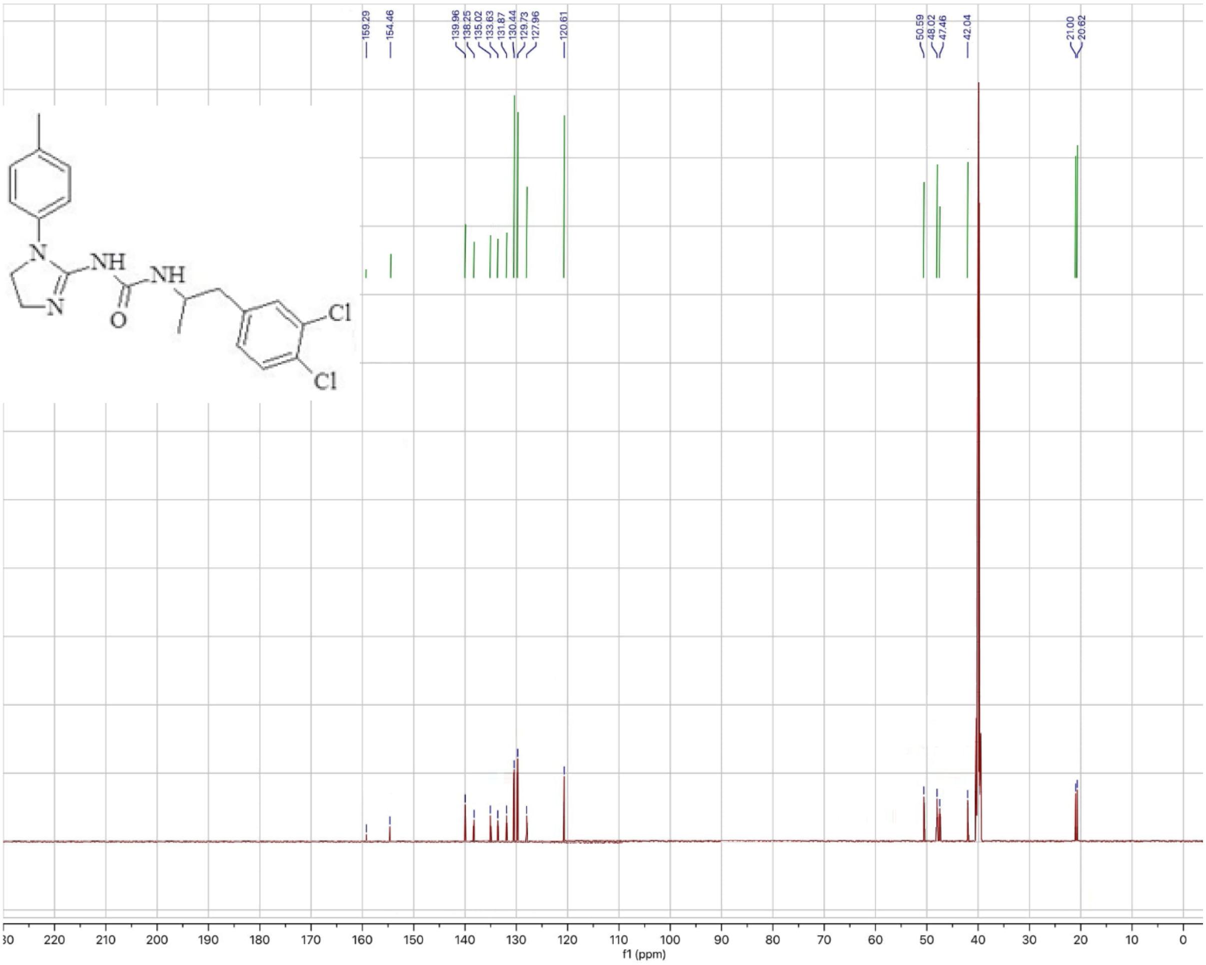

MS spectrum: $C_{20}H_{22}Cl_2N_4O$ (m.m. calc. 405.1243). HRMS (ESI) *m/z* $[M+H]^+$: 405.1271.

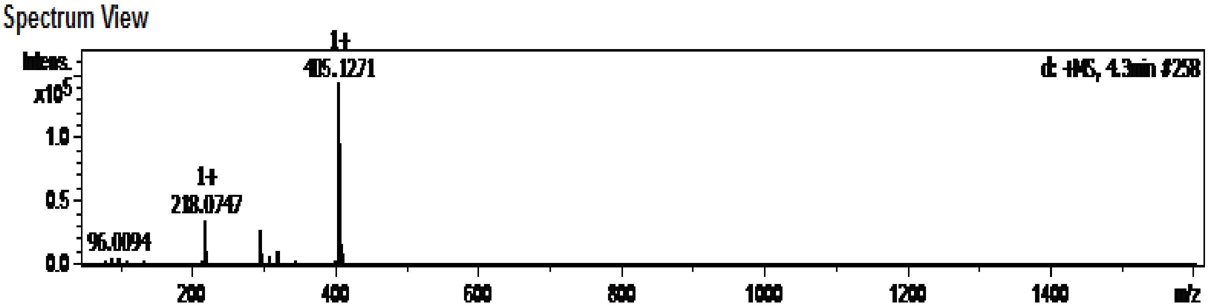

^{1}**H NMR spectrum**

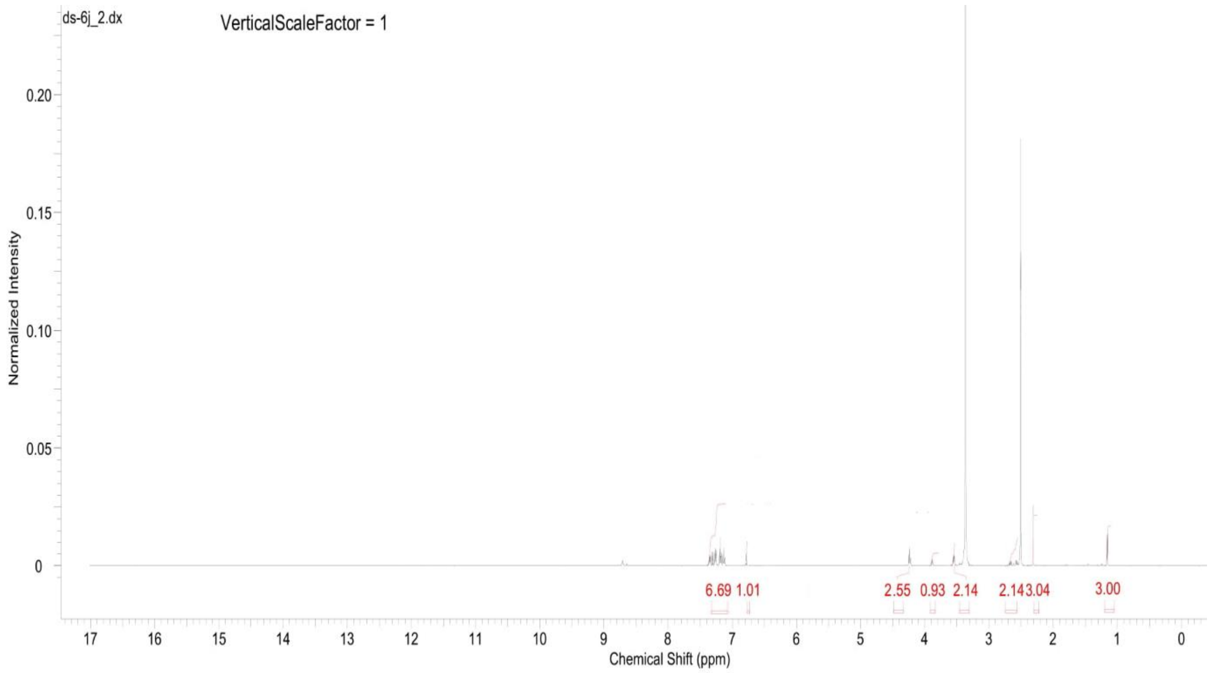

Compound 6k

^{13}C NMR spectrum

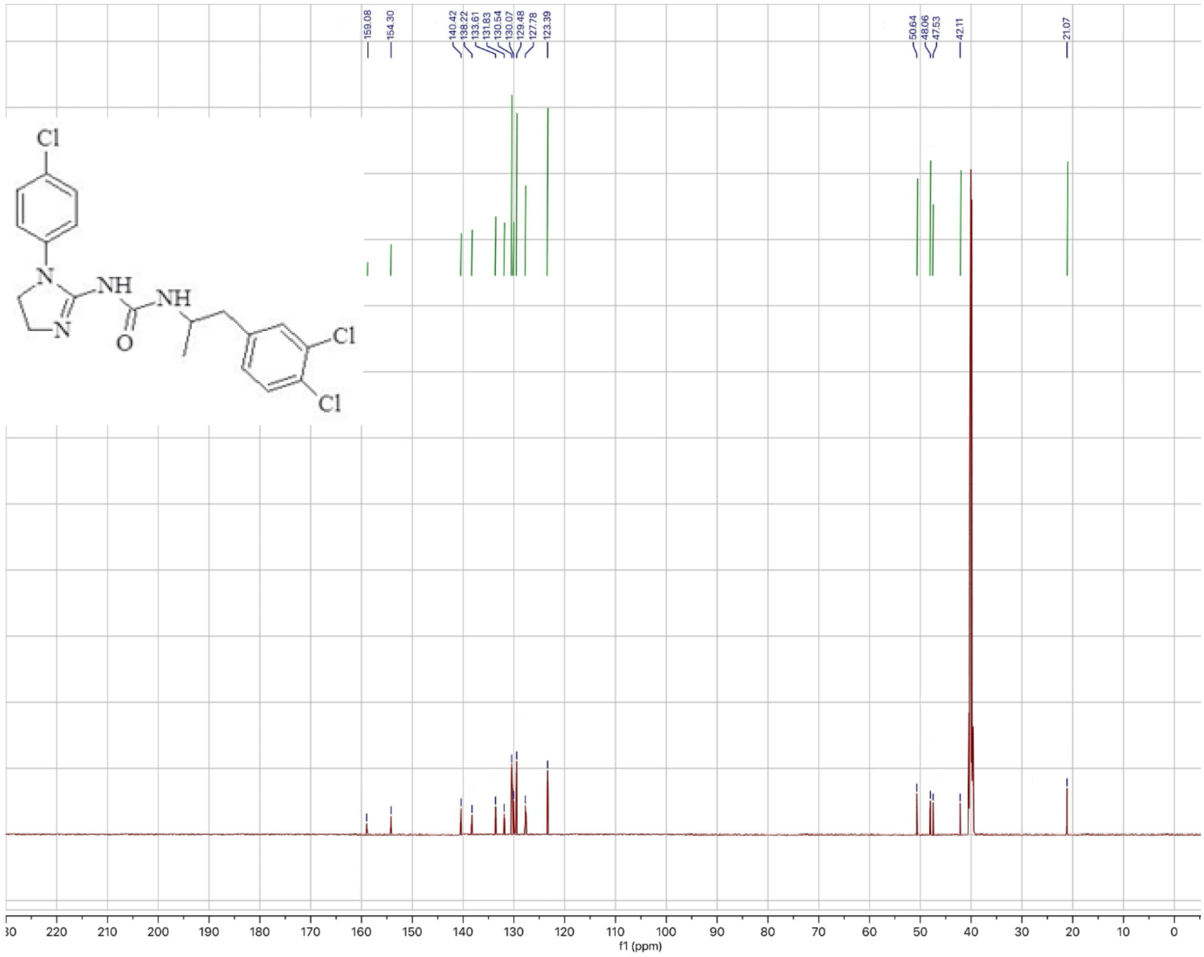

MS spectrum: $C_{19}H_{19}Cl_3N_4O$ (m.m. calc. 425.0670). HRMS (ESI) *m/z* $[M+H]^+$: 425.0679.

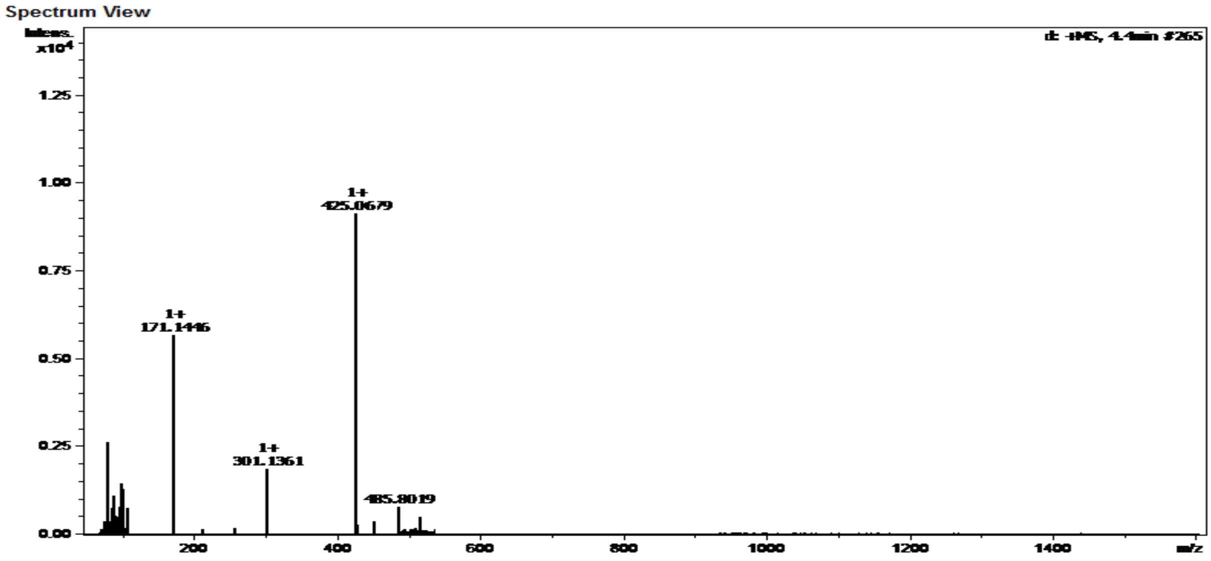

^{1}H NMR spectrum

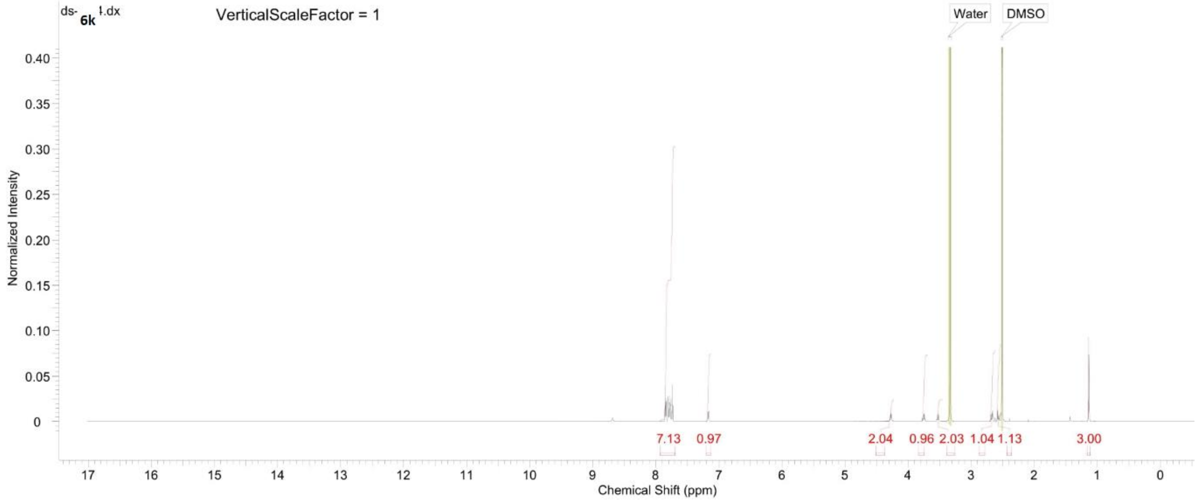

Compound 6l

^{13}C NMR spectrum

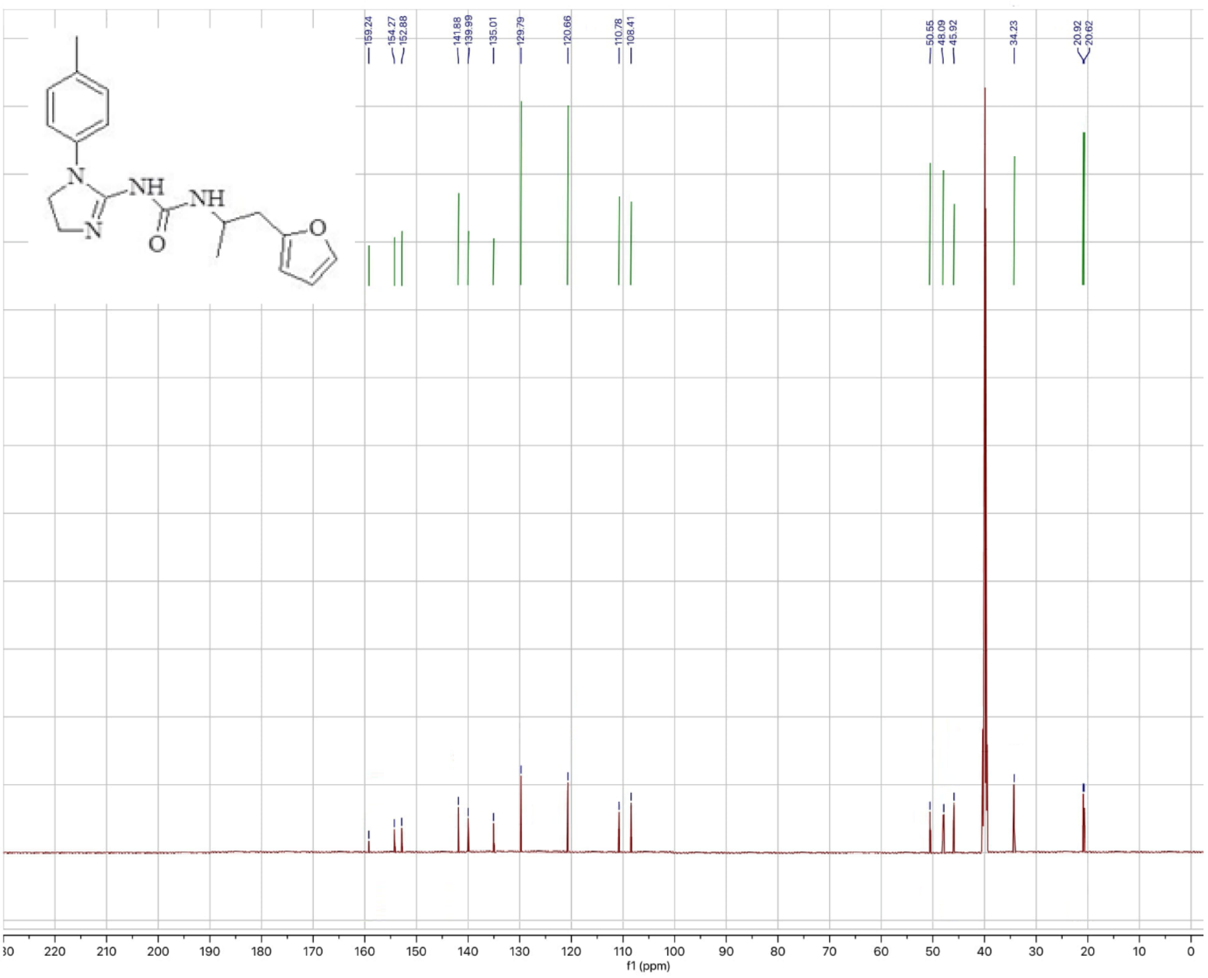

MS spectrum: $C_{18}H_{22}N_4O_2$ (m.m. calc. 327.1816). HRMS (ESI) *m/z* $[M+H]^+$: 327.2054.

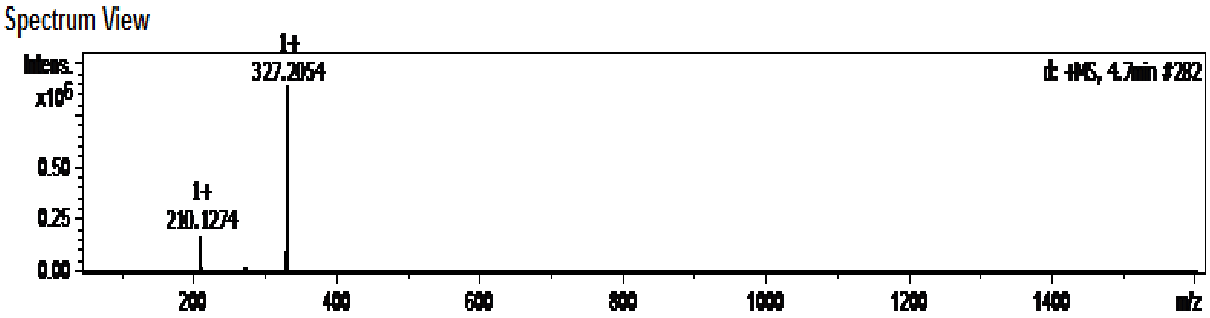

^{1}H NMR spectrum

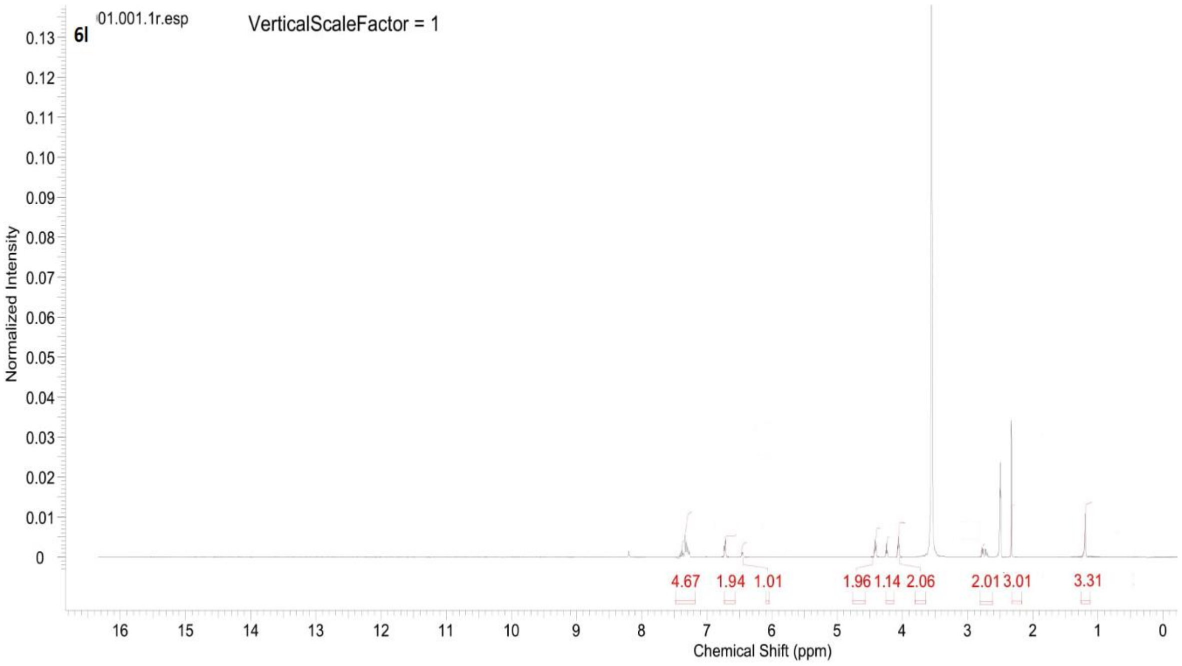

Compound 6m

^{13}C NMR spectrum

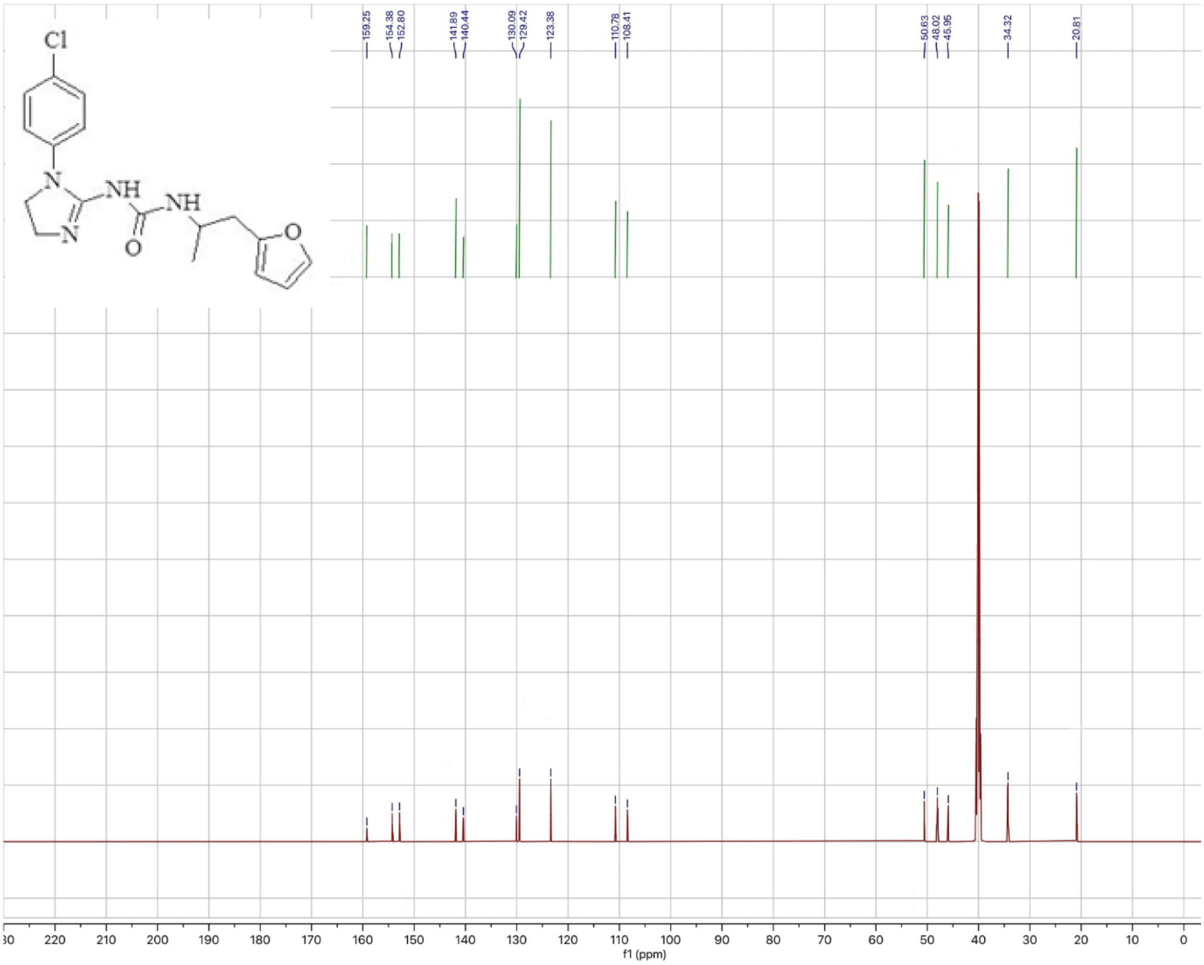

MS spectrum: $C_{17}H_{19}ClN_4O_2$ (m.m. calc. 347.1269). HRMS (ESI) *m/z* $[M+H]^+$: 347.1232.

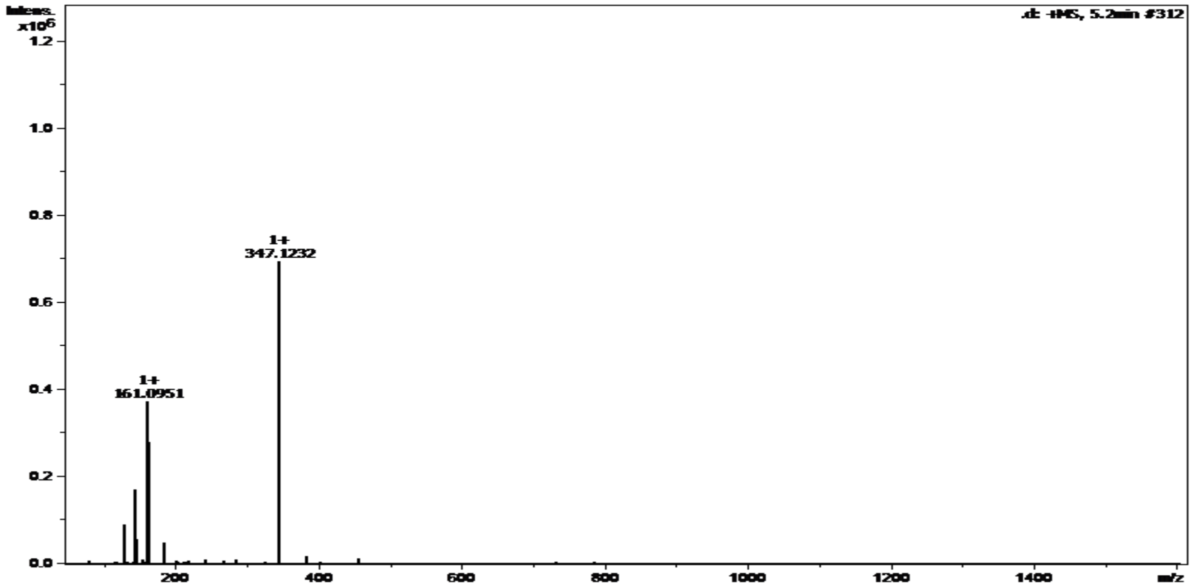

^{1}H NMR spectrum

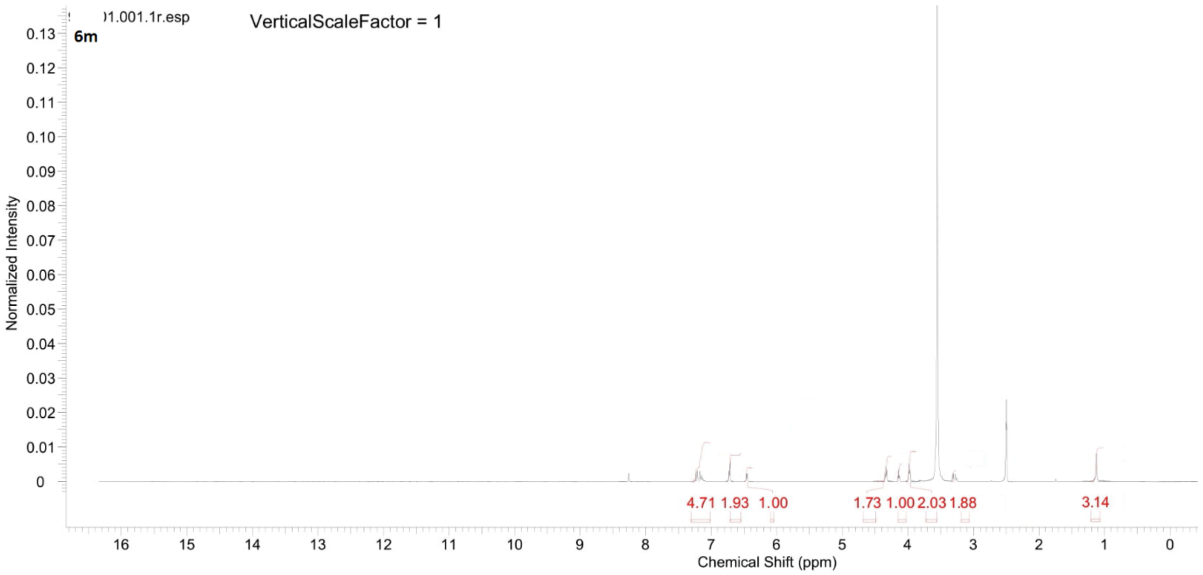

Compound 6n

^{13}C NMR spectrum

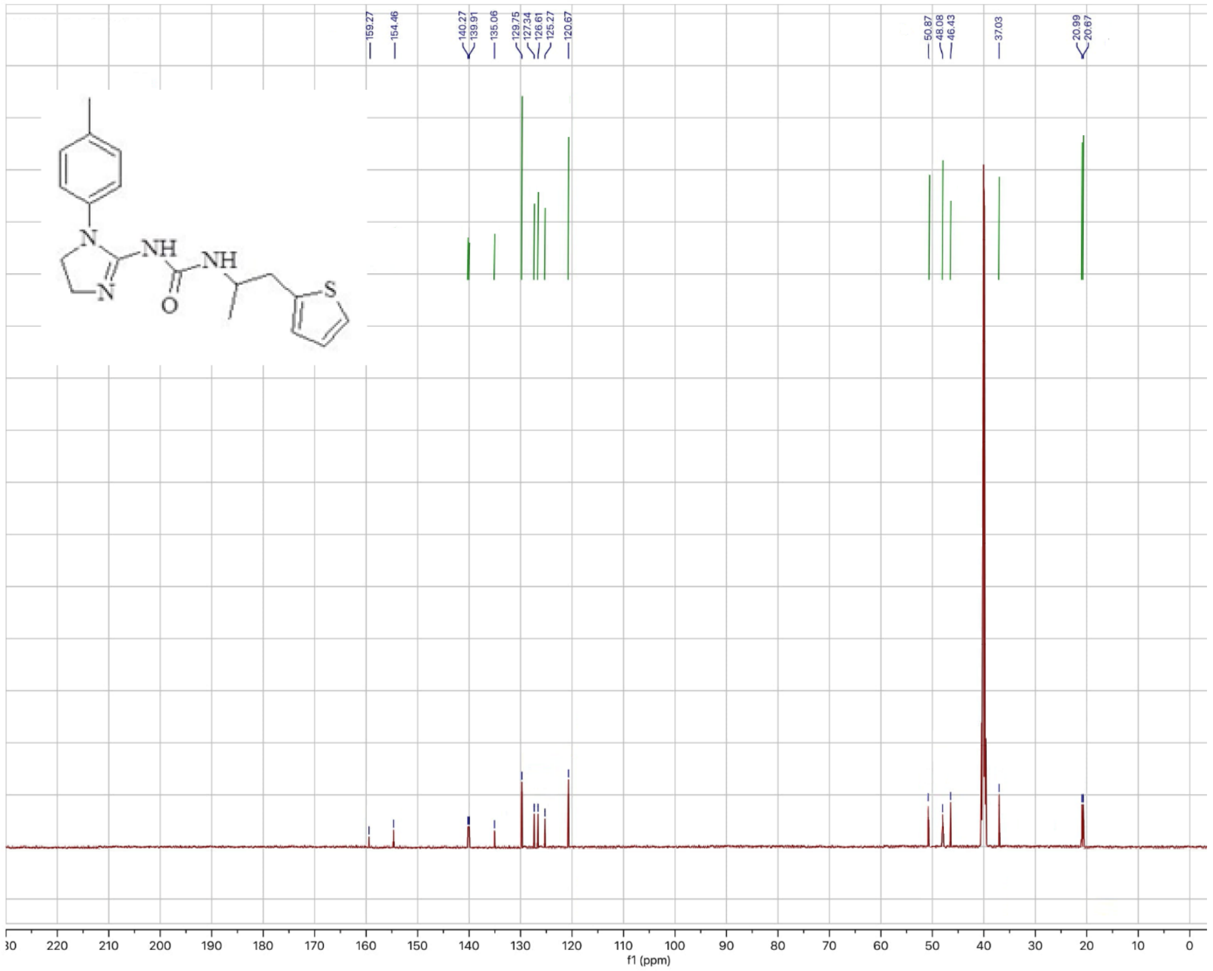

MS spectrum: $C_{18}H_{22}N_4OS$ (m.m. calc. 343.1587). HRMS (ESI) *m/z* $[M+H]^+$: 343.1728.

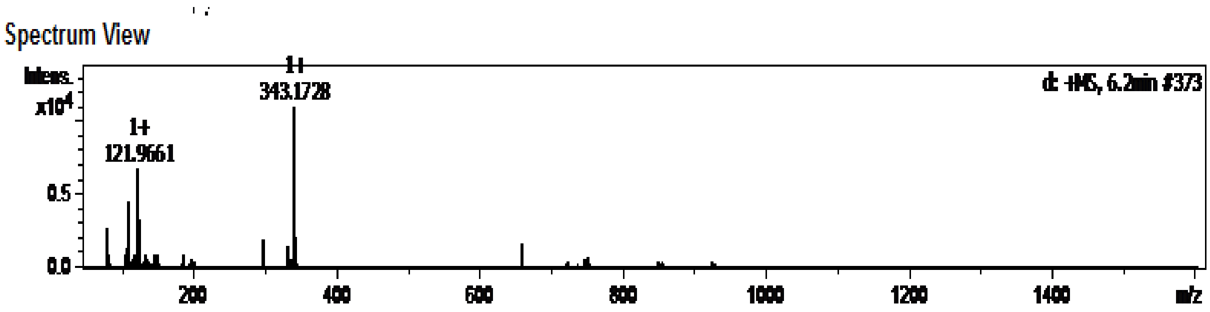

^{1}H NMR spectrum

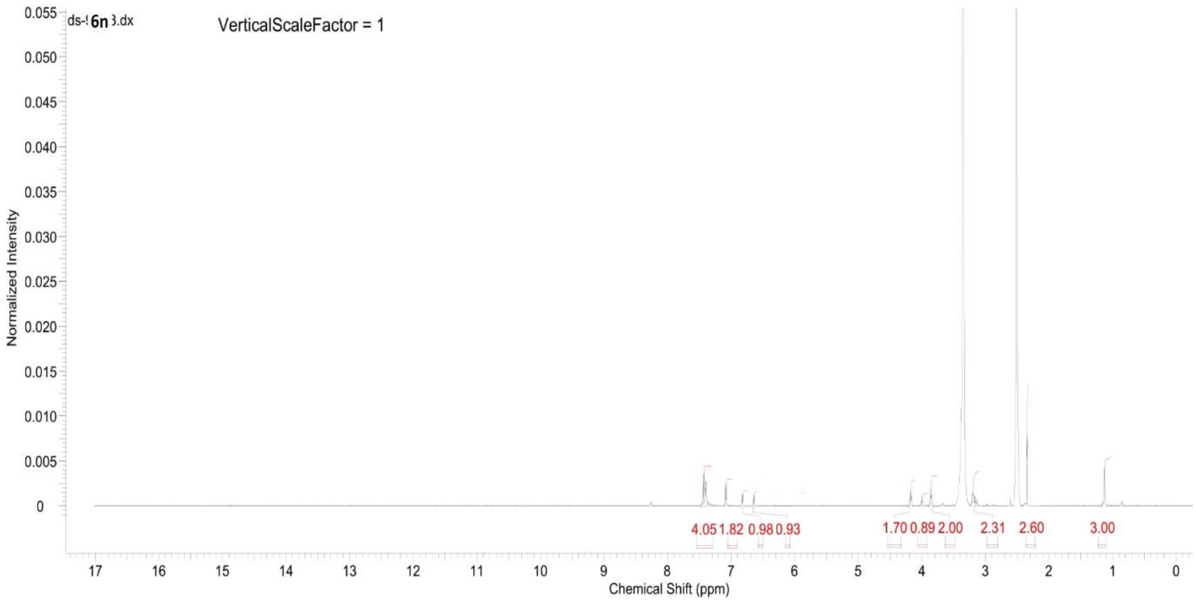

Compound 6o

^{13}C NMR spectrum

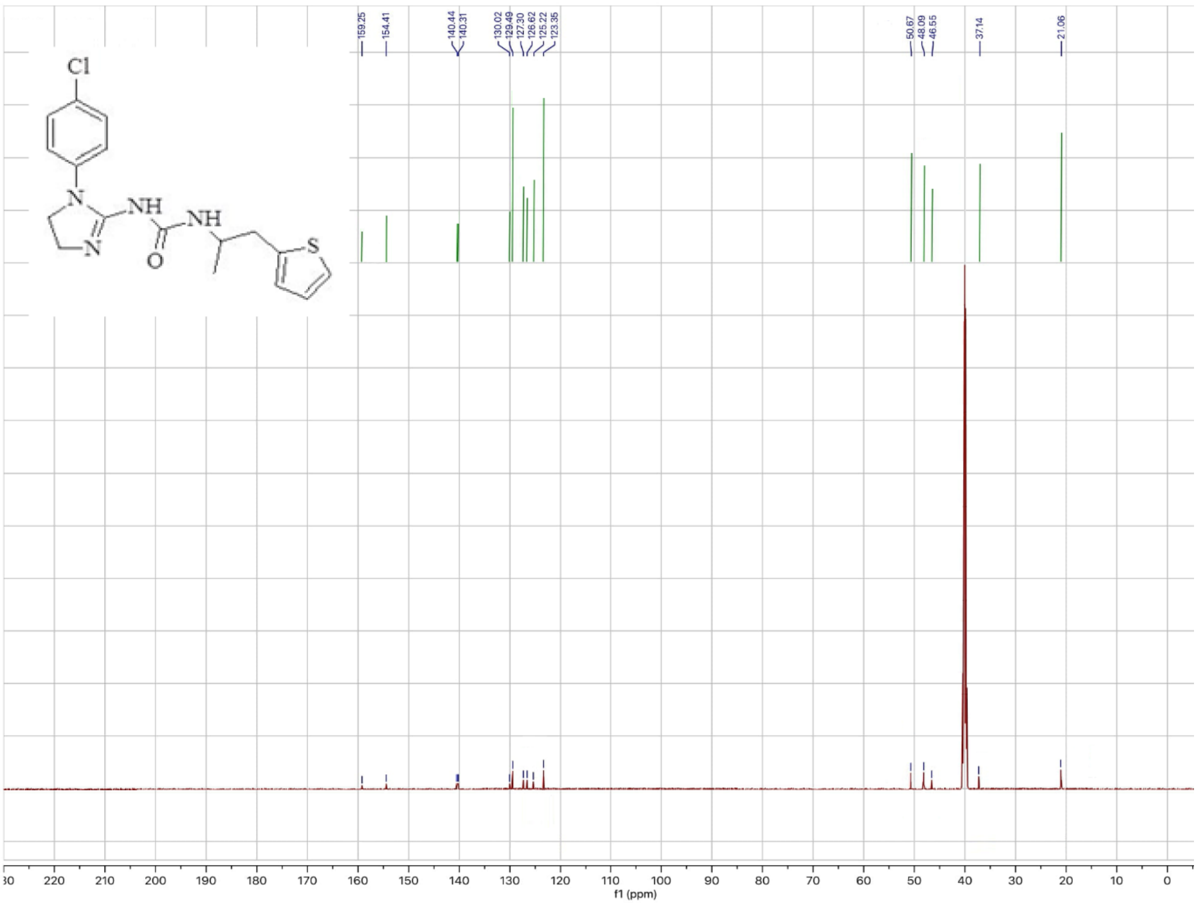

MS spectrum: $C_{17}H_{19}ClN_4OS$ (m.m. calc. 363.1041). HRMS (ESI) *m/z* $[M+H]^+$: 363.1733.

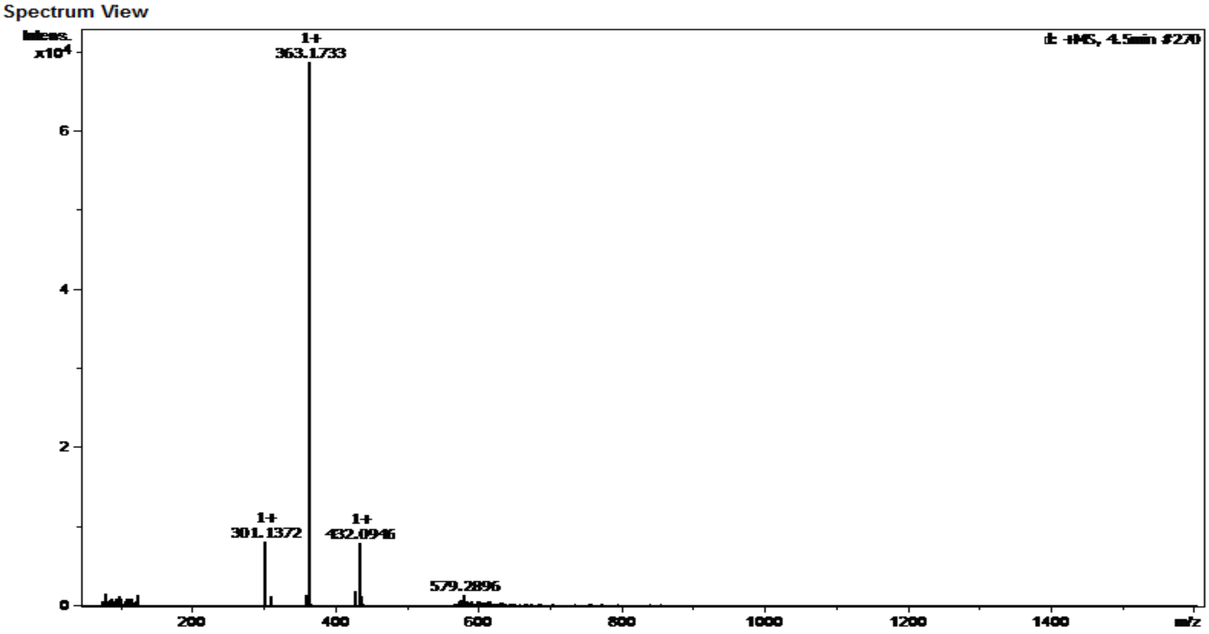

^{1}H NMR spectrum

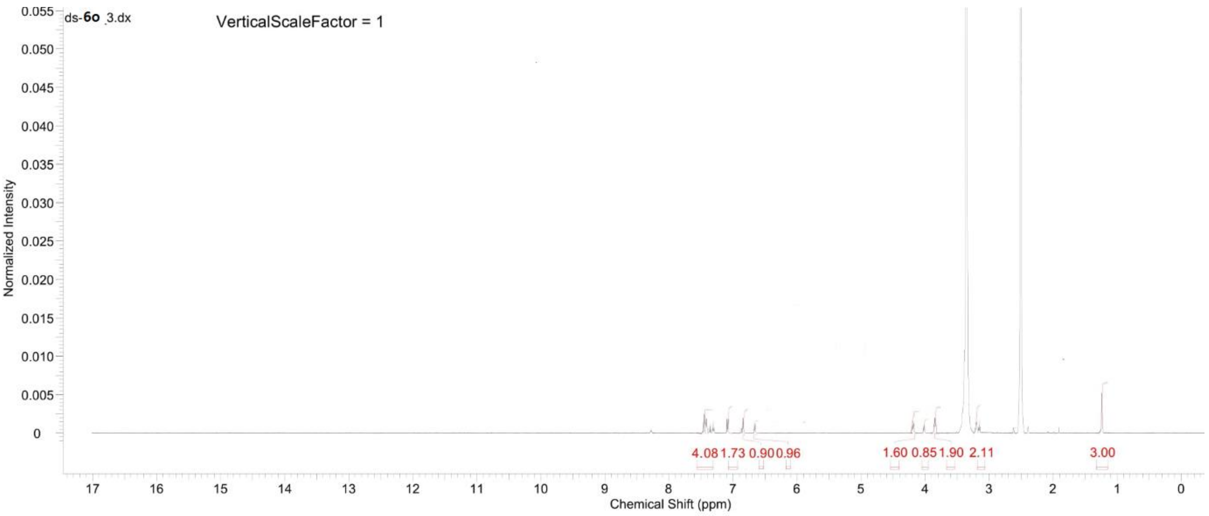

Compound 6p

^{13}C NMR spectrum

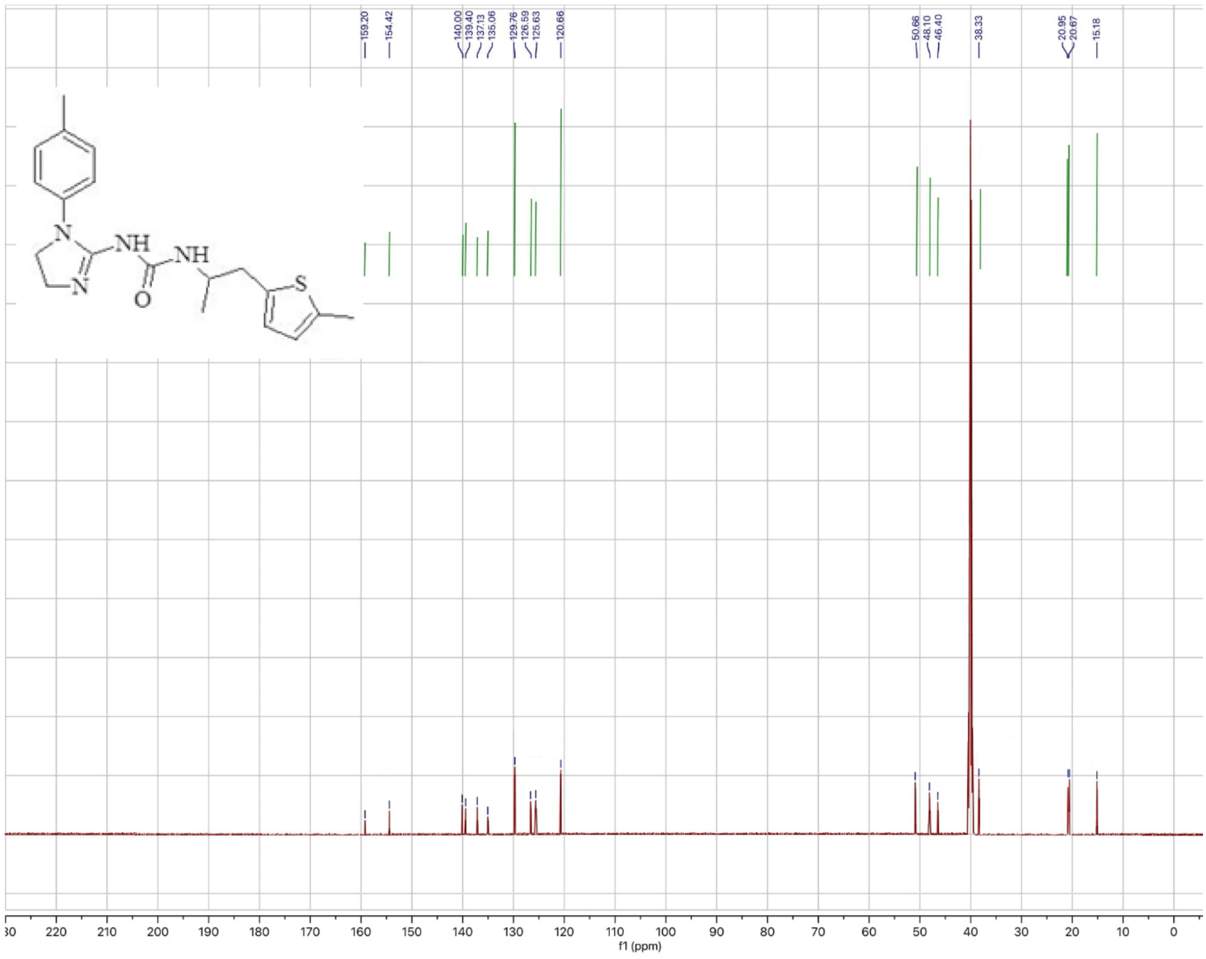

MS spectrum: $C_{19}H_{24}N_4OS$ (m.m. calc. 357.1744). HRMS (ESI) *m/z* $[M+H]^+$: 357.2069.

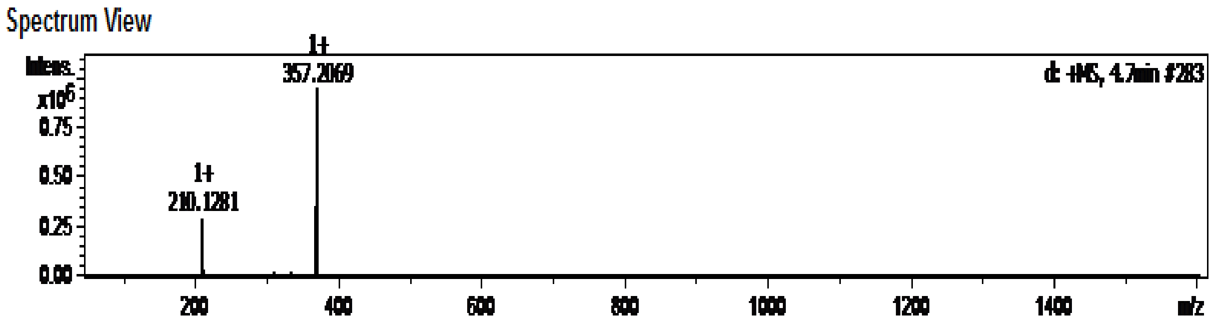

^{1}H NMR spectrum

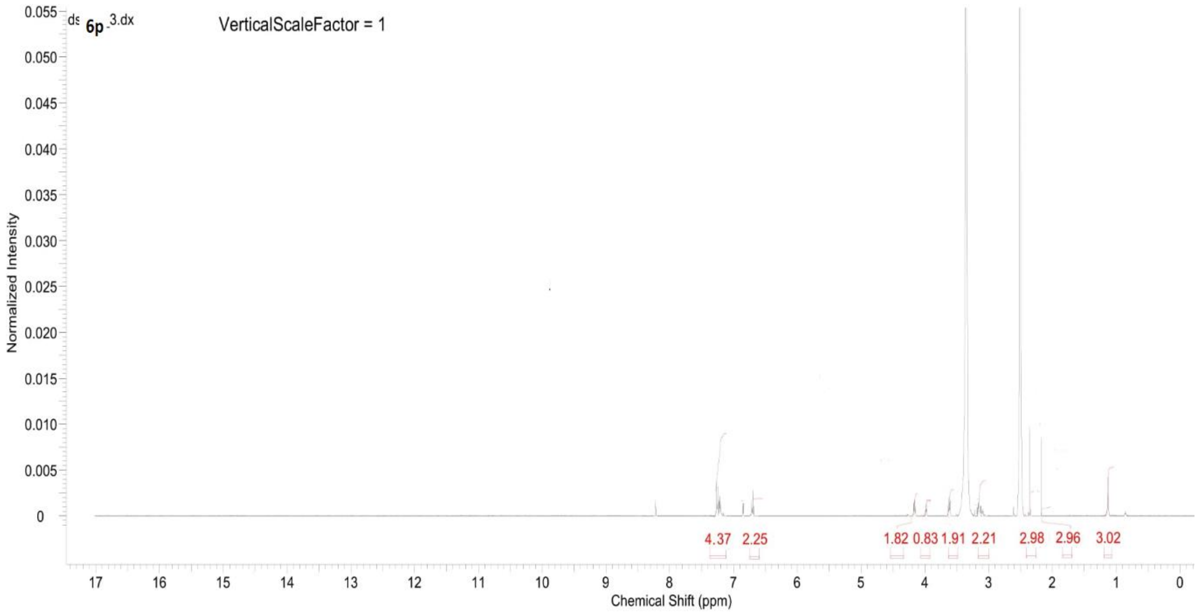

Compound 6r

^{13}C NMR spectrum

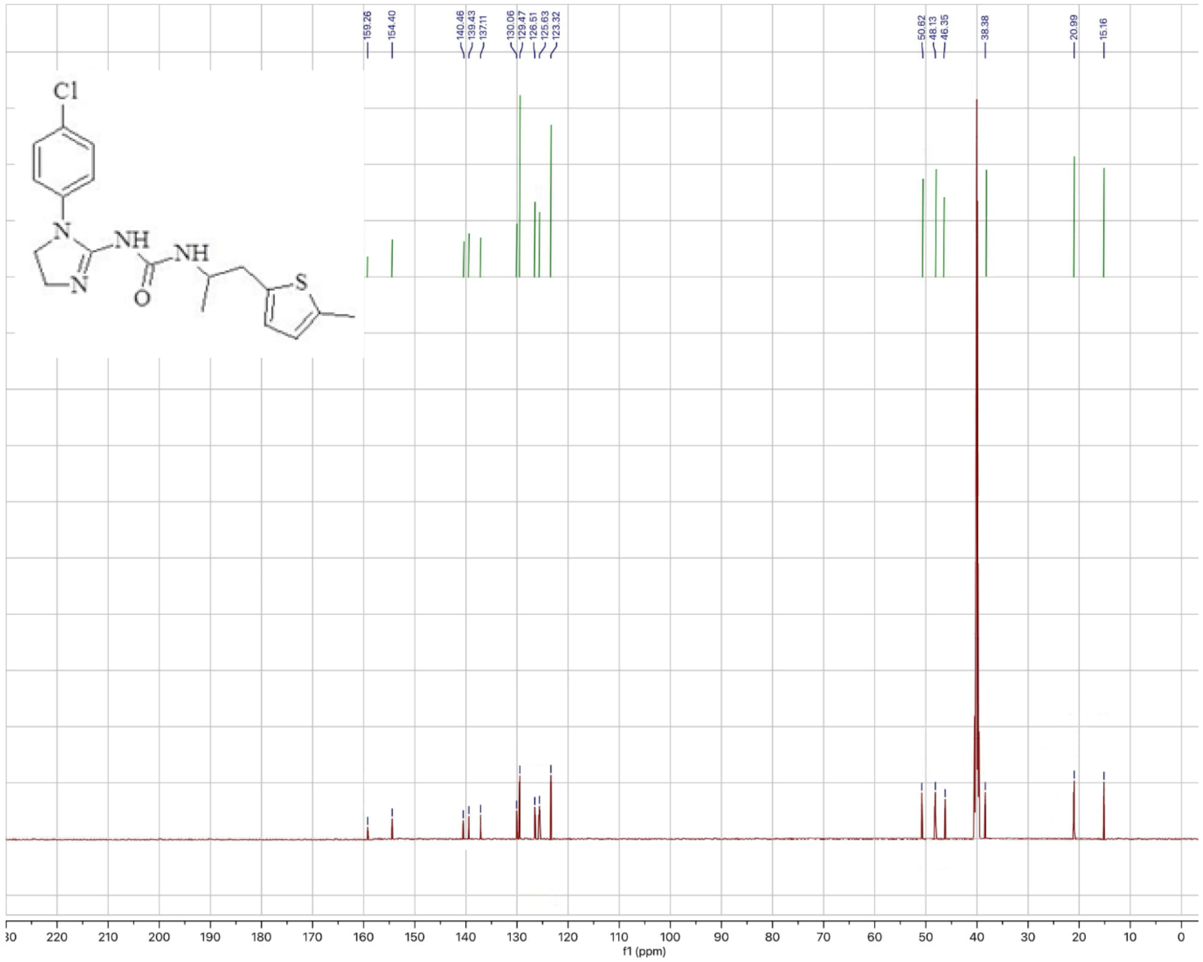

MS spectrum: $C_{18}H_{21}ClN_4OS$ (m.m. calc. 377.1197). HRMS (ESI) *m/z* $[M+H]^+$: 377.1178.

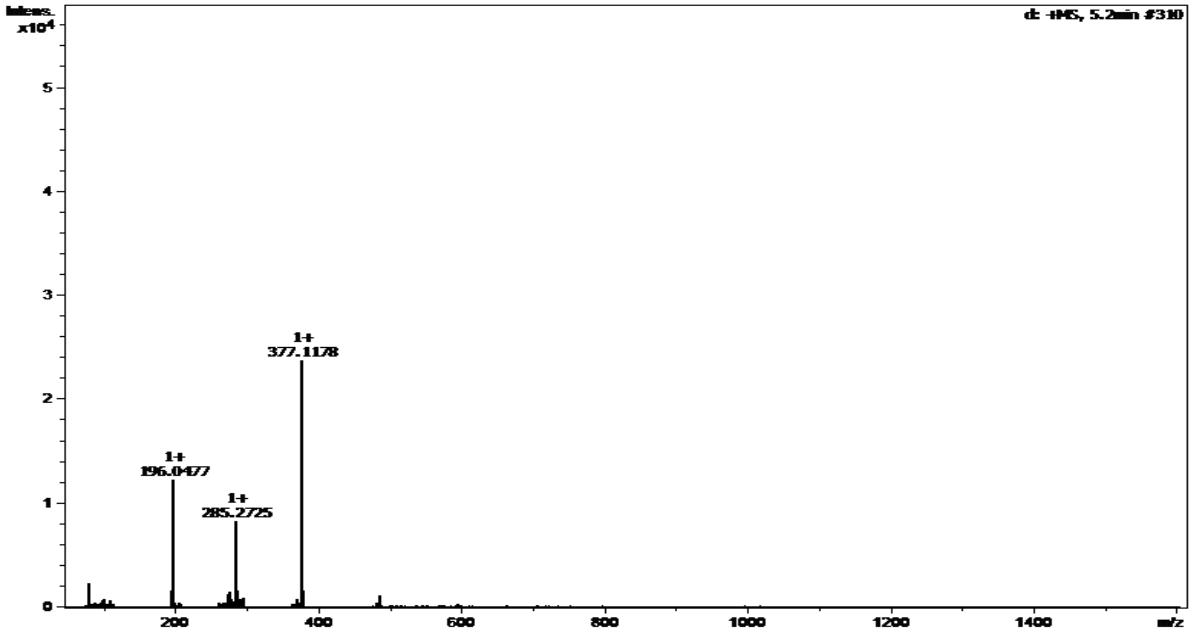

^{1}H NMR spectrum

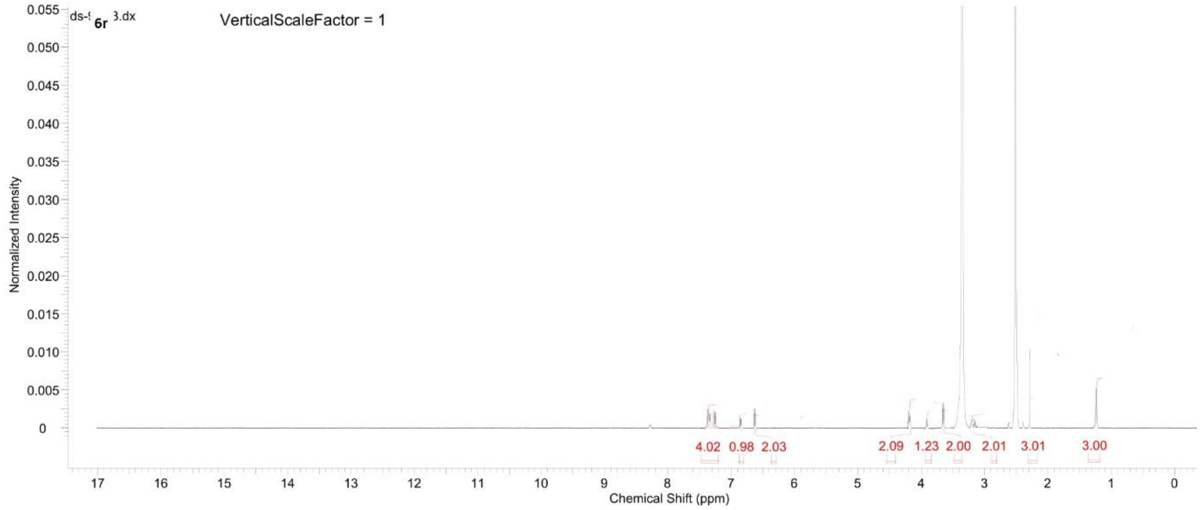

Compound 7a

^{13}C NMR spectrum

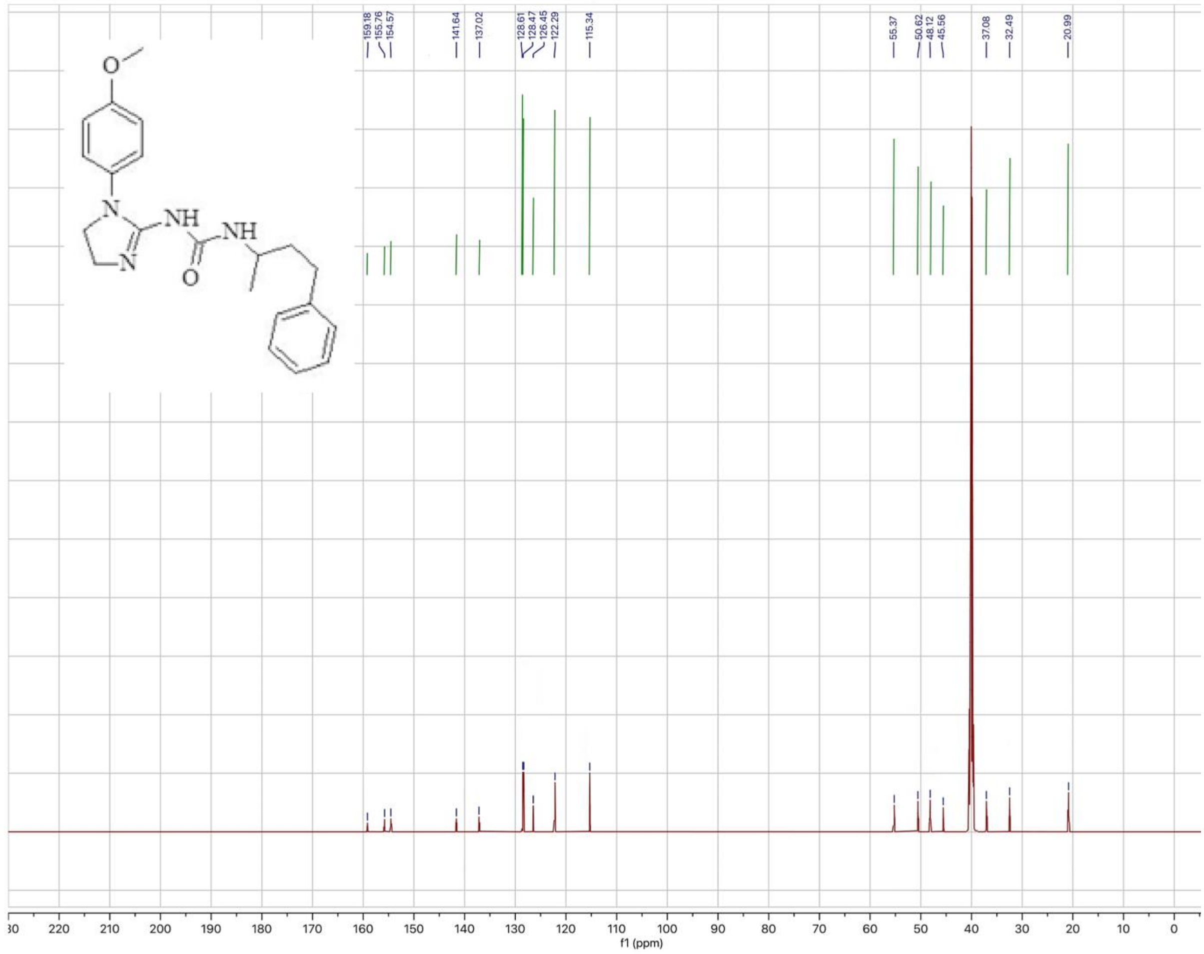

MS spectrum: $C_{21}H_{26}N_4O_2$ (m.m. calc. 367.2128). HRMS (ESI) *m/z* $[M+H]^+$: 367.2181.

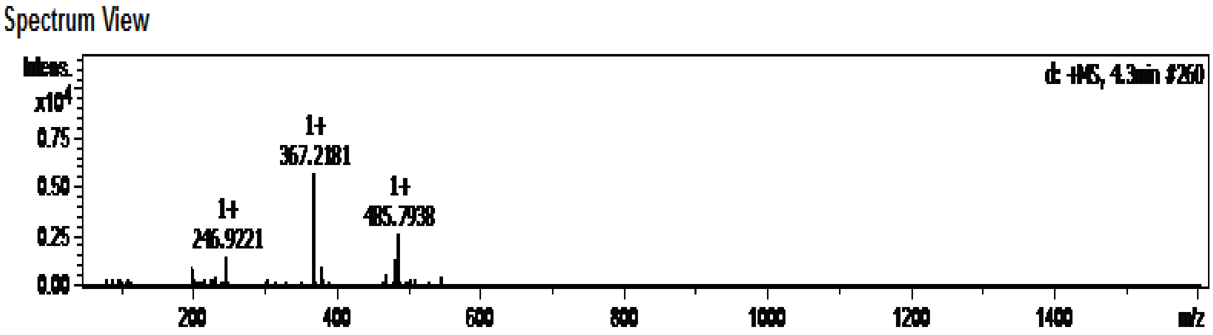

^{1}**H NMR spectrum**

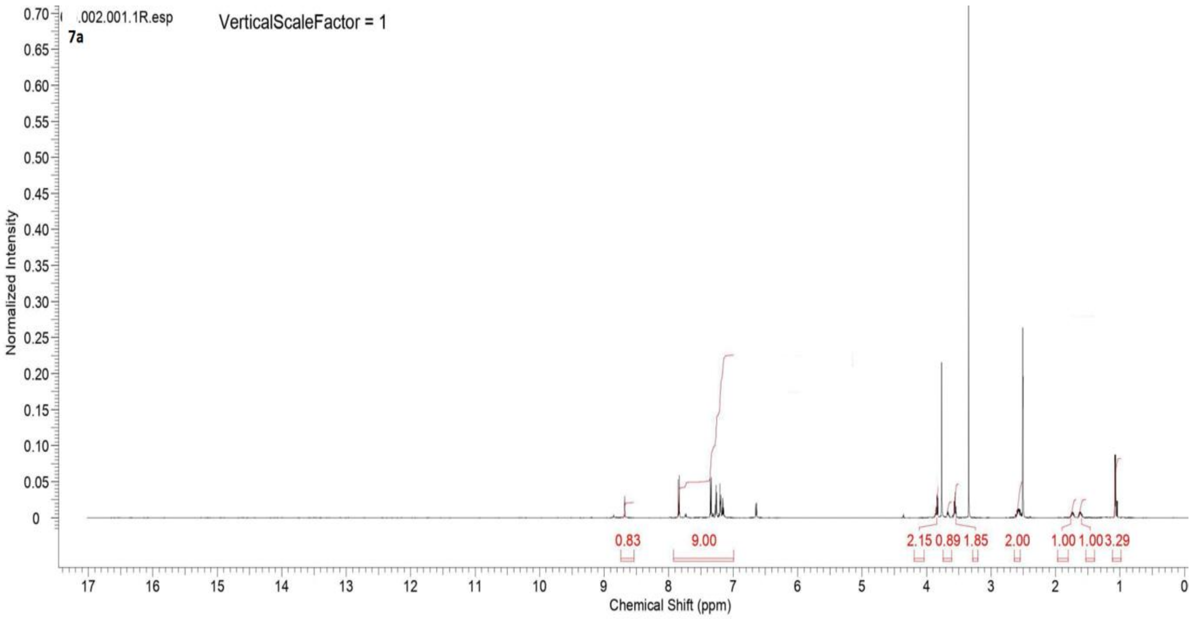

Compound 7b

^{13}C NMR spectrum

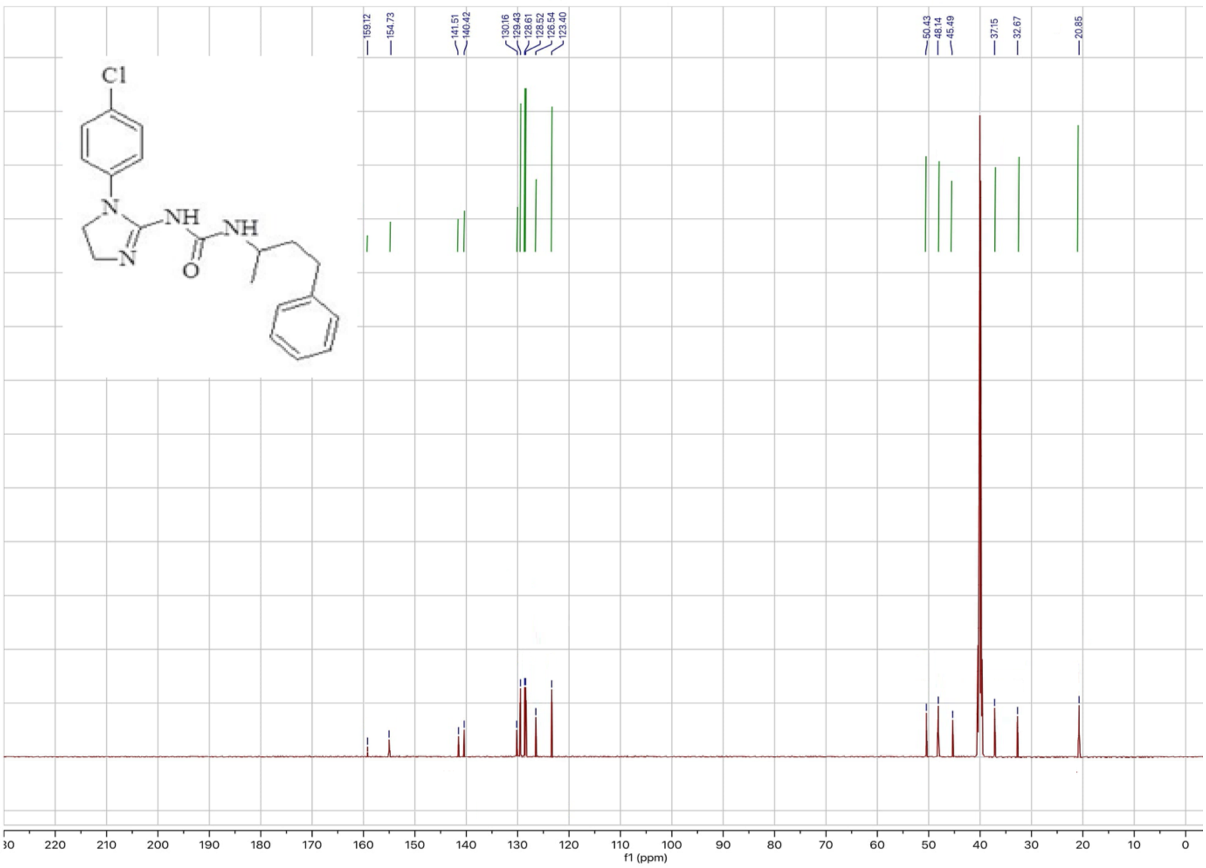

MS spectrum: $C_{20}H_{23}ClN_4O$ (m.m. calc. 371.1633). HRMS (ESI) *m/z* $[M+H]^+$: 371.1720.

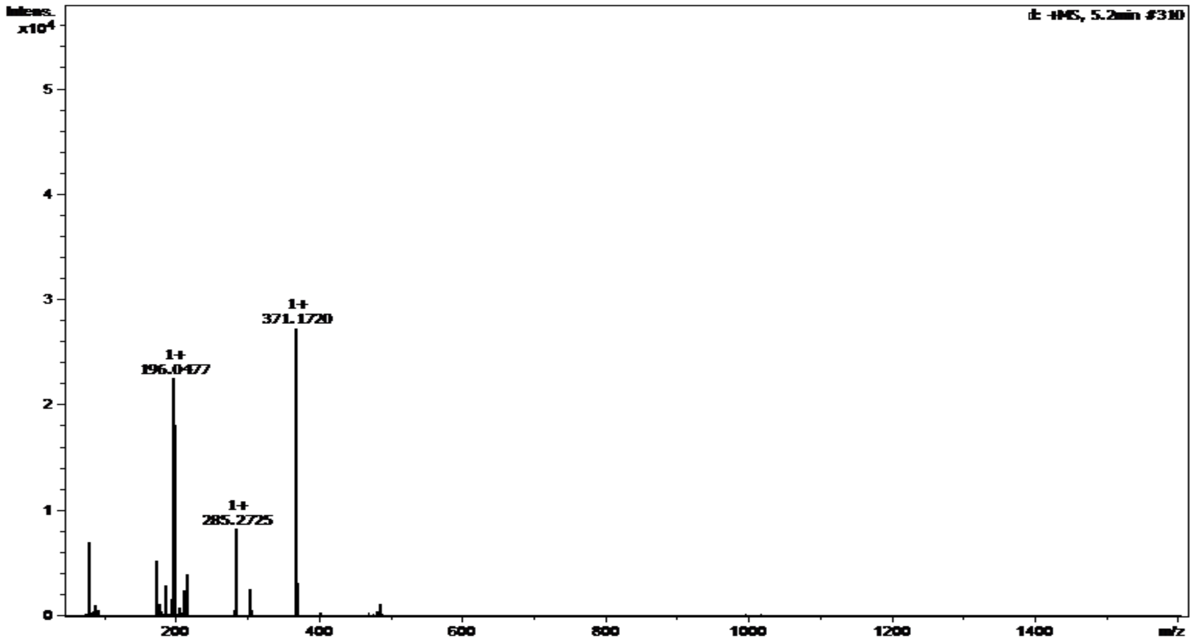

^{1}H NMR spectrum

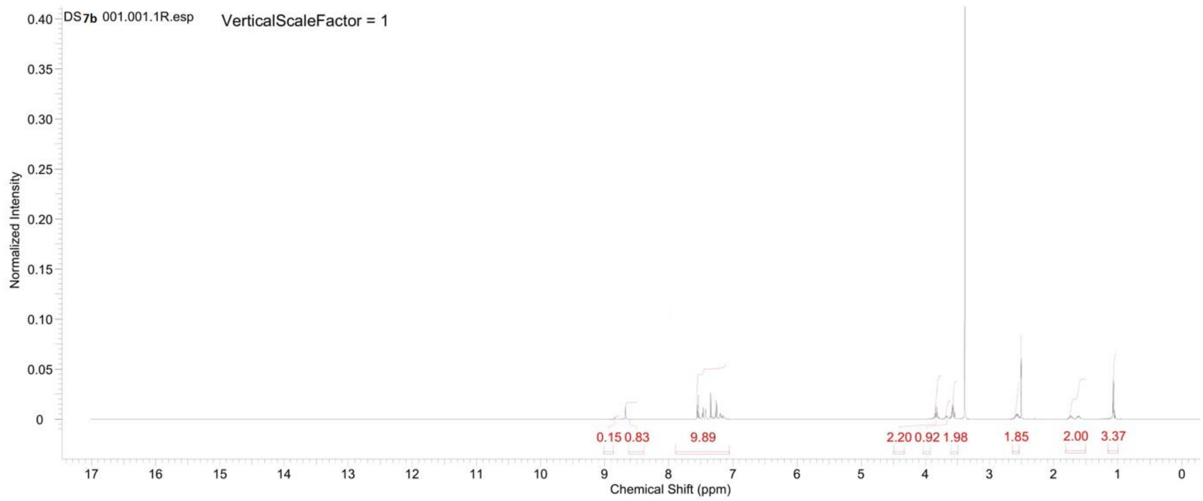

Compound 7c

^{13}C NMR spectrum

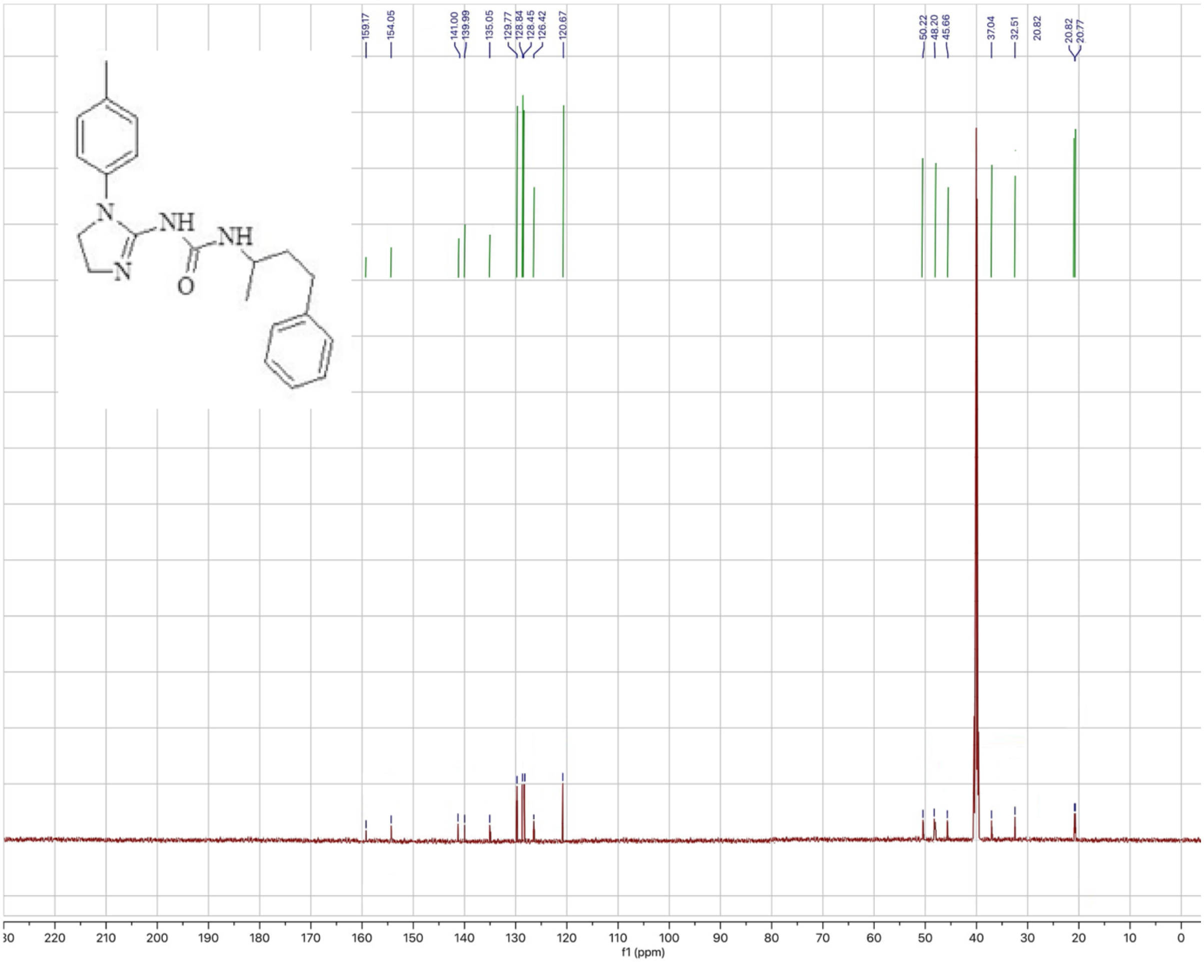

MS spectrum: $C_{21}H_{26}N_4O$ (m.m. calc. 351.2179). HRMS (ESI) *m/z* $[M+H]^+$: 351.2234.

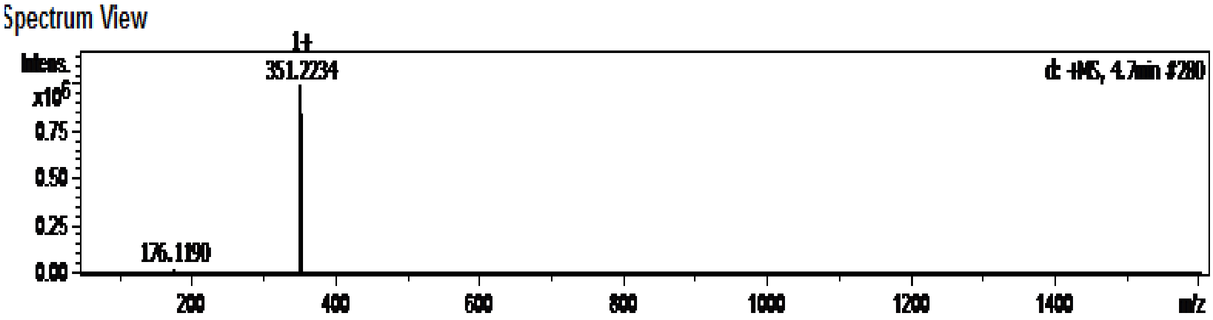

^{1}H NMR spectrum

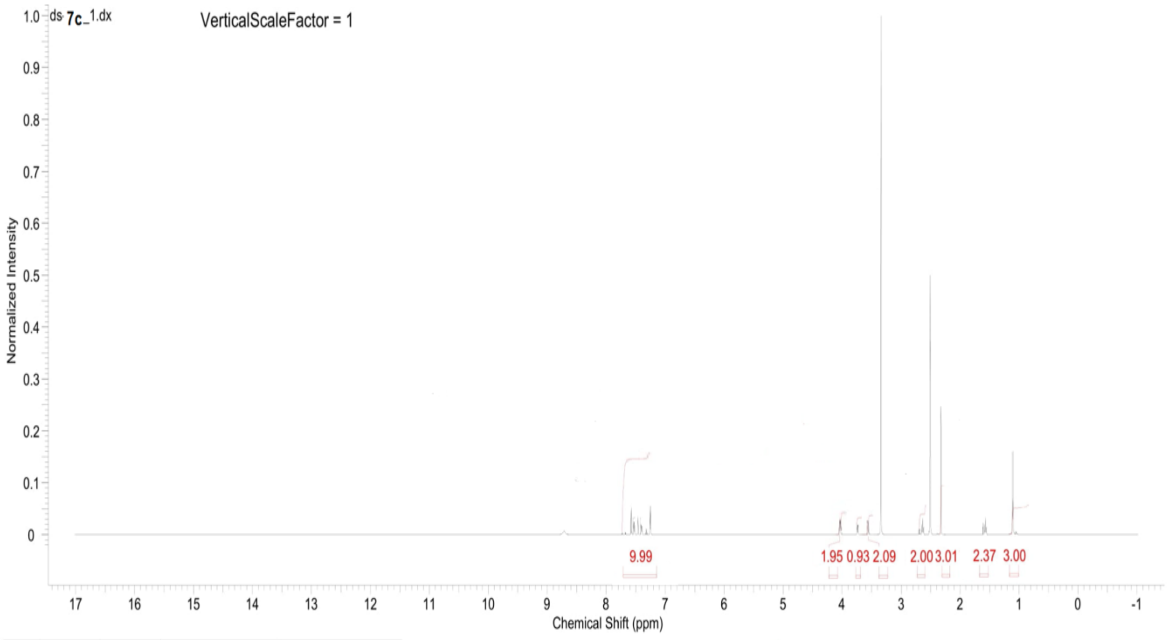

Compound 7d

^{13}C NMR spectrum

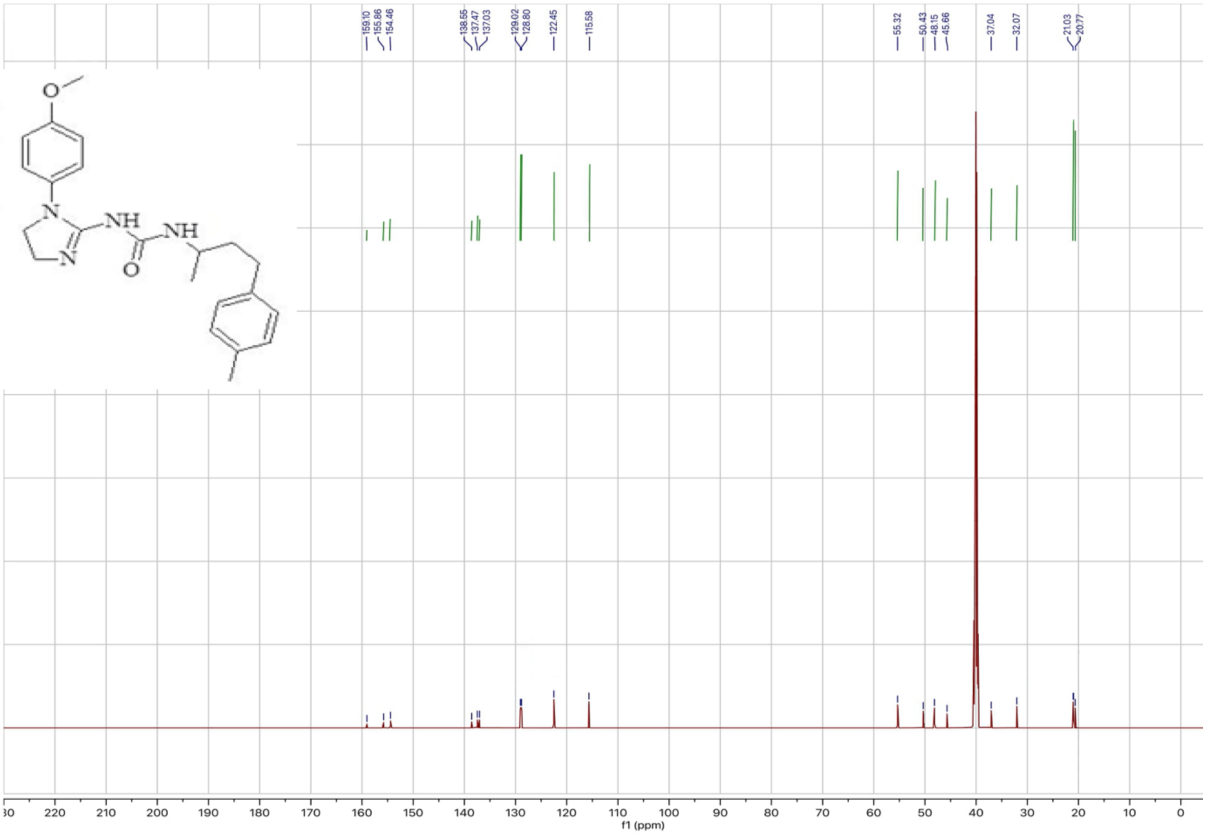

MS spectrum: $C_{22}H_{28}N_4O_2$ (m.m. calc. 381.2285). HRMS (ESI) *m/z* $[M+H]^+$: 381.1758.

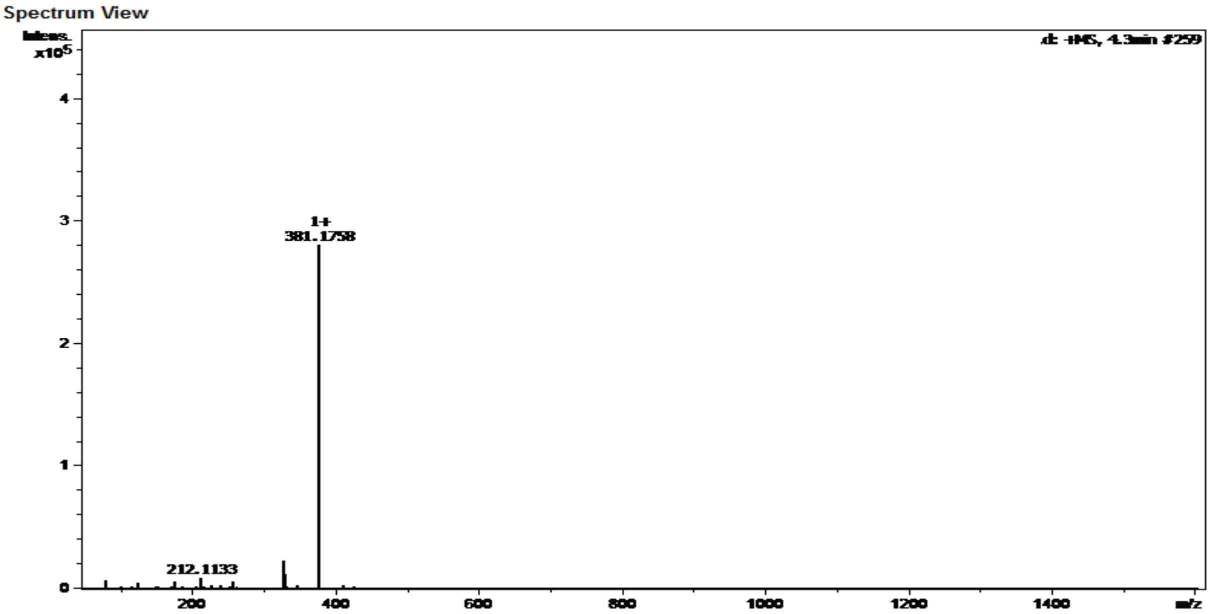

^{1}H NMR spectrum

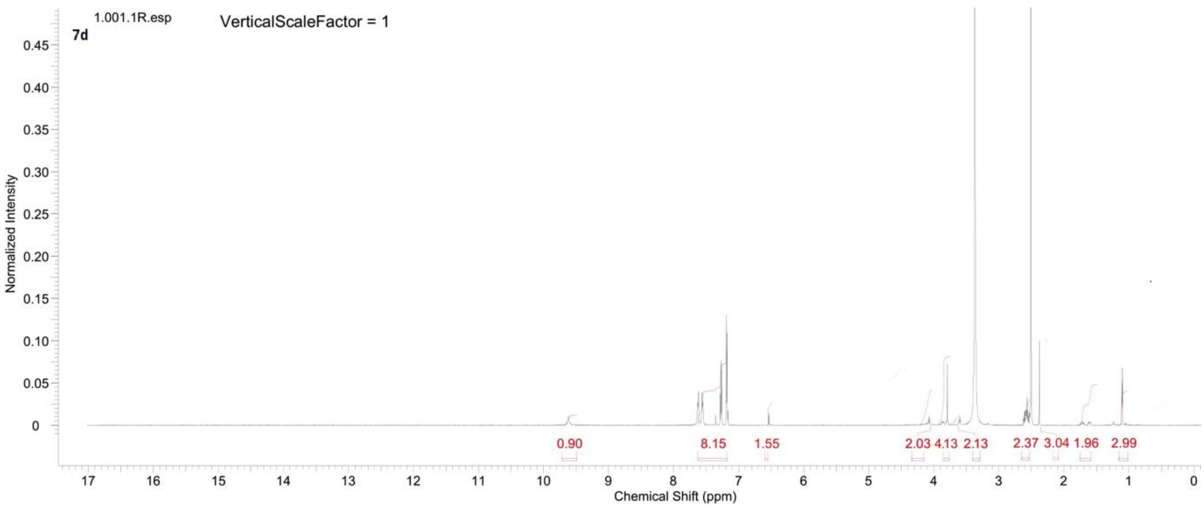

Compound 7e

^{13}C NMR spectrum

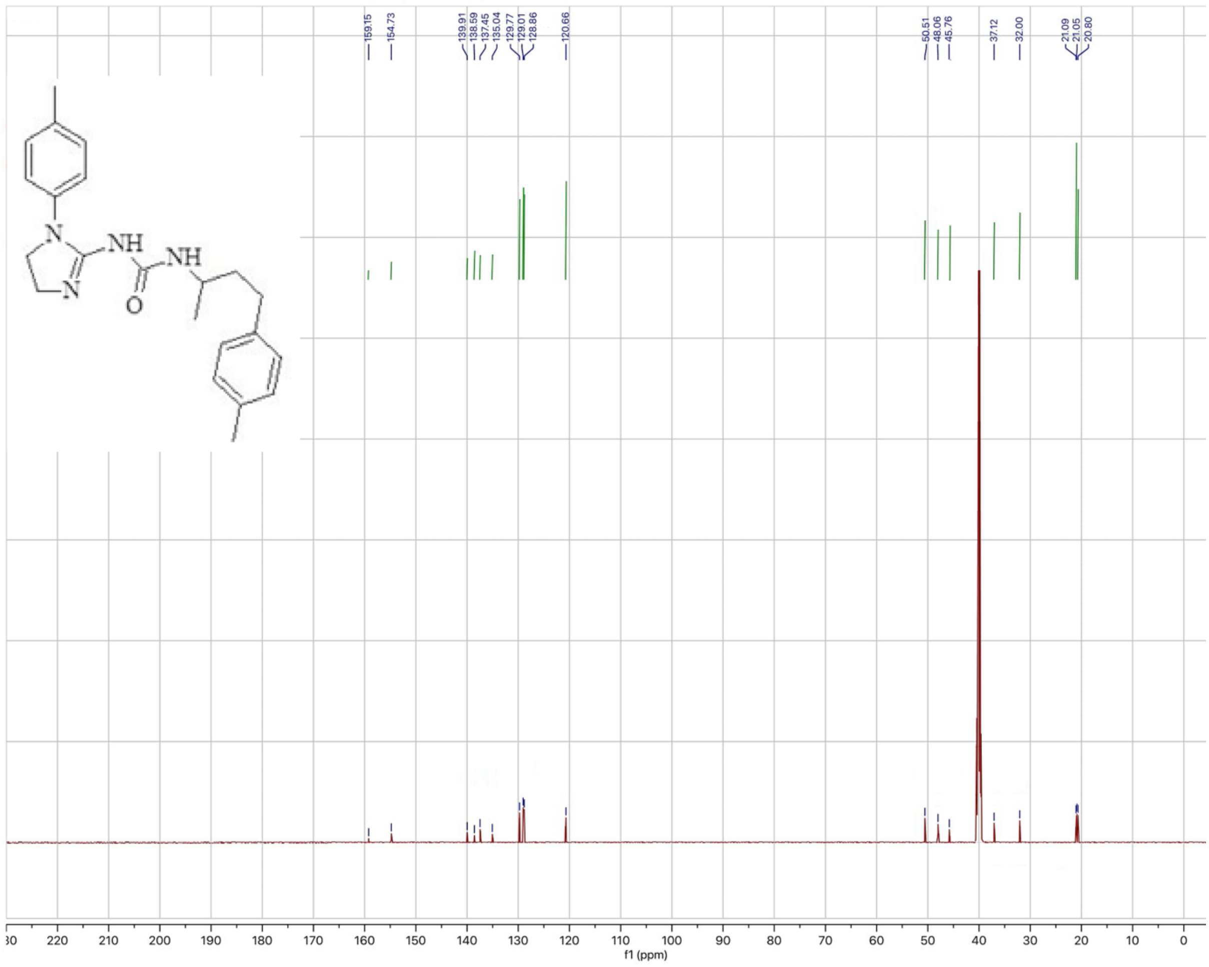

MS spectrum: $C_{22}H_{28}N_4O$ (m.m. calc. 365.2336). HRMS (ESI) *m/z* $[M+H]^+$: 365.2142.

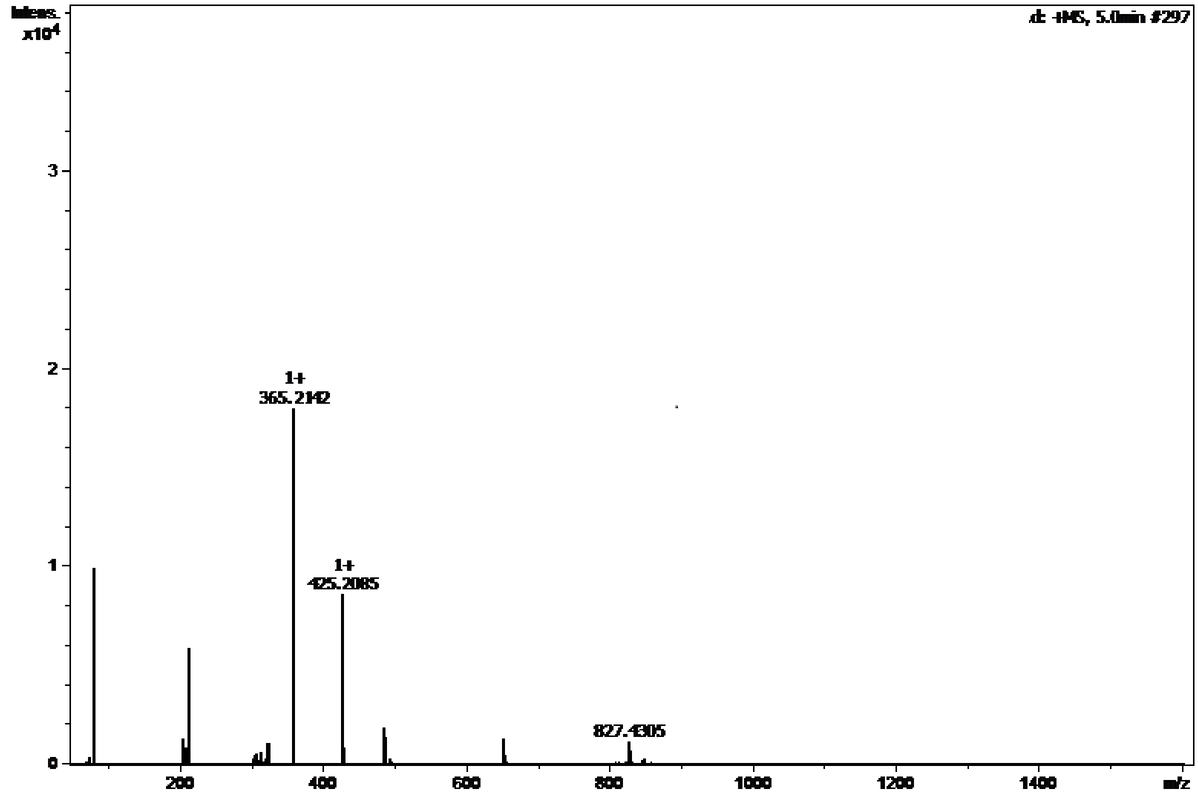

^1H NMR spectrum

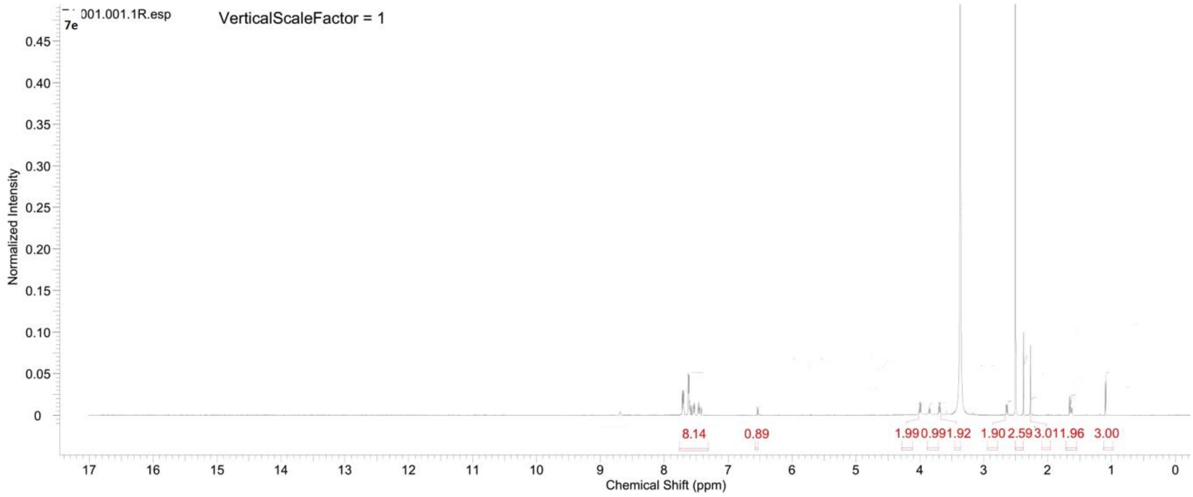

Compound 7f

^{13}C NMR spectrum

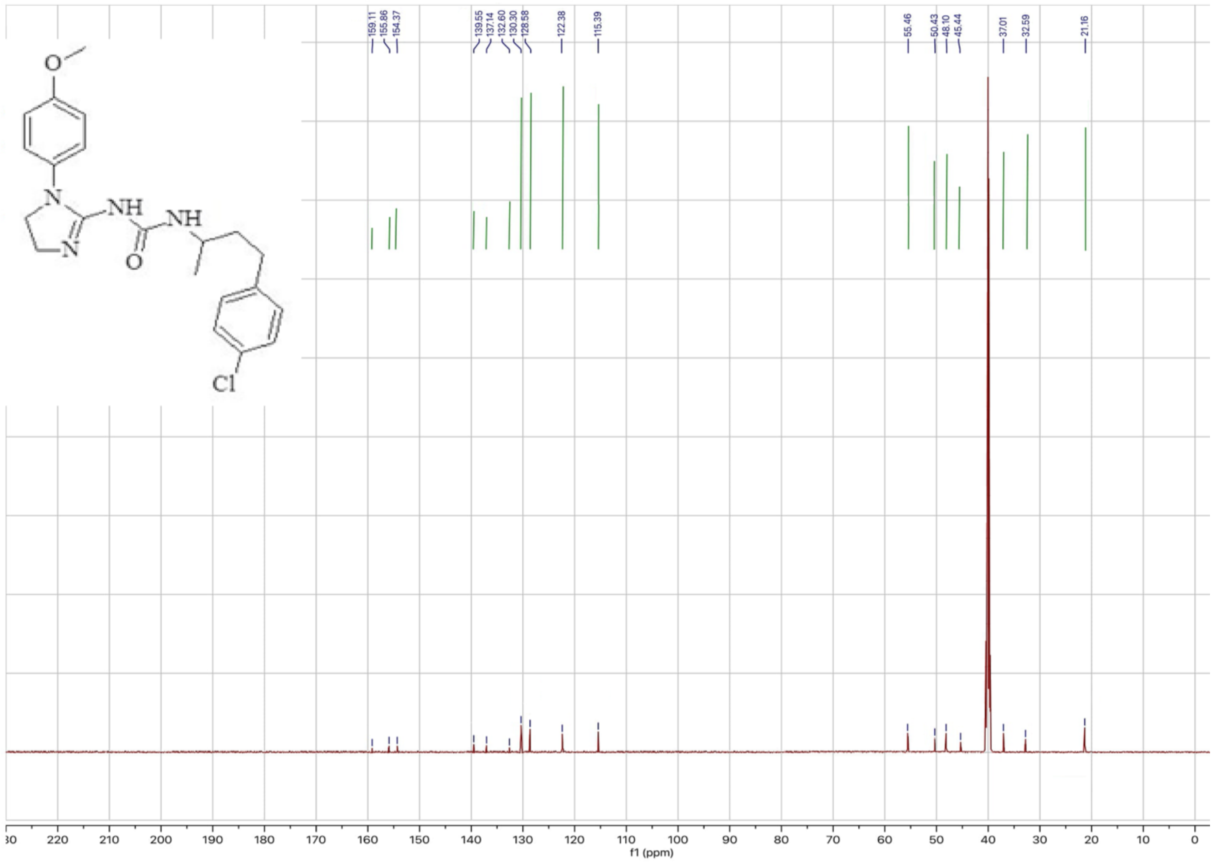

MS spectrum: $C_{21}H_{25}ClN_4O_2$ (m.m. calc. 401.1739). HRMS (ESI) *m/z* [M+H]$^+$: 401.1365.

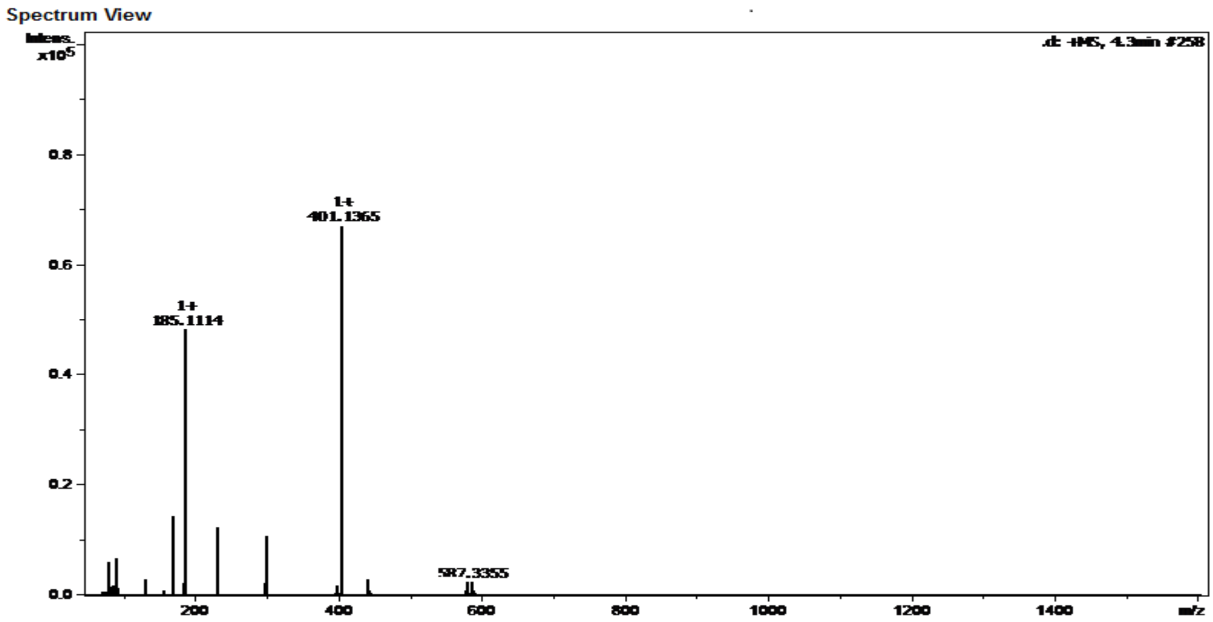

^{1}H NMR spectrum

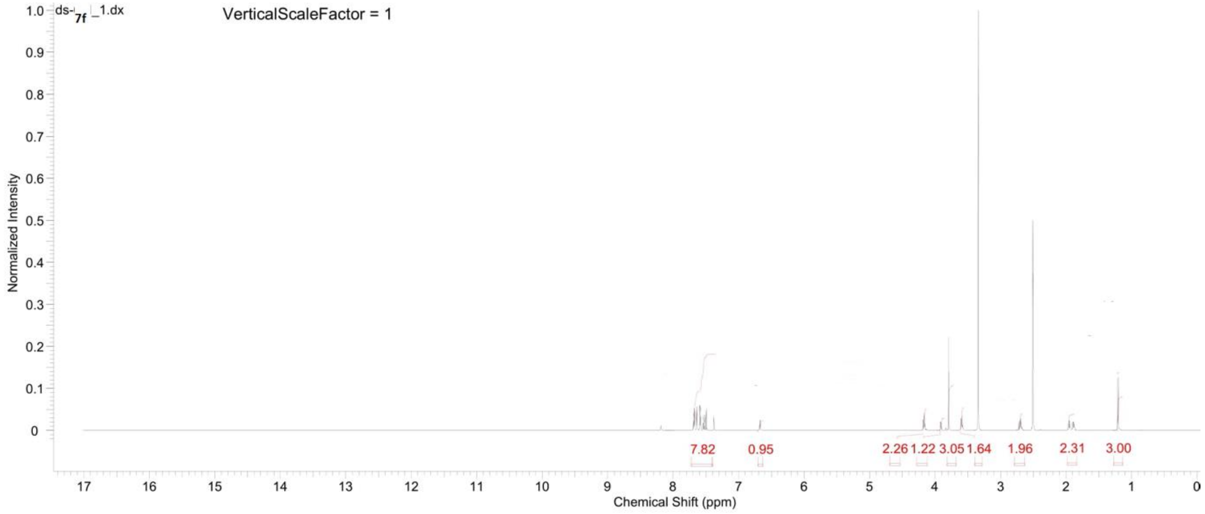

Compound 7g

^{13}C NMR spectrum

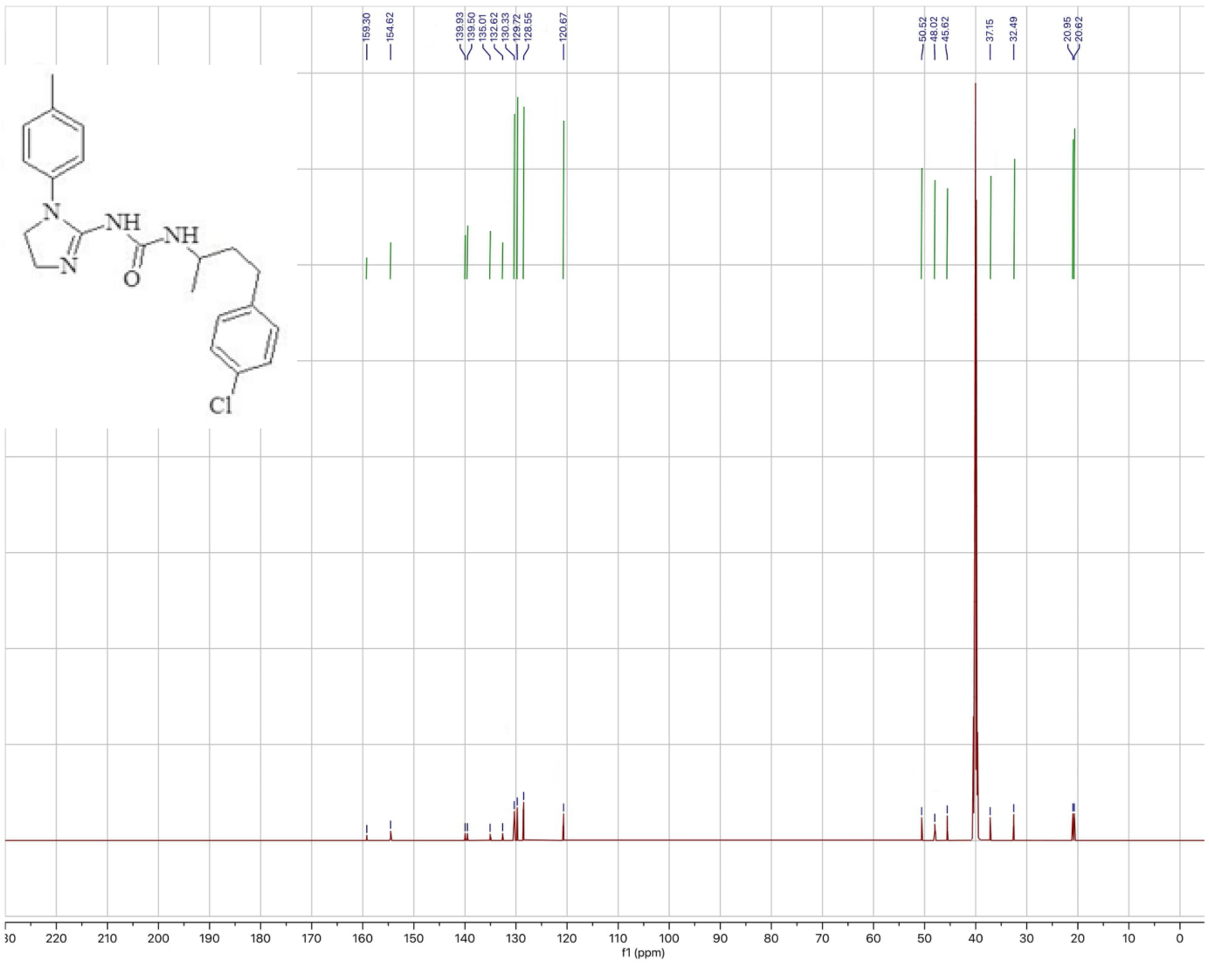

MS spectrum: $C_{21}H_{25}ClN_4O$ (m.m. calc. 385.1790). HRMS (ESI) *m/z* $[M+H]^+$: 385.1604.

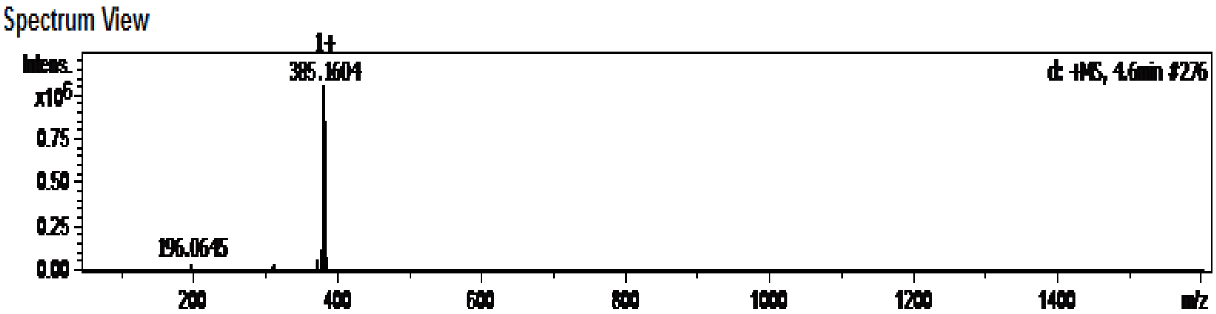

^{1}H NMR spectrum

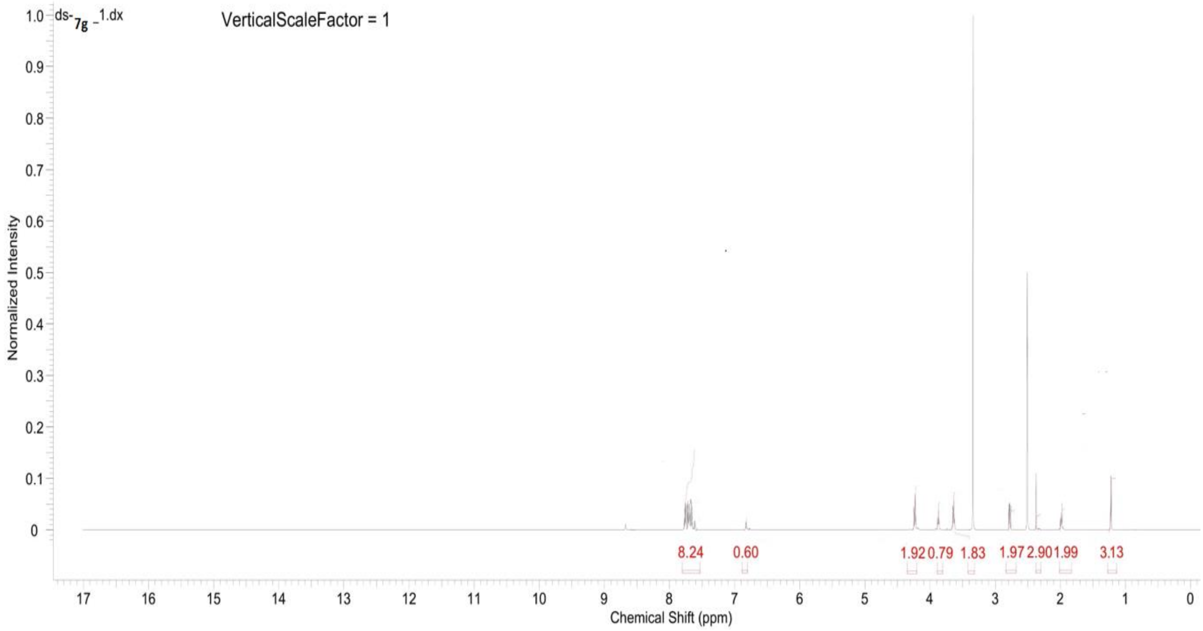

Compound 7h

^{13}C NMR spectrum

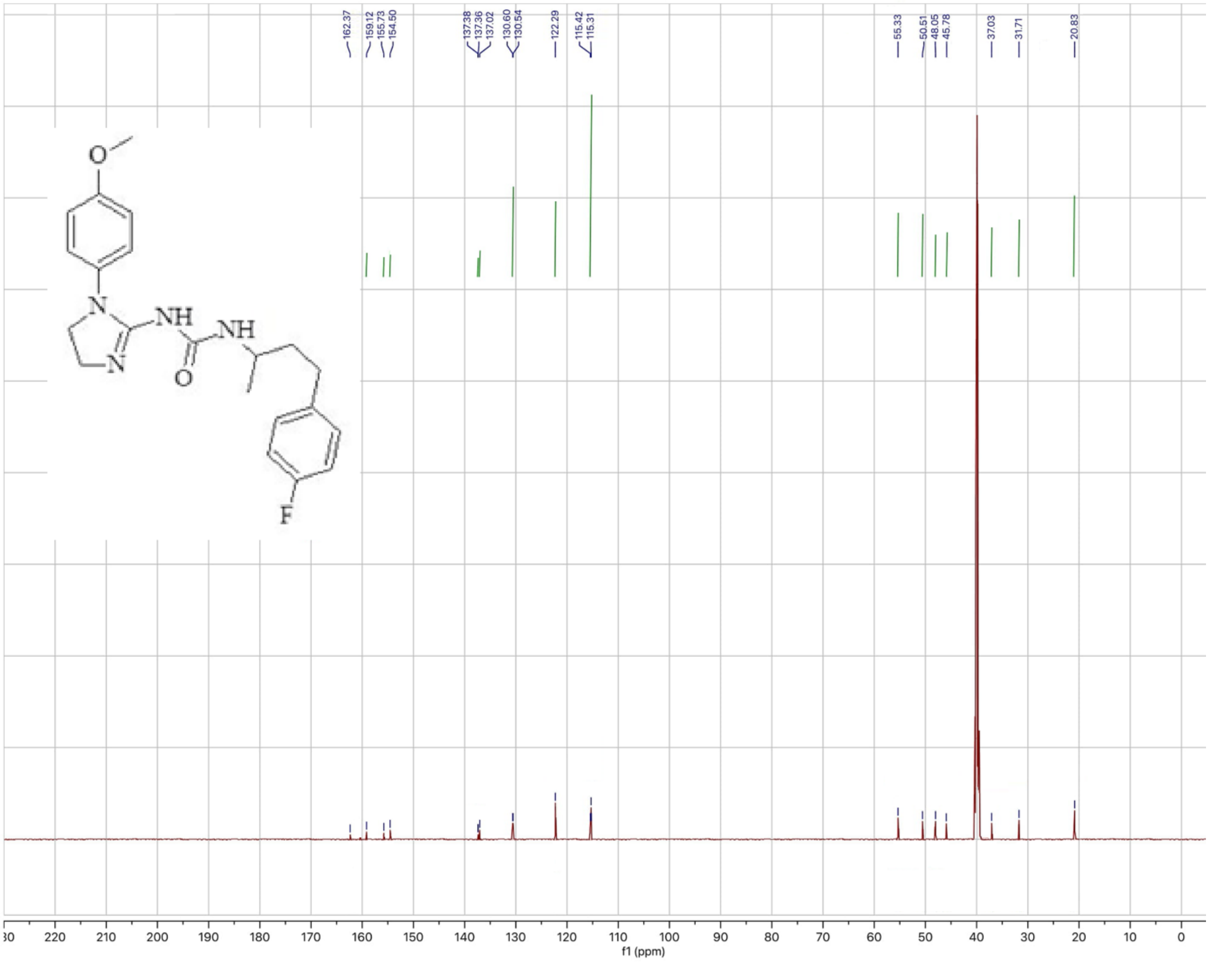

MS spectrum: $C_{21}H_{25}FN_4O_2$ (m.m. calc. 385.2034). HRMS (ESI) *m/z* $[M+H]^+$: 385.2501.

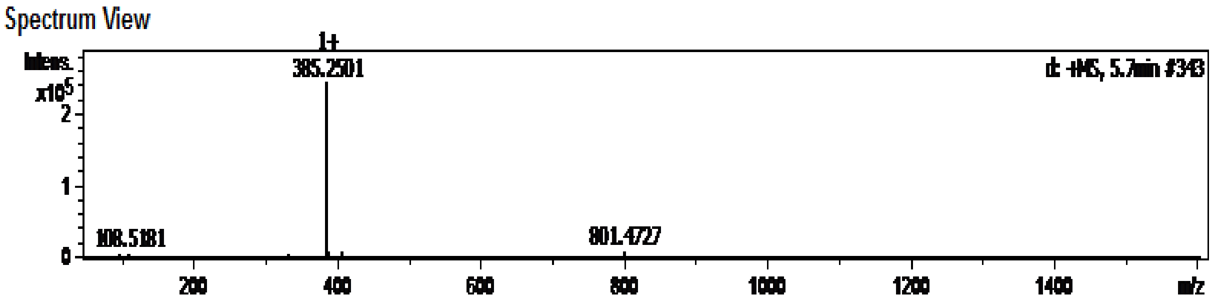

^{1}H NMR spectrum

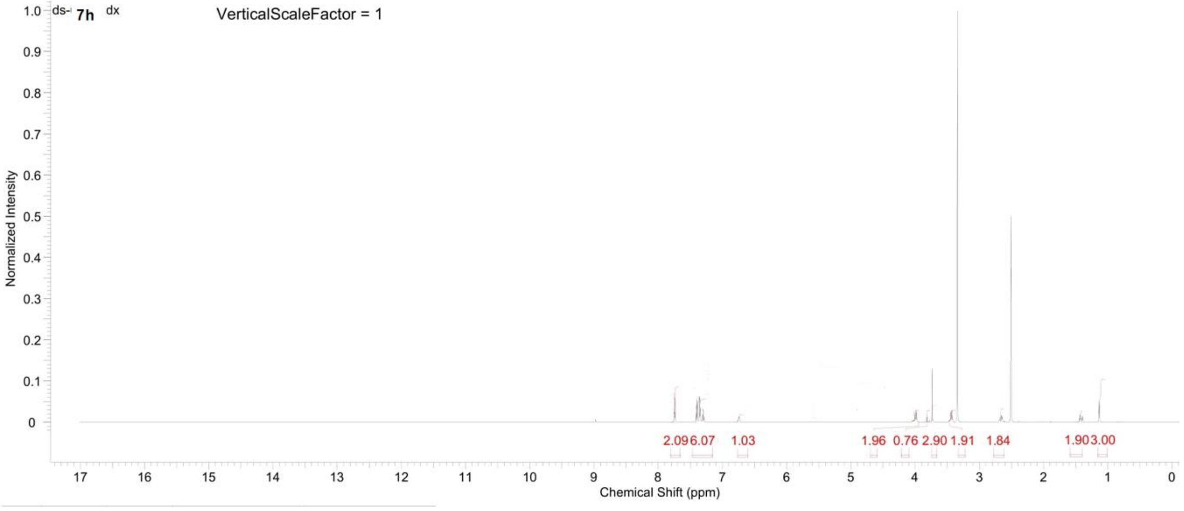

Compound 7i

^{13}C NMR spectrum

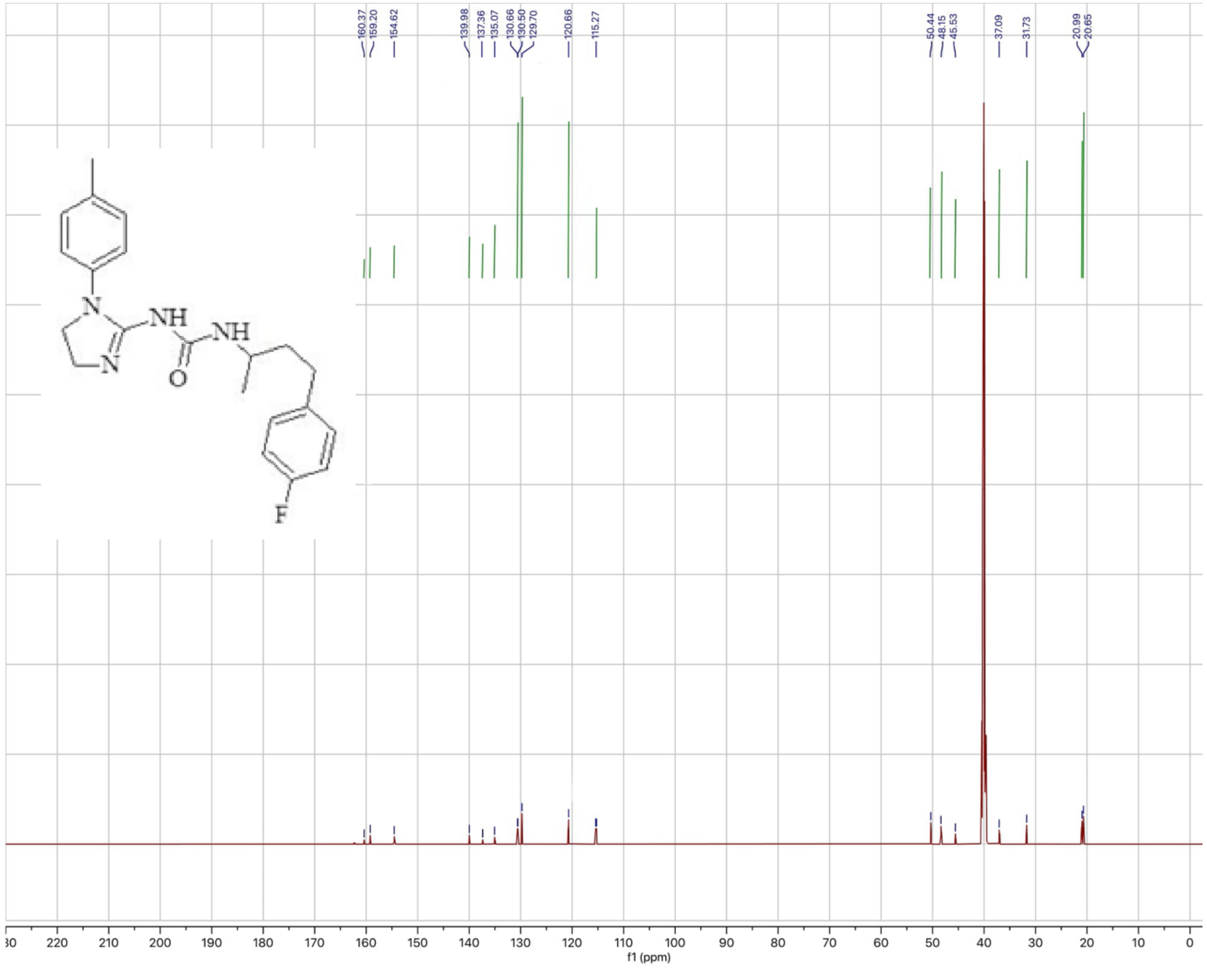

MS spectrum: $C_{21}H_{25}FN_4O$ (m.m. calc. 369.2085). HRMS (ESI) *m/z* $[M+H]^+$: 369.2069.

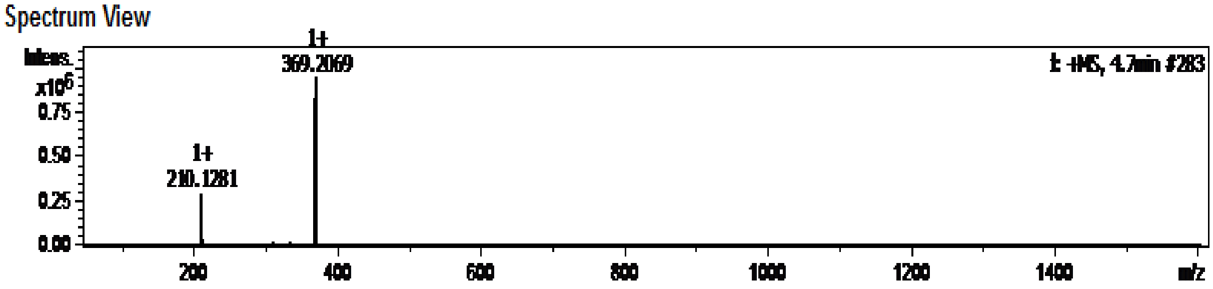

^{1}H NMR spectrum

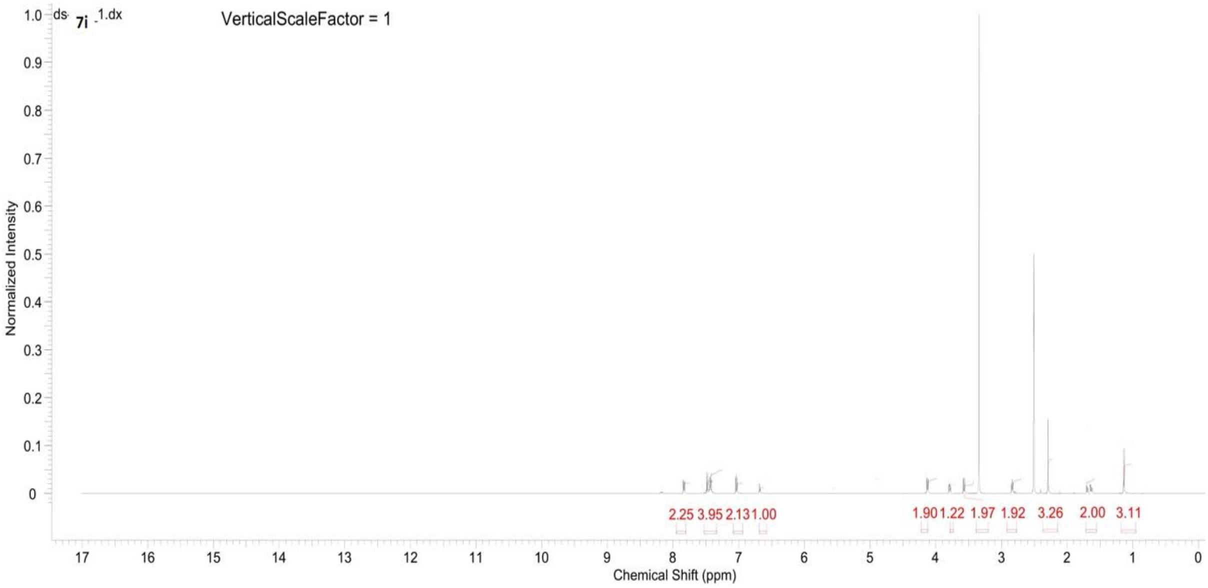

Compound 7j

^{13}C NMR spectrum

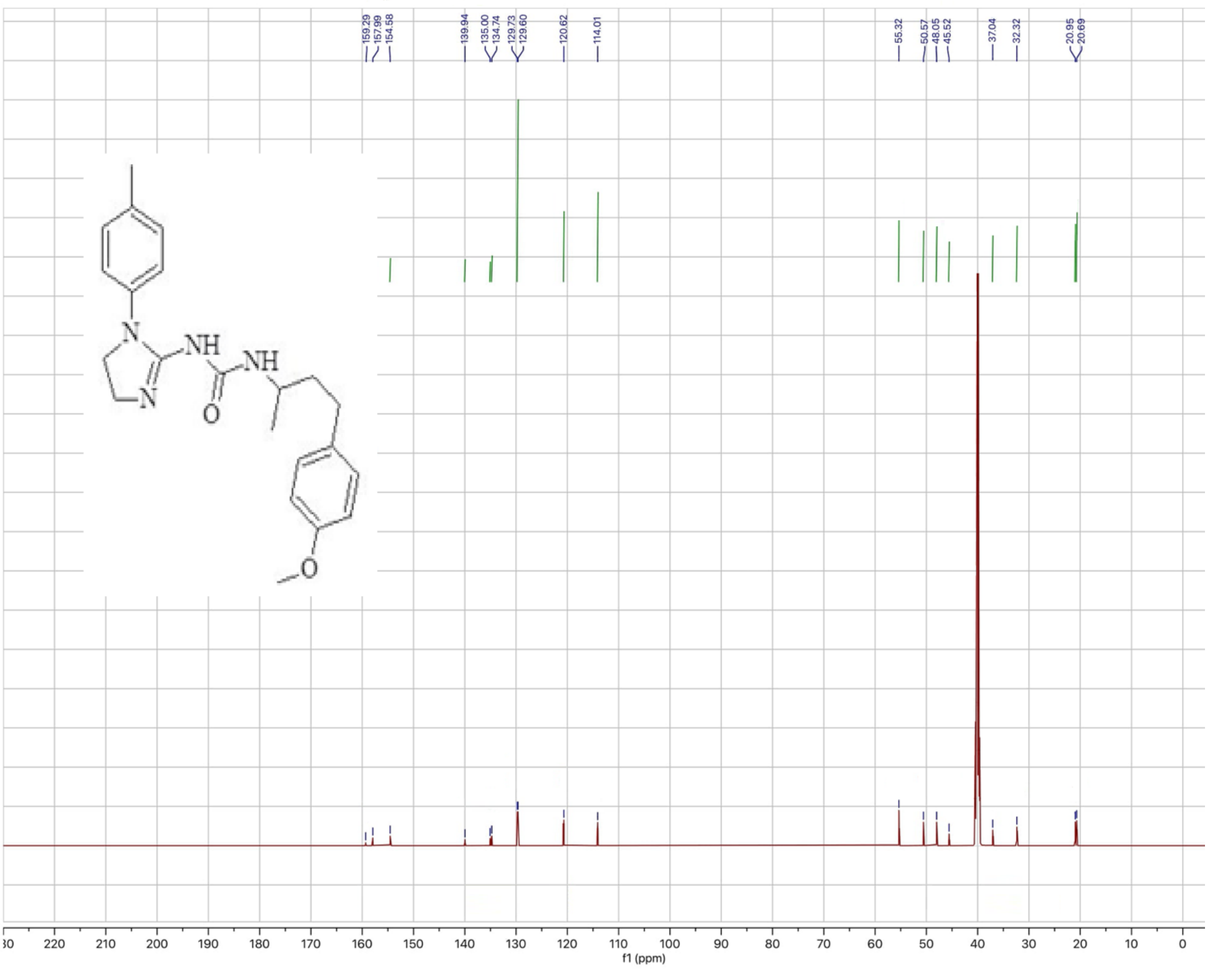

MS spectrum: $C_{22}H_{28}N_4O_2$ (m.m. calc. 381.2285). HRMS (ESI) *m/z* $[M+H]^+$: 381.234.

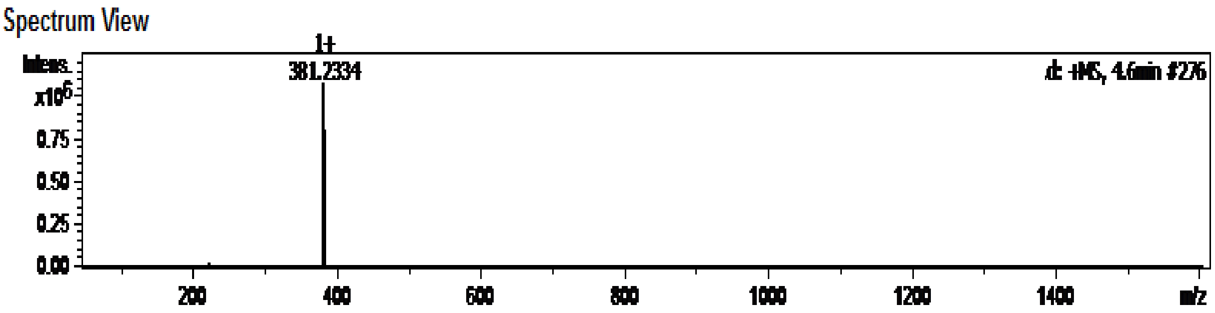

^{1}H NMR spectrum

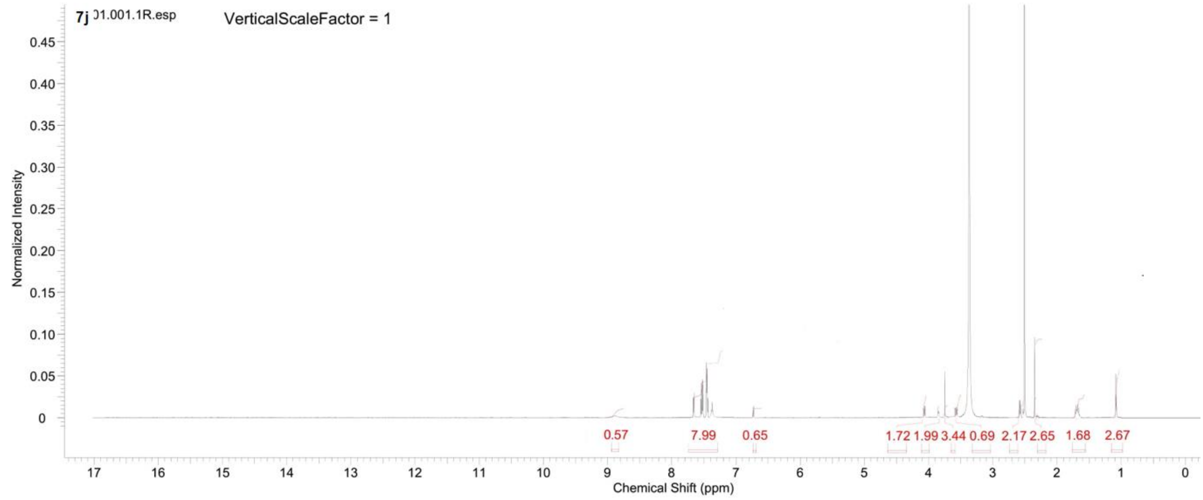

Compound 7k

^{13}C NMR spectrum

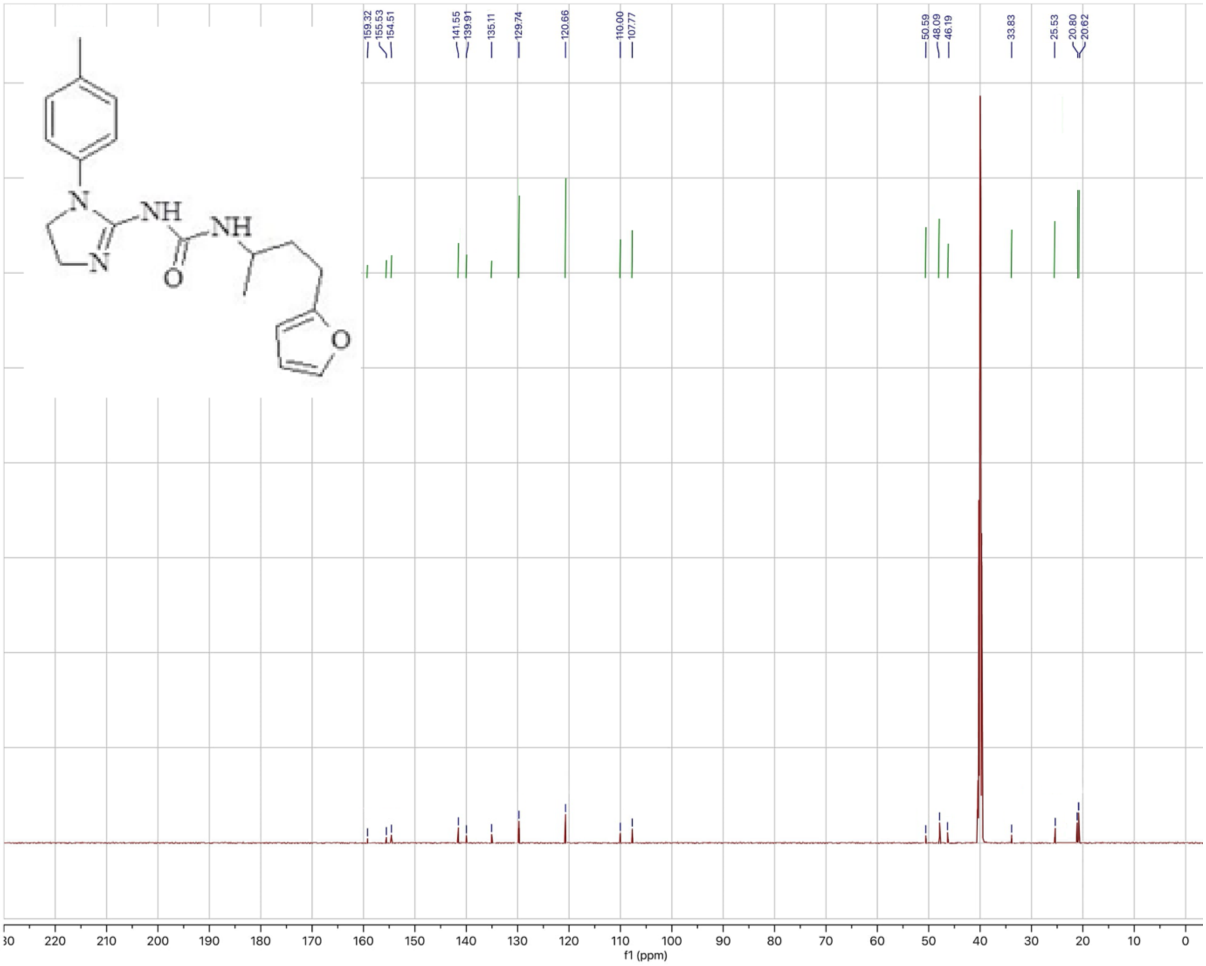

MS spectrum: $C_{19}H_{24}N_4O_2$ (m.m. calc. 341.1972). HRMS (ESI) *m/z* $[M+H]^+$: 341.2591.

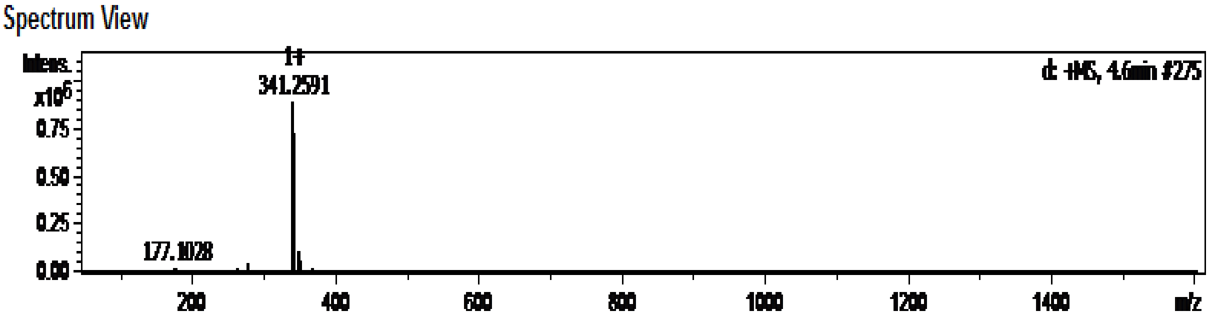

^{1}H NMR spectrum

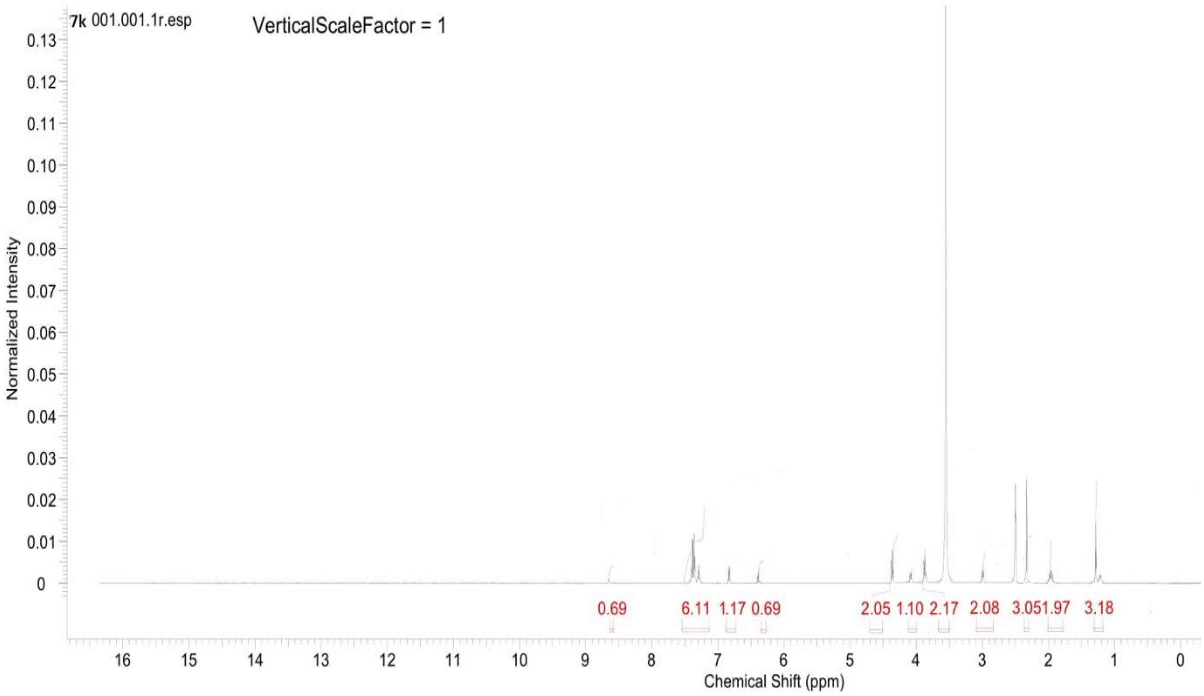

Compound 7l

^{13}C NMR spectrum

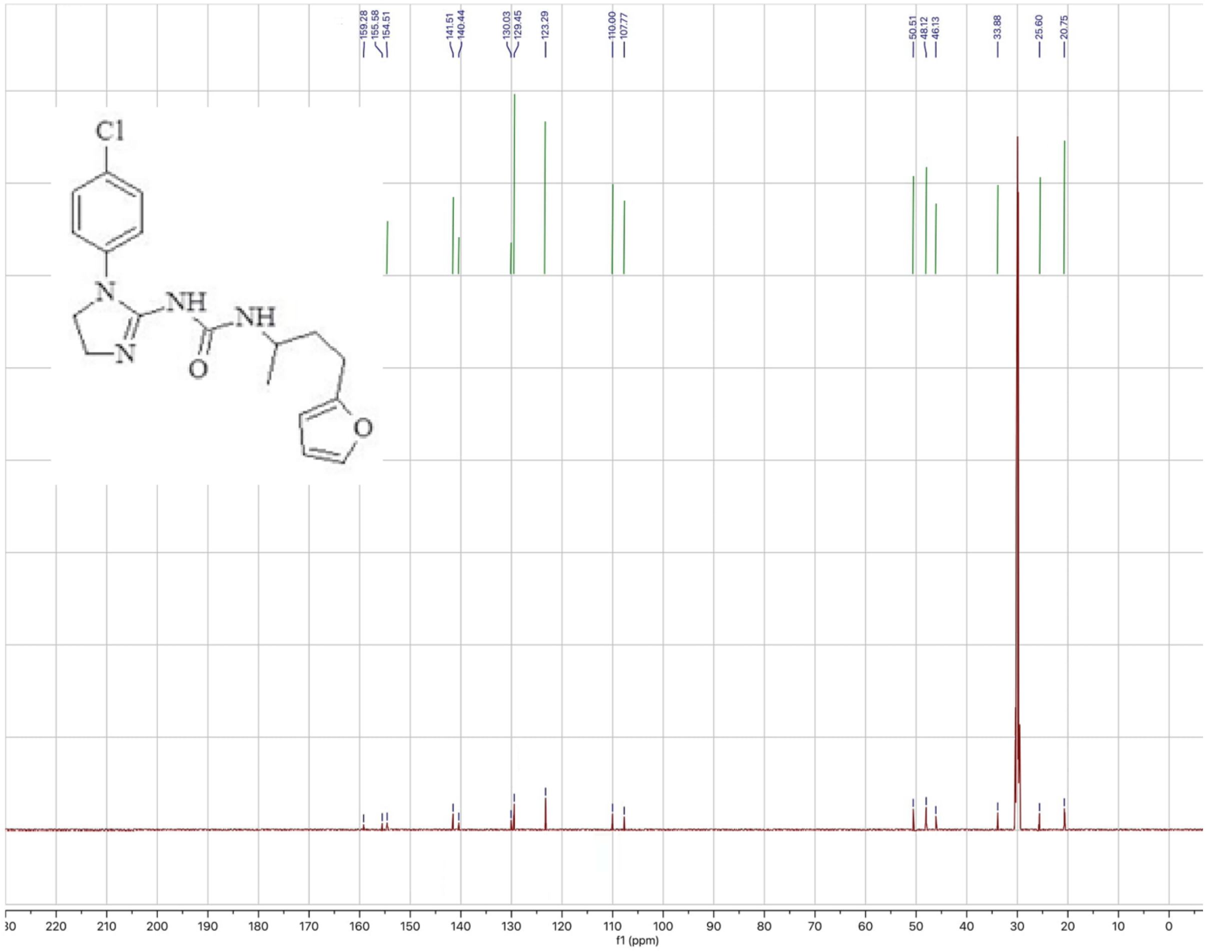

MS spectrum: $C_{18}H_{21}ClN_4O_2$ (m.m. calc. 361.1426). HRMS (ESI) *m/z* [M+H]$^+$: 361.2080.

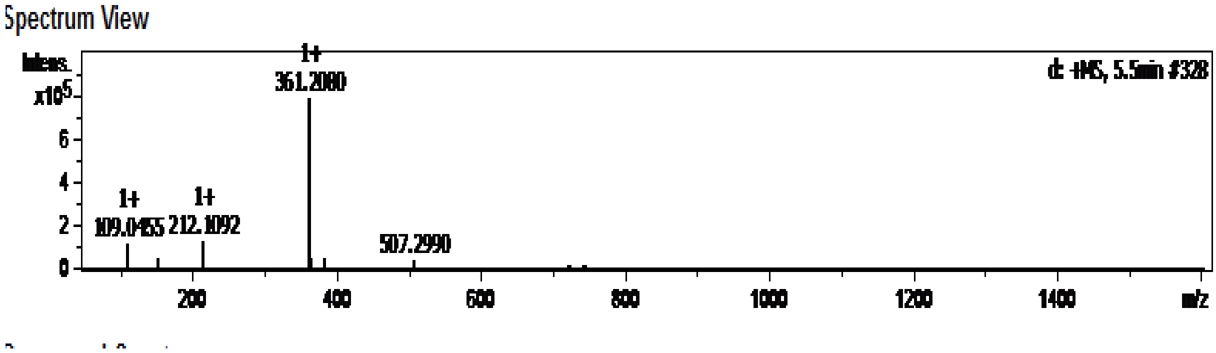

^{1}H NMR spectrum

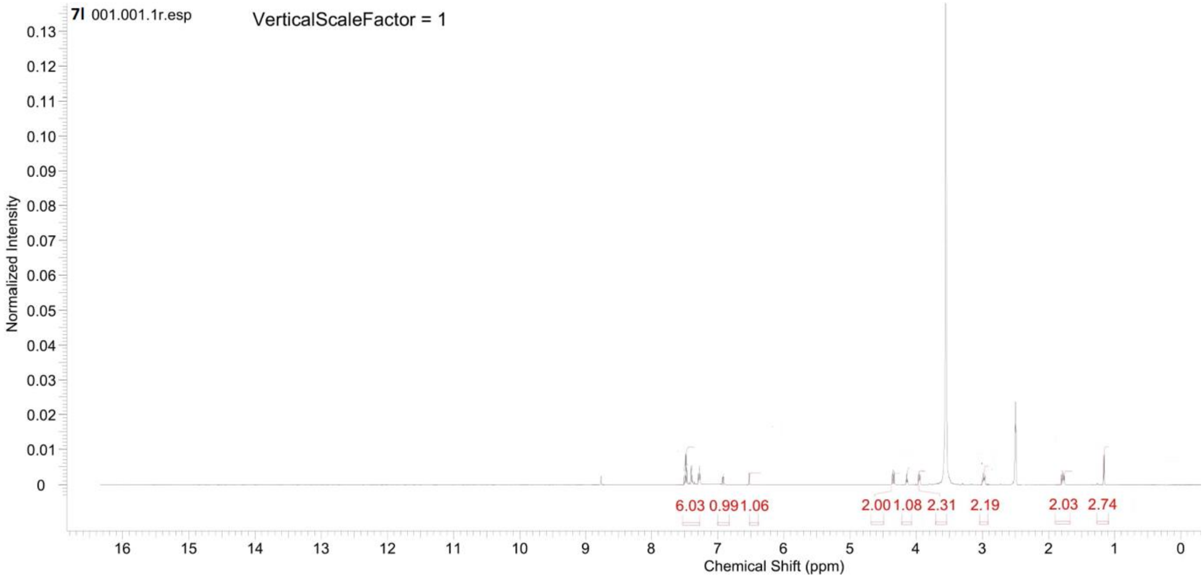

Compound 7m

^{13}C NMR spectrum

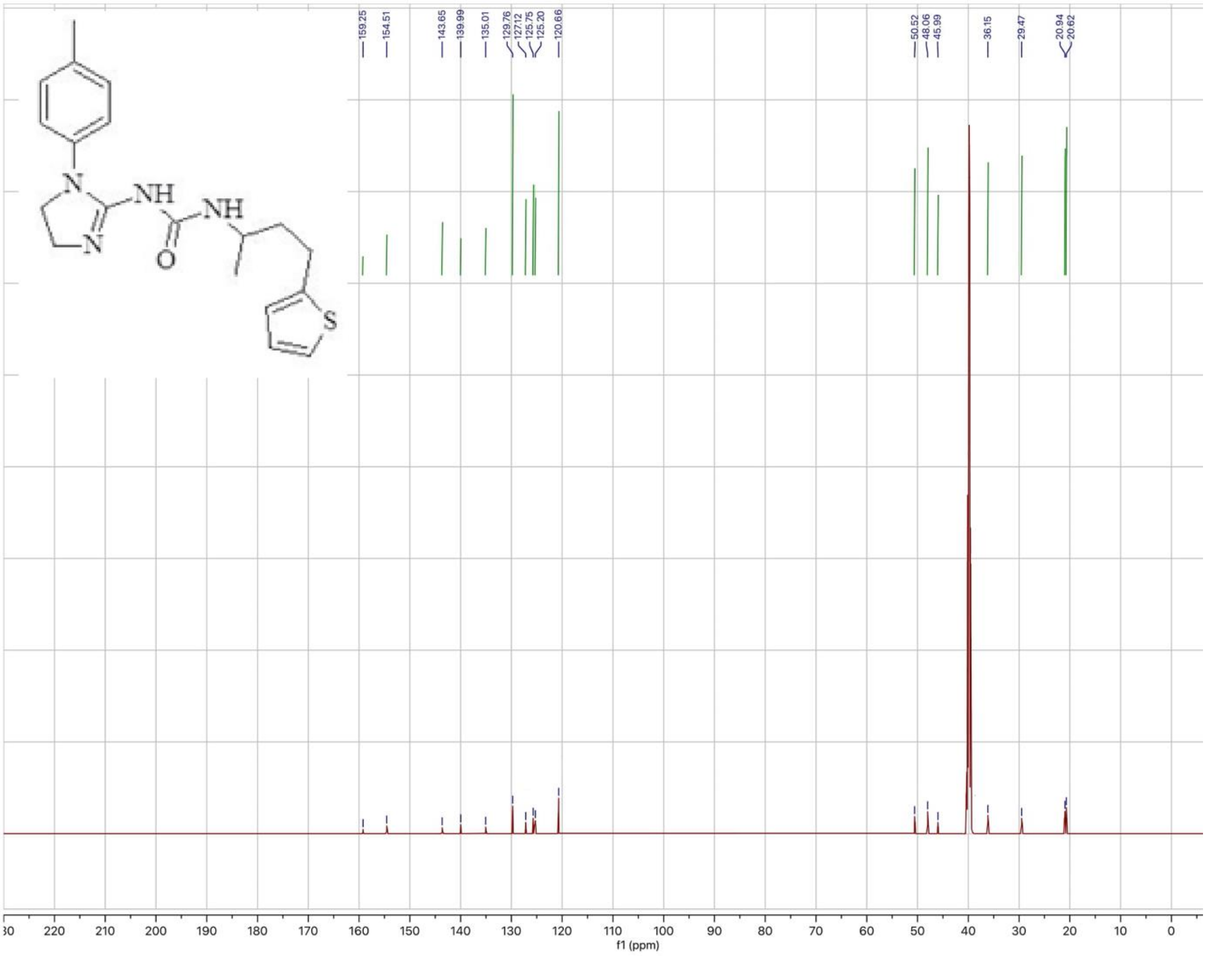

MS spectrum: $C_{19}H_{28}N_4OS$ (m.m. calc. 357.1744). HRMS (ESI) *m/z* [M+H]$^+$: 357.1744.

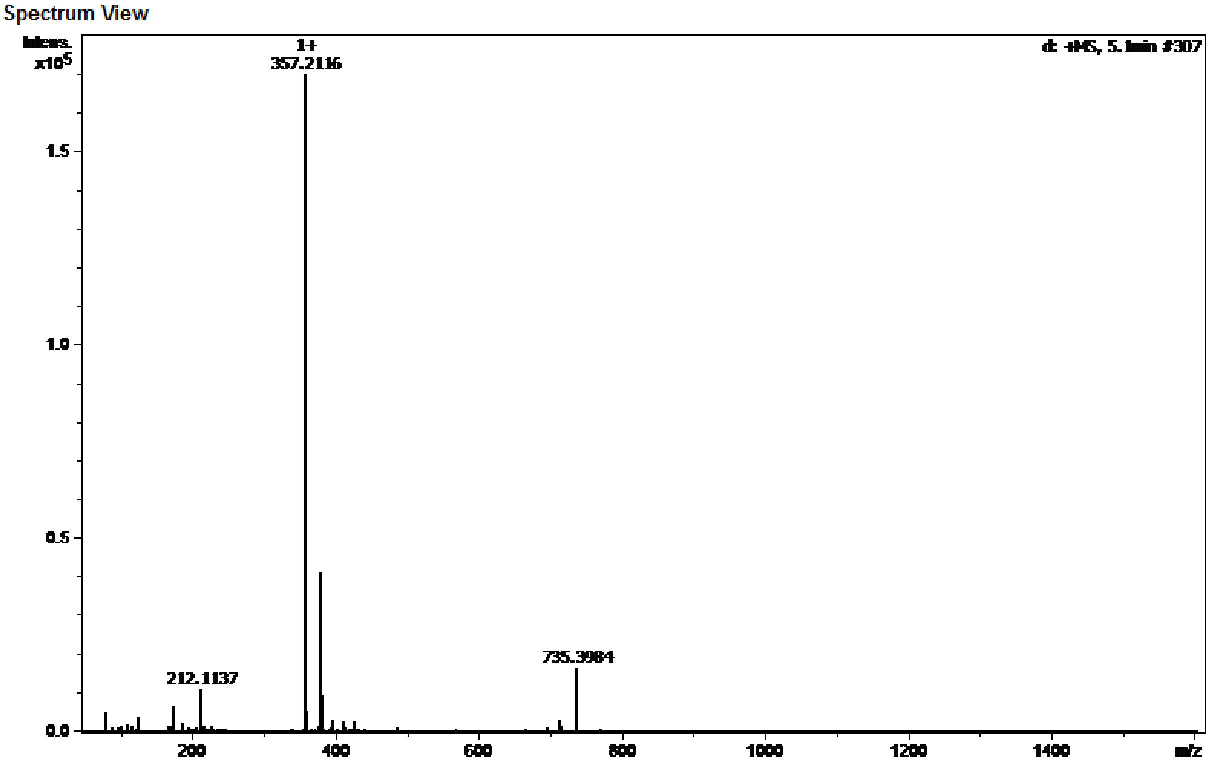

^{1}H NMR spectrum

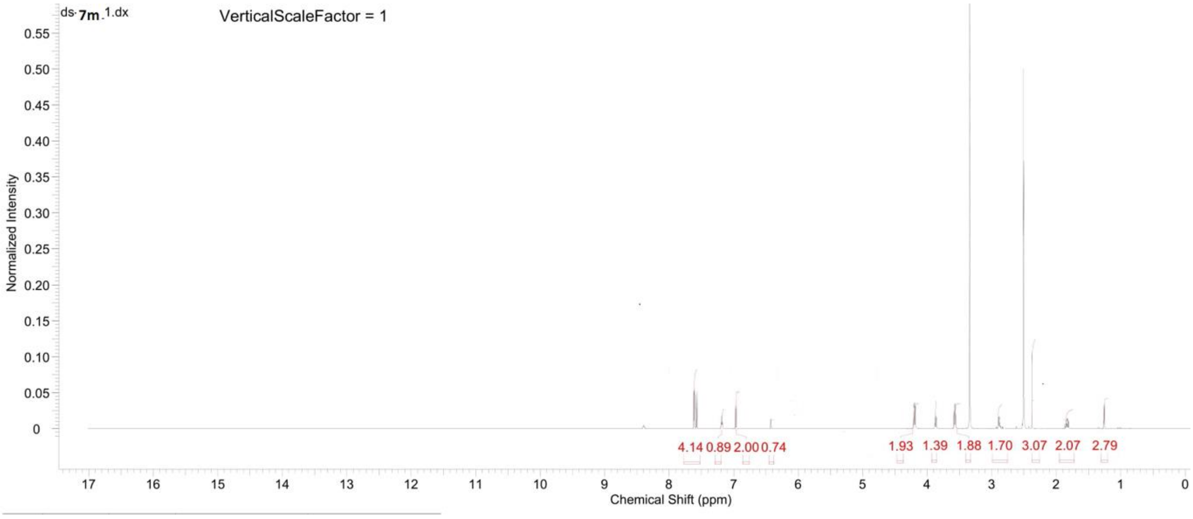

Compound 7n

^{13}C NMR spectrum

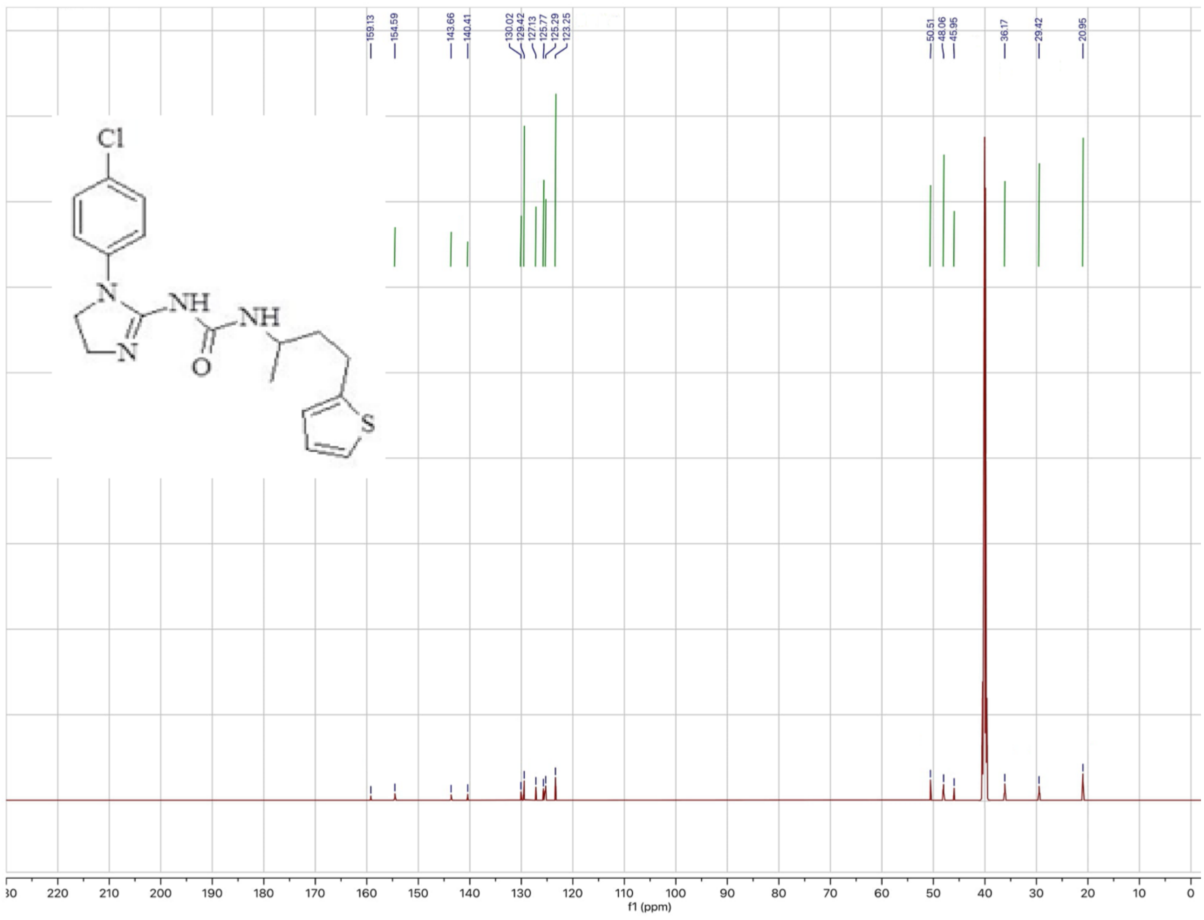

MS spectrum: $C_{18}H_{21}ClN_4OS$ (m.m. calc. 377.1197). HRMS (ESI) *m/z* $[M+H]^+$: 377.1122.

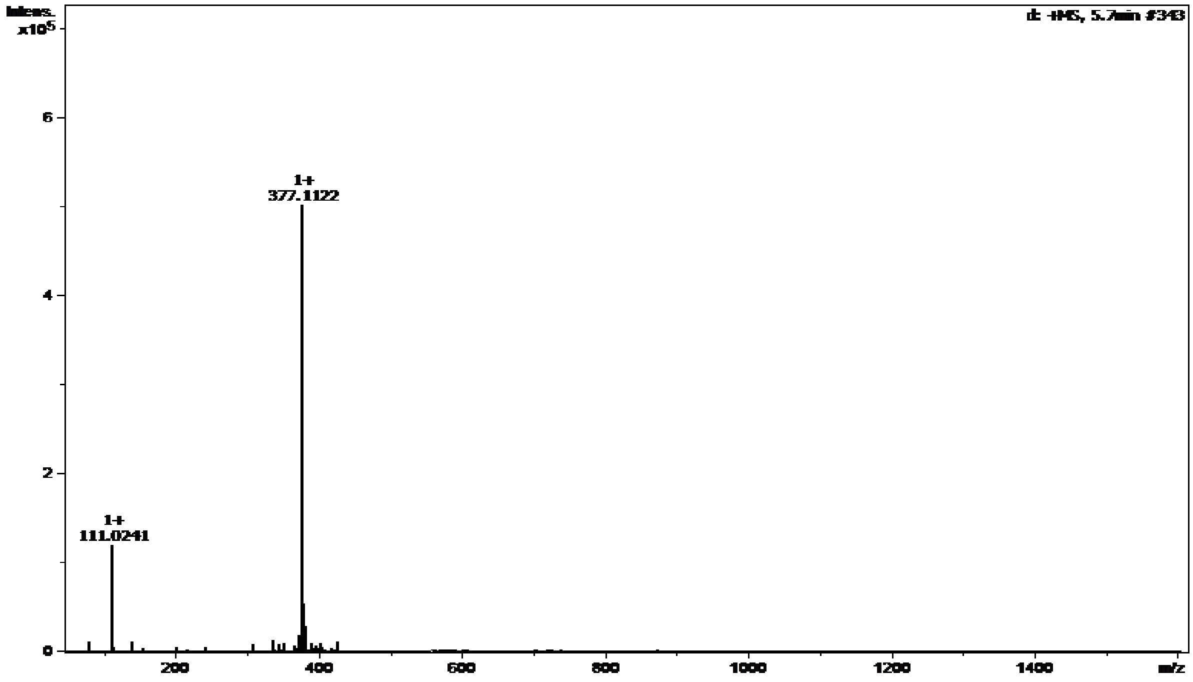

^{1}H NMR spectrum

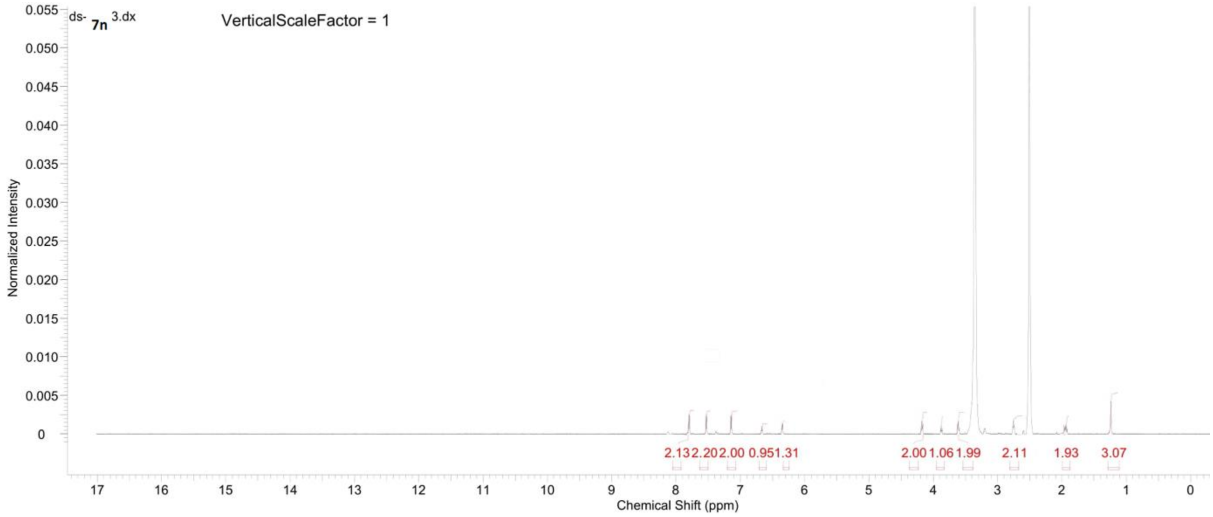

Compound 7o

^{13}C NMR spectrum

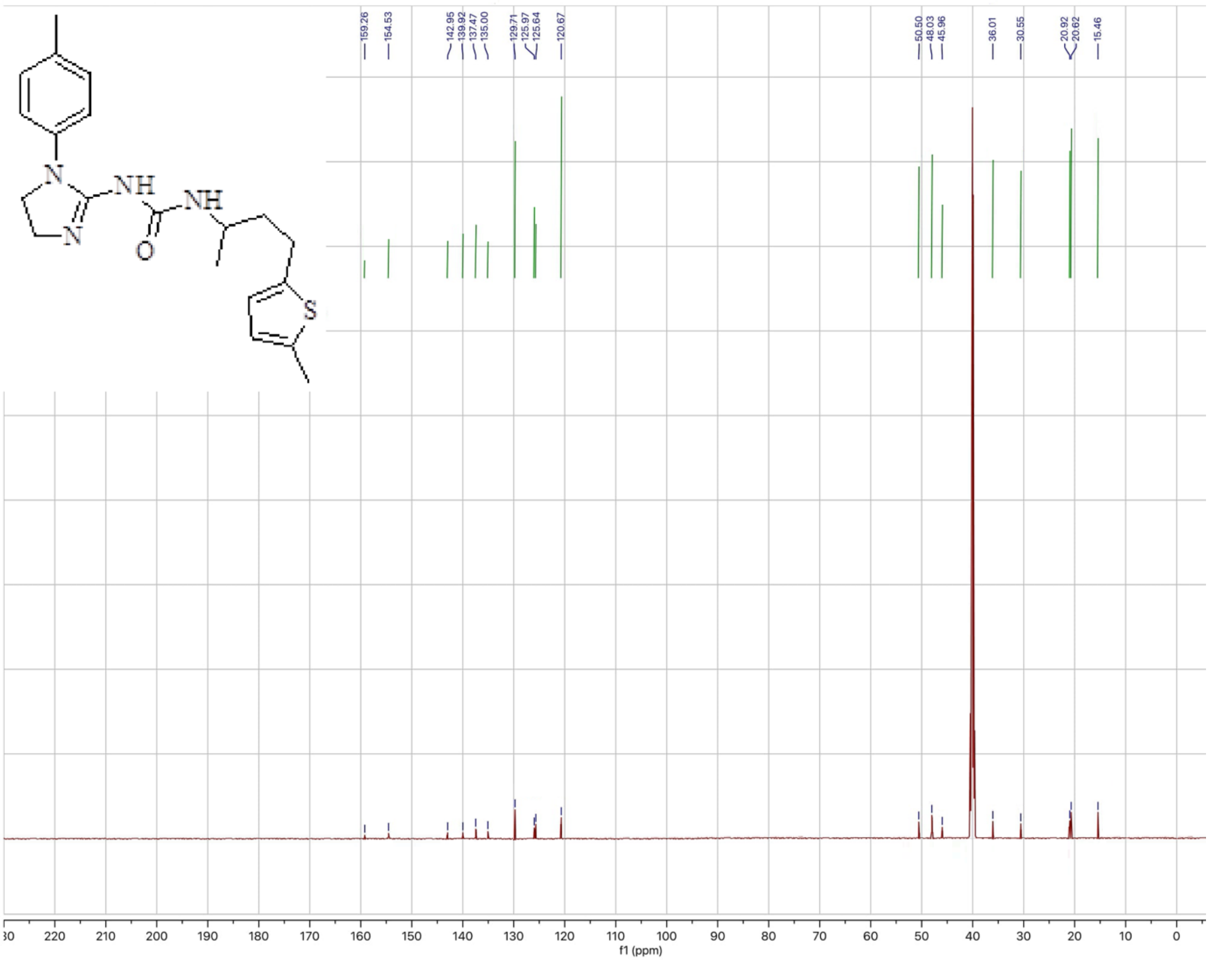

MS spectrum: $C_{20}H_{26}N_4OS$ (m.m. calc. 371.1900). HRMS (ESI) *m/z* $[M+H]^+$: 371.1632.

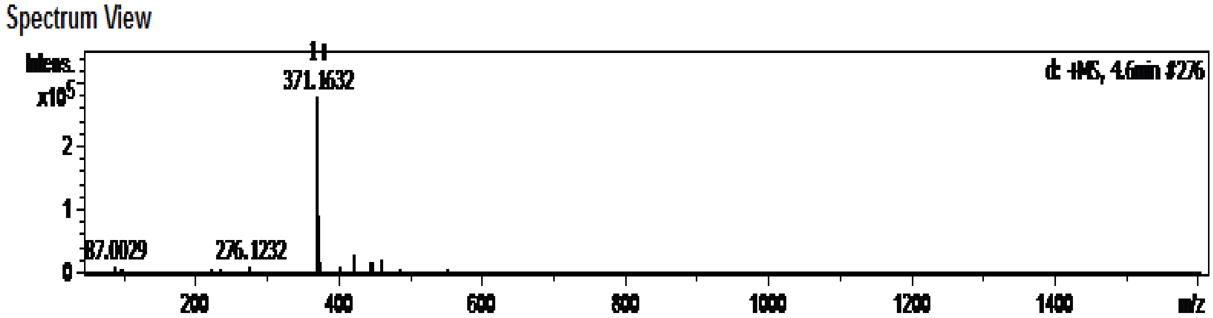

^{1}H NMR spectrum

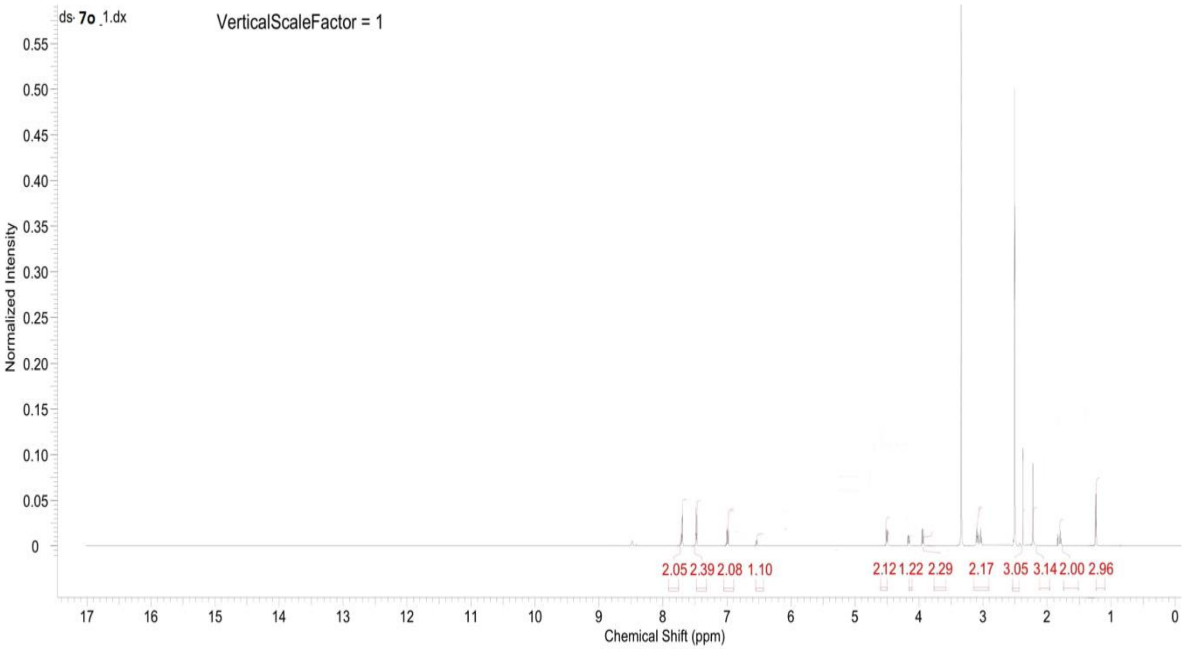

Compound 7p

^{13}C NMR spectrum

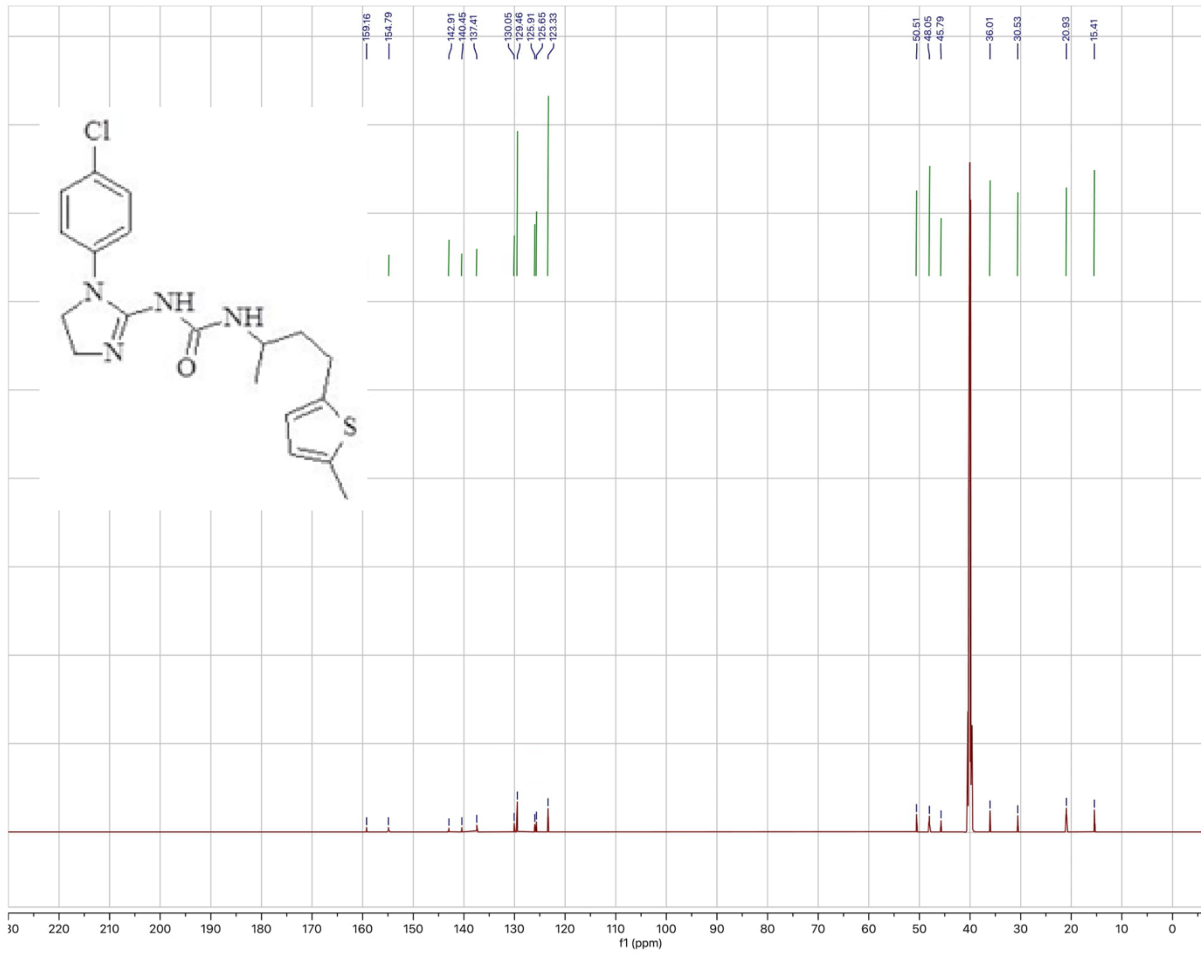

MS spectrum: $C_{19}H_{23}ClN_4OS$ (m.m. calc. 391.1354). HRMS (ESI) *m/z* [M+H]$^+$: 391.1201.

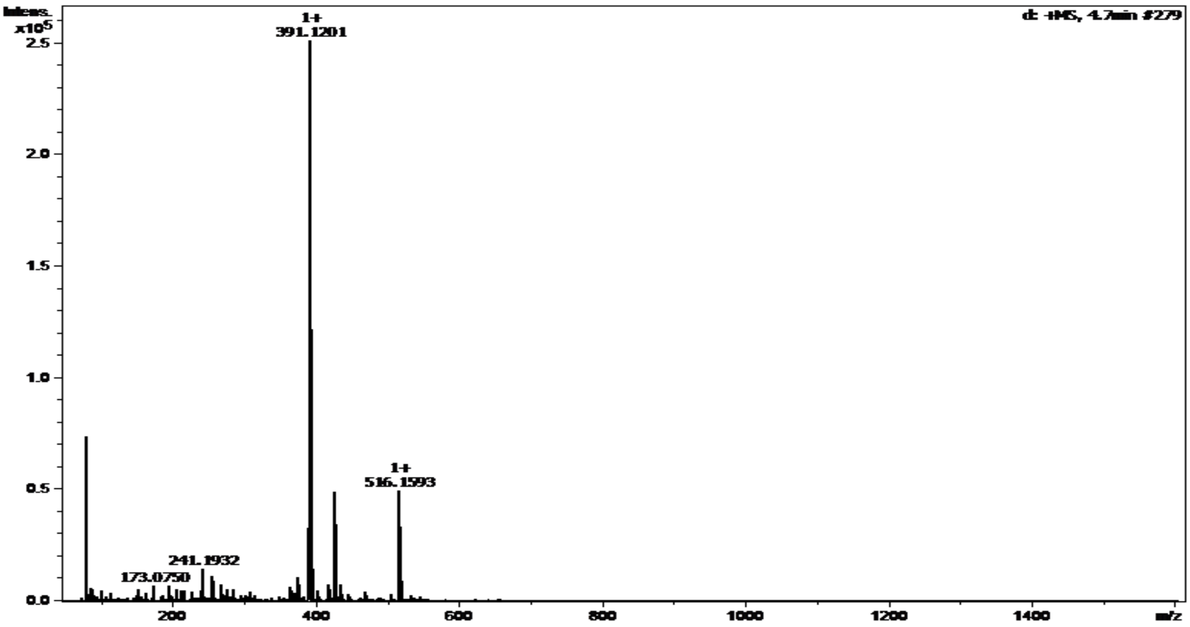

^{1}H NMR spectrum

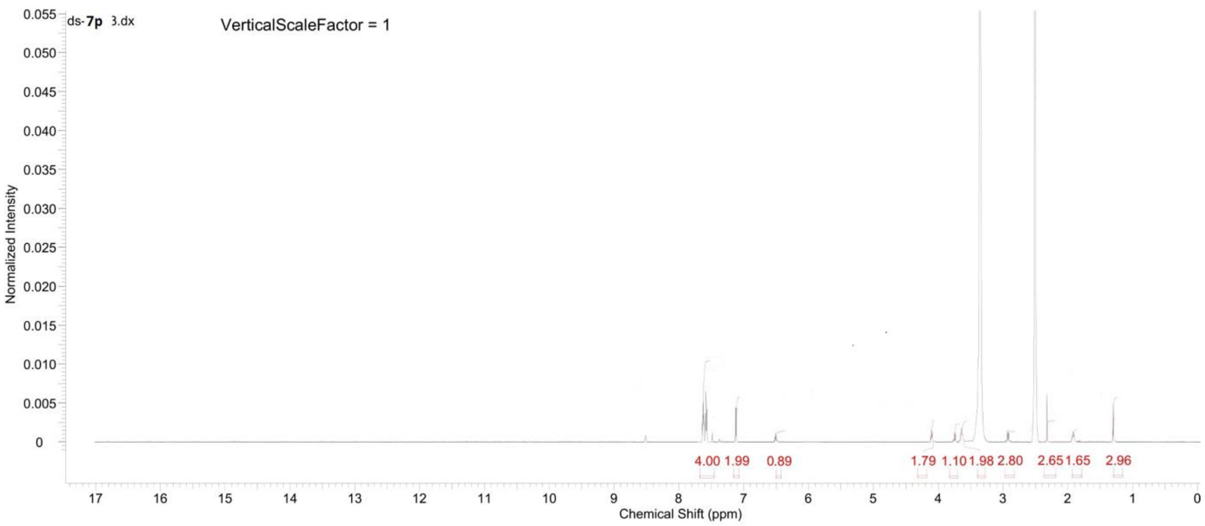

Supplement: Supplementary file 1 [file molecules-29-00571-s001.zip › molecules-2764557-supplementary.pdf]
